# In-Depth Studies of Ground- and Excited-State Properties of Re(I) Carbonyl Complexes Bearing 2,2′:6′,2″-Terpyridine and 2,6-Bis(pyrazin-2-yl)pyridine Coupled with π-Conjugated Aryl Chromophores

**DOI:** 10.1021/acs.inorgchem.1c02151

**Published:** 2021-11-30

**Authors:** Agata Szlapa-Kula, Magdalena Małecka, Anna M. Maroń, Henryk Janeczek, Mariola Siwy, Ewa Schab-Balcerzak, Marcin Szalkowski, Sebastian Maćkowski, Tomasz Pedzinski, Karol Erfurt, Barbara Machura

**Affiliations:** † Institute of Chemistry, University of Silesia, ninth Szkolna Str., 40-006 Katowice, Poland; ‡ Centre of Polymer and Carbon Materials, Polish Academy of Sciences, 34 M. Curie-Sklodowska Str., 41-819 Zabrze, Poland; § Institute of Physics, Faculty of Physics, Astronomy and Informatics, Nicolaus Copernicus University, 5 Grudziadzka Str., 87-100 Toruń, Poland; ∥ Faculty of Chemistry, Adam Mickiewicz University in Poznań, 89b Umultowska, 61-614 Poznań, Poland; ⊥ Department of Chemical Organic Technology and Petrochemistry, Silesian University of Technology, Krzywoustego 4, 44-100 Gliwice, Poland

## Abstract

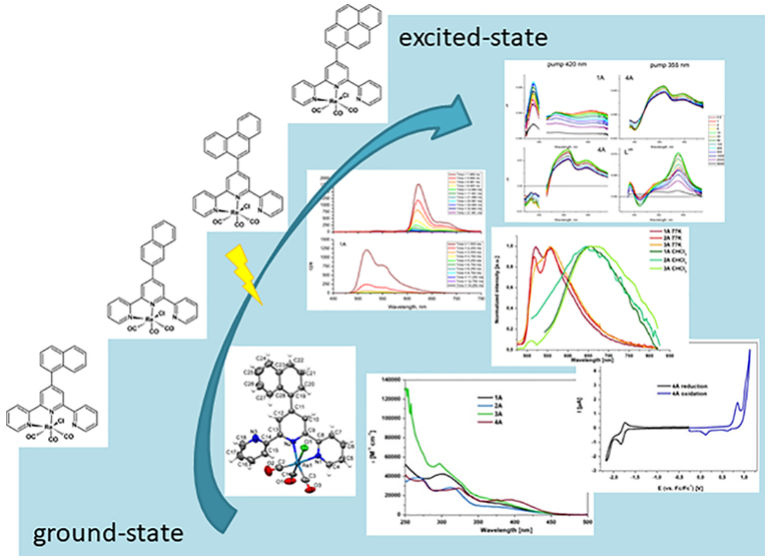

In the current work, comprehensive photophysical and electrochemical studies were performed for eight rhenium(I) complexes incorporating 2,2′:6′,2″-terpyridine (terpy) and 2,6-bis(pyrazin-2-yl)pyridine (dppy) with appended 1-naphthyl-, 2-naphthyl-, 9-phenanthrenyl, and 1-pyrenyl groups. Naphthyl and phenanthrenyl substituents marginally affected the energy of the MLCT absorption and emission bands, signaling a weak electronic coupling of the appended aryl group with the Re(I) center. The triplet MLCT state in these complexes is so low lying relative to the triplet ^3^IL_aryl_ that the thermal population of the triplet excited state delocalized on the organic chromophore is ineffective. The attachment of the electron-rich pyrenyl group resulted in a noticeable red shift and a significant increase in molar absorption coefficients of the lowest energy absorption of the resulting Re(I) complexes due to the contribution of intraligand charge-transfer (ILCT) transitions occurring from the pyrenyl substituent to the terpy/dppy core. At 77 K, the excited states of [ReCl(CO)_3_(L^*n*^-κ^2^*N*)] with 1-pyrenyl-functionalized ligands were found to have predominant ^3^IL_pyrene_/^3^ILCT_pyrene→terpy_ character. The ^3^IL/^3^ILCT nature of the lowest energy excited state of [ReCl(CO)_3_(4′-(1-pyrenyl)-terpy-κ^2^*N*)] was also evidenced by nanosecond transient absorption and time-resolved emission spectroscopy. Enhanced room-temperature emission lifetimes of the complexes [ReCl(CO)_3_(L^*n*^-κ^2^*N*)] with 1-pyrenyl-substituted ligands are indicative of the thermal activation between ^3^MLCT and ^3^IL/^3^ILCT excited states. Deactivation pathways occurring upon light excitation in [ReCl(CO)_3_(4′-(1-naphthyl)-terpy-κ^2^*N*)] and [ReCl(CO)_3_(4′-(1-pyrenyl)-terpy-κ^2^*N*)] were determined by femtosecond transient absorption studies.

## Abstract 2

A series of eight rhenium(I) complexes with 2,2′:6′,2″-terpyridine (terpy) and 2,6-di(pyrazin-2-yl)pyridine (dppy) substituted with 1-naphthyl, 2-naphthyl, 9-phenanthrenyl, and 1-pyrenyl groups was synthesized, and the effect of the tris-heterocyclic core and aryl substituent on selected properties of the obtained [ReCl(CO)_3_(L^*n*^-κ^2^*N*)] was studied in detail. The complexes [ReCl(CO)_3_(L^*n*^-κ^2^*N*)] with 1-pyrenyl-subsituted ligands exhibited greatly enhanced room-temperature photoluminescence lifetimes, consistent with the formation of an equilibrium between the ^3^MLCT and ^3^IL/^3^ILCT excited states.

## Introduction

Transition-metal complexes with 2,2′:6′,2″-terpyridines and their structural analogues have been receiving widespread attention from scientists due to their optical, electrochemical, catalytic, and medicinal properties, making these compounds appealing for potential applications in biological imaging,^1,2^ catalysis,^3−7^ and organic light-emitting devices.^8−10^

Most of the Re(I) carbonyl complexes [ReCl(CO)_3_(L^*n*^-κ^2^*N*)] bearing substituted terpy-like ligands coordinated to the metal center in a bidentate way that have been developed so far^11−20^ emit from the triplet excited state of metal to ligand charge-transfer character (^3^MLCT). Generally, they are weakly emissive at room temperature and have short excited-state lifetimes. Exploring remote substituent effects in [ReCl(CO)_3_(L^*n*^-κ^2^*N*)] with 4′-(4-substituted phenyl)terpyridine ligands, Fernández-Terán and Sévery demonstrated that the introduction of the strongly electron donating −NMe_2_ group leads to the switching from ^3^MLCT to ^3^ILCT (intraligand charge transfer), which is accompanied by significant lengthening of the excited-state lifetime (380 vs 1.5 ns). The obtained [ReCl(CO)_3_(L^*n*^-κ^2^*N*)] with 4′-(4-NMe_2_-phenyl)-2,2′:6′,2″-terpyridine has been successfully used as photosensitizers for hydrogen production, reaching TON_Re_ values of over 2100.^20^ Prolonged lifetimes have also been confirmed for some Re(I) diamine carbonyls [ReX(CO)_3_(phen-TPA)] (X = Cl, Br; TPA = triphenylamine) with the emitting state of ^3^ILCT nature,^21^ and they have been supported for some other terpyridine Re(I) complexes [ReCl(CO)_3_(L^*n*^-κ^2^*N*)] incorporating strong electron-donating substituents by the Wang group^22^ and our group.^14,16,23^

To extend room-temperature triplet excited state lifetimes and improve the photophysical properties of transition-metal complexes, many other strategies have been reported.^24−40^ Among them, there is the bichromophoric approach,^24−31,41−47^ based on the attachment of an organic chromophore with a nonemissive triplet state close in energy to an emissive ^3^MLCT state. Between the ^3^MLCT and ^3^IL states sharing a similar energy, an excited-state equilibrium may be established. In such a case, the organic chromophore repopulates the luminescent ^3^MLCT excited state, playing the role of an energy “reservoir” or excited-state storage element.^24−31,41−47^ Extending luminescence lifetimes via the excited-state equilibration strategy, however, is not accompanied by a quantum yield increase.^28^ The most popular chromophores used for this method are π-conjugated aryl groups: e.g., anthryl and pyrenyl.^24−31,41−47^

**Scheme 1:**
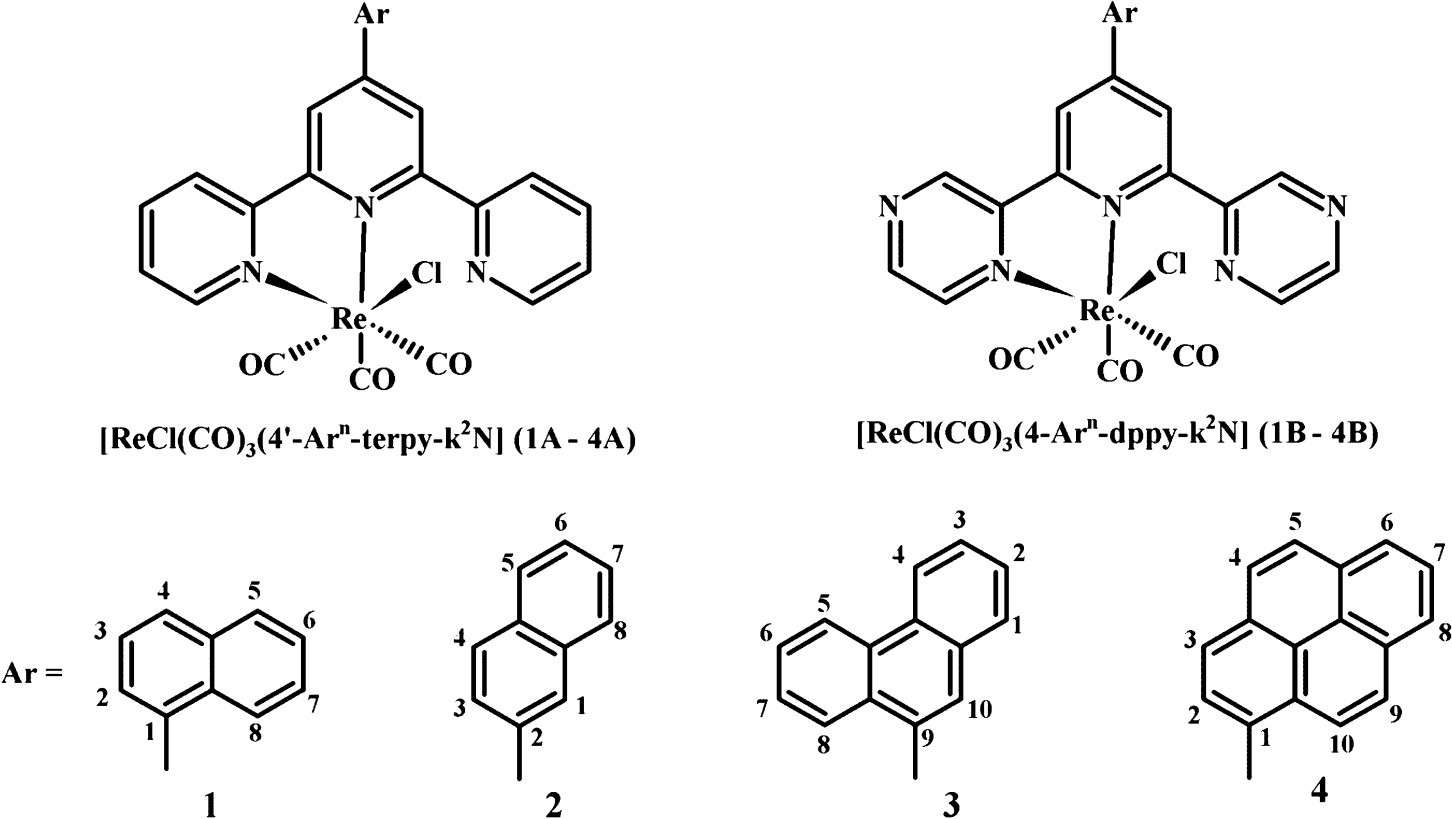
Structures of Rhenium(I) Carbonyl Complexes Studied in the Present Work

In the present work, the ground- and excited-state properties of new Re(I) carbonyl complexes bearing 2,2′:6′,2″-terpyridine (terpy) and 2,6-bis(pyrazin-2-yl)pyridine (dppy) substituted with π-conjugated aryl groups (Scheme 1) were explored by cyclic voltammetry, absorption and emission spectroscopy, and transient absorption, and they were elucidated with the use of density functional theory (DFT) and time-dependent DFT. The attached naphthyl, phenanthrenyl, and pyrenyl substituents differ among themselves in the number of fused rings, and the naphthyl group is attached to the central pyridine ring of the terpy/dppy core via its 1- and 2-positions in order to investigate the effect of the torsional strain due to the inter-ring H···H and hydrogen−π-ring repulsive interactions.

The main emphasis in these studies was placed on the examination of the effect of the π-conjugated aryl substituent and triimine core on the electrochemical and luminescence properties of the resulting [ReCl(CO)_3_(L^*n*^-κ^2^*N*)].

## Results and Discussion

### Synthesis and Characterization

All of the complexes [ReCl(CO)_3_(4′-Ar^*n*^-terpy-κ^2^*N*)] (**1A**–**4A**) and [ReCl(CO)_3_(4-Ar^*n*^-dppy-κ^2^*N*)] (**1B**–**4B**) were obtained by refluxing 1:1 mixtures of [Re(CO)_5_Cl] with the corresponding 4′-Ar^*n*^-terpy or 4-Ar^*n*^-dppy ligand (see details in the Supporting Information). The molecular structures of **1A**–**4A** and **1B**–**4B** were confirmed by ^1^H and ^13^C NMR spectroscopy (Figures S1–S8), the FT-IR technique (Figures S9–S16), elemental analysis, and HR-MS spectrometry (Figures S17–S24). Due to the bidentate coordination mode of 4′-Ar^*n*^-terpy/4-Ar^*n*^-dppy, peripheral ring protons become magnetically distinct and show separate signals in ^1^H NMR spectra, with an integration corresponding to one proton for each peak. For [ReCl(CO)_3_(4′-Ar^*n*^-terpy-κ^2^*N*)] with naphthyl-substituted 2,2′:6′,2″-terpyridines (**1A** and **2A**), the complete signal assignment in the ^1^H and ^13^C NMR spectra was achieved with the aid of the two-dimensional techniques ^1^H–^1^H COSY, ^1^H–^13^C HMQC, and ^1^H–^13^C HMBC (Figures S1 and S2). It is worth noting that the signals of the central pyridine protons of **2A** show significant downfield shifts relative to those recorded for complex **1A**, bearing a more sterically hindered 1-naphthyl unit. The FT-IR spectra of [ReCl(CO)_3_(4′-Ar^*n*^-terpy-κ^2^*N*)] (**1A**–**4A**) and [ReCl(CO)_3_(4-Ar^*n*^-dppy-κ^2^*N*)] (**1B**–**4B**) exhibit a sharp, intense C≡O stretching band (2025–2019 cm^–1^) and two poorly resolved bands in a lower energy range (1936–1875 cm^–1^), which is consistent with a facial geometry of CO ligands in the moiety {Re(CO)_3_}^+^. The increase in the average value of CO stretching frequencies of [ReCl(CO)_3_(4-Ar^*n*^-dppy-κ^2^*N*)] (1957 cm^–1^ for **1B**, 1948 cm^–1^ for **2B**, 1943 cm^–1^ for **3B**, and 1950 cm^–1^ for **4B**) in relation to that for [ReCl(CO)_3_(4′-Ar^*n*^-terpy-κ^2^*N*)] (1943 cm^–1^ for **1A**, 1944 cm^–1^ for **2A**, 1937 cm^–1^ for **3A**, and 1938 cm^–1^ for **4A**) is indicative of a weaker donor capacity of the 4-Ar^*n*^-dppy ligand in comparison to the corresponding 4′-Ar^*n*^-terpy ligand.^48^

The molecular structure of **1A** has been additionally determined by an X-ray analysis. The coordination sphere of Re(I) in **1A** is best described as a highly distorted octahedron (Figure S25), and it has a small bite angle N(2)–Re(1)–N(1) of 74.38(10)°, an increase in the C(2)–Re(1)–N(2) angle to above 90° (100.80(17)°), and elongation of Re–N_central pyridine_ (2.218(3) Å) relative to Re–N_peripheral pyridine_ (2.164(3) Å). With reference to the related systems,^10−19^ these structural features are mainly induced by the κ^2^*N* coordination of 4′-Ar^*n*^-terpy and strong steric repulsion between the noncoordinated pyridine and C(2)–O(2) group. The noncoordinated pyridyl ring is inclined to the central pyridine plane at 48.17°, while the dihedral angle between the 1-naphthyl and central pyridine planes is 38.53°. Additional structural data of **1A**, along with the thermal properties of [ReCl(CO)_3_(4′-Ar^*n*^-terpy-κ^2^*N*)] (**1A**–**4A**) and [ReCl(CO)_3_(4-Ar^*n*^-dppy-κ^2^*N*)] (**1B**–**4B**) are available in Tables S2–S6 in the Supporting Information.

### Electronic Structure Calculations

To get a better understanding of the effect of aromatic groups (Ar) on the electronic structures of [ReCl(CO)_3_(4′-Ar^*n*^-terpy-κ^2^*N*)] and [ReCl(CO)_3_(4-Ar^*n*^-dppy-κ^2^*N*)], calculations at the DFT/PBE0/def2-TZVPD/def2-TZVP level were performed for all molecules **1A**–**4A** and **1B**–**4B** (see Table S7 and Figures S27–S30 in the Supporting Information).

**Figure 1:**
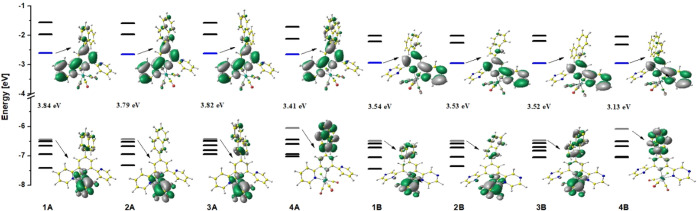
Partial molecular orbital energy level diagrams for **1A**–**4A** and **1B**–**4B**.

The partial molecular orbital energy-level diagrams for **1A**–**4A** and **1B**–**4B**, together with the plots of the frontier molecular orbitals of [ReCl(CO)_3_(4′-Ar^*n*^-terpy-κ^2^*N*)] and [ReCl(CO)_3_(4-Ar^*n*^-dppy-κ^2^*N*)] are shown in Figure 1.

For both series **1A**–**4A** and **1B**–**4B**, the LUMO is negligibly affected by the aryl group introduced into terpy and dppy cores. Its energy varies from −2.64 eV to −2.60 eV for [ReCl(CO)_3_(4′-Ar^*n*^-terpy-κ^2^*N*)] (**1A**–**4A**) and from −2.95 eV to −2.94 eV for [ReCl(CO)_3_(4-Ar^*n*^-dppy-κ^2^*N*)] (**1B**–**4B**). The replacement of peripheral pyridyl rings in 4′-Ar^*n*^-terpy by pyrazinyl rings in 4-Ar^*n*^-dppy results in the stabilization of the LUMO energy levels of [ReCl(CO)_3_(4-Ar^*n*^-dppy-κ^2^*N*)] relative to [ReCl(CO)_3_(4′-Ar^*n*^-terpy-κ^2^*N*)] by ∼0.3 eV. For all of the molecules **1A**–**4A** and **1B**–**4B**, the LUMO resides predominately on the coordinated rings of terpy/dppy cores.

The HOMO of **1A**–**3A** and **1B**–**3B** is largely located on the {Re(CO)_3_Cl} unit, and its energy is almost independent of the attached aryl group. The replacement of the peripheral pyridyl rings in 4′-Ar^*n*^-terpy by pyrazinyl ones in 4-Ar^*n*^-dppy results in a negligible stabilization of the HOMO energy levels of [ReCl(CO)_3_(4-Ar^*n*^-dppy-κ^2^*N*)] relative to [ReCl(CO)_3_(4′-Ar^*n*^-terpy-κ^2^*N*)], by ∼0.05 eV. The HOMO–LUMO gap across the series **1A**–**3A** varies marginally from 3.79 eV (**2A**) to 3.84 eV (**1A**), and it is comparable to the values reported previously for [ReCl(CO)_3_(terpy-κ^2^*N*)] (3.89 eV) and [ReCl(CO)_3_(4′-Ph-terpy-κ^2^*N*)] (3.82) eV^14,23^ (Figure S29). Similarly to the terpyridine Re(I) complexes **1A**–**3A**, the HOMO–LUMO gap of **1B**–**3B** is insensitive to the attached aryl group (3.54 eV for **1A** and **2A** and 3.52 eV for **3A**). In relation to [ReCl(CO)_3_(4′-Ar^*n*^-terpy-κ^2^*N*)], however, it becomes noticeably smaller as a result of the stabilization of the LUMO energy level in [ReCl(CO)_3_(4-Ar^*n*^-dppy-κ^2^*N*)].

In contrast to **1A**–**3A** and **1B**–**3B**, the HOMO of **4A** and **4B** is principally localized on the pyrenyl group attached to the terpy/dppy core, and it is effectively destabilized relative to the HOMO levels of **1A**–**3A** and **1B**–**3B**, respectively. In both series, an increase in the HOMO energy level of **4A** and **4B** is ∼0.4 eV. This leads to a noticeable decrease in the HOMO–LUMO gap in the case of **4A** (3.41 eV) and **4B** (3.13 eV) in comparison to that of other complexes [ReCl(CO)_3_(4-Ar^*n*^-dppy-κ^2^*N*)] and [ReCl(CO)_3_(4′-Ar^*n*^-terpy-κ^2^*N*)] investigated within this work.

The calculations also indicate that the attachment of the pyrenyl to the terpy/dppy core leads to a noticeable decrease in ionization potentials (IPs) of **4A** and **4B** in relation to **1A**–**3A** and **1B**–**3B**, respectively. Across the series **1A**–**3A** and **1B**–**3B**, calculated IP values vary marginally depending on the aryl substituent. In turn, the triimine core was found to affect electronic affinities (EAs), and the replacement of the peripheral pyridyl rings in 4′-Ar^*n*^-terpy by pyrazinyl rings in 4-Ar^*n*^-dppy results in an increase in EA values (Table S8).

### Electrochemistry

The cyclic voltammetry (CV) and differential pulse voltammetry (DPV) were performed to experimentally estimate the HOMO and LUMO energy levels of [ReCl(CO)_3_(4′-Ar^*n*^-terpy-κ^2^*N*)] and [ReCl(CO)_3_(4-Ar^*n*^-dppy-κ^2^*N*)] complexes (**1A**–**4B** and **1B**–**4B**). The relevant electrochemical data of **1A**–**4A** and **1B**–**4B** are gathered in Table 1 and Table S9, while Figure S31 shows the CVs and DPVs for **1A**–**4A** and **1B**–**4B**. The ferrocene/ferrocenium couple was used as the reference redox couple. The values of IP and EA, which are closely related to HOMO and LUMO levels, were estimated from the first oxidation and reduction waves, respectively.^49^

**Table 1:** Electrochemical Data for Compounds **1A**–**4A** and **1B**–**4B**

compound	*E*_1red_^onset^ (V)	*E*_1ox_^onset^ (V)	IP^a^ (CV)	EA^b^ (CV)	*E*_g(CV)_^c^ (eV)	*E*_g(OPT)_^d^ (eV)
**1A**	–1.67	0.73	–5.83	–3.43	2.40	2.62
**2A**	–1.63	0.67	–5.77	–3.47	2.30	2.62
**3A**	–1.67	0.66	–5.76	–3.43	2.33	2.65
**4A**	–1.65	0.71	–5.81	–3.45	2.36	2.67
**1B**	–1.32	0.96	–6.06	–3.78	2.28	2.42
**2B**	–1.46	0.90	–6.00	–3.64	2.36	2.40
**3B**	–1.35	0.83	–5.93	–3.75	2.18	2.42
**4B**	–1.29	0.92	–6.02	–3.81	2.21	2.47

a IP = −5.1 – *E*_ox_.

b EA = −5.1 – E_red_.

c *E*_g(CV)_ = *E*_ox_^onset^ – *E*_red_^onset^.

d *E*_g(OPT)_ = 1240/λ_onset._

As shown in Table 1, the differences in the values of *E*_1ox_^onset^ for [ReCl(CO)_3_(4′-Ar^*n*^-terpy-κ^2^*N*)] and [ReCl(CO)_3_(4-Ar^*n*^-dppy-κ^2^*N*)] were less than 0.07 and 0.13 V, respectively. Also, *E*_1red_^onset^ values fall in a narrow range, from −1.63 to −1.67 V for **1A**–**4A** and from −1.29 to −1.46 V for **1B**–**4B**. Upon the replacement of the pyridyl by pyrazinyl, the first reduction wave moves into a more positive region. The complexes **1B**–**4B** become easier to reduce but more difficult to oxidize relative to **1A**–**4A**. It can be safely assumed that the reduction in **1A**–**4A** and **1B**–**4B** occurs in the triimine core. As supported by DFT studies, the LUMO of these systems is largely contributed by π* orbitals of the terpy/dppy core, and additional N atoms in the peripheral rings (dppy) result in lowering of the LUMO energy level of Re(I) complexes bearing 2,6-bis(pyrazin-2-yl)pyridine derivatives, which is manifested in the shift of the first reduction peaks of **1B**–**4B** toward less negative potentials. The first oxidation waves of **1A**–**3A** and **1B**–**3B** most probably correspond to the Re(I)-based oxidation process Re(I)/Re(II). As reported in refs (50 and 51), the first oxidation of the free Ar^1–3^-terpy and Ar^1–3^-dppy was found to occur at significantly higher potentials in relation to the corresponding Re(I) complexes **1A**–**3A** and **1B**–**3B**. The values of *E*_1ox_^onset^ of **4A** and **4B** are comparable with those for **1A**–**3A** and **1B**–**3B**, and they are also consistent with those reported for the free terpy and dppy functionalized with 1-pyrenyl.^50,51^ On the basis of the DFT results, showing that the HOMO of **4A** and **4B** is principally localized on the pyrenyl group attached to the terpy/dppy core, it can be suggested that the oxidation in **4A** and **4B** occurs in the pyrene core.

### Absorption Spectroscopy and TD-DFT Calculations

**Figure 2:**
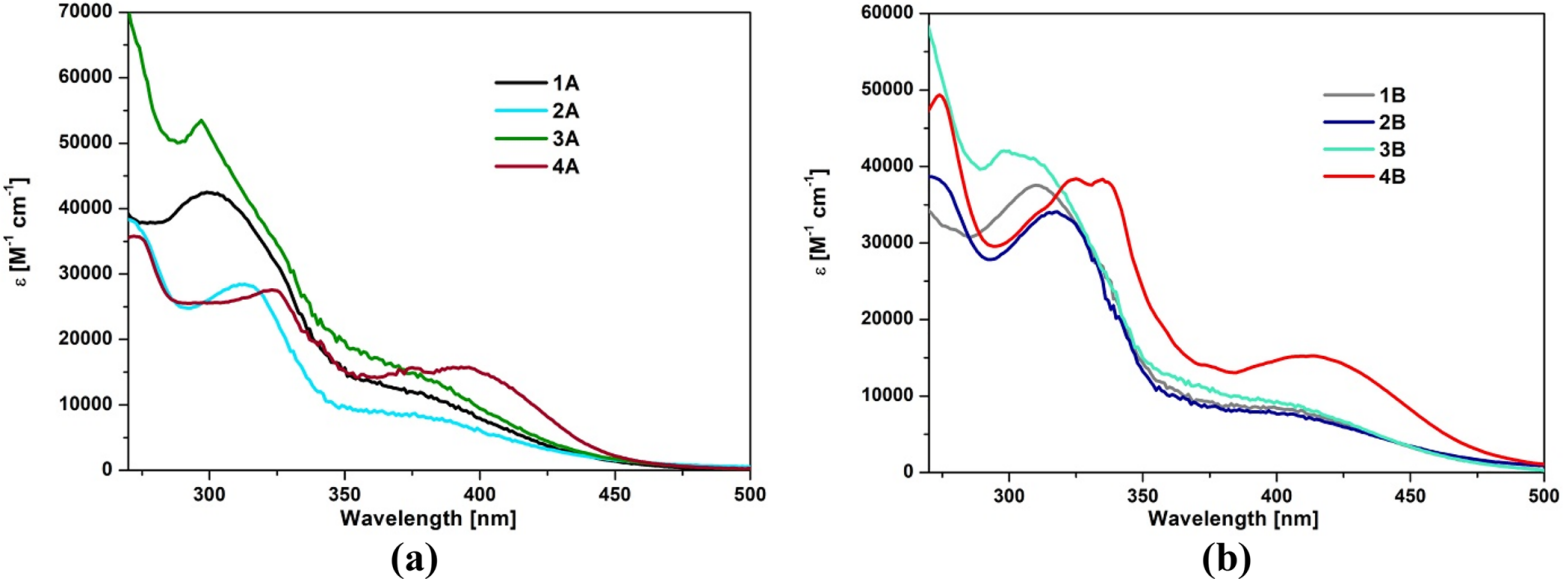
UV–vis absorption spectra of **1A**–**4A** (a) and **1B**–**4B** (b) in acetonitrile.

The electronic absorption spectra of **1A**–**4A** and **1B**–**4B** were recorded in acetonitrile and chloroform and in thin films on a glass substrate (Figure 2 and Figures S32–S35). The spectroscopic data are summarized in Table S10. All of the complexes [ReCl(CO)_3_(4′-Ar^*n*^-terpy-κ^2^*N*)] (**1A**–**4A**) and [ReCl(CO)_3_(4-Ar^*n*^-dppy-κ^2^*N*)] (**1B**–**4B**) exhibited absorption properties of typical [ReX(CO)_3_(diimine)]^0/+^ chromophores, with intense bands below 350 nm due to the organic ligand π → π* transitions and moderate absorption in the range 350–480 nm attributed to the electronic transitions of charge-transfer (CT) character (Figure 2).

From **1A**–**4A** to **1B**–**4B**, the low-energy band experiences a bathochromic shift (Figure S34), which can be rationalized by stronger electron-withdrawing abilities of Ar-dppy due to the presence of additional nitrogen atoms in the peripheral rings, leading to a decrease in the LUMO energy level of [ReCl(CO)_3_(4-Ar^*n*^-dppy-κ^2^*N*)] in relation to [ReCl(CO)_3_(4′-Ar^*n*^-terpy-κ^2^*N*)]. In both **1A**–**4A** and **1B**–**4B** series, the attachment of the pyrenyl group to the central pyridine ring of the terpy or dppy core leads to a noticeable red shift of the lowest energy absorption of the resulting Re(I) complexes, while the effect of naphthyl and phenanthrenyl units on the position of the lowest-energy absorption of [ReCl(CO)_3_(4-Ar^*n*^-dppy-κ^2^*N*)] and [ReCl(CO)_3_(4′-Ar^*n*^-terpy-κ^2^*N*)] is rather marginal. In comparison to [ReCl(CO)_3_(terpy-κ^2^*N*)] and [ReCl(CO)_3_(4′-Ph-terpy-κ^2^*N*)]/[ReCl(CO)_3_(4-Ph-dppy-κ^2^*N*)], there is a noticeable intensity increase of the visible light absorption for **1A**–**4A** and **1B**–**4B**, which can be attributed to the introduction of extended aryl groups into the terpy or dppy core^14,23^ (Figure S32). With an increase in the solvent polarity, the CT absorption of **1A**–**4A** to **1B**–**4B** experiences a hypsochromic shift, typical of rhenium(I) tricarbonyl diimine complexes with a metal to ligand charge-transfer (MLCT) absorption band^52^ (Table S10 and Figure S34).

Given these observations, that is, a blue shift of the low-energy band of **1A**–**4A** to **1B**–**4B** with increasing solvent polarity and higher light absorption in the case of **4A** to **4B**, the low-energy absorption of **1A**–**3A** and **1B**–**3B** is most likely of MLCT character, while the visible part of the electronic spectra of **4A** and **4B** is most probably a combination of ILCT and MLCT transitions. Such an assignment is in agreement with the TD-DFT calculations presented in Table S11 and Figure S36.

For **1A**–**3A** to **1B**–**3B**, the dominant calculated transition for the lowest energy absorption band is the S_0_ → S_2_ excitation, which corresponds to charge transfer from the {Re(CO)_3_Cl} moiety to the π* orbital of the 4′-Ar^*n*^-terpy or 4-Ar^*n*^-dppy ligand. The same MLCT character can be assigned to the excitations S_0_ → S_1_ appearing at the red end of the visible absorption band of **1A**–**3A** to **1B**–**3B** and S_0_ → S_3_ (**2A**, **2B**) and S_0_ → S_4_ (**1A**, **3A**, **1B**, **3B**) making a contribution to the blue edge of the lowest-lying absorption band. The excitations S_0_ → S_3_ (**1A**, **3A**, **1B**, **3B**) and S_0_ → S_4_ (**2A**, **2B**) are IL (intraligand) with an admixture of MLCT in nature, and they contribute to the blue edge of the lowest-lying absorption band. In comparison to the S_0_ → S_2_ transition, all they have much lower oscillator strengths.

For complexes **4A** and **4B**, the electronic excitations S_0_ → S_2_, S_0_ → S_3_, and S_0_ → S_4_ (**4A**) and S_0_ → S_3_ and S_0_ → S_4_ (**4B**) conserve the MLCT character. In contrast, the transitions S_0_ → S_1_ and S_0_ → S_5_ (**4A**) and S_0_ → S_1_, S_0_ → S_2_, and S_0_ → S_5_ (**4B**) are intraligand and MLCT in nature. The significant increase in their oscillator strengths can be assigned to the contribution of intraligand charge-transfer (ILCT) transitions originating from charge delocalization from the pyrenyl group to the terpy/dppy acceptor core.

### Luminescence Studies

**Figure 3:**
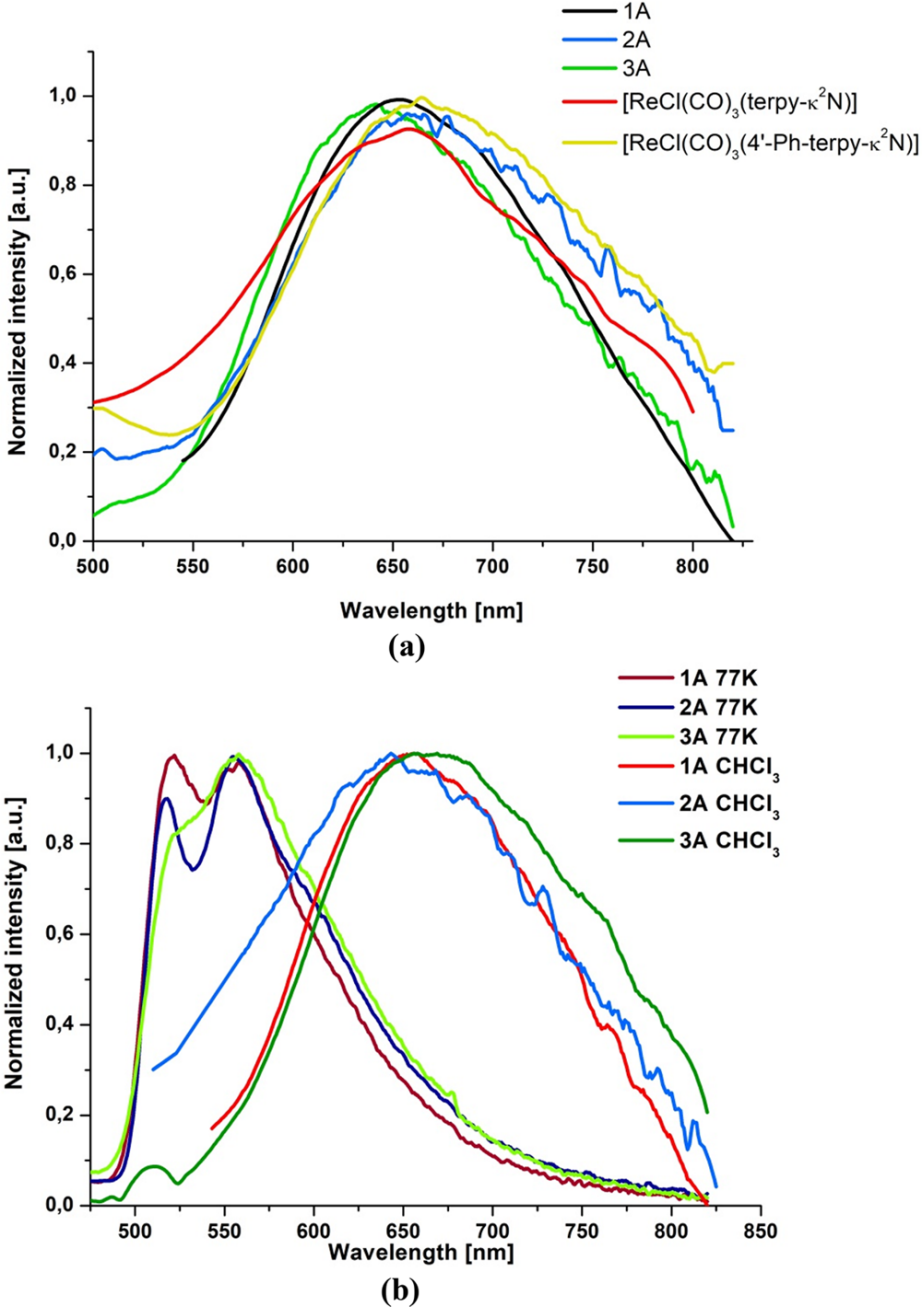
(a) Normalized emission spectra of **1A**–**3A** in CH_3_CN in comparison to the emission spectra of [ReCl(CO)_3_(terpy-κ^2^*N*)] and [ReCl(CO)_3_(4′-Ph-terpy-κ^2^*N*)] in CH_3_CN. Reproduced from refs (14 and 23). Copyright John Wiley and Sons 2018 and Royal Society of Chemistry 2020, respectively. (b) Normalized emission spectra of **1A**–**3A** in CHCl_3_ and a rigid matrix at 77 K.

**Figure 4:**
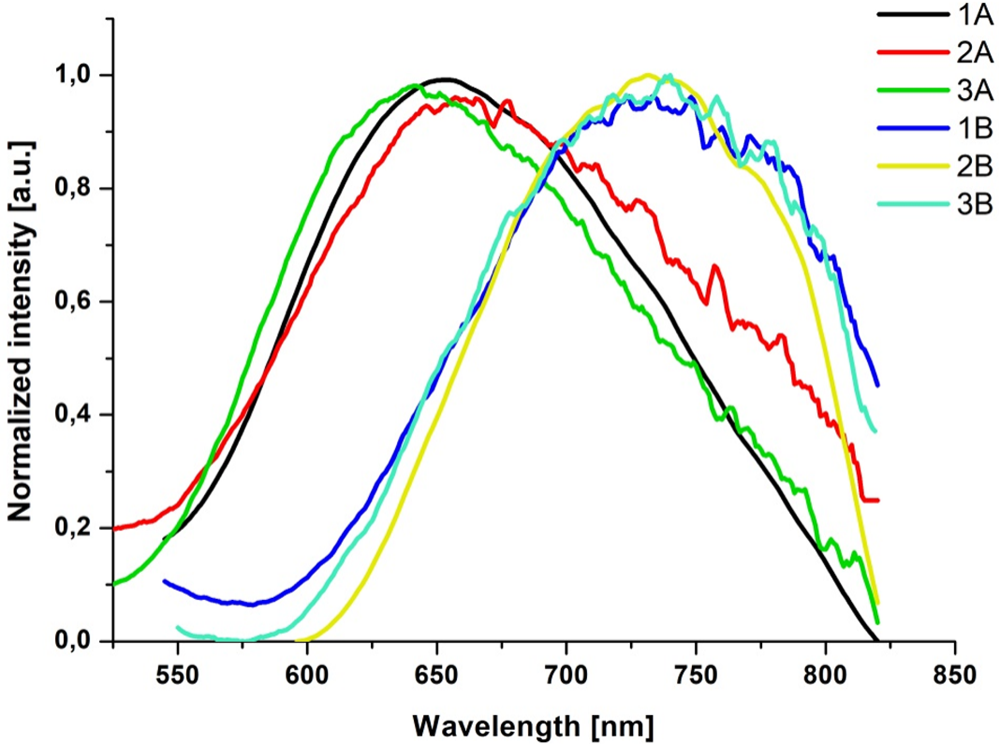
Normalized emission spectra of **1B**–**3B** compared to the emission spectra of **1A**–**3A** in CH_3_CN.

**Figure 5:**
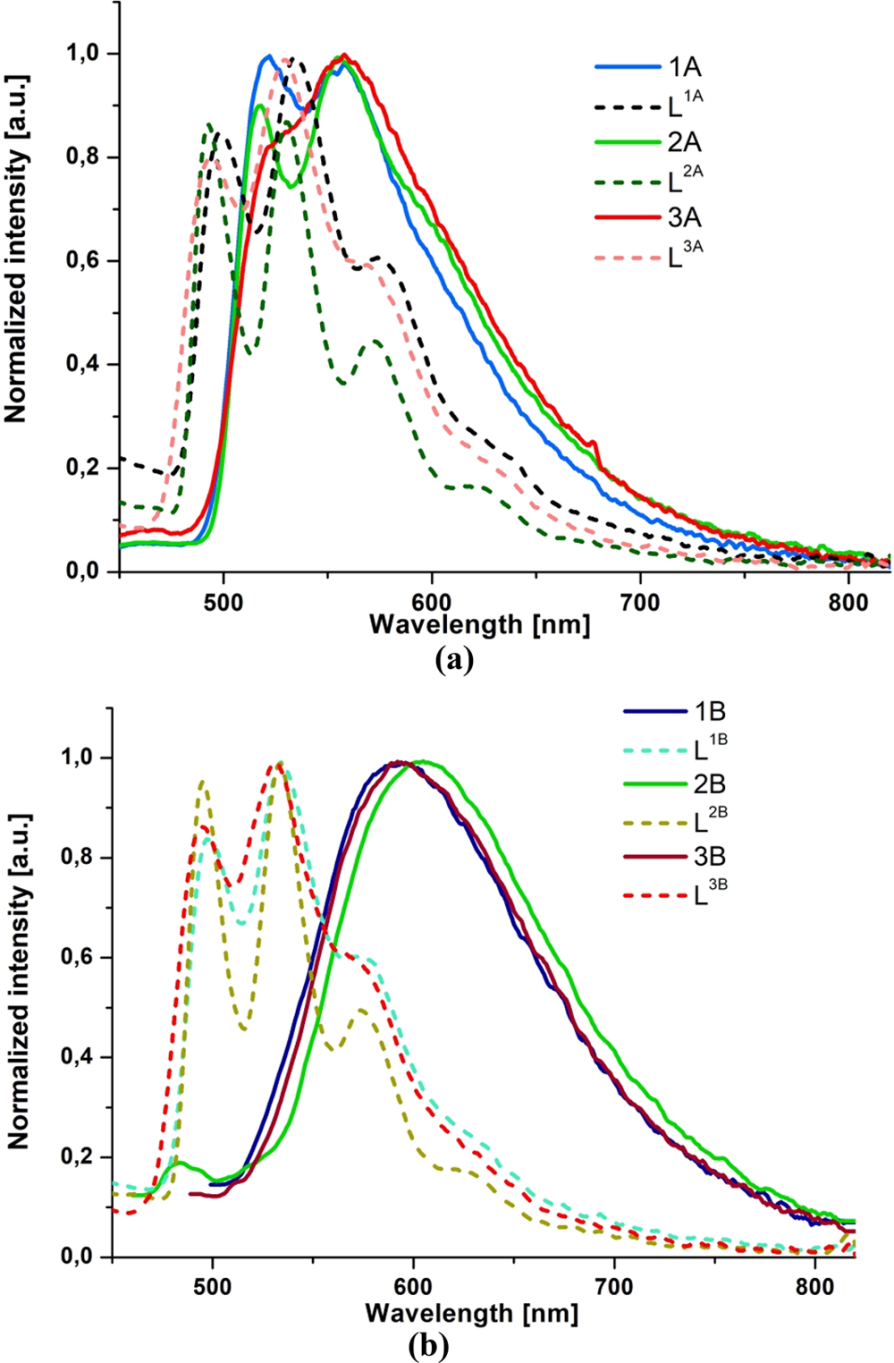
(a) Phosphorescence spectra of **1A**–**3A** versus the phosphorescence spectra of the free ligands at 77 K. (b) Phosphorescence spectra of **1B**–**3B** versus the phosphorescence spectra of the free ligands at 77 K. The triplet ligand emissions were induced by addition of 10% ethyl iodide and recorded at 77 K in BuCN.

**Figure 6:**
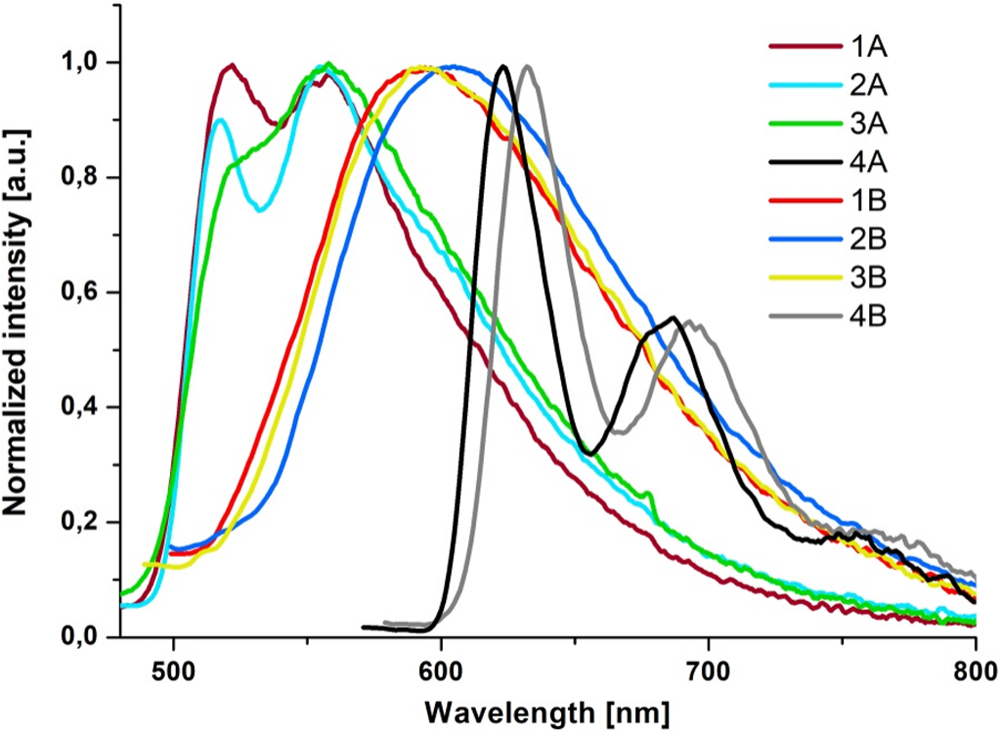
Comparison of low-temperature emission spectra of **4A** and **4B** with low-temperature emission spectra of **1A**–**3A** to **1B**–**3B**.

**Figure 7:**
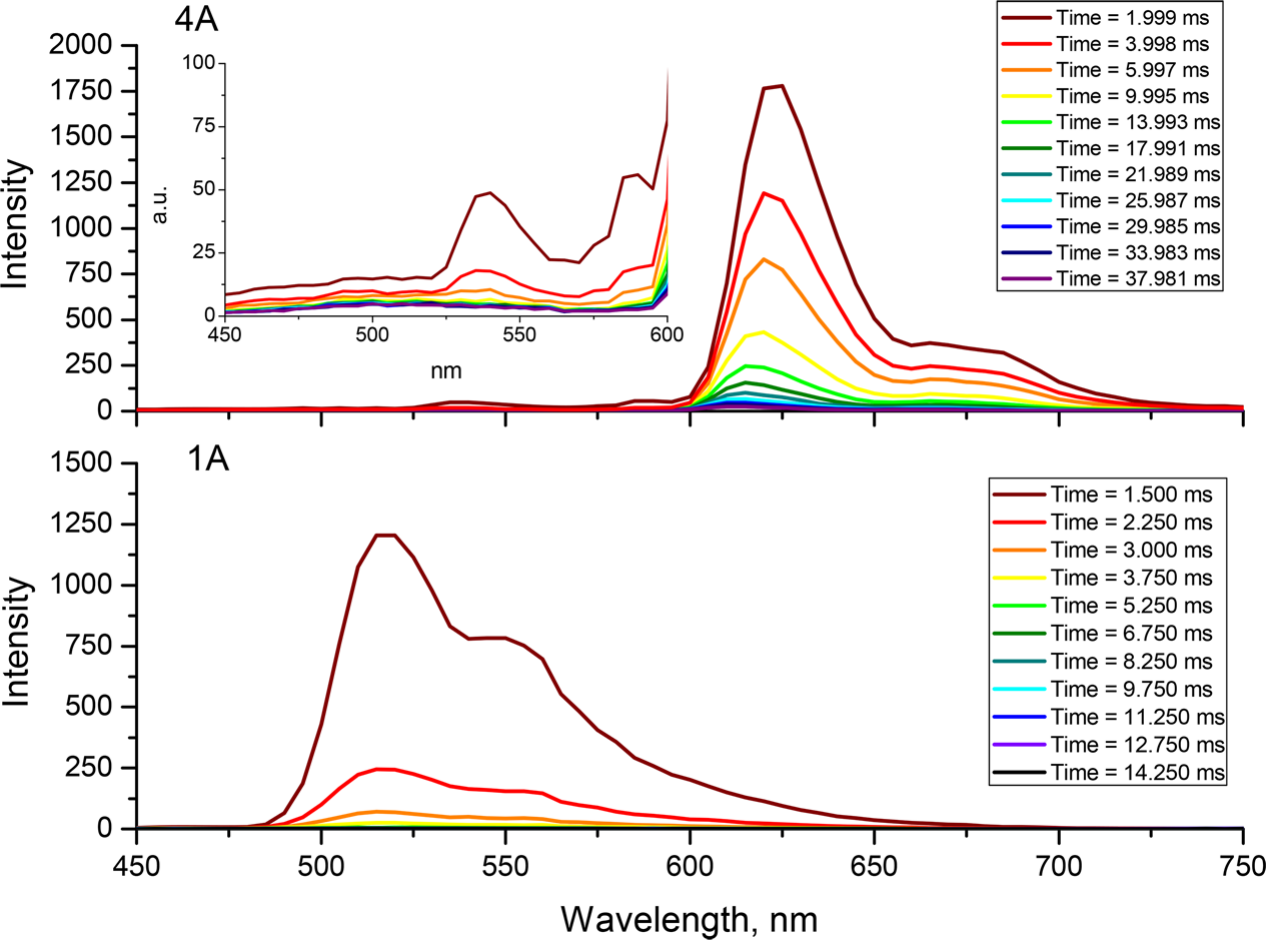
Time-resolved emission spectrum of **4A** and **1A** at 77 K excited at 395 nm.

**Figure 8:**
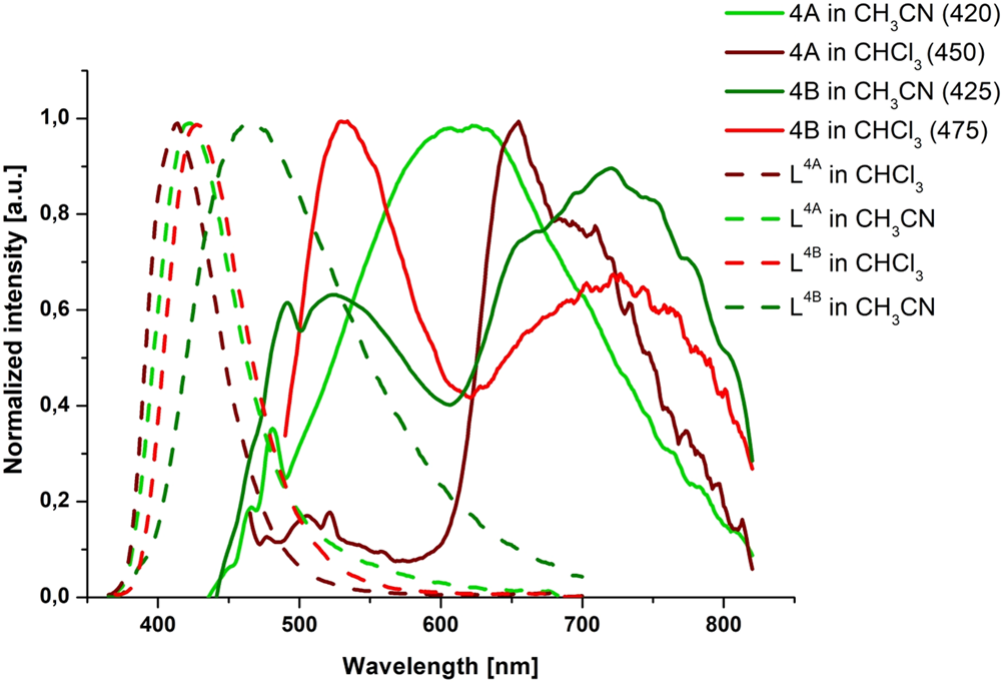
Normalized room-temperature emission spectra of the free ligands and complexes **4A** and **4B** in CH_3_CN and in CHCl_3_. Excitation wavelengths are given in parentheses. Reproduced from refs (50 and 51). Copyright Elsevier 2020 and 2021.

**Figure 9:**
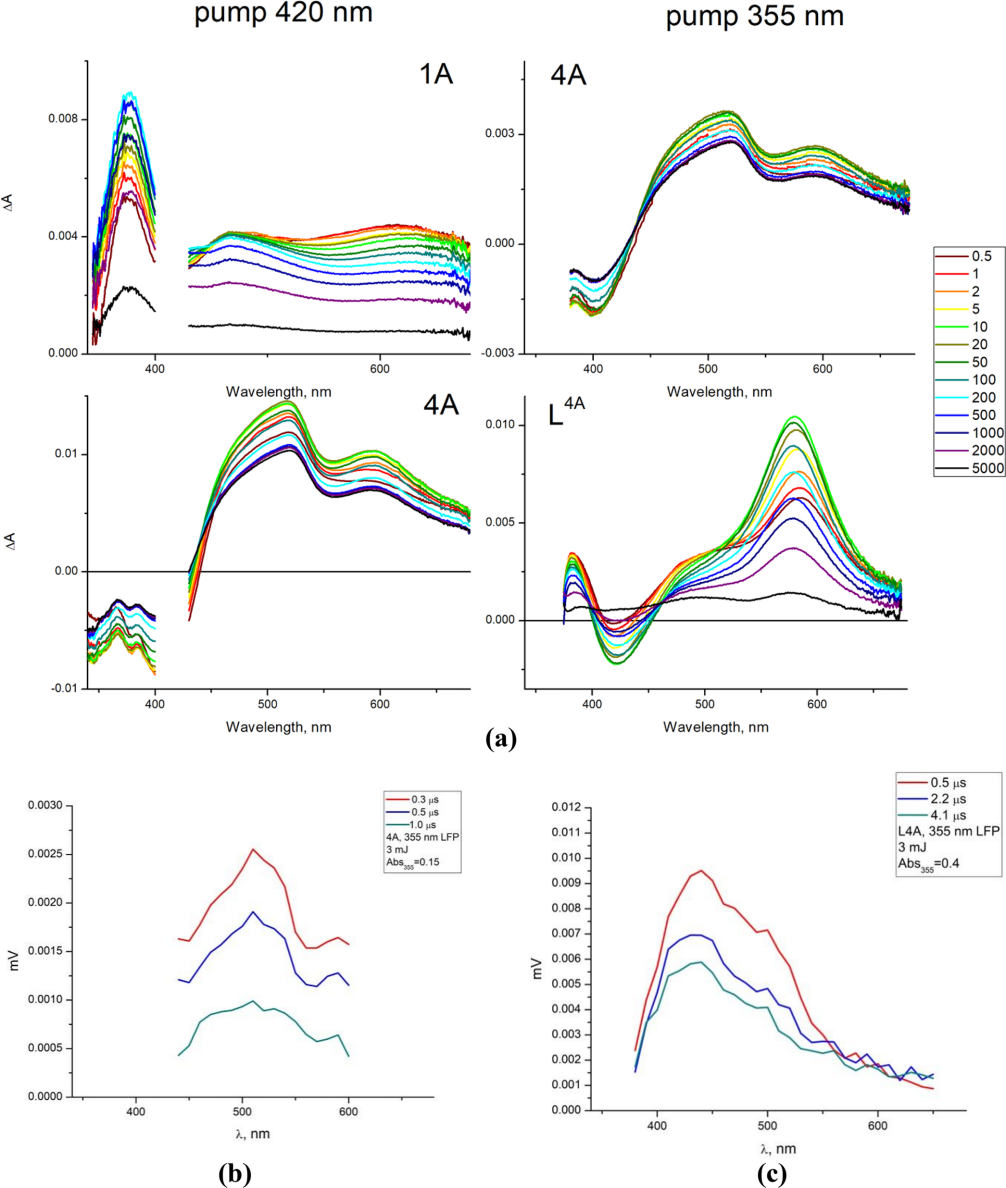
(a) fsTA spectra of **1A**, **4A**, and **L**^**4A**^ measured using 420 and 355 nm pump light: energies per pulse equal to 0.18 μJ (**1A**), 0.20 μJ (**4A**, 420 nm), 0.20 (**4A**, 355 nm), 0.26 μJ (**L**^**4A**^); delay time window 0.5–5000 ps. (b) nsTA spectra of **4A**. (c) nsTA spectra of **L**^**4A**^.

The photoluminescence (PL) properties of the synthesized Re(I) complexes were investigated in two solvents of different polarities (CHCl_3_, ε = 4.8; CH_3_CN, ε = 37.5), in a rigid matrix at 77 K (BuCN), and in the solid state as powders and thin films. The emission spectral data of **1A**–**4A** and **1B**–**4B** are gathered in Table 2. The normalized emission spectra of the synthesized Re(I) complexes upon photoexcitation at the lowest absorption band are shown in Figures 3–9 and Figures S37–S58 in the Supporting Information.

**Table 2:** Summary of Photoluminescence Properties of Complexes **1A**–**4A** and **1B**–**4B**^a^

	CH_3_CN				CHCl_3_				solid				BuCN (77 K)		
	λ_exc_ (nm)	λ_em_ (nm)	τ_av_ (ns)	φ (%)	λ_exc_ (nm)	λ_em_ (nm)	τ_av_ (ns)	φ (%)	λ_exc_ (nm)	λ_em_ (nm)	τ_av_ (ns)	φ (%)	λ_exc_ (nm)	λ_em_ (nm)	τ_av_ (μs)
**1A**	410	654	4.5	1.9	420	660	6.5	7.7	420	628	162.8	4.8	420	522, 558	249.2
**2A**	405	663	4.0	1.1	450	645	6.1	9.8	400	578	102.2	1.6	420	518, 556	95.6
**3A**	410	641	3.1	0.6	440	665	4.2	6.8	370	619	13.0	0.6	420	522 sh, 558	104.6
**4A**	420	620	3.3	6.1	450	500	4.65	<0.1	nd	nd			440	627, 680, 756	5738.7
						650, 696 sh	4.404	7.6							
**1B**	400	750	3.4	2.4	480	730	4.5	6.9	440	651	55.9	9.7	400	597	2.1
**2B**	385	680	3.5	1.8	480	730	4.9	10.4	440	666	63.1	10.7	440	600	2.3
**3B**	440	737	3.3	5.0	480	736	4.4	6.4	435	642	78.9	9.8	410	595	2.2
**4B**	425	520	4.0	0.3	475	532	8.2	0.5	nd	nd			450	632, 694, 770	1933.9
		708	122.9	4.4		730	130.9	10.9							

a τ_av_ denotes the average lifetime. Experimental errors for emission lifetimes are given in Figure S37 in the Supporting Information.

In solution, the maximum emission energies of **1A**–**3A** are similar to each other and resemble those for [ReCl(CO)_3_(terpy-κ^2^*N*)] and [ReCl(CO)_3_(4′-Ph-terpy-κ^2^*N*)],^14,23^ implying that the emitting state in these complexes is of the same origin, and it is only slightly affected by the aryl group attached to the terpy core (Figure 3a and Table 2). All of these complexes exhibit broad and structureless steady-state emission spectra with maxima in the narrow range 641–663 nm for CH_3_CN and 645–665 nm for CHCl_3_, with lifetimes in the nanosecond domain and rather low emission quantum yields. From the solution at room temperature to the solid state and matrix at 77 K, the emission of **1A**–**3A** exhibits a hypsochromic shift (Figure 3b). Such behavior is typical for ^3^MLCT excited states. As the rigidity of the medium increases and solvent reorganization decreases, the ^3^MLCT excited state is destabilized and consequently the excited- to ground-state energy gap is increased, which is reflected in a blue shift of the emission and leads to a significant decrease in the nonradiative decay rate constant.^53−55^

The photoluminescence spectra of **1B**–**3B** also originated from ^3^MLCT states at room temperature. All of these compounds exhibit broad and nonstructured emission bands with maxima in the range 680–750 nm in solution, 651–666 nm in the solid state, and 595–600 nm at 77 K, short lifetimes in solution, and longer lifetimes in the solid state and a rigid matrix at 77 K. In comparison to **1A**–**3A**, the emission energies of **1B**–**3B** are significantly red shifted, consistent with the LUMO energy stabilization and decrease in HOMO–LUMO energy gap upon replacing the terpy core by the dppy core (Figure 4).

Another striking difference between **1A**–**3A** and **1B**–**3B** concerns spectral profiles and excited-state lifetimes of the emission at 77 K. The frozen-state emission bands of **1B**–**3B** remain broad and nonstructured, clearly indicating that they still originate from ^3^MLCT states. As shown in Figure 5, the frozen-state emissions of **1B**–**3B** occur at significantly lower energies relative to the phosphorescence of the free ligands. For **1A**–**3A** at 77 K, the emission band shows a vibrionic structure, and the triplet emission of **1A**–**3A** largely overlaps with the phosphorescence of the free ligands (Figure 5). A larger contribution of ^3^IL in Re(I) complexes with naphthyl- and phenanthrenyl-substituted terpyridine ligands is also manifested in a noticeable increase of emission lifetimes of **1A**–**3A** relative to **1B**–**3B** (Table 2). The replacement of the terpy core by the dppy core results in an increase in the energy gap between ^3^MLCT and ^3^IL in **1B**–**3B.**

The ^3^MLCT emission nature of **1A**–**3A** was additionally confirmed by theoretical results. The energies of the theoretical phosphorescence emission, corresponding to the difference Δ*E*_T_1_–S_0__, reproduce the experimental data well, differing from the experimental values by 0.09–0.13 eV.

The spin density surface plots generated from the T_1_ states of complexes **1A**–**3A** (Table S12) depict spin density to be localized on the {Re(CO)_3_Cl} unit and π* orbitals of the pyridine rings coordinated to the Re(I) center, supporting that the lowest energy triplet state in these complexes is ^3^MLCT.

The introduction of the pyrene ring into the terpy/dppy core substantially affects the photophysical properties of the Re(I) complexes (**4A** and **4B**). Low-temperature (77 K) emission spectra of **4A** and **4B** resemble each other. They appear at lower energy in relation to those for **1A**–**3A** to **1B**–**3B** and show a well-resolved vibronic structure (Figure 6). As the frozen-state emission of **4A** and **4B** falls in the range of the phosphorescence of the free ligands and pyrene, and it is significantly red shifted relative to the emission of the parent Re(I) complexes, we can assume that their emission at 77 K occurs predominately from the excited state of ^3^IL_pyrene_ with an admixture of ^3^ILCT_pyrene→terpy_ character (Figures S50 and S51). Such an assignment is supported by lifetimes in milliseconds, 1–3 orders of magnitude longer than those for **1A**–**3A** to **1B**–**3B** chromophores at 77 K (Table 2). Also, spin density surface plots generated from the T_1_ states of complexes **4A** and **4B** illustrate that the spin density is localized on the pyrene and central pyridine of terpy/dppy (Table S13).

To further examine the nature of the lowest triplet state of [ReCl(CO)_3_(L^*n*^-κ^2^*N*)] with pyrene-substituted ligands, time-resolved emission spectra of **4A** and **4B** at 77 K were recorded (Figure 7 and Figures S61–S64). For both complexes, the presence of two components was revealed. The higher energy emission in the range below 600 nm closely resembles the ^3^MLCT emission spectrum at low temperature (77 K) for **1A** and **1B**. The longer lifetime of the structured component with a maximum at 620 nm is typical of the organic chromophore phosphorescence, and it can be assigned to ^3^IL_pyrene_ with a small admixture of ^3^ILCT_pyrene→terpy_. These findings allow us to conclude that an intramolecular energy transfer from the ^3^MLCT excited state to the ^3^IL_pyrene_/^3^ILCT_pyrene→trimine_ state occurs in **4A** and **4B**.

The room-temperature photoluminescence spectra of **4A** and **4B** in solution are shown in Figure 8. The emission spectra of deaerated solutions of **4A** in CHCl_3_ and **4B** in CH_3_CN and CHCl_3_ are composed of two well-separated bands, as reported previously for other bichromophoric systems.^47,56^ The emission peak at longer wavelengths is quenched upon exposure to air (Figure S58), demonstrating that it arises from a triplet state. The possibility that the emission peak at shorter wavelength is due to the free ligand that has dissociated from the complex can be excluded. The examined Re(I) complexes show stability and photostability in the media used (Figure S59 and S60), and the high-energy band of **4A** in CHCl_3_ and **4B** in CH_3_CN and CHCl_3_ is bathochromically shifted relative to the emission of the appropriate free ligand (Figure 8). As shown in Figures S52–S55, the singlet ligand-centered excited state is promoted by higher energy excitation, and the ratio of the fluorescence and phosphorescence is dependent on the excitation wavelength. In comparison to the free ligand, however, the complex fluorescence is clearly quenched, suggesting the occurrence of energy transfer from ^1^IL/^1^ILCT to ^1^MLCT via a Förster resonance energy transfer (FRET) mechanism (Figures S56 and S57).

In the case of the deaerated solution **4A** in CH_3_CN, the prevailing emission originates from the singlet excited state. To better understand the differences between **4A** in CH_3_CN and **4A** in CHCl_3_, the efficiency of the energy transfer from ^1^IL/^1^ILCT to ^1^MLCT occurring through the FRET mechanism was calculated using the equation , where QY_**4A**_ is the quantum yield of the fluorescence emanating from the **L**^**4A**^ ligand in the complex **4A**, while QY_L^**4A**^_ is the quantum yield of the free ligand.^47^ Noticeably less efficient Förster energy transfer was found for **4A** in CH_3_CN (87%) in comparison to **4A** in CHCl_3_ (99%). According to ref (47), this residual fluorescence in the acetonitrile solution of **4A** is sufficient to conceal the triplet emission (see also Figures S58 and S64). The overlap of the phosphorescence with much stronger fluorescence prevented us from determining the phosphorescence lifetime of **4A** in CH_3_CN (Table 2). For **4A** in CHCl_3_, due to the greater FRET efficiency (99%), both fluorescence and phosphorescence were observed.^47^

Most remarkably, the room-temperature phosphorescence lifetimes of **4A** in CHCl_3_ and **4B** in CH_3_CN/CHCl_3_ are 2–3 orders of magnitude longer than those for **1A**–**3A** to **1B**–**3B** chromophores. Such a significant prolongation of excited-state lifetimes indicates the thermal activation between closely lying ^3^MLCT and ^3^IL/^3^ILCT. The pyrene chromophore repopulates the luminescent ^3^MLCT excited state and acts as an excited-state storage element, resulting in an extension of the ^3^MLCT emission. The longer lifetime of **4A** in relation to **4B** seems to be a result of the greater energy separation between the triplet states ^3^MLCT and ^3^IL_pyrene_/^3^ILCT_pyrene→trimine_ in the case of **4A** (Figure 6) and thus the larger contribution of the lowest excited state ^3^IL_pyrene_/^3^ILCT_pyrene→trimine_ with a much longer lifetime.^47^ However, it should be noted that the phosphorescence lifetimes of **4B** in CH_3_CN/CHCl_3_ may be affected to some extent by the presence of the greater residual fluorescence in the case of **4B** in comparison to **4A** in CHCl_3_ (Figure 6 and Figures S61–S64).

The solid-state emission is changed upon the replacement of naphthyl and phenanthrenyl substituents by the pyrenyl substituent. In contrast to **1A**–**3A** and **1B**–**3B**, the solid-state emission of **4A** and **4B** is completely quenched, which can be assigned to aggregation-caused quenching (Table 2). Preliminary investigations of the PL ability of Re(I) complexes in film blends are given in Table S14 and Figures S65–S68 in the Supporting Information.

### Femto- and Nanosecond Transient Absorption

The excited-state dynamics and the nature of the lowest triplet state of **4A** were investigated using transient absorption spectroscopy in femtosecond (fsTA) and nanosecond (nsTA) regimes, and the results for **4A** were compared to those for the free ligand **L**^**4A**^ and **1A**.

**Figure 10:**
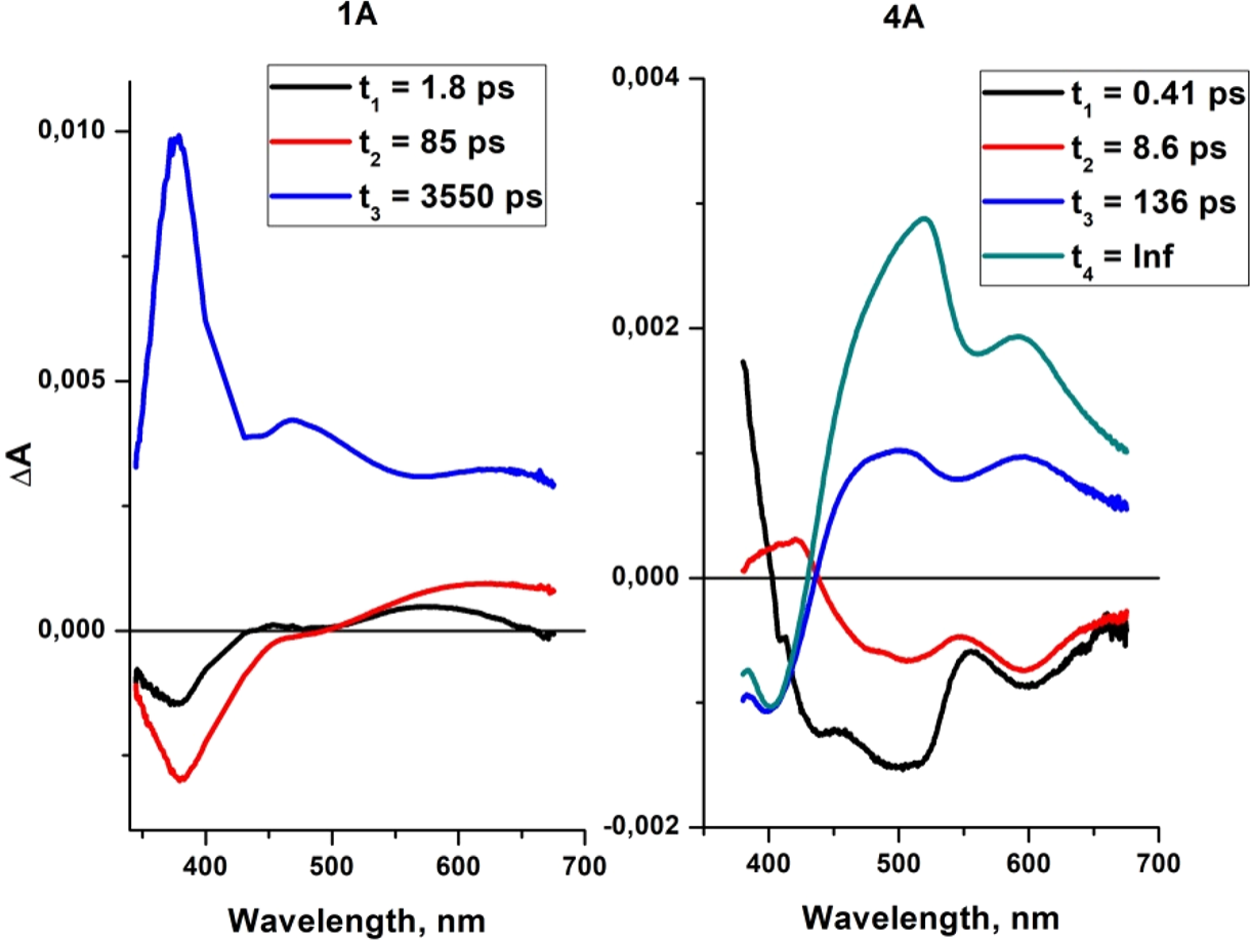
Decay-associated spectra of **1A** and **4A**.

The fsTA spectra of **1A** display only positive features across the UV and visible region (Figure 9). According to refs (57−59), they are indicative of ^3^MLCT excited-state absorptions. The intense band at 376 nm is typical of the absorption of the bipyridine anion radical bipy^•–^, whereas the excited-state absorption (ESA) in the visible part is attributed to Cl/L^•–^ → Re (ligand to metal charge transfer, LMCT) transitions. Due to the high similarity between **1A** and previously reported [ReCl(CO)_3_(terpy-κ^2^*N*)]^18^ and [ReCl(CO)_3_(bipy)],^57−59^ we can safely state that the excitation of **1A** produces the ^1^MLCT state, which undergoes femtosecond intersystem crossing (ISC) and simultaneously populates an intermediate π → π* intraligand state (^3^IL) and a vibrationally hot ^3^MLCT state. The conversion of the ^3^IL excited state into the ^3^CT state occurs on a picosecond time scale. Such an assignment is supported by a global lifetime analysis, given in Figure S69 in the Supporting Information. Three components, revealed by the global lifetime analysis and characterized by time constants *t*_*i*_, are attributed to the conversion of the intermediate state ^3^IL to ^3^MLCT, vibrational relaxation of the lowest triplet state ^3^CT, and ground-state recovery times, respectively (Figure 10). The ultrafast intersystem crossing occurs in a time range (∼140 fs) shorter than the internal response function (IRF = 175 fs).

The fsTA spectra of **4A** collected upon excitation at 355 nm, populating predominately the ^1^ILCT state, are characterized by a clear ground-state bleaching (GSB) at the wavelength ∼400 nm and excited-state absorption (ESA) with two discernible maxima at 517 and 594 nm. The GSB is in accordance with ^1^MLCT/^1^ILCT of **4A**, and it covers the range of the fluorescence of the **L**^**4A**^ (due to their overlapping) (see Figure 9 and Figures S70–S72). The high similarity between the line shapes of the relaxed excited state of **4A** in the fsTA regime and spectra in nsTA allows us to safely assume that no additional excited states occur between the femtosecond and nanosecond time scales. The spectral similarities in nsTA spectra between the complex **4A** and **L**^**4A**^ indicate that the lowest triplet state of **4A** is of ^3^IL_pyrene_/^3^ILCT_pyrene→terpy_ character, while a strong resemblance of the temporal evolution of the transient absorption spectra of **4A** excited at 355 nm and those measured for **4A** upon excitation of 420 nm allows us to assume that **4A** follows the same photophysical pathway on excitation at 355 and 420 nm (Figure 9).

**Figure 11:**
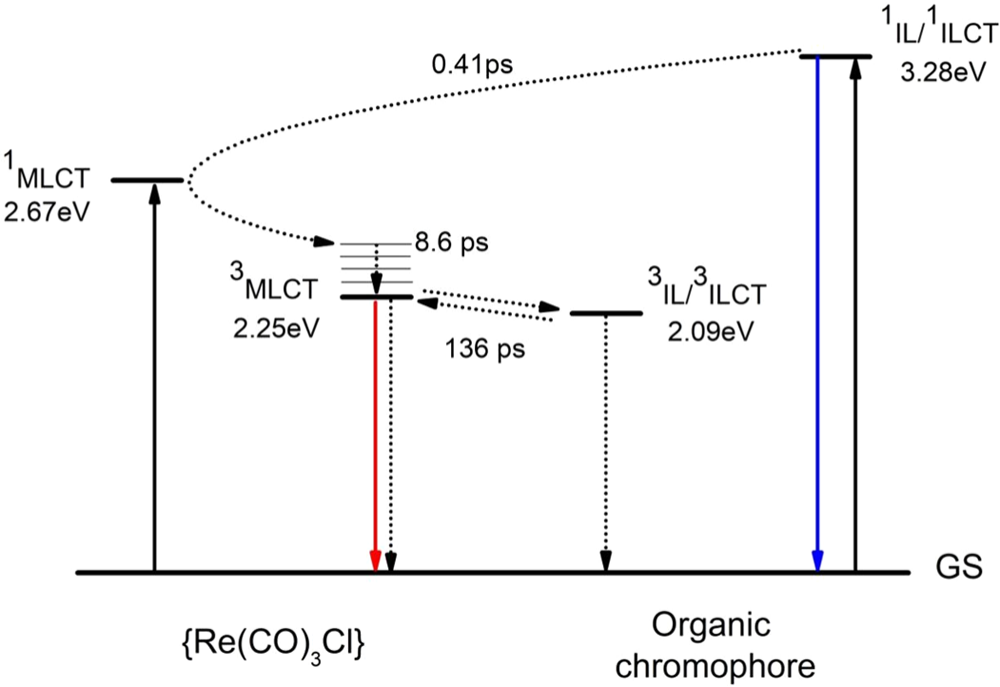
Schematic energy level diagram with the deactivation pathway proposed for **4A**.

The proposed energy level diagram of **4A** with the deactivation pathway, demonstrated in Figure 11, is well supported by the global lifetime analysis of **4A** (Figure 11).

A global lifetime analysis confirmed that the fsTA data of **4A** are best described in terms of four components, each characterized by a time constant *t*_*i*_ (Figure 10 and Figure S70 and S71). Upon excitation at 355 nm, the excited state ^1^**L**^4A*^ is formed (DAS_1_), and the molecules undergo energy transfer through the FRET mechanism to the ^1^MLCT* state, which promptly undergoes intersystem crossing (ISC) to the ^3^MLCT* (*t*_1_ = 0.41 ps). The DAS_2_ is negative across the whole visible spectral region, reflecting the increase in the 400–700 nm excited state, which corresponds to the relaxed ^3^MLCT state. The *t*_2_ relaxation is due to reorganization processes occurring within a supramolecular moiety comprising the Re(I) chromophore and local solvent molecules, also including rotation about the pyrene–terpy bond. The relaxed ^3^MLCT state undergoes triplet–triplet energy transfer into ^3^IL/^3^ILCT localized on **L**^**4A**^ (*t*_3_ = 136 ps). DAS_4_ corresponds to the absorption spectrum of the fully relaxed lowest triplet state ^3^IL/^3^ILCT.

## Conclusions

A series of eight rhenium(I) complexes with 2,2′:6′,2″-terpyridine (terpy) and 2,6-bis(pyrazin-2-yl)pyridine (dppy) substituted with 1-naphthyl, 2-naphthyl, 9-phenanthrenyl, and 1-pyrenyl groups was synthesized, and the effect of the tris-heterocyclic core and aryl substituent on selected properties of the obtained [ReCl(CO)_3_(L^*n*^-κ^2^*N*)] was studied in detail. All complexes exhibited high melting temperatures above 200 °C, and they can form amorphous materials with very high *T*_g_ values. The ligands functionalized with naphthyl and phenanthrenyl units were found to have a rather marginal effect on the electrochemical and optical properties of the resulting Re(I) complexes. In contrast, the introduction of the pyrenyl group to the central pyridine ring of the terpy or dppy core resulted in an increase in the molar absorption coefficients and led to a noticeable bathochromic shift of the lowest energy absorption of [ReCl(CO)_3_(L^*n*^-κ^2^*N*)] due to the large involvement of ^1^ILCT. With regard to the emission properties, the complexes [ReCl(CO)_3_(L^*n*^-κ^2^*N*)] with 1-pyrenyl-subsituted ligands exhibited greatly enhanced room-temperature photoluminescence lifetimes, consistent with the formation of an equilibrium between the ^3^MLCT and ^3^IL/^3^ILCT excited states. The deactivation pathway occurring upon the light excitation in [ReCl(CO)_3_(4′-(1-naphthyl)-terpy-κ^2^*N*)] and [ReCl(CO)_3_(4′-(1-pyrenyl)-terpy-κ^2^*N*)] was determined by femtosecond transient absorption studies. The excitation of the first complex produces the ^1^MLCT state, which undergoes femtosecond intersystem crossing (ISC) and simultaneously populates an intermediate π → π* intraligand state (^3^IL) and a vibrationally hot ^3^MLCT state. The conversion of the ^3^IL excited state into the ^3^CT state occurs on a picosecond time scale. In the case of [ReCl(CO)_3_(4′-(1-pyrenyl)-terpy-κ^2^*N*)], upon excitation at 355 an 420 nm, the singlet excited state pyrenyl-terpy* is formed, which undergoes an energy transfer through the FRET mechanism to the ^1^MLCT* state, being further transformed to the ^3^MLCT* by ISC. The relaxed ^3^MLCT state undergoes triplet–triplet energy transfer into ^3^IL/^3^ILCT localized on the pyrenyl-terpy ligand.

## Experimental Section

The synthesis and characterization of the Re(I) carbonyl complexes are provided in the Supporting Information. Elemental analysis was recorded on a Vario EL Cube apparatus. NMR spectra were recorded on a Bruker Avance 400 NMR spectrometer in DMSO-*d*_6_ solution. IR spectra were measured using a Nicolet iS5 FTIR spectrophotometer (KBr). High-resolution mass spectrometry analyses were performed on a Waters Xevo G2 Q-TOF mass spectrometer (Waters Corporation) equipped with an ESI source operating in positive-ion mode. Single-crystal X-ray diffraction data were collected on a Gemini A Ultra diffractometer (Mo Kα),^18^ and crystallographic data for **1A** were deposited with the Cambridge Crystallographic Data Center (CCDC 2094604). Differential scanning calorimetry (DSC) studies were carried out with the use of a TA-DSC 2010 apparatus under nitrogen atmosphere, with a heating rate of 20 °C/min. Electrochemical measurements were performed using an Eco ChemieAutolab PGSTAT128n potentiostat.^18^ The electronic spectra were registered on a ThermoScientific Evolution 220 UV/vis spectrometer. The photoluminescence was obtained on a Hitachi F-2500 spectrometer or an FLS-980 fluorescence spectrophotometer.^18^ Femtosecond transient absorption spectra were measured using a Helios pump–probe transient absorption spectrometer (Ultrafast Systems).^18^ Nanosecond transient absorption spectra were recorded according to the procedure given previously.^18^ The calculations were performed using the GAUSSIAN-16 program package.^60^ More experimental details are given in the Supporting Information.

## Supporting Information Available

Selected experimental details, NMR (^1^H, ^13^C, and 2D), IR, and HR-MS spectra, molecular structure plot and crystal data for **1A**, thermal properties, DFT and TD-DFT calculations, electrochemical data, UV–vis spectra, luminescence spectra, spin density surface plots of selected Re(I) complexes, stability and photostability of **4A** and **4B**, time-resolved emission spectra for **1A**, **4A** ,and **4B** in CHCl_3_ and CH_3_CN and at 77 K, PL spectra of **1A** and **4A** in a PVK:PBD matrix, and nano- and femtosecond transient absorption spectra of **1A** and **4A** (PDF)

## Accession Codes

CCDC 2094604 contains the supplementary crystallographic data for this paper. These data can be obtained free of charge via www.ccdc.cam.ac.uk/data_request/cif, or by emailing data_request@ccdc.cam.ac.uk, or by contacting The Cambridge Crystallographic Data Centre, 12 Union Road, Cambridge CB2 1EZ, UK; fax: +44 1223 336033.
