# Supplementary material for: In-Depth Studies of Ground- and Excited-State Properties of Re(I) Carbonyl Complexes Bearing 2,2′:6′,2″-Terpyridine and 2,6-Bis(pyrazin-2-yl)pyridine Coupled with π-Conjugated Aryl Chromophores
Source: Inorg Chem. 2021 Nov 30;60(24):18726–38. doi: 10.1021/acs.inorgchem.1c02151 (PMC8693190; doi:10.1021/acs.inorgchem.1c02151)
Supplement: Supplementary file 1 — ic1c02151_si_001.pdf [file ic1c02151_si_001.pdf]

Supporting Information for the manuscript:

In-depth studies of ground- and excited-state properties of Re(I) carbonyl complexes bearing 2,2′:6′,2″-terpyridine and 2,6-bis(pyrazin-2-yl)pyridine coupled with π-conjugated aryl chromophores

Agata Szlapa-Kula [†], *Magdalena Małecka* [†], *Anna M. Maroń* [†], *Henryk Janeczek*[‡], *Mariola Siwy*[‡], *Ewa Schab-Balcerzak*[‡], *Marcin Szalkowski* [ǀ], *Sebastian Maćkowski* [ǀ], *Tomasz Pedzinski*[§], *Karol Erfurt*[‖], *Barbara Machura* [†*]

[†]Institute of Chemistry, University of Silesia, 9th Szkolna Str., 40-006 Katowice, Poland

[‡]Centre of Polymer and Carbon Materials, Polish Academy of Sciences, 34 M. Curie-Sklodowska Str., 41-819 Zabrze, Poland

[ǀ]Institute of Physics, Faculty of Physics, Astronomy and Informatics, Nicolaus Copernicus University, 5 Grudziadzka Str., 87-100 Toruń, Poland

[§]Faculty of Chemistry, Adam Mickiewicz University in Poznań, 89b Umultowska, 61-614 Poznań, Poland

[‖]Department of Chemical Organic Technology and Petrochemistry, Silesian University of Technology, Krzywoustego 4, 44-100 Gliwice, Poland

Table of Contents

1. Experimental Details:<br>General procedure for the synthesis of rhenium(I) carbonyl complexes<br>General characterization of Re(I) carbonyl complexes<br>Film and device preparation<br>HR-MS measurements<br>Crystal structure determination and refinement<br>Computational studies<br>Electrochemistry<br>Steady-state and time-resolved emission spectroscopy<br>Electroluminescence spectra measurements<br>Nanosecond transient absorption spectroscopy<br>Photodamage test and fluence analysis of solution samples for fs studies<br>Femtosecond transient absorption spectroscopy	
2. Fluence dependence and photo-stability tests of **1A**, **4A** and **L^{4A}**	Table S1
3. ^{1}H NMR and ^{13}C NMR spectra of **1A–4A** and **1B-4B**	Figure S1–S8
4. IR spectra of **1A–4A** and **1B-4B**	Figure S9–S16
5. HR-MS spectra of **1A–4A** and **1B-4B**	Figure S17–S24
6. The molecular structure of **1A** with thermal ellipsoids set at 50% probability for non-hydrogen atoms	Figure S25
7. Crystal data and structure refinement of the complex **1A**	Table S2
8. Bond lengths [Å] and bond angles for **1A**	Table S3
9. Short π•••π interactions in the crystal structure of **1A**	Table S4
10. X—Y•••Cg(J)(π-ring) interactions for **1A**	Table S5
11. Thermal properties of Re(I) complexes	Table S6
12. DSC thermograms for the complex 2A	Figure S26
13. Experimental and theoretical bond lengths [Å] and angles [°] for **1A**	Table S7
14. Selected molecular orbitals of the complexes **1A**–**4A** along with their percentage composition	Figure S27
15. Selected molecular orbitals of the complexes **1B**–**4B** along with their percentage composition	Figure S28
16. The partial molecular orbital energy level diagrams for **1A**–**4A** compared to [ReCl(CO)$_3$(terpy-κ^{2}N)] and [ReCl(CO)$_3$(4′-Ph-terpy-κ^{2}N)], along with the plots of the frontier molecular orbitals of [ReCl(CO)$_3$(4′-Ar^n-terpy-κ^{2}N)]	Figure S29
17. The partial molecular orbital energy level diagrams for **1B**–**4B** compared to [ReCl(CO)$_3$(terpy-κ^{2}N)] and [ReCl(CO)$_3$(4′-Ph-terpy-κ^{2}N)], along with the plots of the frontier molecular orbitals of [ReCl(CO)$_3$(4′-Ar^n-terpy-κ^{2}N)]	Figure S30
18. Calculated ionization potentials and electron affinities (vertical and adiabatic), energy gap, as well as hole and electrons reorganization energies and extraction potentials (DFT/PBE1PBE/def2-TZVPD/def2-TZVP) for **1A**–**4A** and **1B**–**4B**	Table S8
19. Cyclic voltammetry (CV, right side) and differential pulse voltammetry (DPV, left side) of **1A**–**4A** and **1B**–**4B**. Processes were	Figure S31

recorded in dichloromethane with 0.1M Bu_4NPF_6 as the supporting electrolyte under argon atmosphere at scan rate 100 mV/s	
20. Electrochemical properties of **1A–4A** and **1B–4B**	Table S9
21. UV-Vis absorption spectra of **1A–4A** and **1B–4B** in $CHCl_3$ and CH_3CN, compared to UV-Vis spectra of $ReCl(CO)_3$(terpy-κ^2N)], [$ReCl(CO)_3$(4′-Ph-terpy-κ^2N)] and [$ReCl(CO)_3$(4-Ph-dppy-κ^2N)]	Figure S32
22. The absorption maxima and molar extinction coefficient for **1A–4A** and **1B–4B** in two solvents of different polarity ($CHCl_3$ and CH_3CN) and in thin film on glass substrate	Table S10
23. UV-Vis absorption spectra of **1A–4A** and **1B–4B** in comparison to the electronic spectra of the free ligands	Figure S33
24. The impact of the triimine core and solvent polarity on the absorption properties of the investigated Re(I) complexes	Figure S34
25. UV-Vis absorption spectra of **1A–4A** and **1B–4B** in film together with photoluminescence (PL) spectrum of PVK:PBD	Figure S35
26. Electronic spectra of **1A–4A** to **1B–4B** in acetonitrile alongside with the theoretical results at computed at TD-DFT/PCM/PBE0/def2-TZVPD/def2-TZVP level with the use of the PCM model at polarities corresponding to CH_3CN	Figure S36
27. The energies and characters of the spin-allowed electronic transitions assigned to the lowest wavelength absorption bands of **1A–4A** to **1B–4B** computed at TD-DFT/PBE0/def2-TZVPD/def2-TZVP level with the use of the PCM model at polarities corresponding to CH_3CN	Table S11
28. Emission spectral data of **1A–4A** and **1B–4B** in two solvents of different polarity ($CHCl_3$, ε = 4.8 and CH_3CN, ε = 37.5), rigid matrix at 77 K (BuCN) and solid state as a powder	Figure S37
29. Normalized emission spectra of **1A–4A** in $CHCl_3$, along with the emission spectra of [$ReCl(CO)_3$(terpy-κ^2N)] and [$ReCl(CO)_3$(4′-Ph-terpy-κ^2N)]	Figure S38
30. Normalized emission spectra of **1A–4A** in CH_3CN, along with the emission spectra of [$ReCl(CO)_3$(terpy-κ^2N)] and [$ReCl(CO)_3$(4′-Ph-terpy-κ^2N)]	Figure S39
31. Normalized emission spectra of **1A–4A** in rigid matrix at 77 K, along with the emission spectra of [$ReCl(CO)_3$(terpy-κ^2N)] and [$ReCl(CO)_3$(4-Ph-terpy-κ^2N)]	Figure S40
32. Normalized emission spectra of **1A–4A** in the solid state, along with the emission spectra of [$ReCl(CO)_3$(terpy-κ^2N)] and [$ReCl(CO)_3$(4′-Ph-terpy-κ^2N)]	Figure S41
33. Normalized phosphorescence spectra of **1B–4B** in CH_3CN, along with the emission spectrum of [$ReCl(CO)_3$(4-Ph-dppy-κ^2N)]	Figure S42
34. Normalized phosphorescence spectra of **1B–4B** in $CHCl_3$, along with emission spectrum of [$ReCl(CO)_3$(4-Ph-dppy-κ^2N)]	Figure S43
35. Normalized emission spectra of **1B–4B** in the rigid matrix at 77 K, along with the emission spectrum of [$ReCl(CO)_3$(4-Ph-dppy-κ^2N)]	Figure S44
36. Normalized emission spectra of **1B–3B** in the solid state, along with the emission spectrum of [$ReCl(CO)_3$(4-Ph-dppy-κ^2N)]	Figure S45
37. Normalized phosphorescence spectra of **1A–4A** and **1B–4B** in $CHCl_3$	Figure S46
38. Normalized phosphorescence emission spectra of **1A–4A** and **1B–4B**	Figure S47

at 77 K	
39. Normalized emission spectra of **1A–3A** and **1B–3B** in the solid state	Figure S48
40. Normalized emission spectra of **1A–3A** in $CHCl_3$, solid state and rigid matrix at 77 K	Figure S49
41. Phosphorescence spectra of **4B** at 77 K along with the phosphorescence spectra of the free ligands and pyrene	Figure S50
42. Phosphorescence spectra of **4A** and [ReCl(CO)$_3$(terpy-κ^{2}N)] at 77K along with the phosphorescence spectra of the free ligands and pyrene	Figure S51
43. Excitation and emission spectra of **4A** in CH_3CN	Figure S52
44. Excitation and emission spectra of **4A** in $CHCl_3$	Figure S53
45. Excitation and emission spectra of **4B** in CH_3CN	Figure S54
46. Excitation and emission spectra of **4B** in $CHCl_3$	Figure S55
47. Comparison of fluorescence band of L^{4A} and 4A in $CHCl_3$ estimated at excitation wavelength 350nm. EnT estimated as 99%	Figure S56
48. Comparison of fluorescence band of L^{4A} and **4A** in CH_3CN estimated at excitation wavelength 350nm. EnT estimated as 87%	Figure S57
49. Emission spectra of deaerated and aerated solutions of **4A** and **4B** in CH_3CN and $CHCl_3$ solutions, demonstrating that the emission peak at longer wavelength arises from a triplet state	Figure S58
50. The energies of theoretical phosphorescence emissions, calculated from the difference between the ground singlet and the triplet state $\Delta E_{T_1-S_0}$, along with the experimental values and the spin density surface plots for **1A–3A**	Table S12
51. The energies of theoretical phosphorescence emissions, calculated from the difference between the ground singlet and the triplet state $\Delta E_{T_1-S_0}$, along with the experimental values and the spin density surface plots for **4A** and **4B**	Table S13
52. UV-Vis spectra **4A** and **4B** in CH_3CN and $CHCl_3$ recorded once every two hours over 24h at room temperature	Figure S59
53. UV-Vis spectra **4A** in CH_3CN and $CHCl_3$ after exposure of Xe arc lamp radiation	Figure S60
54. Comparison of TRES spectra of **1A** and **4A** in butyronitrile rigid matrix at 77K (time window 40ms, excitation wavelength 395 nm)	Figure S61
55. Comparison of TRES map of **4A** at room temperature (RT) in CH_3CN (time window 100ns, excitation wavelength 405 nm) and in $CHCl_3$ (time window 50μs, excitation wavelength 475 nm) and low temperature (LT)	Figure S62
56. Comparison of TRES slices of **4A**, **1A** and steady-state emission spectra of pairs 4A-L^{4A} and 1A-L^{1A} at 77K	Figure S63
57. Comparison of TRES map of **4B** at room temperature (RT, time window 2μs, excitation wavelength 405 nm)in CH_3CN and CHCl3 and low temperature (LT, time window 40μs, excitation wavelength 420nm).	Figure S64
58. Photoluminescence of Re(I) complexes in thin films and blends with of 15wt% complexes dispersed molecularly in PVK:PBD.	Table S14
59. PL spectra of 2A in film and blend (15wt.%).	Figure S65
60. UV-Vis absorption spectra of 1A–4A and 1B–4B in film together	Figure S66

with photoluminescence (PL) spectrum of PVK:PBD.	
61. (a) Electroluminescence spectra of selected diodes and (b) diagram with maximal reached EL intensity with λ_{EL} position.	Figure S67
62. The PL spectra of **1A** and **4A** dispersed molecularly in PVK:PBD matrix.	Figure S68
63. Summary of the global lifetime analysis of **1A** (pump 420 nm) containing 3D fsTA map, residual map, time traces at several wavelength, evolution associated spectra, decay associated spectra	Figure S69
64. Summary of the global lifetime analysis of **4A** (pump 355 nm) containing 3D fsTA map, residual map, time traces at several wavelength, evolution associated spectra, decay associated spectra	Figure S70
65. Summary of the global lifetime analysis of **4A** (pump 420 nm) containing 3D fsTA map, residual map, time traces at several wavelength, evolution associated spectra, decay associated spectra	Figure S71
66. Fits of decay curves of triplet-triplet excited state absorption maxima of **4A** (λ_{ESA} = 510 nm) (A) and $\mathbf{L^{4A}}$ (λ_{ESA} = 420 nm) (B)	Figure S72

Experimental details

General procedure for the synthesis of rhenium(I) carbonyl complexes

The corresponding 4′-Ar^n-terpy or 4-Ar^n-dppy ligand (0.27 mmol) was added to a suspension of [Re(CO)$_5$Cl] (0.27 mmol) and toluene (35 mL). The reaction mixture was heated at reflux during 8h under argon atmosphere. The formed precipitate was filtered off and washed with diethyl ether, dried in the air, and then purified by repeated recrystallization from toluene.

General characterization of Re(I) carbonyl complexes

[ReCl(CO)$_3$(4′-Ar^1-terpy-κ^{2}N)] (1A): Yield: 90%. ^{1}H NMR (400 MHz, DMSO-d$_6$) δ 9.10 (d, J = 5.1 Hz, 1H, **H**^{C1}), 9.04 (s, 1H, **H**^{B4}), 8.96 (d, J = 8.2 Hz, 1H, **H**^{C4}), 8.79 (d, J = 4.3 Hz, 1H, **H**^{A1}), 8.33 (t, J = 7.8 Hz, 1H, **H**^{C3}), 8.15 (d, J = 8.2 Hz, 1H, **H**^{D9}), 8.11 (d, J = 8.6 Hz, 1H, **H**^{D6}), 8.05 (t, J = 7.7 Hz, 1H, **H**^{A3}), 8.00 (s, 1H, **H**^{B2}), 7.98 – 7.90 (m, 2H, **H**$^{A4+D4}$), 7.78 (t, J = 6.5 Hz, 2H, **H**$^{C2+D8}$), 7.71 (t, J = 7.6 Hz, 1H, **H**^{D2}), 7.67 – 7.59 (m, 3H, **H**$^{A2+D7+D3}$). ^{13}C NMR (100 MHz, DMSO-d$_6$) δ 197.72 (**C**^{CO}), 194.49 (**C**^{CO}), 190.98 (**C**^{CO}), 161.00 (**C**^{B1}), 157.61 (**C**^{A5}), 156.79 (**C**^{B5}), 156.23 (**C**^{C5}), 152.75 (**C**^{C1}), 151.74 (**C**^{B3}), 149.26 (**C**^{A1}), 140.02 (**C**^{C3}), 136.90 (**C**^{A3}), 134.74 (**C**^{D5}), 133.35 (**C**^{D10}), 129.99 (**C**^{D9}), 128.87 (**C**^{D6}), 128.18 (**C**^{B2}), 127.95 (**C**^{D8}), 127.54 (**C**^{C2}), 126.57 (**C**^{D2}), 125.60 (**C**^{C4}), 125.47 (**C**^{D7}), 125.29 (**C**^{A2}), 125.25 (**C**^{D3}), 124.98 (**C**^{B4}), 124.60 (**C**^{D4}), 124.37 (**C**^{A4}). IR (KBr, cm^{-1}): 2020 (vs), 1915 (vs) and 1892 (vs) v(C≡O); 1612 (m), v(C=N) and v(C=C). HRMS (ESI) (m/z): [M-Cl]$^+$ calcd for $[C_{28}H_{17}N_3O_3Re]^+$ 630.0827. Found 630.0825. Anal. Calcd for $C_{28}H_{17}ClN_3O_3Re$ · ¾ C_7H_8 (734.21 g/mol): C, 54.39; H, 3.16; N, 5.72. Found: C, 54.79; H, 2.93; N, 5.71. DSC: I heating scan: crystal→ crystal transition = 147, 169°C; T_m = 266°C, T_c = 272°C; T_m = 291°C; II heating scan: T_g = 175 °C, T_c = 280°C, T_m = 301 °C.

[ReCl(CO)$_3$(4′-Ar^2-terpy-κ^{2}N)] (2A): Yield: 87%. ^{1}H NMR (400 MHz, DMSO-d$_6$) δ 9.25 (s, 1H, **H**^{B4}), 9.17 (d, J = 8.3 Hz, 1H, **H**^{C4}), 9.09 (d, J = 5.0 Hz, 1H, **H**^{C1}), 8.87 (s, 1H, **H**^{D10}), 8.82 (d, J = 4.6 Hz, 1H, **H**^{A1}), 8.42 (t, J = 7.7 Hz, 1H, **H**^{C3}), 8.38 – 8.31 (m, 2H, **H**$^{B2+D3}$), 8.16 (d, J = 8.7 Hz, 1H, **H**^{D2}), 8.13 – 8.02 (m, 3H, **H**$^{A3+D5+D8}$), 7.94 (d, J = 7.8 Hz, 1H, **H**^{A4}), 7.83 – 7.76 (m, 1H, **H**^{C2}), 7.68 – 7.61 (m, 3H, **H**$^{A2+D6+D7}$). ^{13}C NMR (100 MHz, DMSO-d$_6$) δ 197.81 (**C**^{CO}), 194.46 (**C**^{CO}), 190.99 (**C**^{CO}), 161.48 (**C**^{B1}), 157.90 (**C**^{A5}), 157.19 (**C**^{C5}), 156.30 (**C**^{B5}), 152.73 (**C**^{C1}), 150.50 (**C**^{D1}), 149.27 (**C**^{A1}), 139.95 (**C**^{C3}), 136.95 (**C**^{A3}), 133.84 (**C**^{D9}), 133.00 (**C**^{D4}), 131.97 (**C**^{B3}), 128.89 (**C**^{D2}), 128.20 (**C**^{D10}), 127.85 (**C**^{D8}), 127.69 (**C**^{D5}), 127.49 (**C**^{C2}), 127.02 (**C**^{A2}), 125.52 (**C**^{A4}), 125.31 (**C**^{C4}), 125.21 (**C**^{D7}), 124.97 (**C**^{D6}), 124.56 (**C**^{B2}), 124.48 (**C**^{D3}), 120.76 (**C**^{B4}). IR (KBr, cm^{-1}): 2019 (vs), 1914 (vs) and 1876 (vs) v(C≡O); 1613 (m), v(C=N) and v(C=C). HRMS (ESI) (m/z): [M-Cl]$^+$ calcd for $[C_{28}H_{17}N_3O_3Re]^+$ 630.0827. Found 630.0826. Anal. Calcd for $C_{28}H_{17}ClN_3O_3Re$ · ⅛ C_7H_8 (676.62 g/mol): C, 51.26; H, 2.68; N, 6.21. Found: C, 50. 89; H, 2.57; N, 6.32. DSC: I heating scan: crystal→ crystal transition = 209°C; T_m = 288, 298°C; II heating scan: T_g = 196 °C.

[ReCl(CO)$_3$(4′-Ar^3-terpy-κ^{2}N)] (3A): Yield: 63%. ^{1}H NMR (400 MHz, DMSO-d$_6$) δ 9.12 – 9.09 (m, 2H), 9.00 (d, J = 8.3 Hz, 1H), 8.94 (t, J = 8.5 Hz, 2H), 8.80 (d, J = 4.5 Hz, 1H), 8.33 (t, J = 7.6 Hz, 1H), 8.14 (s, 1H), 8.12 (d, J = 7.4 Hz, 1H), 8.08 – 8.03 (m, 2H), 7.99 – 7.92 (m, 2H), 7.83 –

7.68 (m, 6H), 7.62 (dd, J = 6.8, 5.4 Hz, 1H). ^{13}C NMR (100 MHz, DMSO-d$_6$) δ 197.73, 194.50, 191.00, 161.09, 157.63, 156.84, 156.24, 155.29, 154.87, 152.78, 151.85, 149.41, 149.28, 140.04, 136.91, 133.52, 130.57, 130.19, 130.14, 129.24, 129.02, 128.77, 128.13, 127.64, 127.48, 127.43, 125.48, 125.28, 125.00, 124.63, 123.70, 122.97. IR (KBr, cm^{-1}): 2021 (vs), 1911 (vs) and 1876 (vs) ν(C≡O); 1609 (m), ν(C=N) and ν(C=C). HRMS (ESI) (m/z): [M-Cl]$^+$ calcd for $[C_{32}H_{19}N_3O_3Re]^+$ 680.0984. Found 680.0981. Anal. Calcd for $C_{32}H_{19}ClN_3O_3Re$ (715.17 g/mol): C, 53.74; H, 2.68; N, 5.88. Found: C, 54.10; H, 2.60; N, 6.06. DSC: I heating scan: T_m = 297°C; II heating scan: T_g = 238 °C.

[ReCl(CO)$_3$(4′-Ar^4-terpy-κ^{2}N)] (4A): Yield: 79%. ^{1}H NMR (400 MHz, DMSO-d$_6$) δ 9.19 (s, 1H), 9.13 (d, J = 5.2 Hz, 1H), 9.00 (d, J = 8.3 Hz, 1H), 8.83 (d, J = 4.5 Hz, 1H), 8.51 (d, J = 8.0 Hz, 1H), 8.44 – 8.30 (m, 7H), 8.25 (d, J = 9.3 Hz, 1H), 8.20 – 8.14 (m, 2H), 8.08 (t, J = 8.3 Hz, 1H), 8.02 (d, J = 7.7 Hz, 1H), 7.83 – 7.78 (m, 1H), 7.67 – 7.62 (m, 1H). ^{13}C NMR (100 MHz, DMSO-d$_6$) δ 198.22, 194.99, 191.50, 161.50, 158.19, 157.30, 156.77, 153.27, 152.55, 149.78, 140.53, 137.43, 132.36, 132.24, 131.33, 130.78, 129.61, 129.37, 129.15, 129.09, 128.67, 128.25, 128.20, 128.01, 127.78, 127.29, 126.65, 126.23, 126.00, 125.81, 125.59, 125.54, 125.50, 124.49, 124.23, 124.01. IR (KBr, cm^{-1}): 2020 (vs), 1913 (vs) and 1880 (vs) ν(C≡O); 1610 (m), ν(C=N) and ν(C=C). HRMS (ESI) (m/z): [M-Cl]$^+$ calcd for $[C_{34}H_{19}N_3O_3Re]^+$ 704.0984. Found 704.0984. Anal. Calcd for $C_{34}H_{19}ClN_3O_3Re$ (739.19 g/mol): C, 55.24; H, 2.59; N, 5.68. Found: C, 55.33; H, 2.49; N, 5.72. DSC: I heating scan: T_m = 322, 349°C; II heating scan: T_g = 257 °C.

[ReCl(CO)$_3$(4-Ar^1-dppy-κ^{2}N)] (1B): Yield: 78%. ^{1}H NMR (400 MHz, DMSO-d$_6$) δ 10.22 (s, 1H), 9.31 (d, J = 1.3 Hz, 1H), 9.21 (s, 1H), 9.17 (d, J = 2.3 Hz, 1H), 8.98 (d, J = 3.1 Hz, 1H), 8.92 (s, 2H), 8.24 (d, J = 1.3 Hz, 1H), 8.17 (d, J = 8.2 Hz, 1H), 8.14 – 8.09 (m, 1H), 8.02 – 7.97 (m, 1H), 7.83 (d, J = 6.4 Hz, 1H), 7.78 – 7.69 (m, 1H), 7.68 – 7.61 (m, 2H). ^{13}C NMR (100 MHz, DMSO-d$_6$) δ 196.46, 194.56, 189.52, 158.37, 155.15, 153.32, 152.08, 150.96, 147.93, 147.06, 145.89, 145.83, 145.46, 144.07, 134.33, 133.34, 130.25, 129.78, 128.86, 128.69, 128.16, 127.64, 126.64, 125.59, 125.27, 124.44. IR (KBr, cm^{-1}): 2024 (vs), 1932 (vs) and 1914 (vs) ν(C≡O); 1609 (m), ν(C=N) and ν(C=C). HRMS (ESI) (m/z): [M-Cl]$^+$ calcd for $[C_{26}H_{15}N_5O_3Re]^+$ 632.0732. Found 632.0735. Anal. Calcd for $C_{26}H_{15}ClN_5O_3Re$ · ½ C_7H_8 (713.16 g/mol): C, 49.68; H, 2.69; N, 9.82. Found: C, 49.51; H, 3.02; N, 9.91. DSC: I heating scan: T_m = 242°C; II heating scan: T_g = 175 °C.

[ReCl(CO)$_3$(4-Ar^2-dppy-κ^{2}N)] (2B): Yield: 74%. ^{1}H NMR (400 MHz, DMSO-d$_6$) δ 10.36 (s, 1H), 9.44 (s, 1H), 9.20 (s, 1H), 9.15 (s, 1H), 9.00 – 8.92 (m, 3H), 8.90 (s, 1H), 8.57 (s, 1H), 8.34 (d, J = 8.6 Hz, 1H), 8.14 (d, J = 8.6 Hz, 1H), 8.10 – 8.00 (m, 2H), 7.68 – 7.60 (m, 2H). ^{13}C NMR (100 MHz, DMSO-d$_6$) δ 196.54, 194.60, 189.59, 158.74, 155.51, 153.50, 150.92, 150.68, 147.90, 147.06, 145.86, 145.84, 145.33, 144.11, 133.93, 132.90, 131.42, 129.02, 128.88, 128.35, 128.00, 127.70, 127.09, 125.47, 124.45, 121.67. IR (KBr, cm^{-1}): 2023 (vs), 1920 (vs) and 1898 (vs) ν(C≡O); 1612 (m), ν(C=N) and ν(C=C). HRMS (ESI) (m/z): [M-Cl]$^+$ calcd for $[C_{26}H_{15}N_5O_3Re]^+$ 632.0732. Found 632.0734. Anal. Calcd for $C_{26}H_{15}ClN_5O_3Re$ · $^1/_4$ C_7H_8 (690.12 g/mol): C, 48.30; H, 2.48; N, 10.15. Found: C, 47.98; H, 2.66; N, 9.91. DSC: I heating scan: T_m = 305°C with decomposition.

[ReCl(CO)$_3$(4-Ar^3-dppy-κ^{2}N)] (3B): Yield: 61%. ^{1}H NMR (400 MHz, DMSO-d$_6$) δ 10.23 (s, 1H), 9.39 (s, 1H), 9.22 (s, 1H), 9.18 (d, *J* = 2.7 Hz, 1H), 9.03 (d, *J* = 8.4 Hz, 1H), 9.01 – 8.94 (m, 2H), 8.92 (s, 2H), 8.32 (s, 1H), 8.21 (s, 1H), 8.14 (d, *J* = 7.7 Hz, 1H), 8.01 (d, *J* = 8.5 Hz, 1H), 7.83 (t, *J* = 7.3 Hz, 2H), 7.78 – 7.71 (m, 2H). ^{13}C NMR (100 MHz, DMSO-d$_6$) δ 196.47, 194.58, 189.56, 158.47, 155.21, 153.35, 152.22, 150.99, 147.98, 147.06, 145.95, 145.86, 145.49, 144.12, 133.16, 130.52, 130.23, 130.19, 129.38, 129.29, 128.64, 128.29, 127.72, 127.56, 127.52, 125.72, 125.59, 123.72, 123.02. IR (KBr, cm^{-1}): 2023 (vs), 1912 (vs) and 1892 (vs) *ν*(C≡O); 1612 (m), *ν*(C=N) and *ν*(C=C). HRMS (ESI) (m/z): [M-Cl]$^+$ calcd for $[C_{30}H_{17}N_5O_3Re]^+$ 682.0889. Found 682.0891. Anal. Calcd for $C_{30}H_{17}ClN_5O_3Re$ (717.14 g/mol): C, 50.24; H, 2.39; N, 9.77. Found: C, 50.27; H, 2.13; N, 9.77. DSC: I heating scan: T_m = 255°C; II heating scan: T_g = 215 °C.

[ReCl(CO)$_3$(4-Ar^4-dppy-κ^{2}N)] (4B): Yield: 35%. ^{1}H NMR (400 MHz, DMSO-d6) δ 10.17 (s, 1H), 9.37 (s, 1H), 9.23 (s, 1H), 9.16 (d, *J* = 2.7 Hz, 1H), 8.97 (d, *J* = 2.9 Hz, 1H), 8.91 (s, 2H), 8.48 (d, *J* = 8.0 Hz, 1H), 8.40 – 8.29 (m, 8H), 8.14 (t, *J* = 7.7 Hz, 1H). ^{13}C NMR not recorded due to insufficient complex solubility. IR (KBr, cm^{-1}): 2021 (vs), 1932 (vs) and 1895 (vs) *ν*(C≡O); 1609 (m), *ν*(C=N) and *ν*(C=C). HRMS (ESI) (m/z): [M-Cl]$^+$ calcd for $[C_{32}H_{17}N_5O_3Re]^+$ 706.0889. Found 706.0886. Anal. Calcd for $C_{32}H_{17}ClN_5O_3Re$ · ⅞ C_7H_8 (821.78 g/mol): C, 55.72; H, 2.94; N, 8.52. Found: C, 55.72; H, 2.82; N, 8.85. DSC: I heating scan: crystal→ crystal transition=154°C; T_m = 298°C; II heating scan: T_g = 232 °C.

Film and device preparation

Films on glass substrates were obtained from a homogeneous $CHCl_3$ solution (c = 10 mg/mL) of Re(I) carbonyl complexes. They were prepared by spin-coating method at 1000 rpm during 60 s. Then films were dried for 24 h in a vacuum oven at 50 °C. The devices with configuration ITO/PEDOT:PSS/complex/Al and ITO/PEDOT:PSS/PVK:PBD:complex/Al were prepared. First OSSILA substrates with pixilated ITO (100 nm) anodes were covered with PEDOT: PSS (40 nm) by spin-coating technique at 5000 rpm for 60 s and annealed for 10 min at 120 °C. The active layers were spin-coated from chloroform solution at 1000 rpm for 60 s and annealed for15 min at 100 °C. The active layers with thickness about 80 nm for blend with 1 and 2 and 100 nm for blend with 15 wt.% of complex content were obtained. After annealing the Al (110 nm) was vacuum-deposited. The thickness of layers was determined by atomic force microscope (AFM) using TopoMetrix Explorer device, operating in contact mode, in air, in constant force regime.

HR-MS measurements

High resolution mass spectrometry analyses were performed on a Waters Xevo G2 Q-TOF mass spectrometer (Waters Corporation) equipped with an ESI source operating in positive- ion modes. Full-scan MS data were collected from 100 to 1000 Da in positive ion mode with scan time of 0.1 s. To ensure accurate mass measurements, data were collected in centroid mode and mass was corrected during acquisition using leucine enkephalin solution as an external reference (Lock-Spray ™), which generated reference ion at m/z 556.2771 Da ([M+H]$^+$) in positive ESI mode. The accurate mass and composition for the molecular ion adducts were calculated using the MassLynx software (Waters) incorporated with the instrument.

Device settings:

Polarity:	ES+
Analyser:	Resolution Mode
Capillary (kV):	4.0000
Sampling Cone:	40.0000
Extraction Cone:	4.0000
Source Temperature (°C):	120
Desolvation Temperature (°C):	200
Cone Gas Flow (L/Hr):	50.0
Desolvation Gas Flow (L/Hr):	500.0

Crystal structure determination and refinement

To collect X-ray diffraction data of **1A,** a Gemini A Ultra diffractometer with graphite monochromated MoKα radiation was used, and data collection, cell refinement and data reduction were carried out at room temperature with the use of the CrysAlis^{Pro} software [1]. SHELXS-2014 was utilized to solve the structure of **1A** by direct methods, and the refinement was carried out with SHELXL-2014 using full-matrix least-squares on F^2 [2]. The non-hydrogen atoms were refined anisotropically, and the riding model was used to place hydrogen atoms were placed in calculated positions refined using idealized geometries, with fixed isotropic displacement parameters d(C–H) = 0.93 Å. $U_{iso}(H) = 1.2\ U_{eq}$ (C). Toluene could not be modelled satisfactorily, and it was therefore removed from the electron density map using the OLEX2 solvent mask command [3].

Computational studies

The calculations were performed using GAUSSIAN-16 program package [4] at the DFT and TD-DFT level with PBE0 hybrid exchange-correlation functional [5,6], and the basis sets were def2-TZVPD for rhenium and def2-TZVP for other elements [7-9]. Both geometries of the singlet ground state (S_0) and the lowest triplet state (T_1) were fully optimized, and all the subsequent calculations were conducted on the optimized geometries. In all calculations, the acetonitrile solvent environment was simulated using the polarizable continuum model (PCM) correction [10-12]. Absorption properties were calculated by the TD-DFT method. The energies of phosphorescence emissions were calculated from the difference between the ground singlet and the triplet state $\Delta E_{T_1-S_0}$, and the character of the triplet lowest energy excited state was determined using the spin density surface plots.

Electrochemistry

Electrochemical measurements were performed on an Eco ChemieAutolab PGSTAT128n potentiostat in the three-electrode system: glassy carbon electrode (working electrode), platinum coil (auxiliary electrode) and silver (reference electrode) for dichloromethane (Aldrich. HPLC grade) solutions ($1.0 \cdot 10^{-3}$mol/dm^3) under argon atmosphere with electrolyte 0.1M Bu_4NPF_6

(Aldrich. 99%). and scan rate 0.1 V/s. The potentials were calibrated with respect to the internal the ferrocene/ferrocenium couple.

Steady-state and time-resolved emission spectroscopy

Steady-state photoluminescence emission spectra were measured with FLS-980 fluorescence spectrophotometer in solid state and solution at room temperature and in butyronitrile rigid matrix at 77 K. 450 W Xe lamp and photomultiplier (Hamamatsu, R928P) detector were used as light source and detector, respectively. Photoluminescence spectra in solid state as film deposited on a glass substrate and as blends with PVK:PBD on a glass substrate were collected on Hitachi F-2500 spectrometer. The photoluminescence lifetimes were measured using time correlated single photon counting (TCSPC) or multi-channel scaling (MCS) method, with additional measurement of the IRF for the analysis of a fluorescence decay. The quantum yields were determined using integrating sphere absolute method for argon bubbling solutions and solid states (powders). In each measurement, a solvent ($CHCl_3$ or CH_3CN) and Spectralon® were used as reference for diluted solutions and for powder samples, respectively. The emission correction file was applied to take into account the sensitivity of the monochromator, detector, sphere coating and optics to wavelength. Each scans were conducted with 0.25 nm step, 0.2 dwell time as well as repeated 3 times. The FLS-980 software was used to designate the quantum yield values, with error bar not exceeding ± 2% of quantum yield value.

Electroluminescence spectra measurements

A precise voltage supply (Gw Instek PSP-405) with the sample fixed to an XYZ stage was applied to collect electroluminescence (EL) spectra. Light from the OLED device was collected through a 30 mm lens, focused on the entrance slit (50 μm) of a monochromator (Shamrock SR-303i) and detected using a CCD detector (Andor iDus 12305). Typical acquisition times were equal to 10 seconds. The pre-alignment of the setup was done using a 405 nm laser.

Nanosecond transient absorption spectroscopy

A detailed setup for nanosecond transient absorption spectra was given previously [13]. Freshly prepared, diluted solutions of complex **4A** and corresponding ligand **4-Ar^4-terpy** were argon bubbled via 30 min and placed in rectangular 1 cm × 1 cm quartz cells. Samples for nsTA experiments were excited using 355 nm on laser flash photolysis setup described elsewhere[1]. The Nd:YAG laser (Spectra Physics Mountain View, CA, USA, model INDI 40-10) generated the third harmonic pulses of 6–8 ns duration. The monitoring system consisted of a 150 W pulsed Xe lamp with a lamp pulser (Applied Photophysics, Surrey, U.K.), a monochromator (Princeton Instruments, model Spectra Pro SP-2357, Acton, MA, USA), and a R955 model photomultiplier (Hamamatsu, Japan), powered by a PS-310 power supply (Stanford Research System, Sunnyvale, CA, USA). The data processing system consisted of real time acquisition using a digital oscilloscope (WaveRunner 6100A, LeCroy, Chestnut Ridge, NT, USA) which was triggered by a fast photodiode (Thorlabs, DET10M, ~1 ns rise time). The data from the oscilloscope were transferred to a computer equipped with software based on LabView 8.0 (National Instruments, Austin, TX, USA) which controls the

timing and acquisition functions of the system. Data acquired on the nanosecond laser setup were analyzed using Origin 8.0 fitting functions.

Photodamage test and fluence analysis of solution samples for fs studies

Prior femtosecond experiments both fluence dependence and photodamage tests were performed in order to determine the appropriate experiment conditions of for each sample. The fluence dependence and photodamage tests of samples are given below:

Table S1. Fluence dependence and photo-stability tests of **1A**, **4A** and **L^{4A}** prior femtosecond transient absorption experiment.

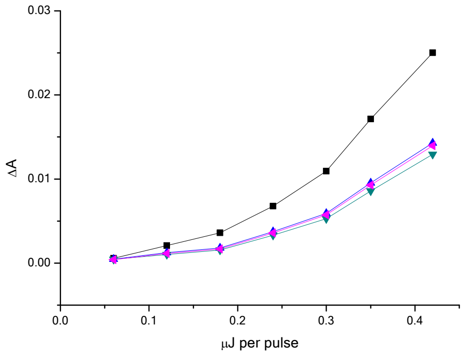

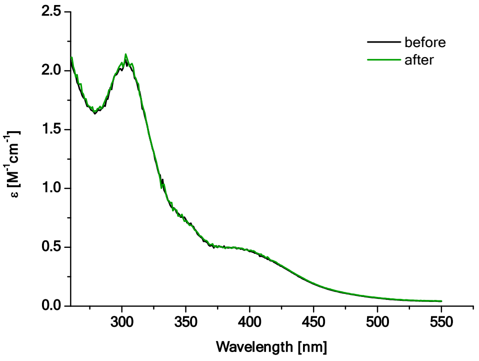

1A: Fluence test were performed at 20 ps time delay, in the range 0.06 to 0.42 μJ per pulse. **1A** showed a linear regime within range 0.06 – 0.18 μJ per pulse. The pump-probe measurement was performed at 0.18 μJ per pulse, approaching the highest fluence within the linear regime. The photodamage test under these conditions displays no loss of absorption after fsTA experiment.

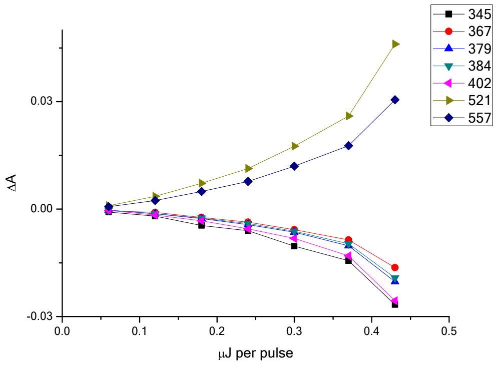

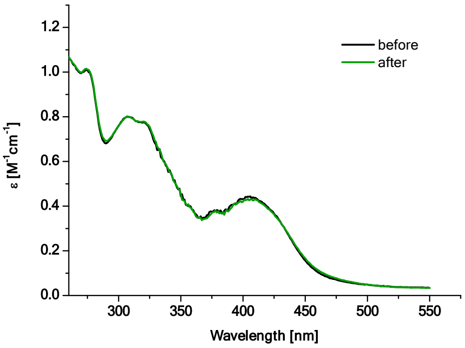

4A: Fluence test were performed at 20 ps time delay, in the range 0.06 to 0.43 μJ per pulse. **4A** showed a linear regime within range 0.06 – 0.24 μJ per pulse. The pump-probe measurement was performed at 0.21 μJ per pulse, approaching the highest fluence within the linear regime. The photodamage test under these conditions displays no loss of absorption after fsTA experiment.

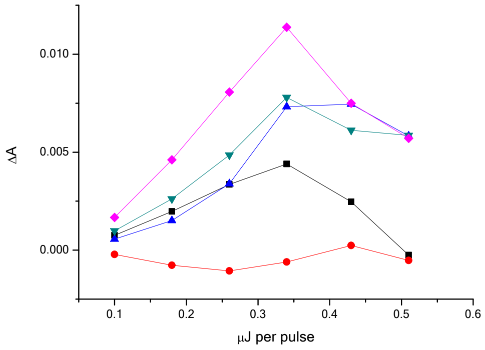

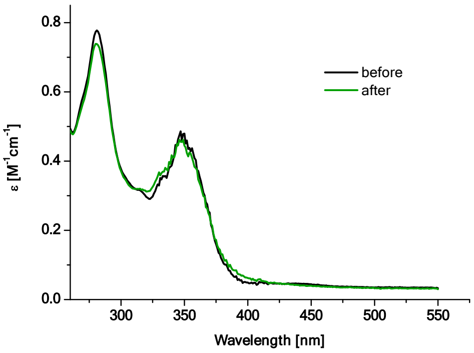

L^{4A}: Fluence test were performed at 20 ps time delay, in the range 0.1 to 0.51 μJ per pulse. L^{4A} showed a linear regime within range 0.10 – 0.26 μJ per pulse. The pump-probe measurement was performed at 0.18 μJ per pulse, approaching the highest fluence within the linear regime with relatively small photodamage of sample. The photodamage test under these conditions displays only minimal loss of absorption (~0.03) after fsTA experiment.

Femtosecond transient absorption spectroscopy

Femtosecond transient absorption spectra were measured using Helios pump-probe transient absorption spectrometer (Ultrafast Systems) with the system setting as in ref. [14]. The solutions of **1A** and **4A** in chloroform (concentrations between 100 – 500 μM) with the absorbance of 0.4–0.5 in the excitation wavelengths were placed in 2 mm path length quartz cells with magnetic stirring, and excited with 420 nm or 355 nm pump pulses. A regenerative amplified femtosecond Ti:sapphire laser system (Astrella, Coherent) delivered pulses under 100 fs duration with 5 mJ pulse with repetition rate of 1 kHz and a central wavelength of 800 nm. The excitation pulses of 420 nm and 355 nm were generated from an optical parametric amplifier (Light Conversion, TOPAS prime). A white light continuum pulse was generated by focusing the residual of the fundamental light at CaF_2 crystal, which was used as a probe beam. The pump pulse was chopped by a mechanical chopper synchronised to one-half of the laser repetition rate (1 kHz), resulting in a pair of spectra with and without the pump, from which absorption changes induced by the pump pulse were estimated. Pump beam was depolarised to mimic dynamical changes of the orientation of the molecules. The delay time between pump and probe pulses was controlled by a moveable delay line in a time scale up to 7.5 ns. For the detection of the transient absorption signals, the white light continuum after passing through the sample was sent to CCD detector installed in the system.

Transient absorption data were prepared using the Surface Xplorer (Ultrafast Systems) software and then analysed with use of Optimus^{TM} [15] software. Correction of background, scattered light subtraction, solvent signal contribution subtraction as well as removing of spikes were performed routinely prior to analysis. Moreover, coherent artefact analysis provided necessary information about IRF (IRF FWHM was estimated as about 144 fs and 181 fs, respectively for 420 nm and 355 nm pump pulse) and allowed to the corrections of the probe chirp. The ΔA vs λ spectra consist of positive and negative bands, in which the former are due to excited-state absorption (ESA), whereas the latter are ground state bleaching (GSB) or stimulated emission (SE) bands. The wavelength range (405 – 425 nm), consisting rest of scattering of pump light, was excluded from the global

analysis, which was performed using linear unidirectional sequential model implemented in Optimus™ [15] software. The analysis allows to the deconvolution of the transient spectra into species-associated spectra (SAS) and provides the decay-associated spectra (DAS) as a linear combination of the SAS of the compartments.

NMR spectra

Complex 1A

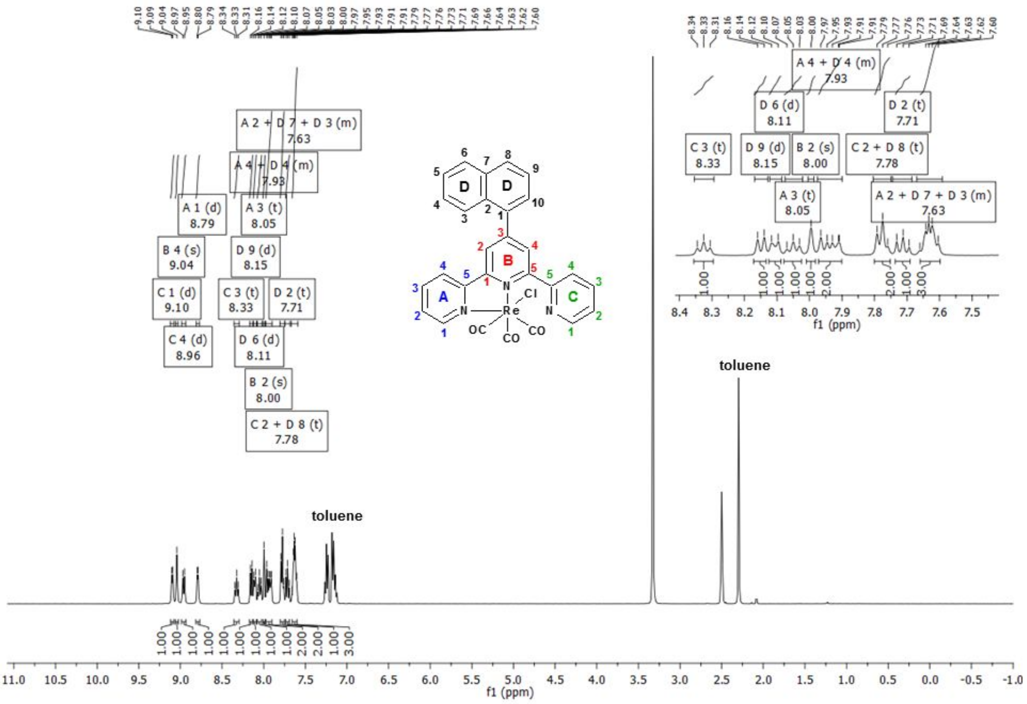

a)

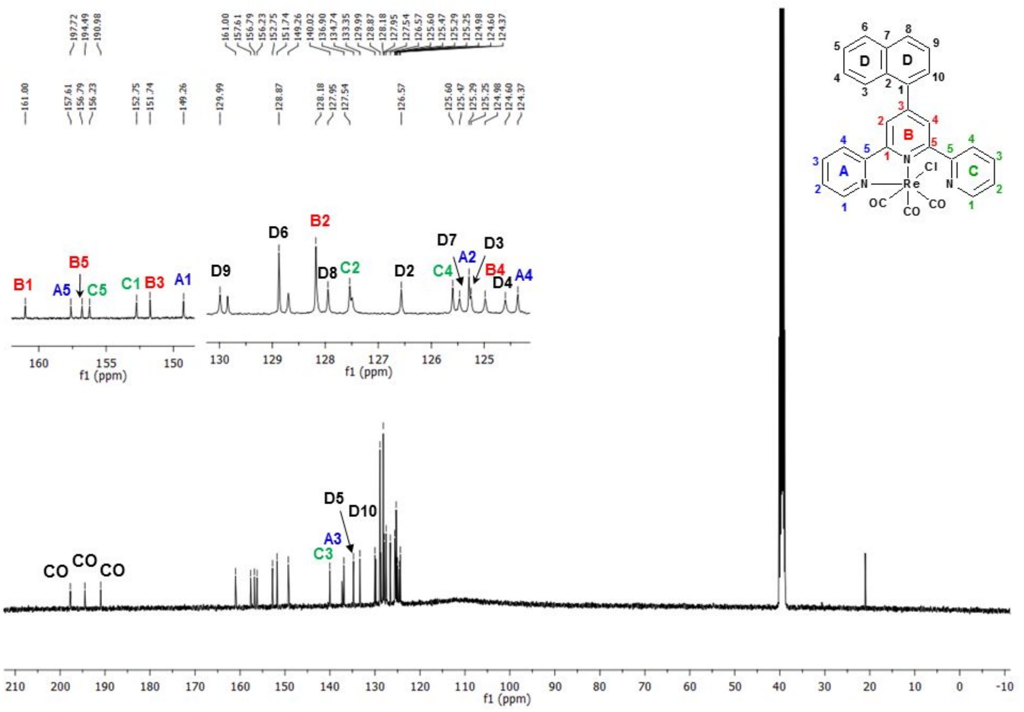

b)

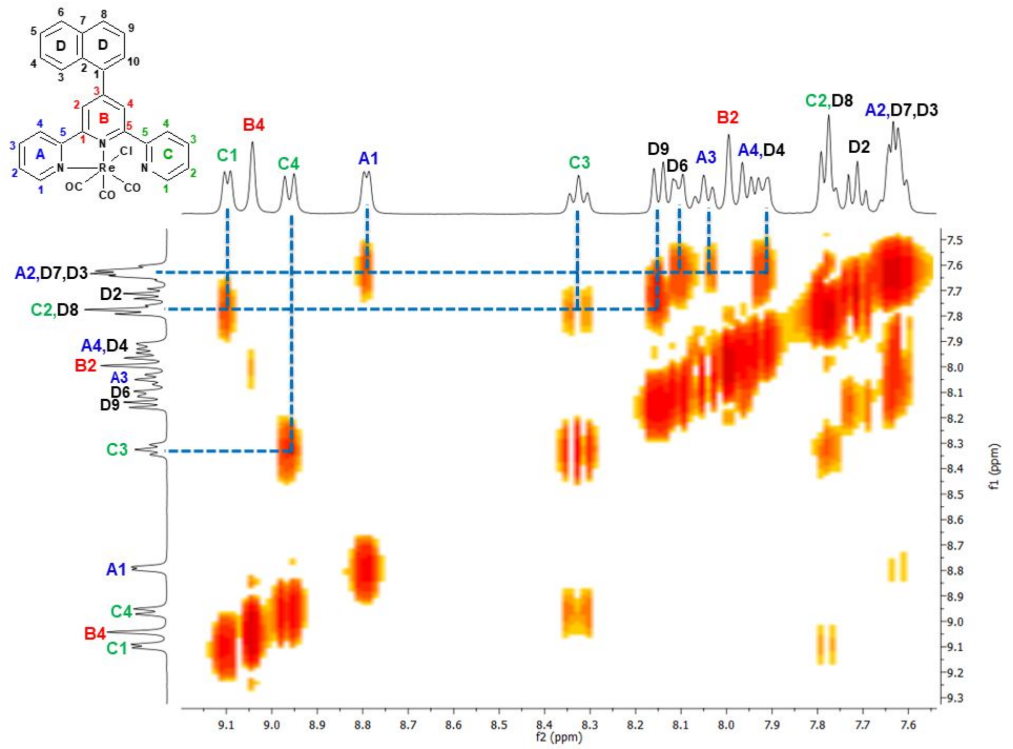

c)

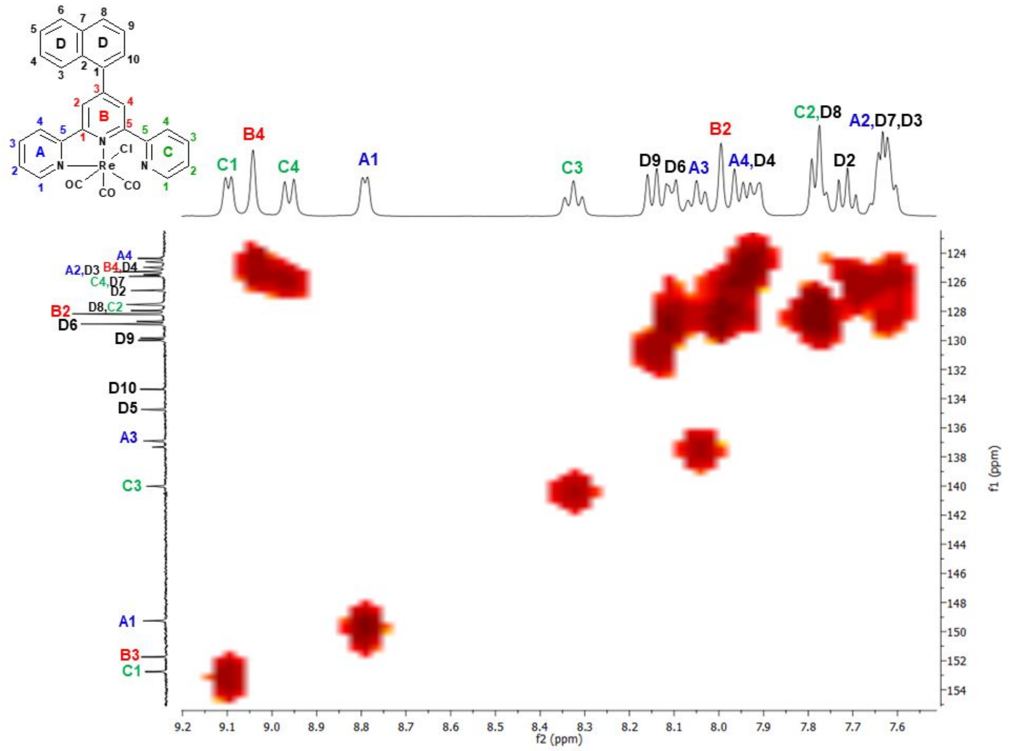

d)

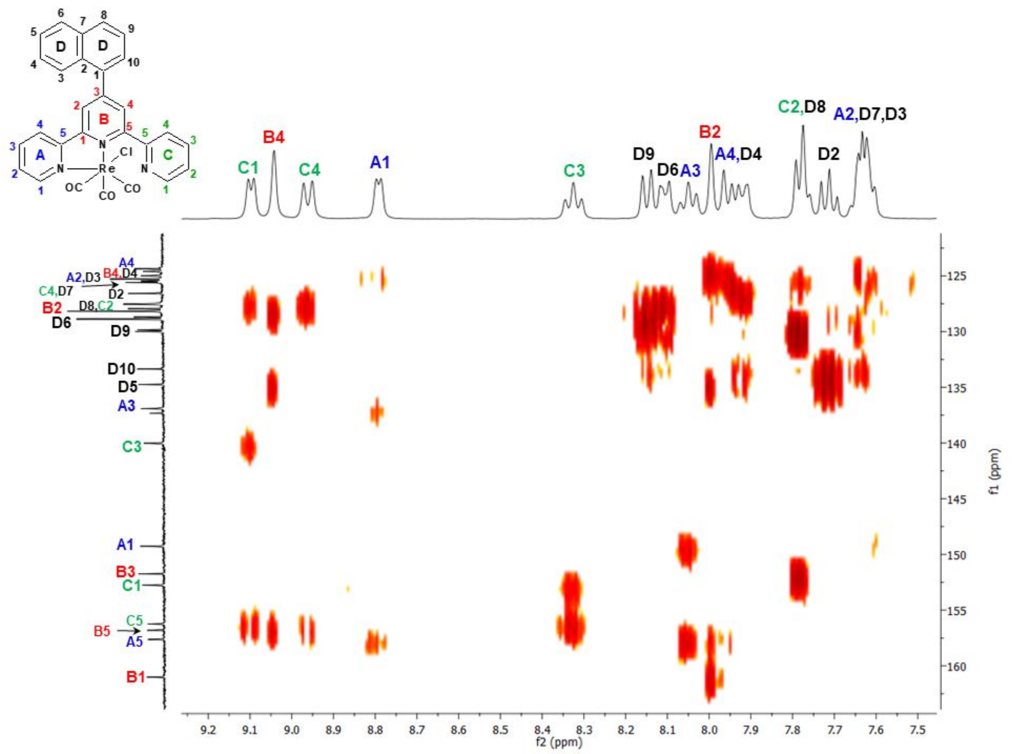

e)

Figure S1. NMR spectra of **1A** in DMSO-d_6: ^{1}H (a), ^{13}C (b), ^{1}H–^{1}H COSY (c), ^{1}H–^{13}C HMQC (d), ^{1}H–^{13}C HMBC (e).

Complex 2A

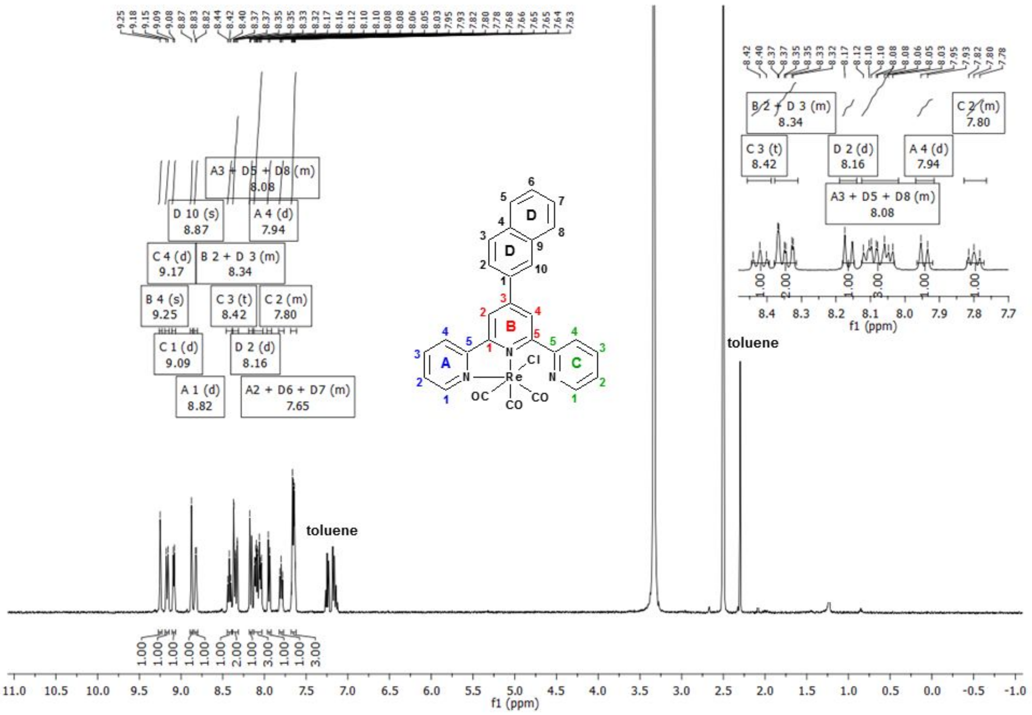

a)

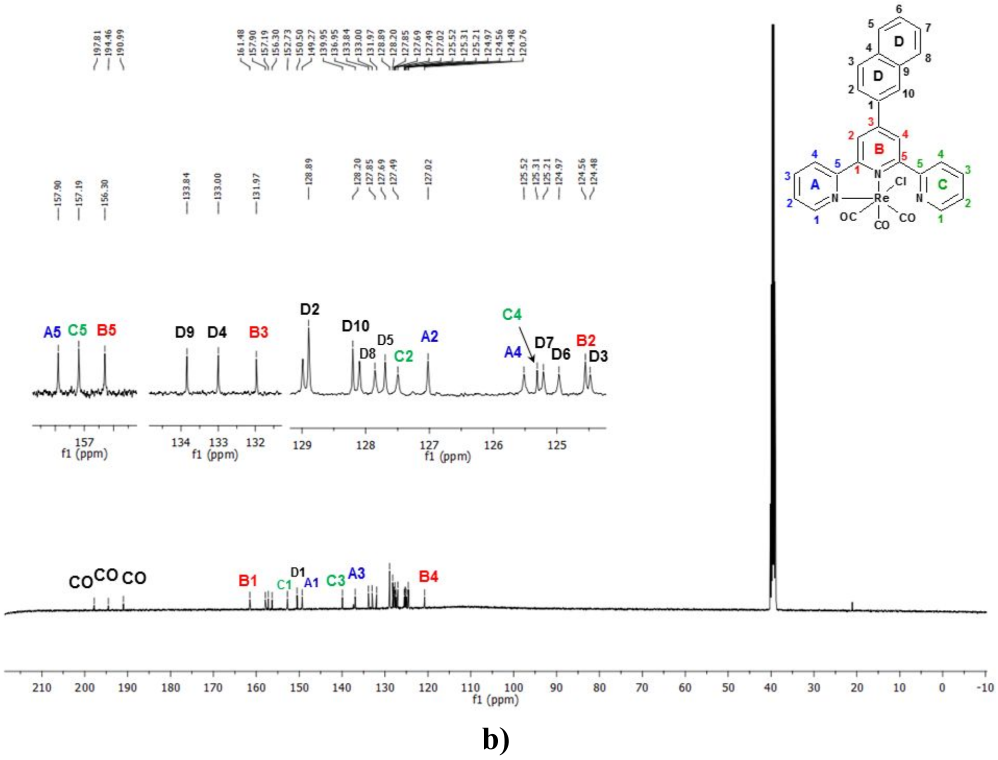

b)

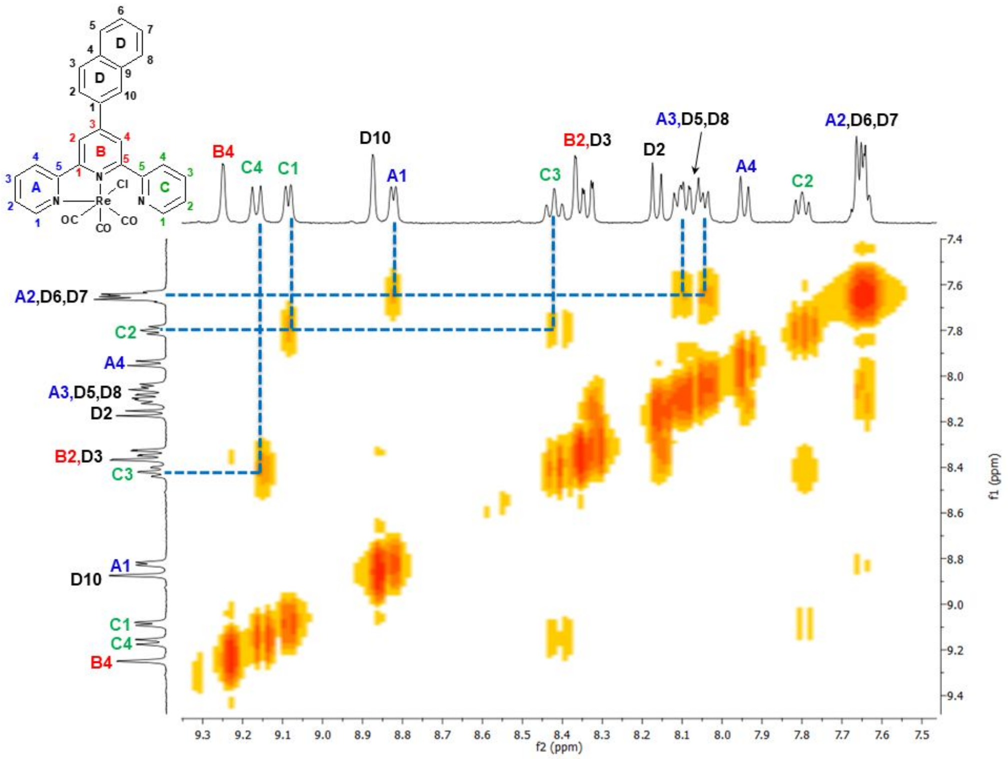

B4
C4
C1
D10
A1
C3
B2,D3
D2
A3,D5,D8
A4
C2
A2,D6,D7
f1 (ppm)
f2 (ppm)

c)

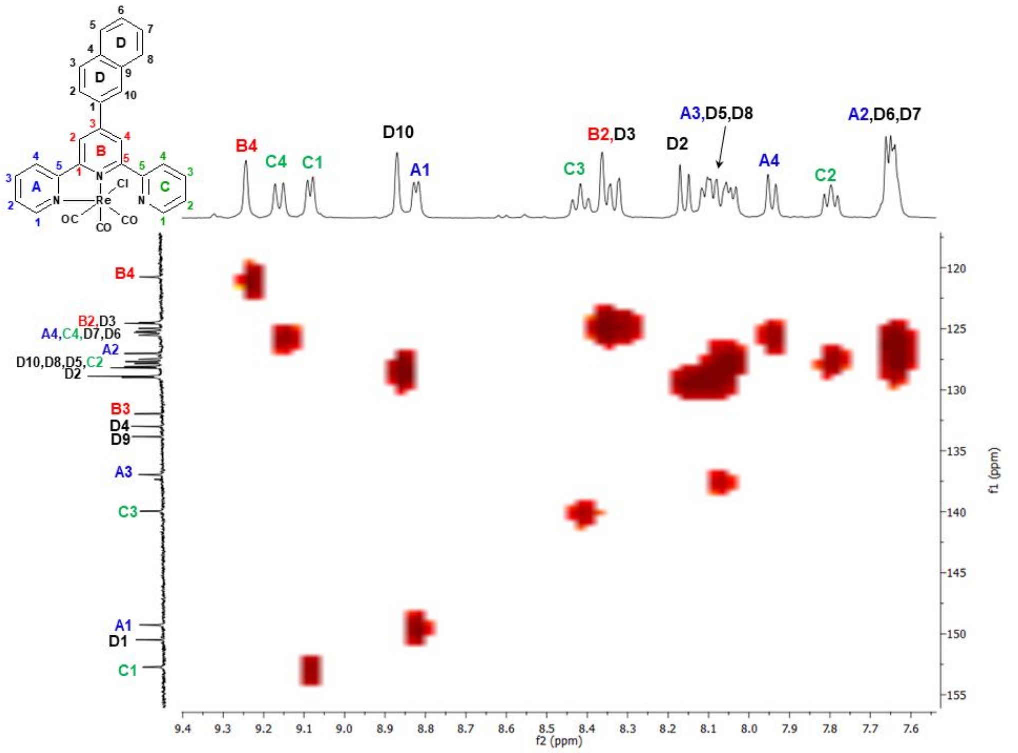

B4
C4
C1
D10
A1
C3
B2,D3
D2
A3,D5,D8
A4
C2
A2,D6,D7
A4,C4,D7,D6
A2
D10,D8,D5,C2
B3
D4
D9
A3
D1
f1 (ppm)
f2 (ppm)

d)

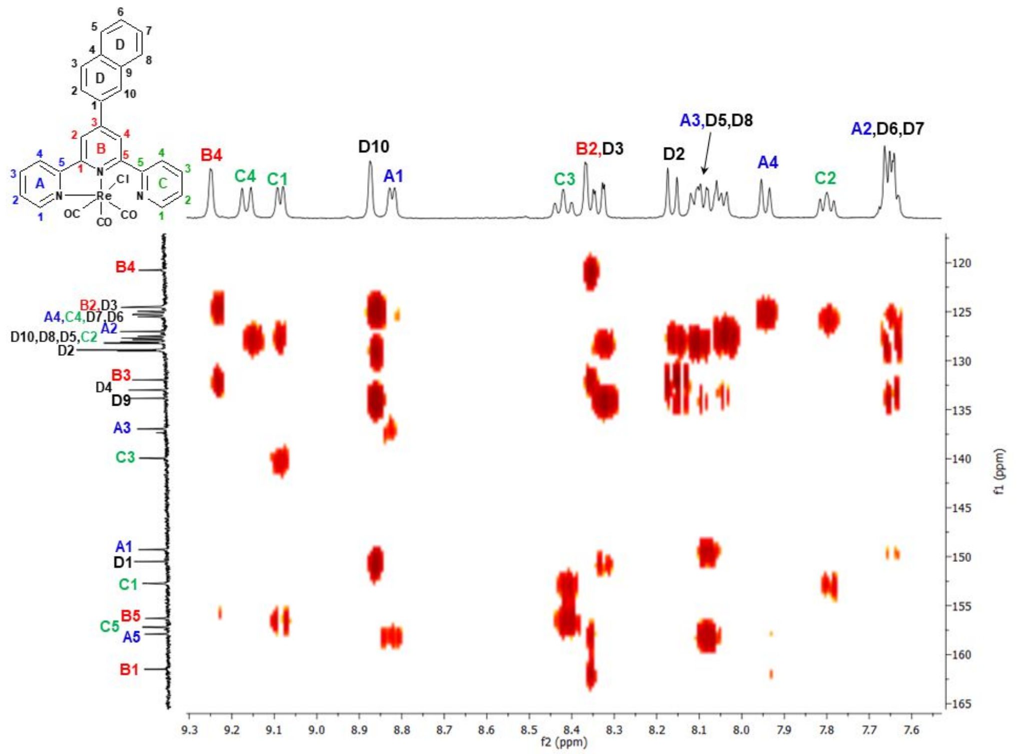

e)

Figure S2. NMR spectra of **2A** in DMSO-d$_6$: ^{1}H (a), ^{13}C (b), ^{1}H–^{1}H COSY (c), ^{1}H–^{13}C HMQC (d), ^{1}H–^{13}C HMBC (e).

Complex 3A

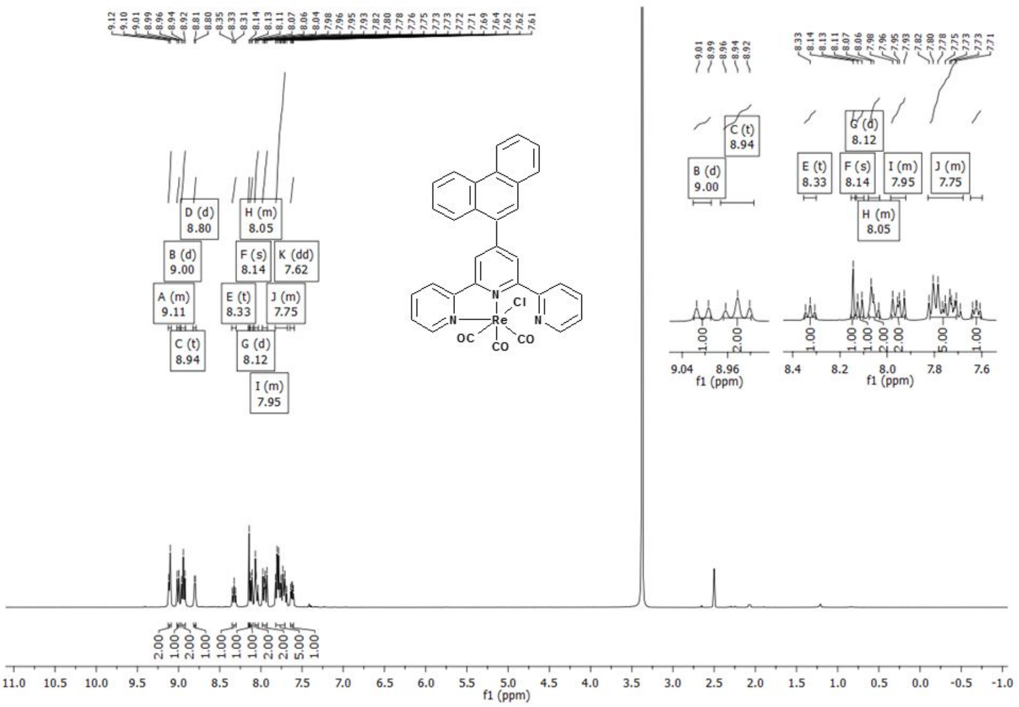

a)

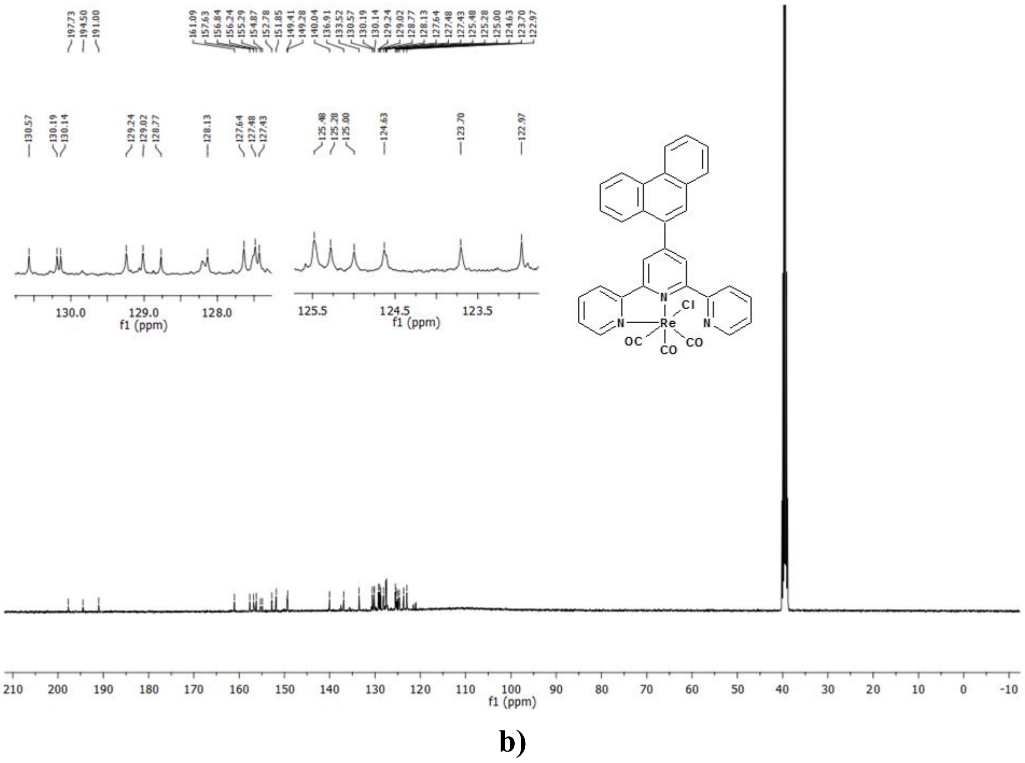

b)

Figure S3. NMR spectra of **3A** in DMSO-d$_6$: ^{1}H (a) and ^{13}C (b).

Complex 4A

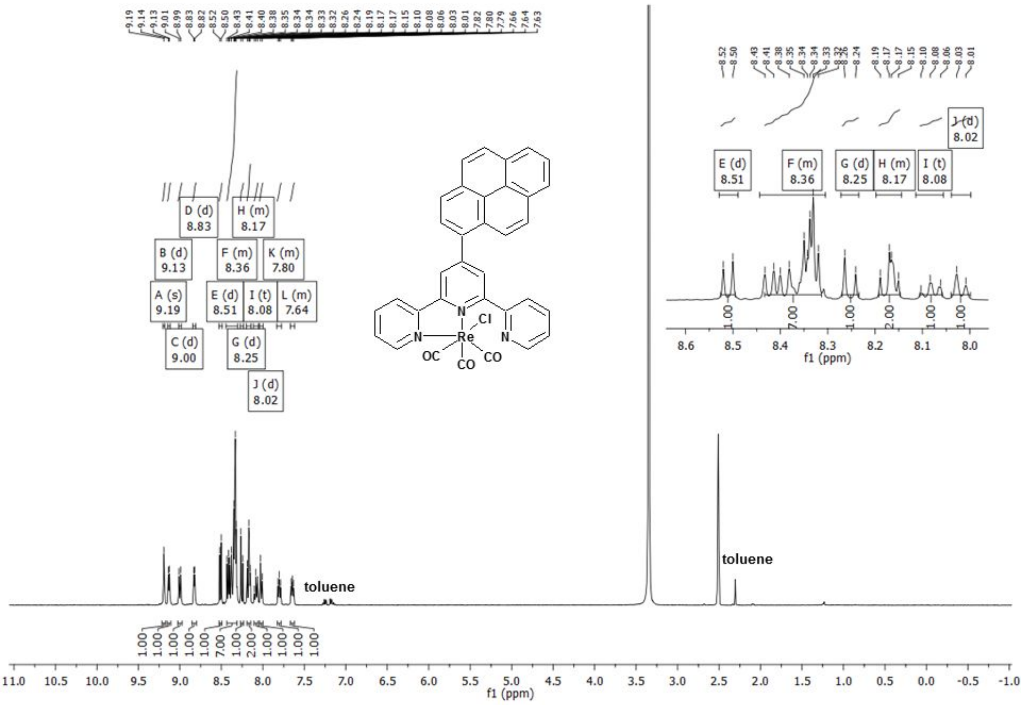

a)

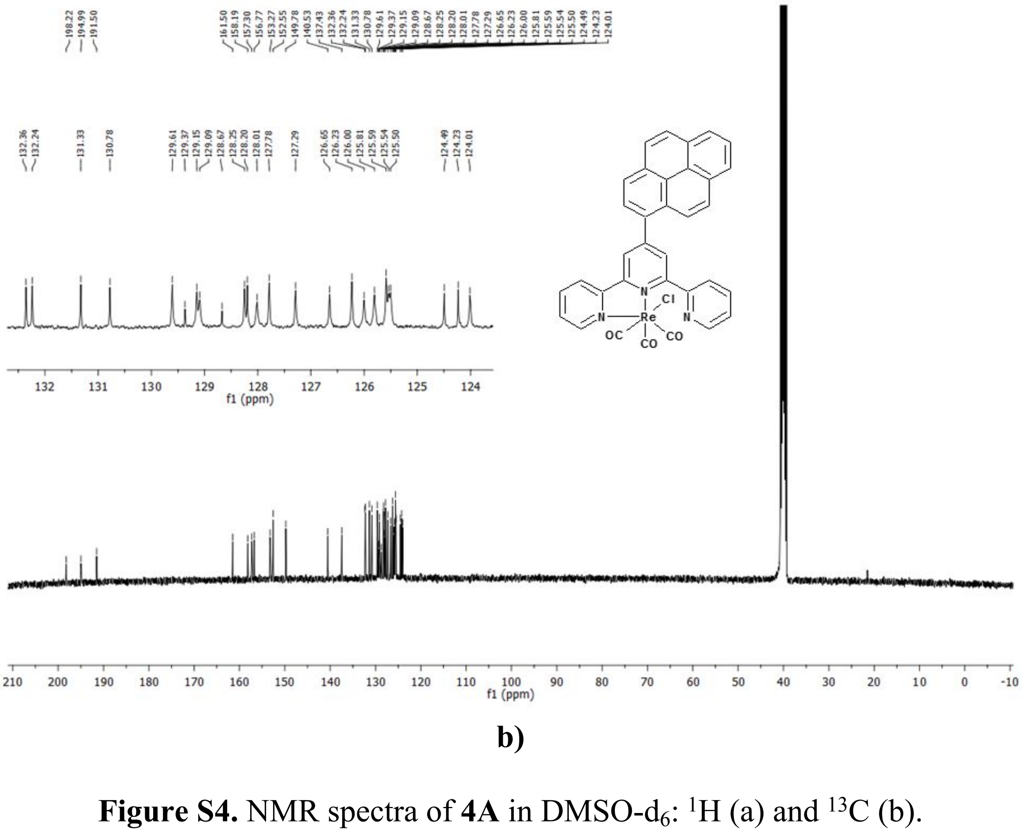

b)

Figure S4. NMR spectra of **4A** in DMSO-d_6: ^1H (a) and ^{13}C (b).

Complex 1B

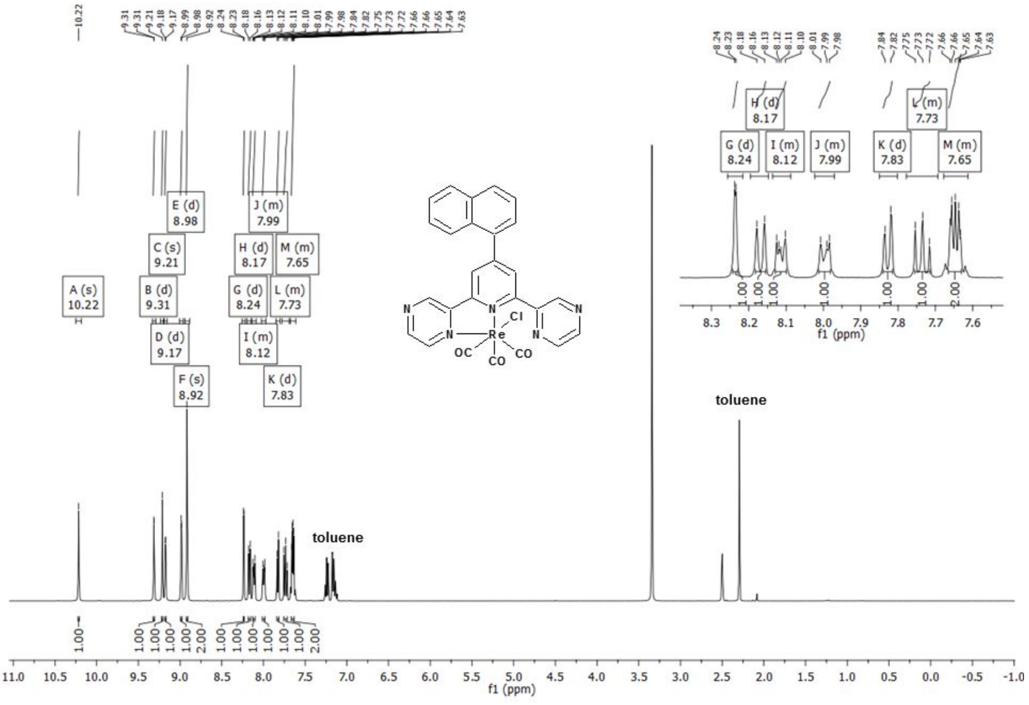

a)

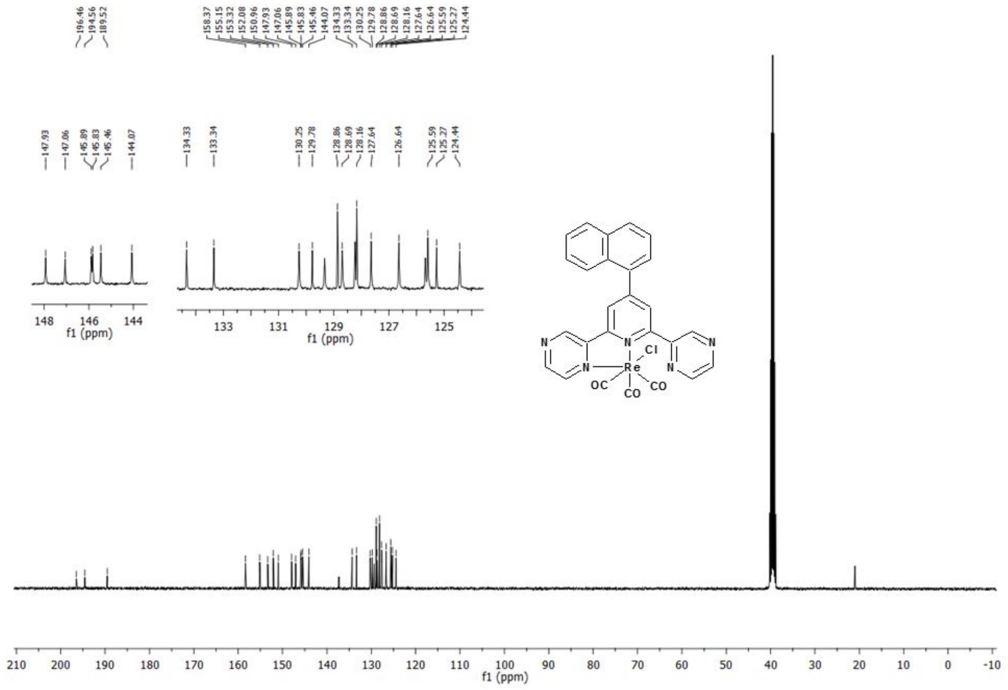

b)

Figure S5. NMR spectra of **1B** in DMSO-d$_6$: ^{1}H (a) and ^{13}C (b).

Complex 2B

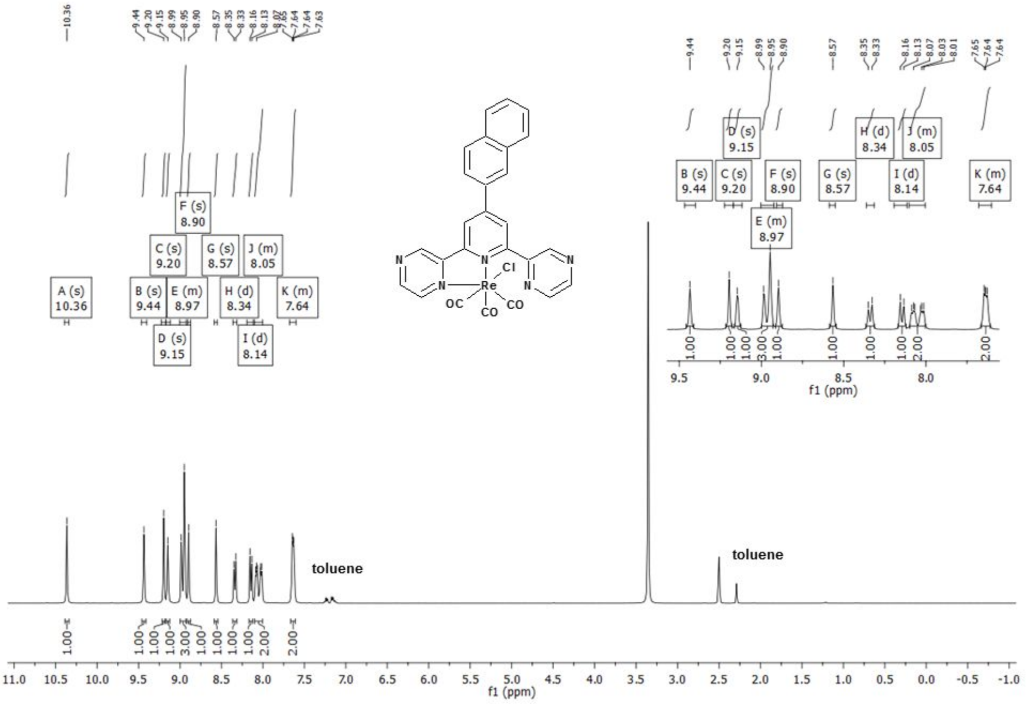

a)

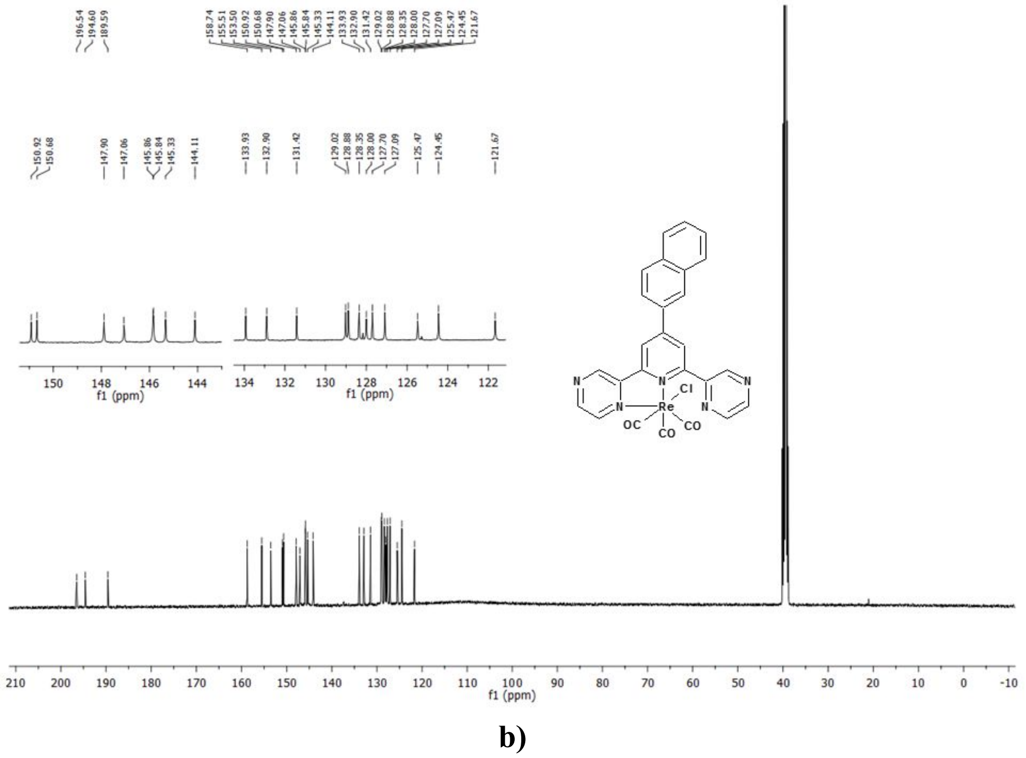

b)

Figure S6. NMR spectra of **2B** in DMSO-d$_6$: ^{1}H (a) and ^{13}C (b).

Complex 3B

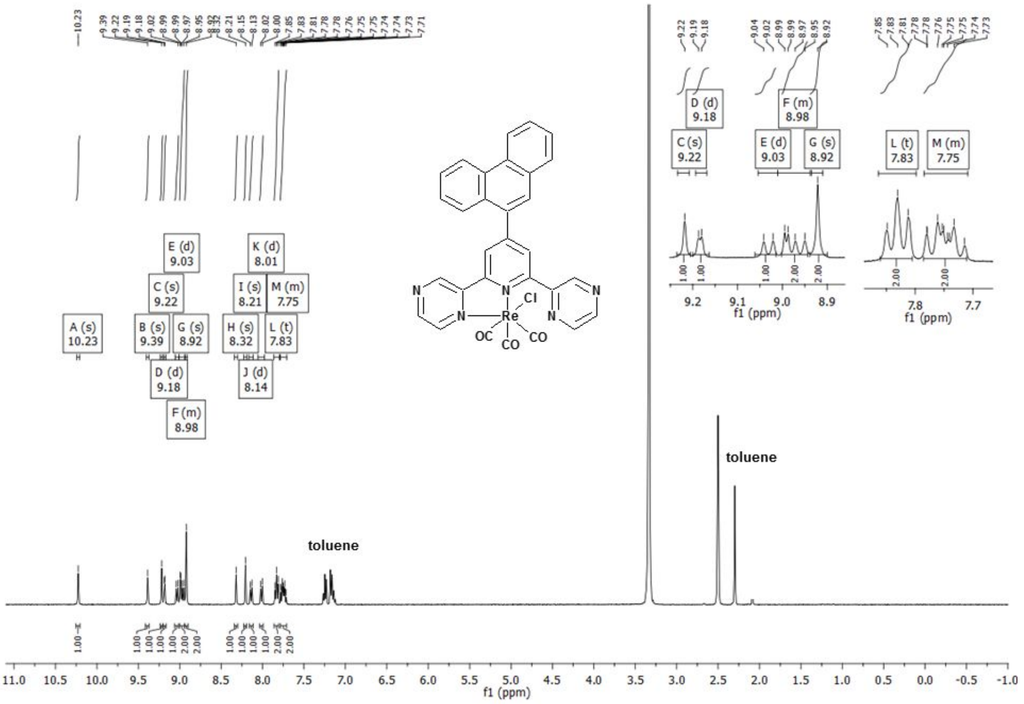

a)

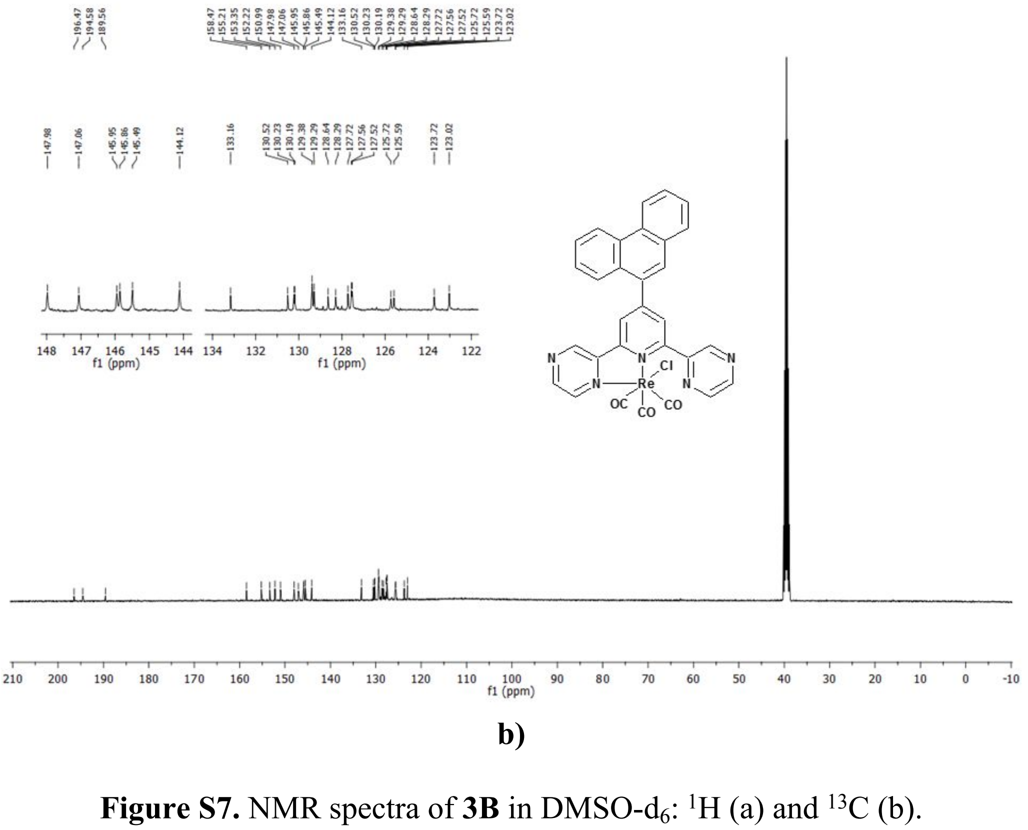

b)

Figure S7. NMR spectra of **3B** in DMSO-d$_6$: ^{1}H (a) and ^{13}C (b).

Complex 4B

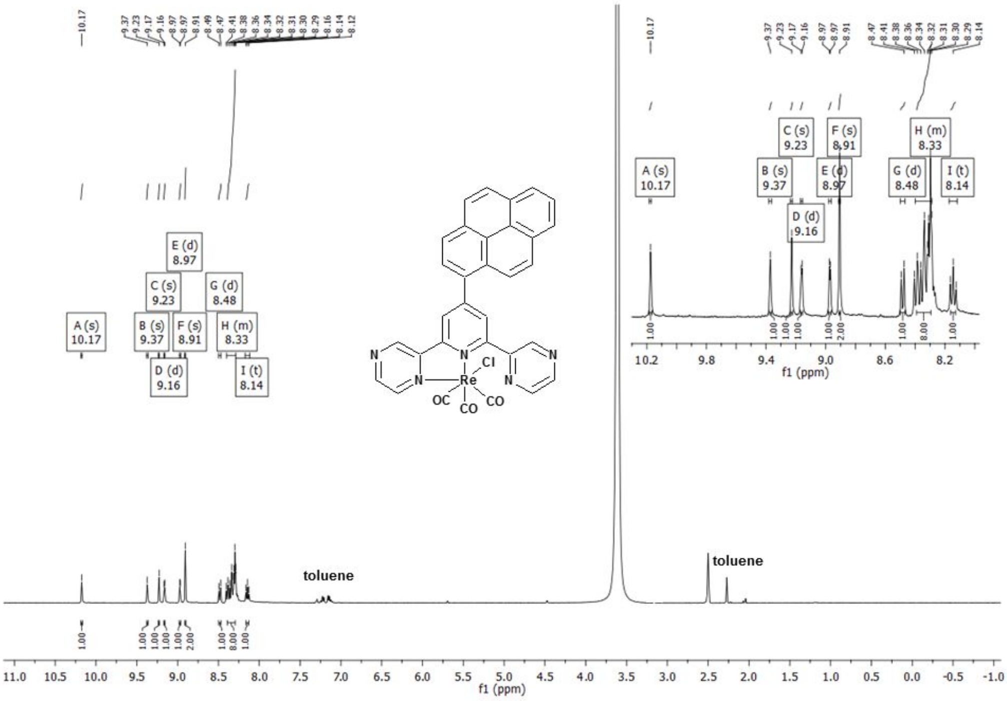

a)

Figure S8. NMR spectra of **4B** in DMSO-d_6: ^{1}H (a) and ^{13}C NMR not recorded due to insufficient complex solubility.

IR spectra

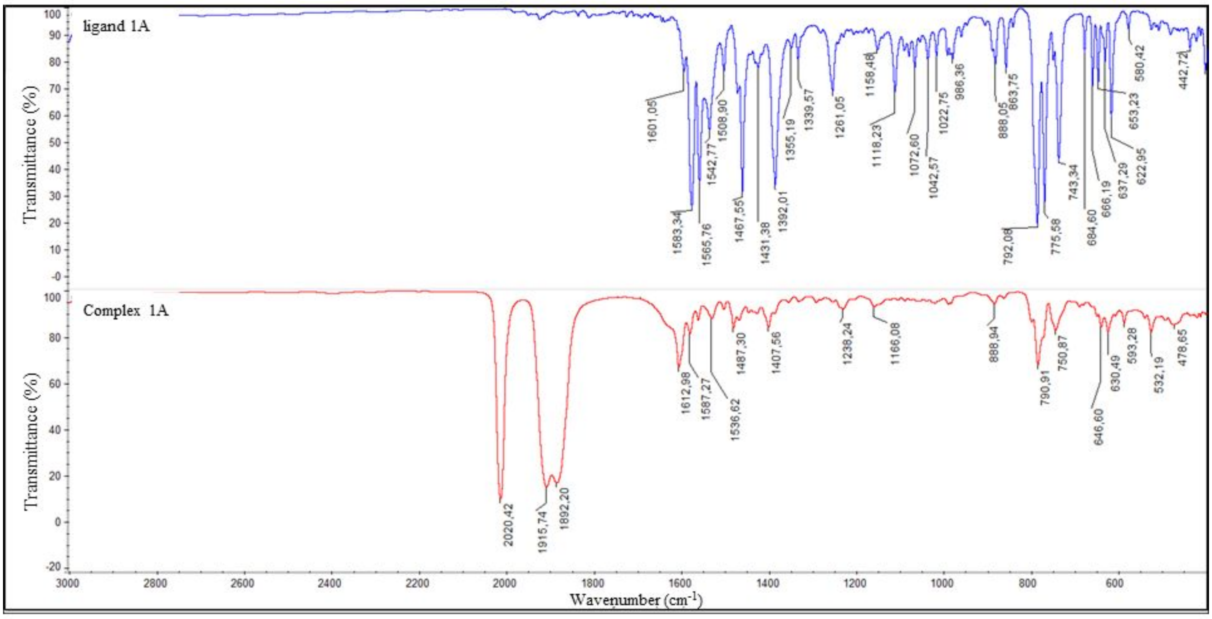

Figure S9. FT-IR spectrum of **1A** along with FT-IR spectrum of 4′-Ar[1]-terpy.

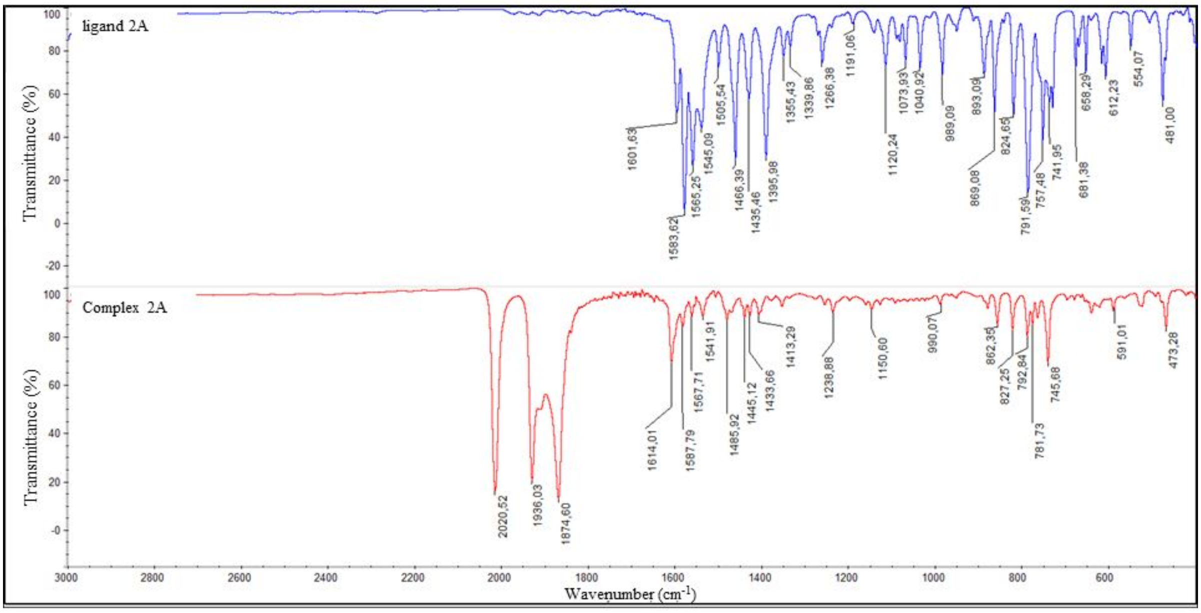

Figure S10. FT-IR spectrum of **2A** along with FT-IR spectrum of 4′-Ar[2]-terpy.

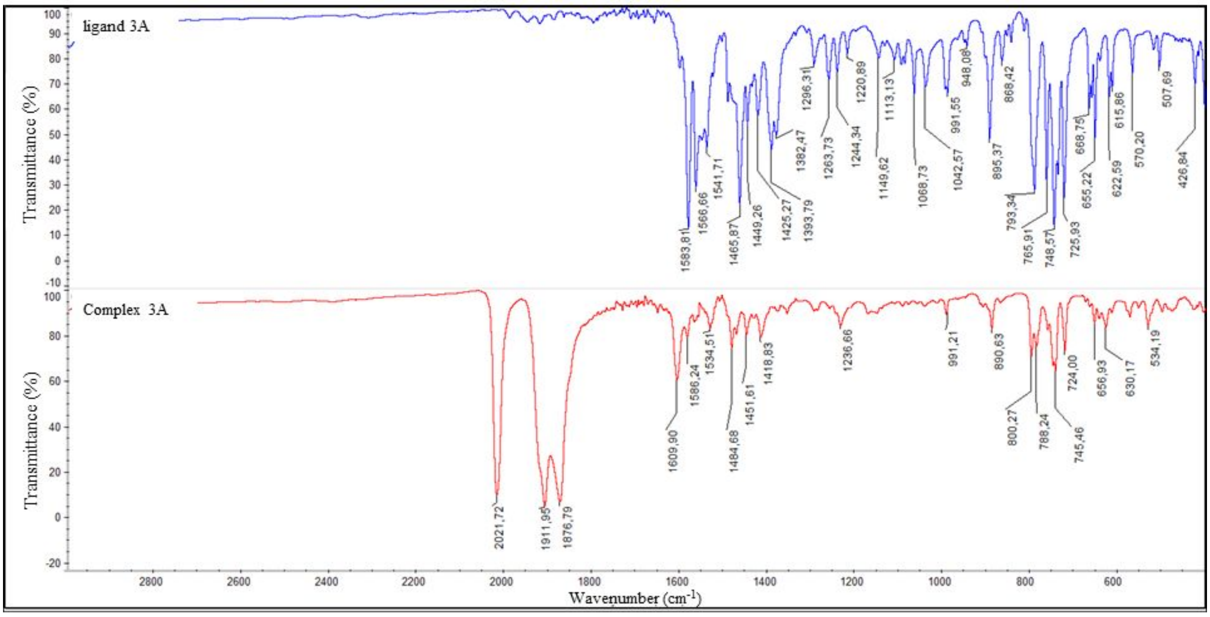

Figure S11. FT-IR spectrum of 3A along with FT-IR spectrum of 4′-Ar^3-terpy.

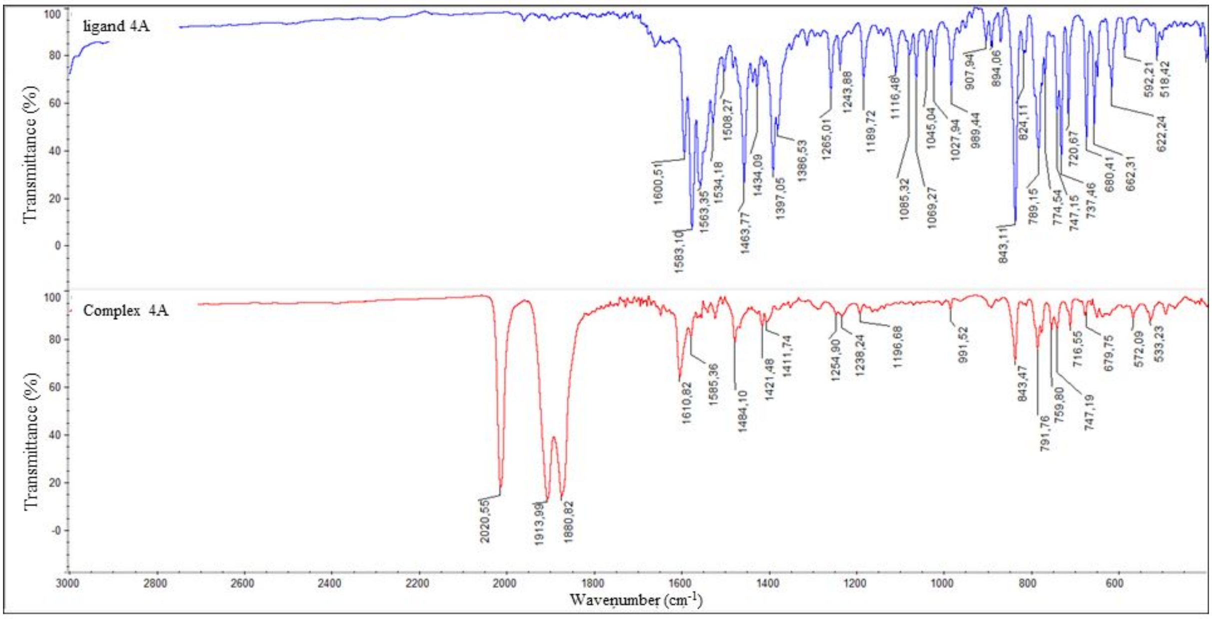

Figure S12. FT-IR spectrum of **4A** along with FT-IR spectrum of 4′-Ar^4-terpy.

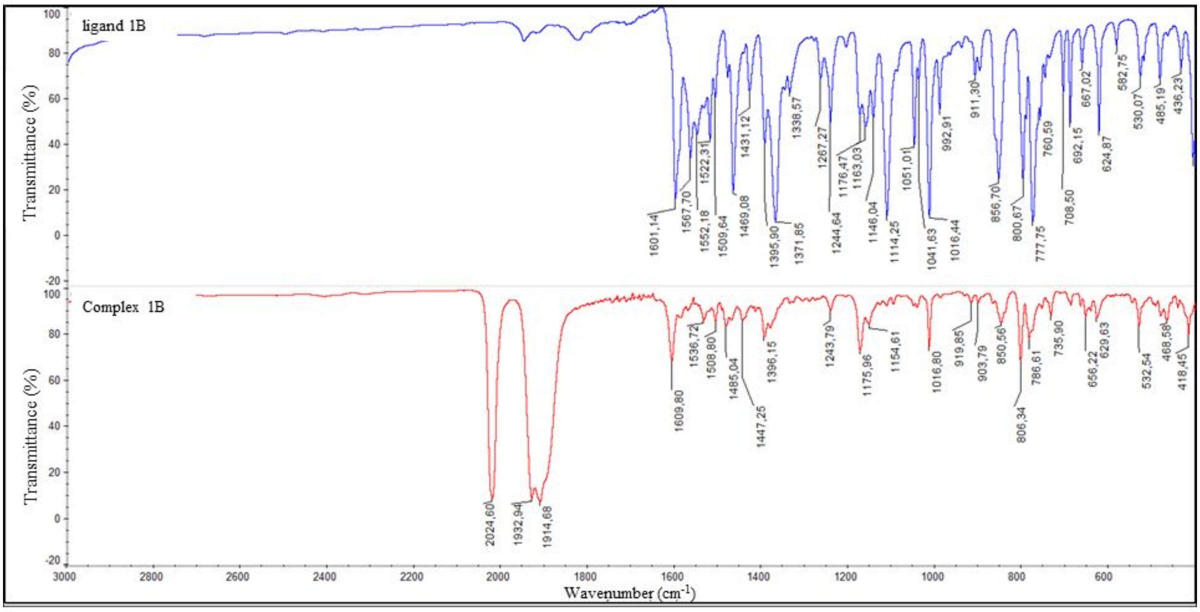

Figure S13. FT-IR spectrum of **1B** along with FT-IR spectrum of 4-Ar[1]-dppy.

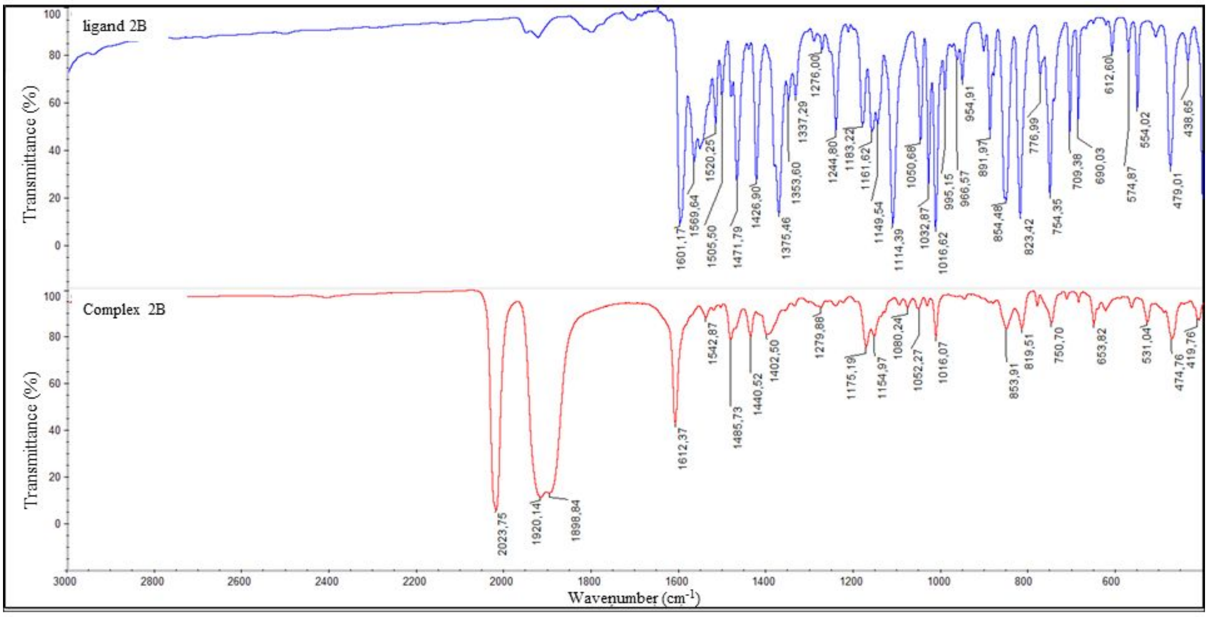

Figure S14. FT-IR spectrum of **2B** along with FT-IR spectrum of 4-Ar[2]-dppy.

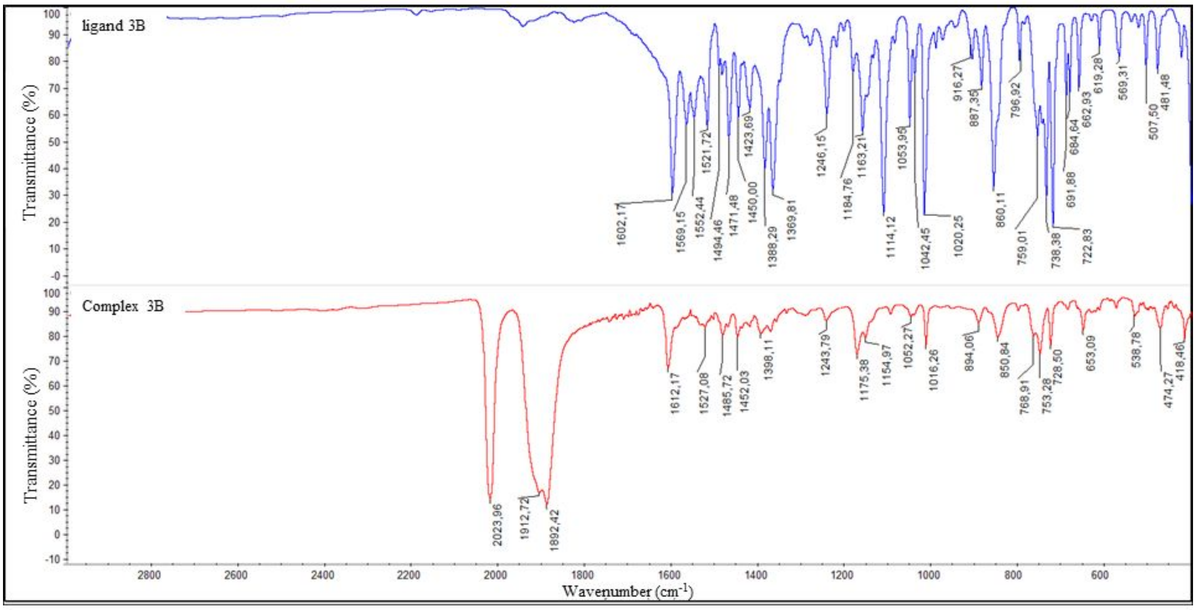

Figure S15. FT-IR spectrum of 3B along with FT-IR spectrum of 4-Ar^3-dppy.

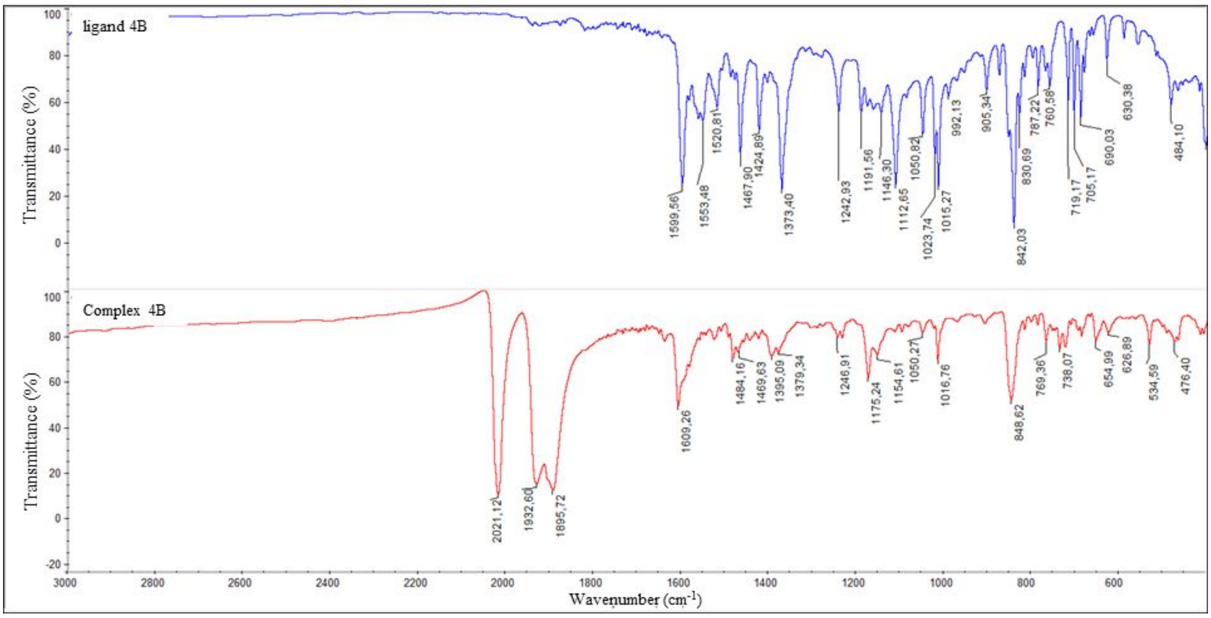

Figure S16. FT-IR spectrum of **4B** along with FT-IR spectrum of 4-Ar^4-dppy.

HR-MS spectrometry

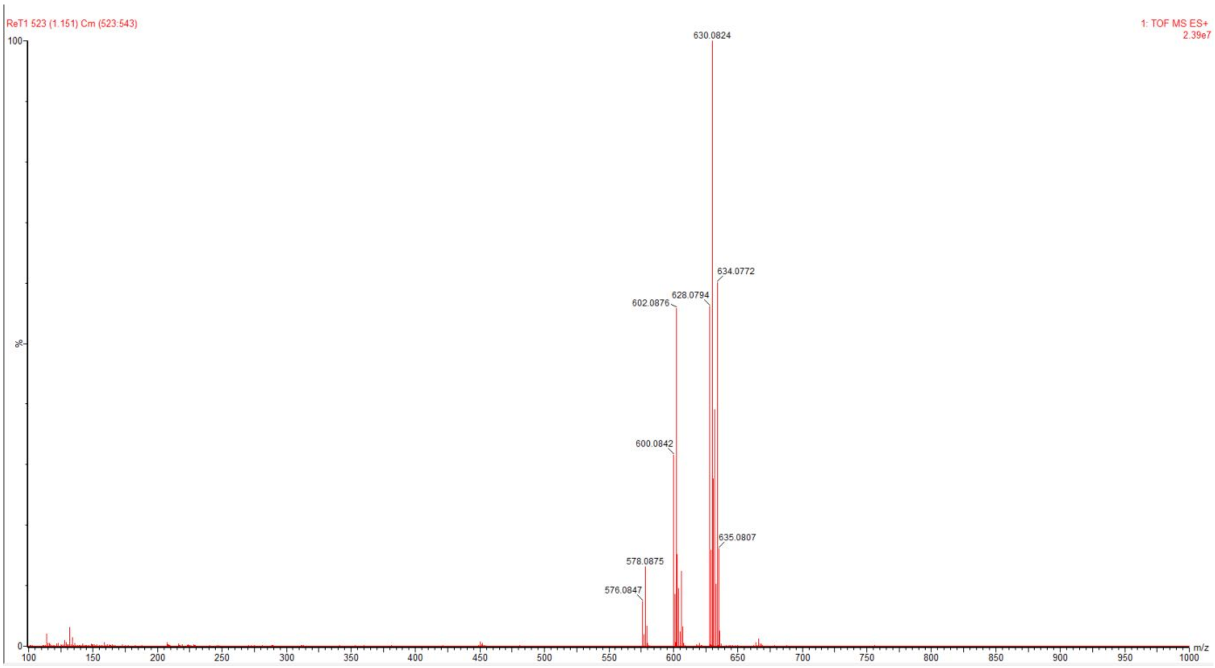

(a)

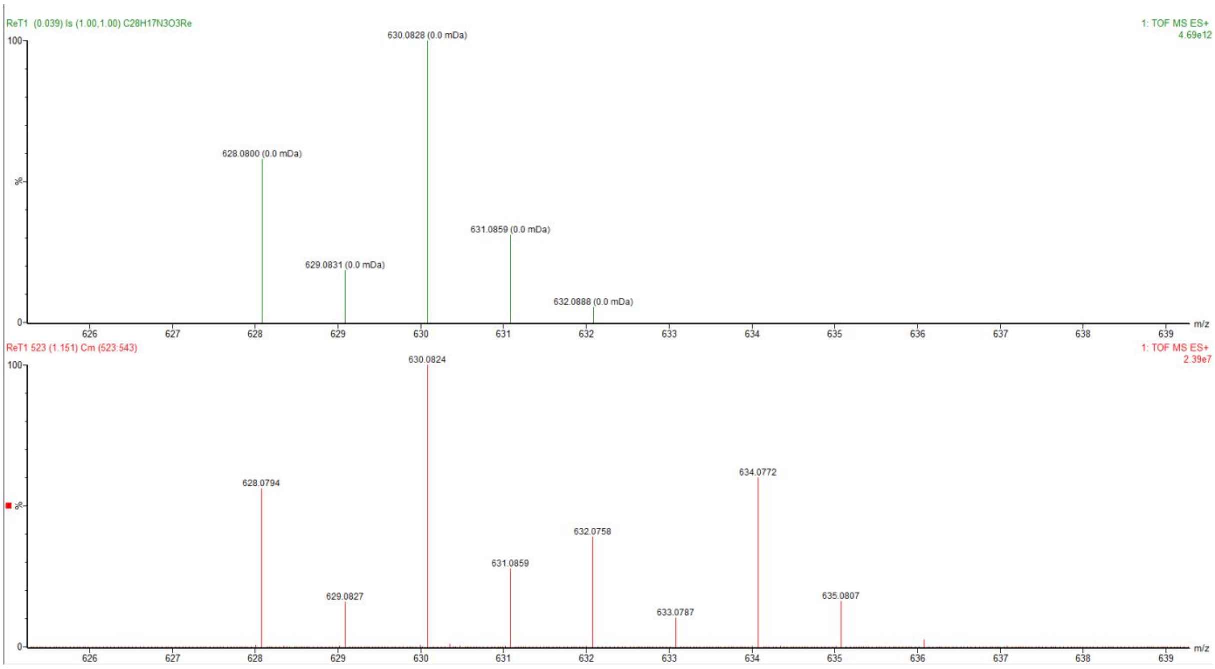

(b)

Figure S17. HRMS spectrum of the complex **1A** (a) full range, (b) isotope simulation

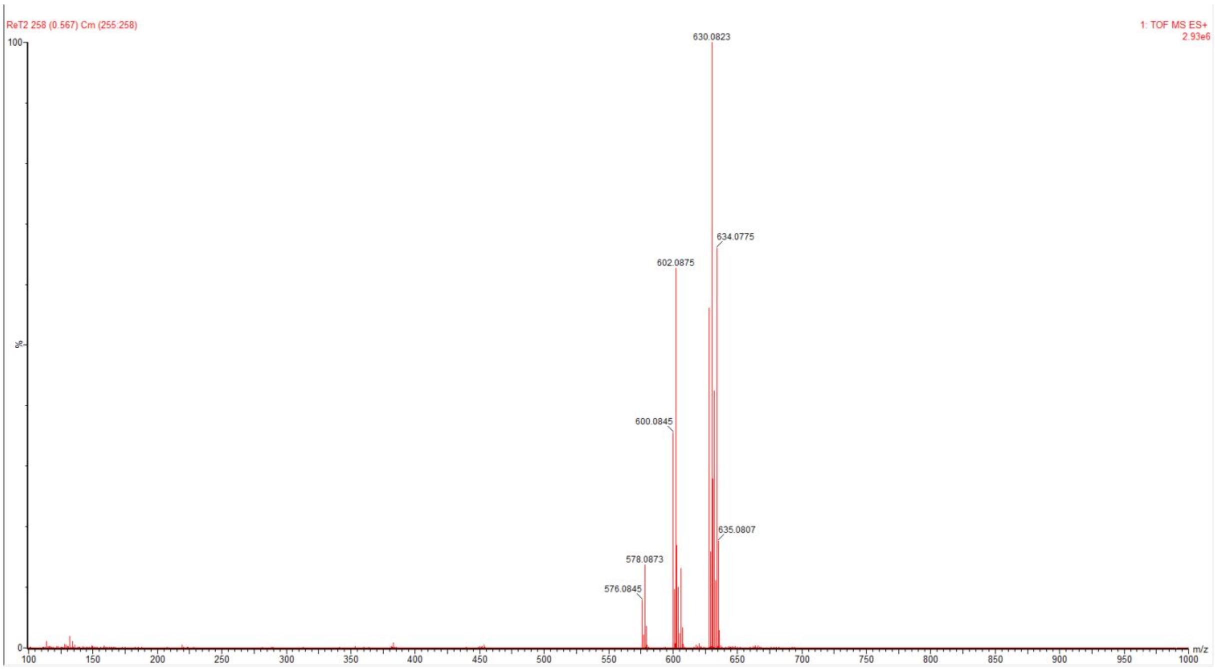

(a)

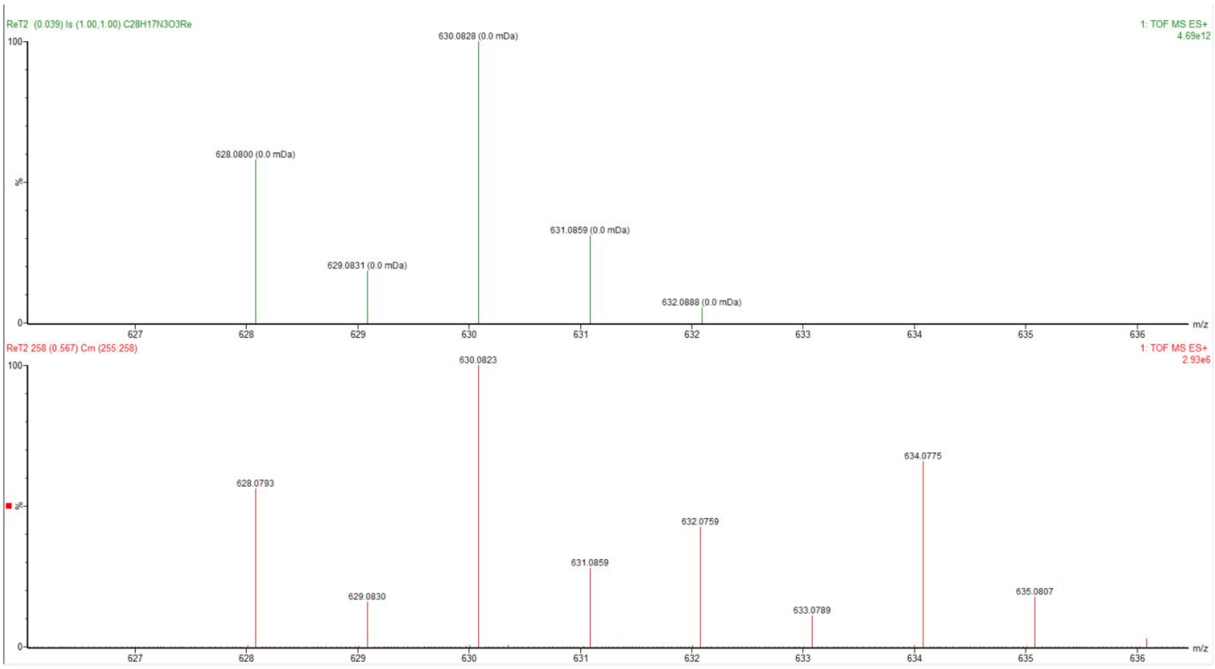

(b)

Figure S18. HRMS spectrum of the complex **2A** (a) full range, (b) isotope simulation.

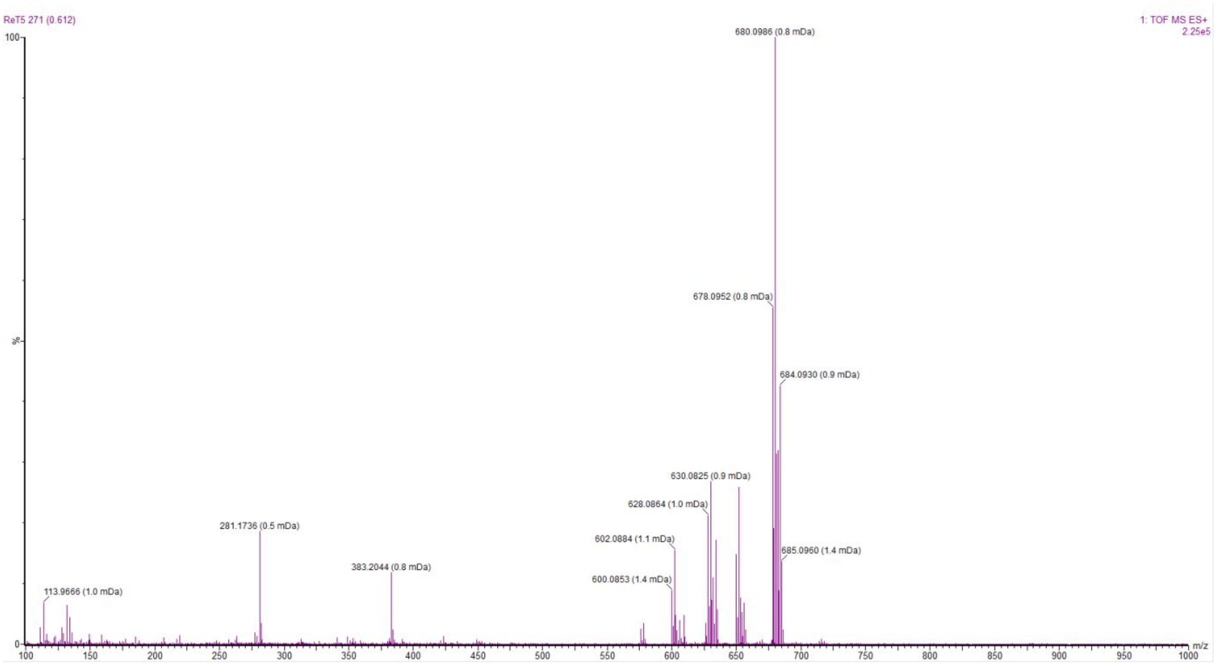

(a)

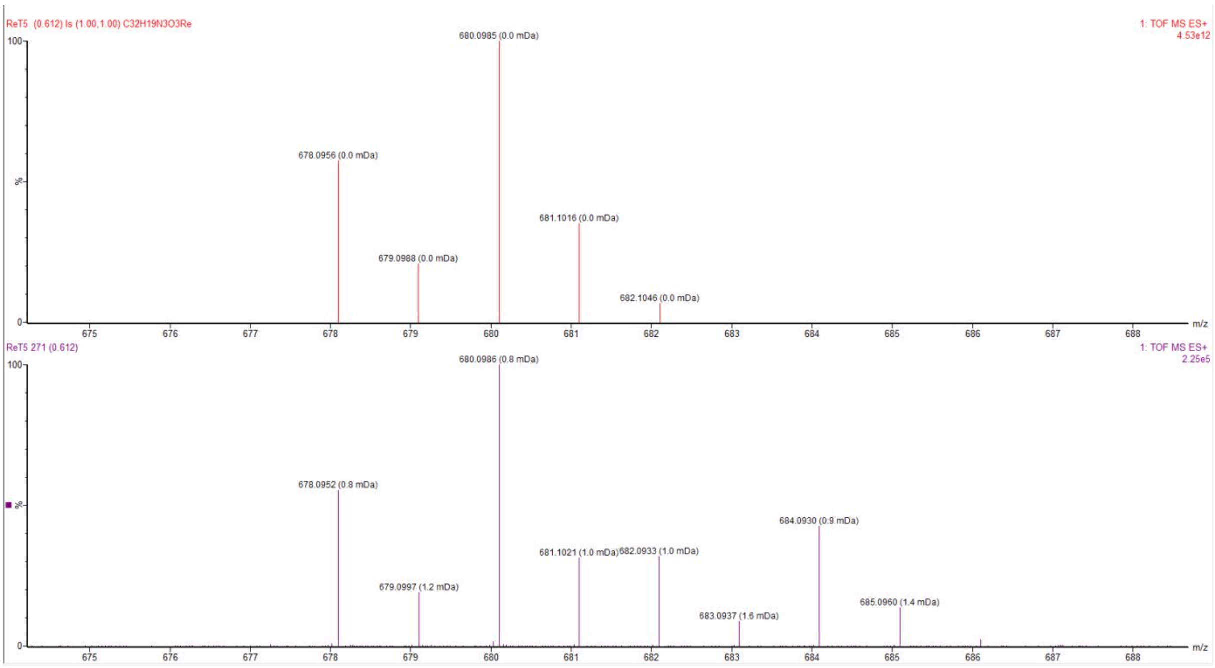

(b)

Figure S19. HRMS spectrum of the complex **3A** (a) full range, (b) isotope simulation.

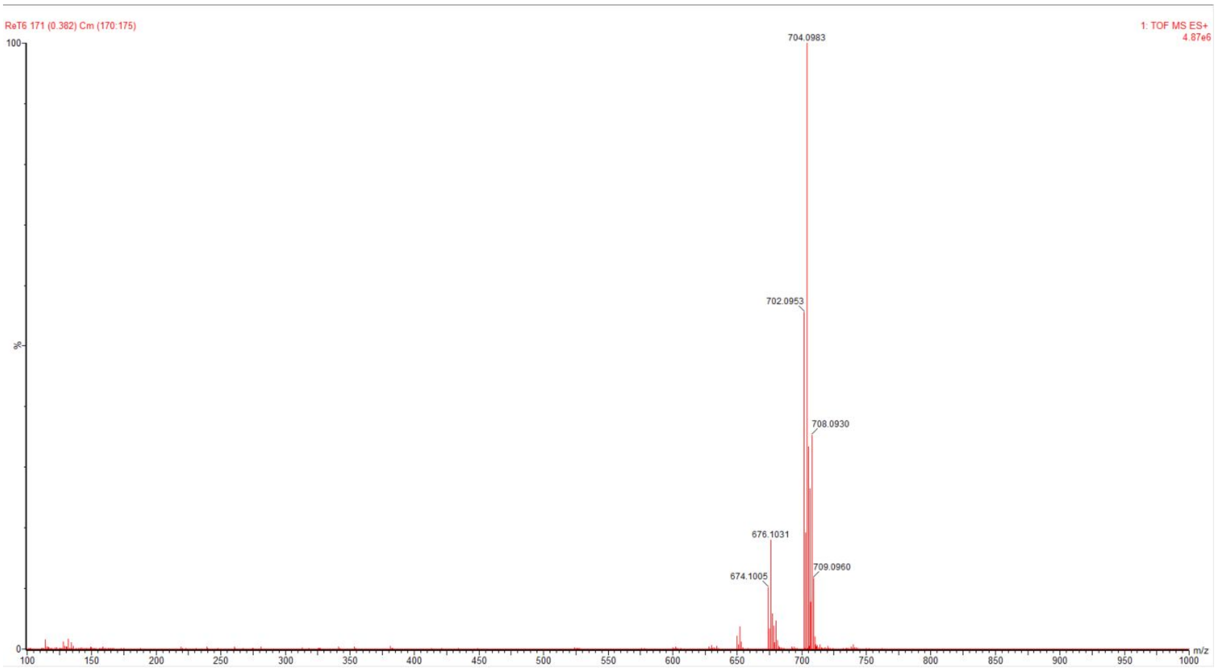

(a)

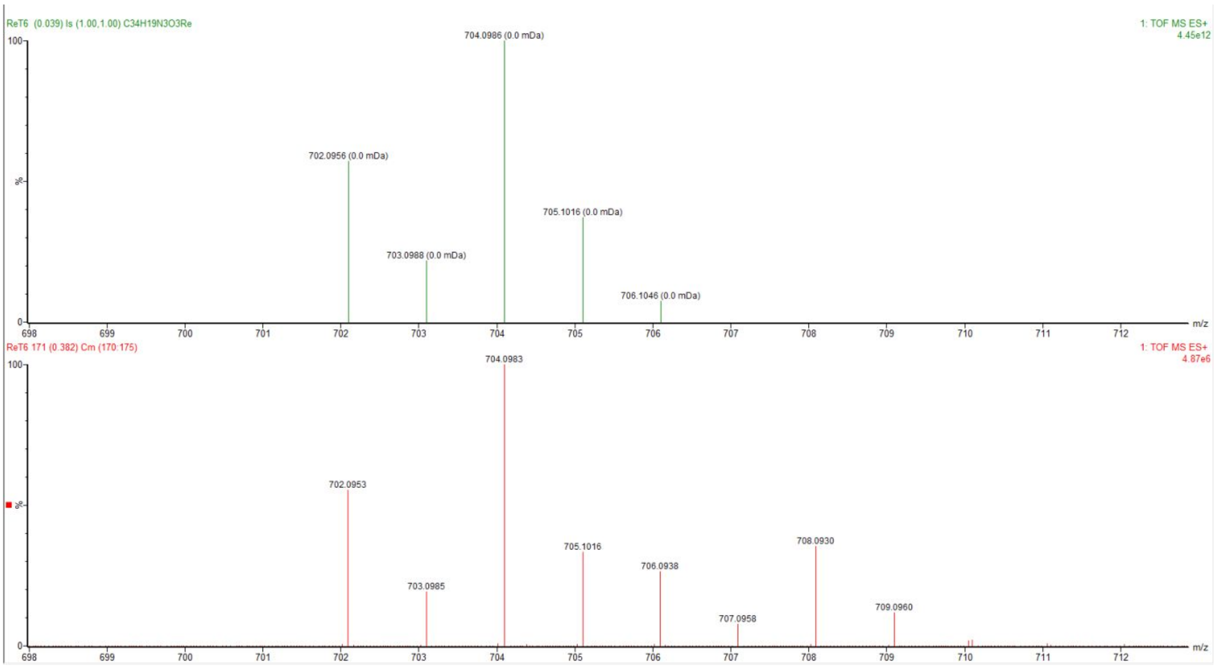

(b)

Figure S20. HRMS spectrum of the complex **4A** (a) full range, (b) isotope simulation.

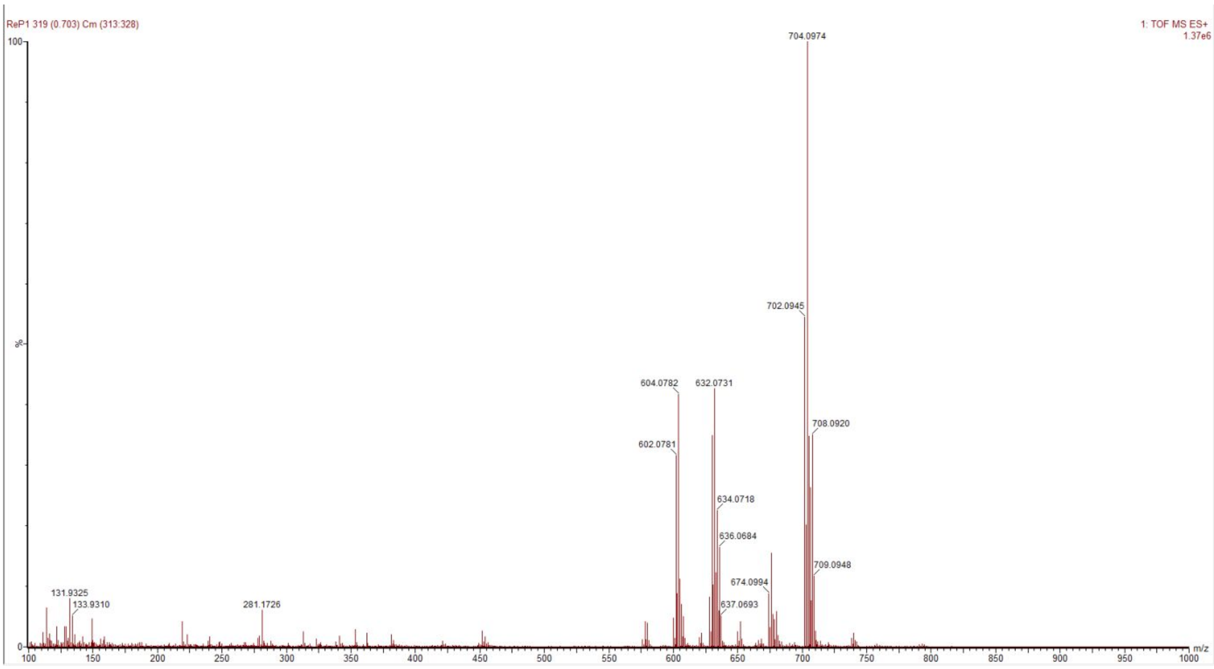

(a)

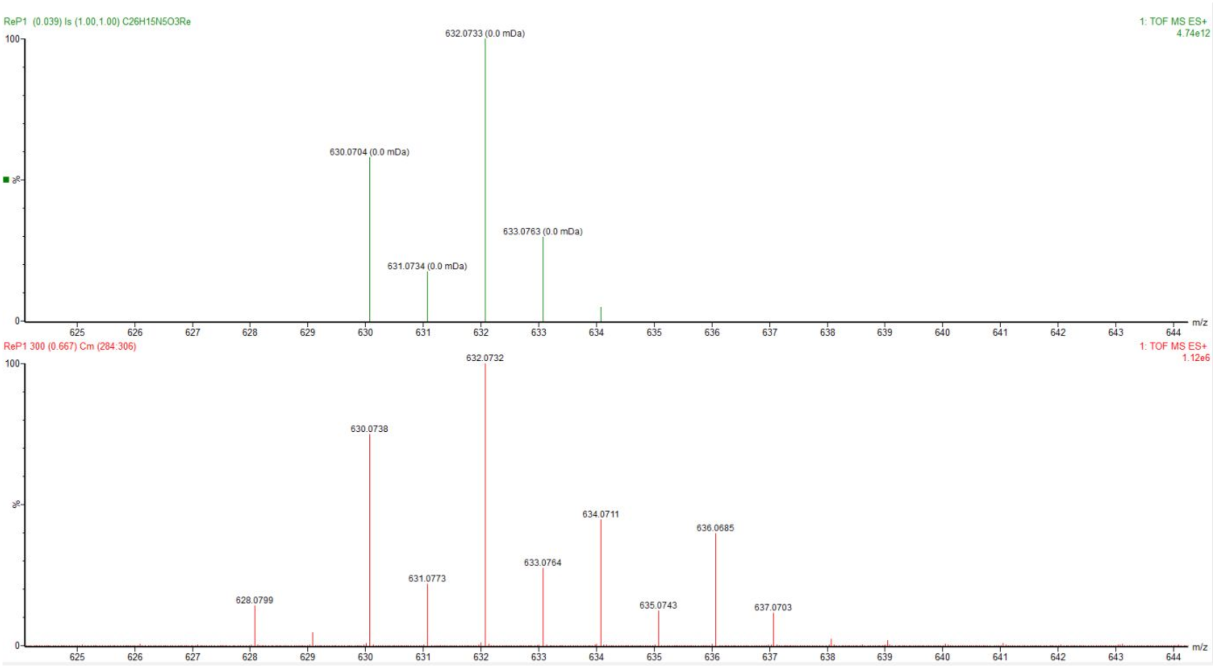

(b)

Figure S21. HRMS spectrum of the complex **1B** (a) full range, (b) isotope simulation.

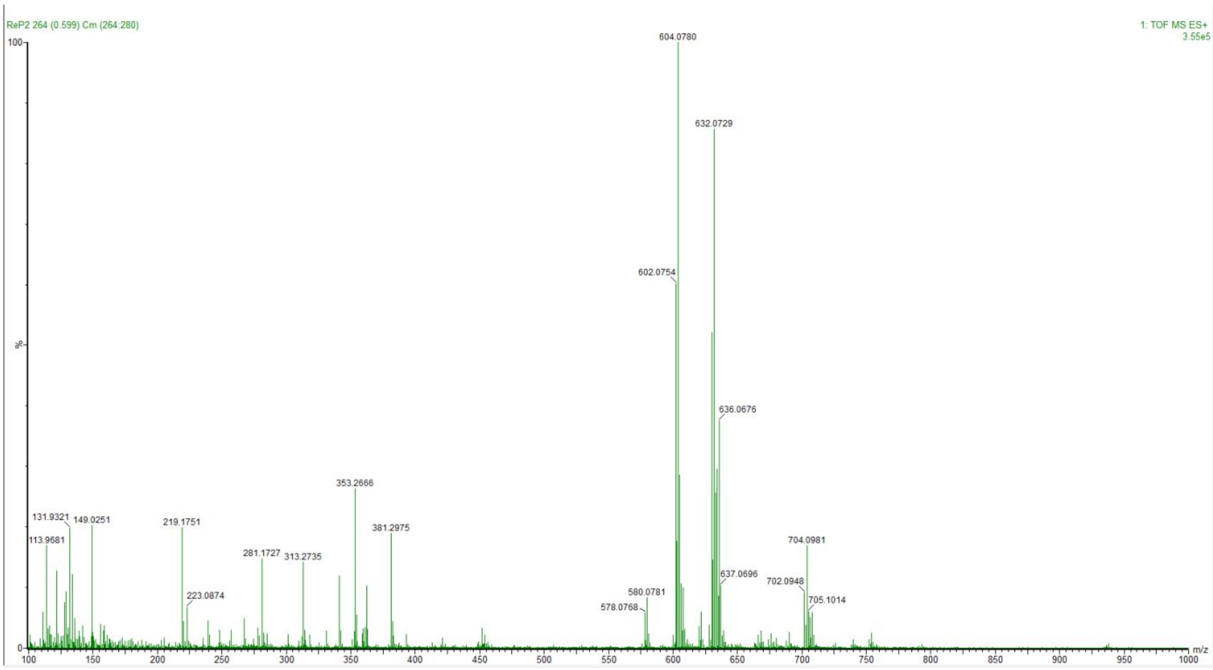

(a)

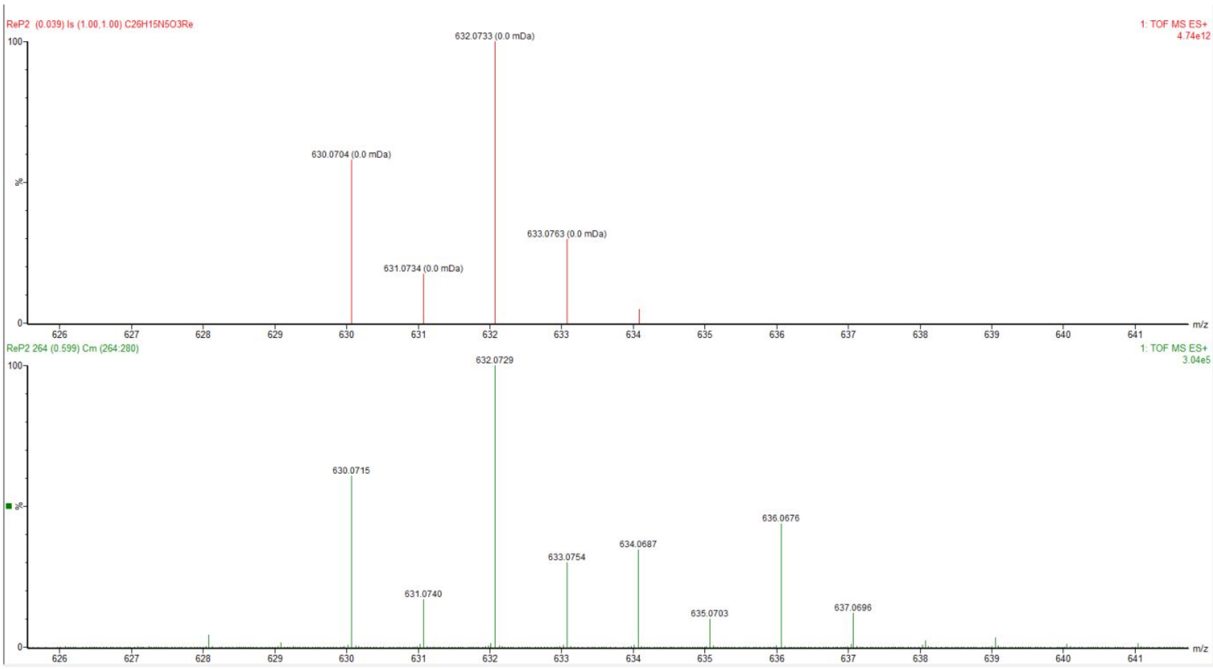

(b)

Figure S22. HRMS spectrum of the complex **2B** (a) full range, (b) isotope simulation.

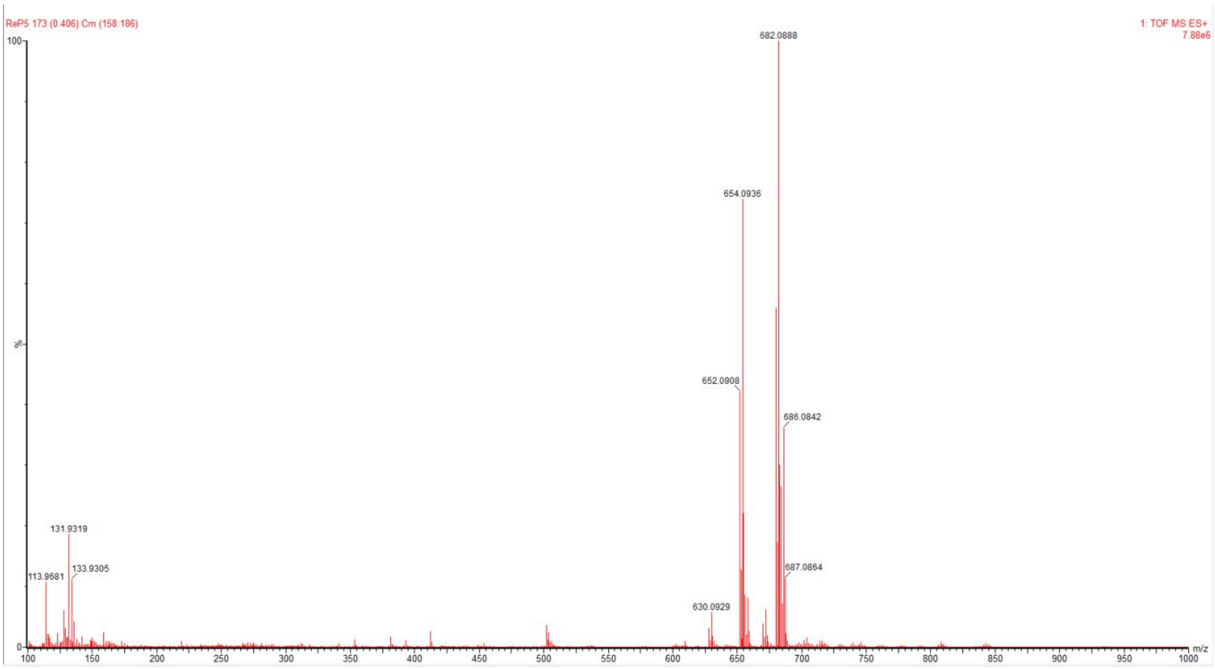

(a)

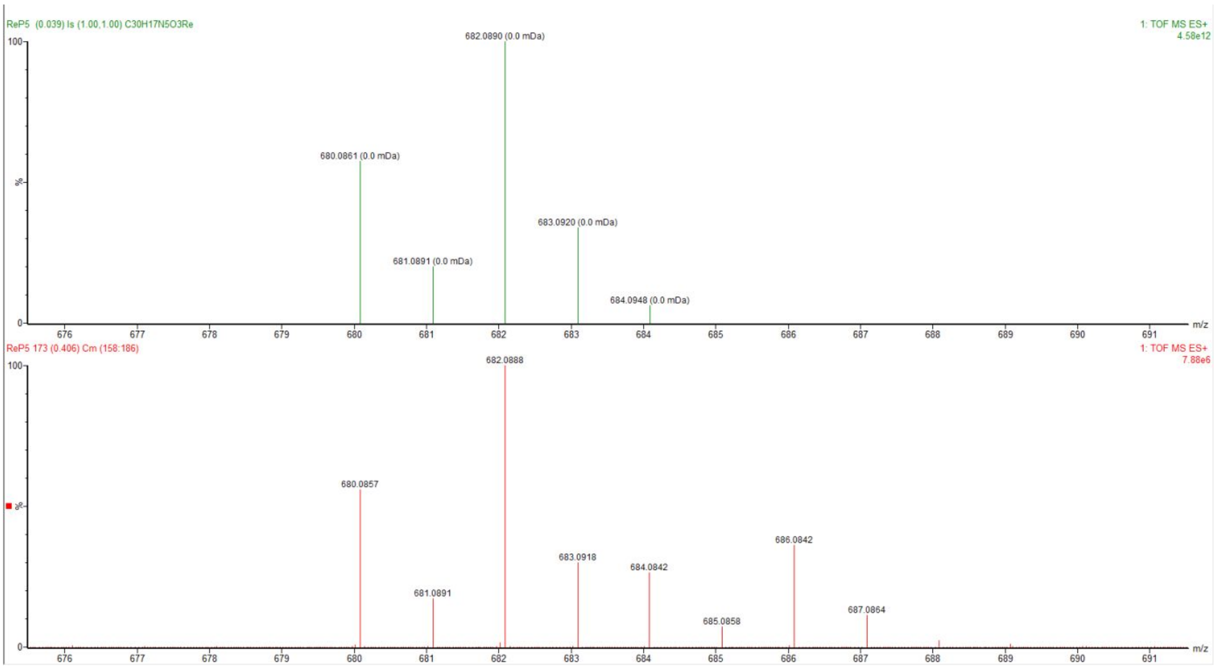

(b)

Figure S23. HRMS spectrum of the complex **3B** (a) full range, (b) isotope simulation.

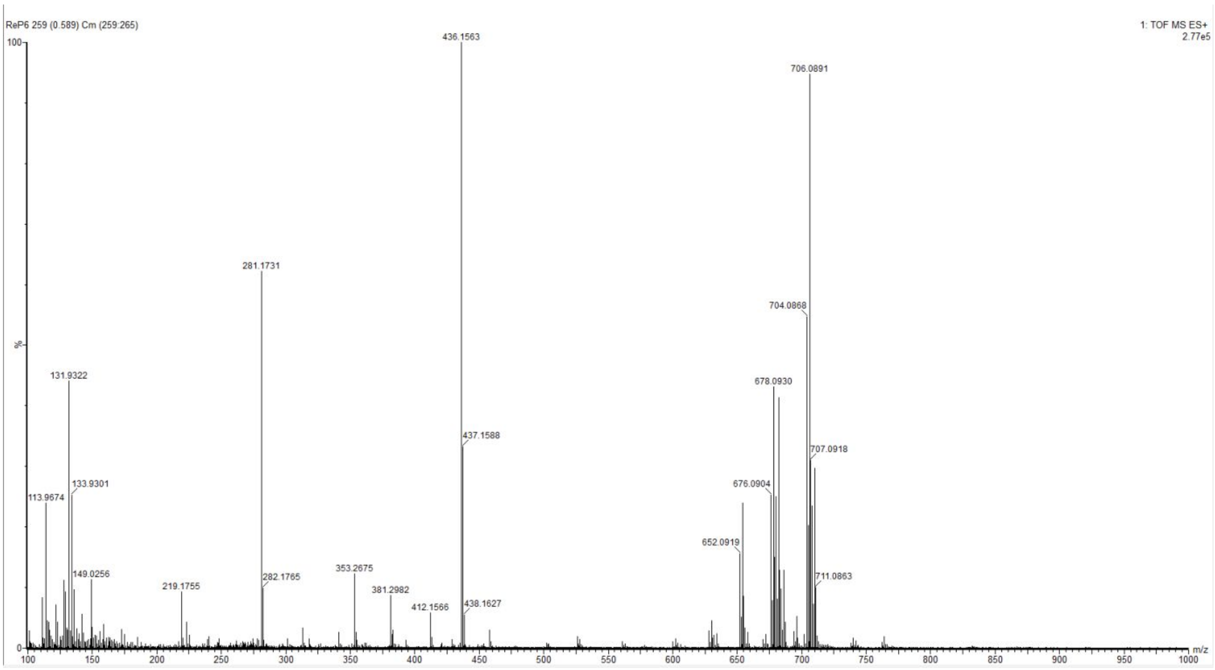

(a)

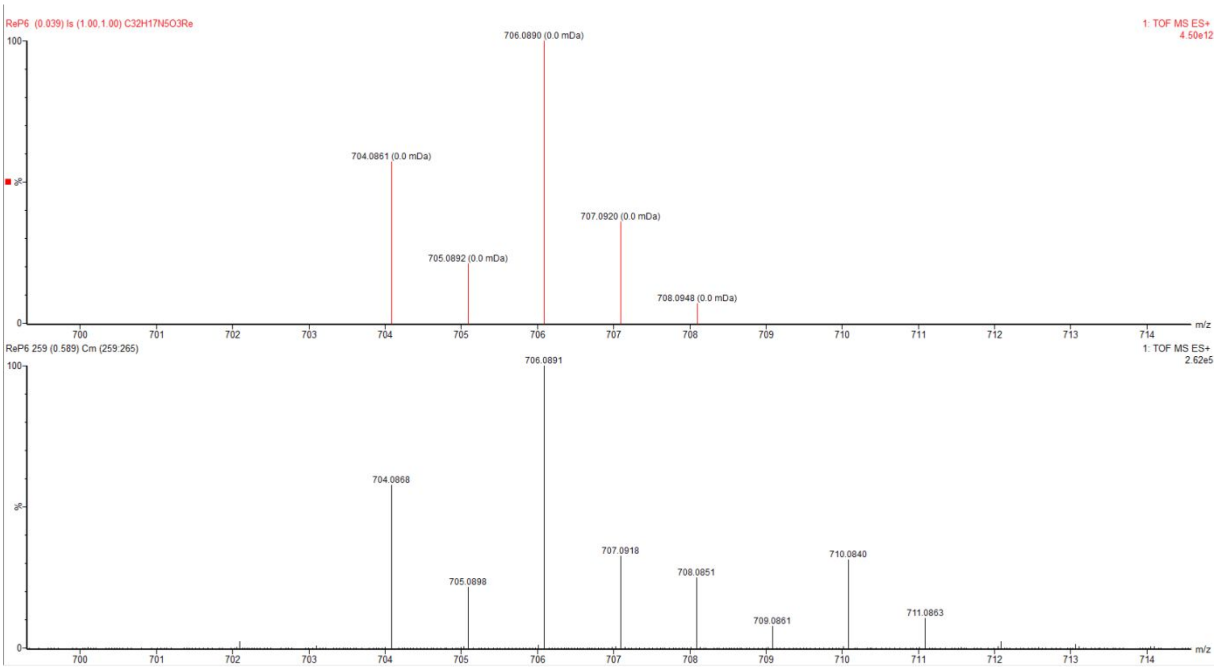

(b)

Figure S24. HRMS spectrum of the complex **4B** (a) full range, (b) isotope simulation**..**

X-Ray analysis

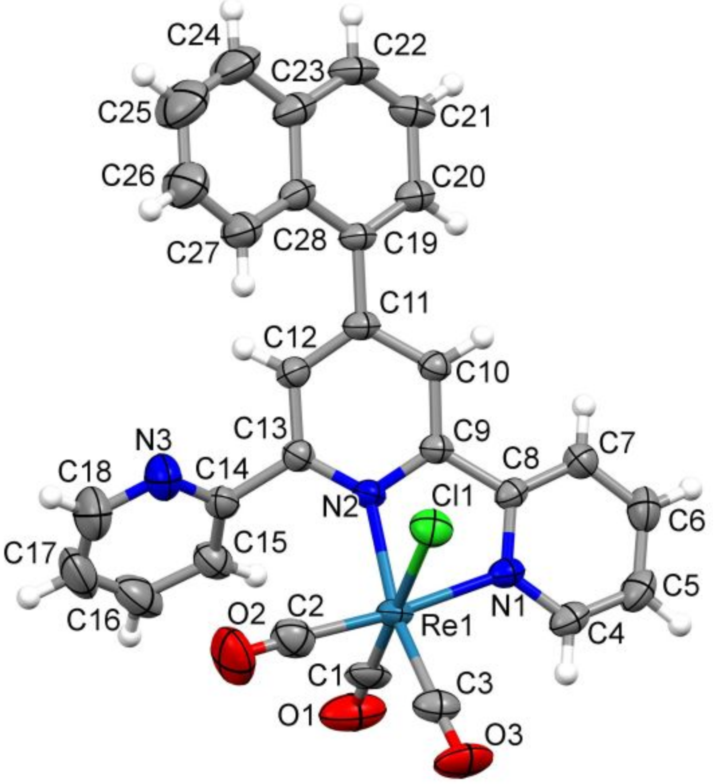

Figure S25. The molecular structure of **1A** with thermal ellipsoids set at 50% probability for non-hydrogen atoms.

Table S2. Crystal data and structure refinement of the complex 1A.

Empirical formula	$C_{28}H_{17}ClN_3O_3Re$
Formula weight	665.09
Temperature [K]	295(2)
Wavelength [Å]	0.71073
Crystal system	triclinic
Space group	$P\bar{1}$
Unit cell dimensions [Å, °]	a = 7.6725(3)
	b = 13.7812(5)
	c = 16.2426(6)
	α = 66.844(4)
	β = 80.493(3)
	γ = 74.284(3)
Volume [Å ^{3}]	1516.70(11)
Z	2
Density (calculated) [Mg/m^3]	1.456
Absorption coefficient [mm^{-1}]	4.123
F(000)	644
Crystal size [mm]	0.14 x 0.16 x 0.35
θ range for data collection [°]	3.3 to 29.5
Index ranges	$-10 \leq h \leq 9$
	$-18 \leq k \leq 16$
	$-21 \leq l \leq 20$
Reflections collected	16162
Independent reflections	7285 (R_{int} = 0.034)
Completeness to 2θ=50.5° [%]	99.81
Max. and min. transmission	1.000 and 0.30836
Data / restraints / parameters	7285 / 0 / 325
Goodness-of-fit on F^2	1.048
Final R indices [I>2σ(I)]	R_1 = 0.0283
	wR_2 = 0.0683
R indices (all data)	R_1 = 0.0334
	wR_2 = 0.0702
Largest diff. peak and hole [eÅ^{-3}]	0.820 and -0.693
CCDC number*	2094604

* Crystallographic data for **1A** were deposited with the Cambridge Crystallographic Data Center, CCDC 2094604. Copies of this information may be obtained free of charge from the Director, CCDC, 12 Union Road, Cambridge CB2 1EZ, UK (Fax: +44 1223 336033; e-mail: deposit@ccdc.cam.ac.uk or www.ccdc.cam.ac.uk).

Table S3. Bond lengths [Å] and angles [°] for **1A**.

Bond lengths	[Å]	Bond angles	[°]
Re(1)–C(1)	1.948(4)	C(2)–Re(1)–C(1)	90.57(17)
Re(1)–C(2)	1.921(4)	C(3)–Re(1)–C(1)	90.69(14)
Re(1)–C(3)	1.913(4)	C(3)–Re(1)–C(2)	87.81(17)
Re(1)–N(1)	2.164(3)	C(1)–Re(1)–N(1)	92.57(14)
Re(1)–N(2)	2.218(3)	C(2)–Re(1)–N(1)	174.53(14)
Re(1)–Cl(1)	2.4847(9)	C(3)–Re(1)–N(1)	96.62(14)
C(1)–O(1)	1.076(5)	C(1)–Re(1)–N(2)	97.00(11)
C(2)–O(2)	1.148(5)	C(2)–Re(1)–N(2)	100.79(14)
C(3)–O(3)	1.145(4)	C(3)–Re(1)–N(2)	168.35(12)
		N(1)–Re(1)–N(2)	74.38(10)
		C(1)–Re(1)–Cl(1)	177.27(10)
		C(2)–Re(1)–Cl(1)	91.46(13)
		C(3)–Re(1)–Cl(1)	91.22(10)
		N(1)–Re(1)–Cl(1)	85.27(7)
		N(2)–Re(1)–Cl(1)	80.82(7)

Table S4. Short π•••π interactions for **1A**.

Cg(I)•••Cg(J)	Cg(I)•••Cg(J) [Å]	α[°]	β[°]	γ [°]	Cg(I)-Perp [Å]	Cg(J)-Perp [Å]
Cg(2)•••Cg(5)[a]	3.870(2)	13.80(18)	28.0	21.1	3.6114(15)	3.4177(16)
Cg(2)•••Cg(5)[b]	3.839(2)	13.80(18)	21.9	21.3	3.5755(15)	3.5612(16)

α = dihedral angle between *Cg*(I) and *Cg*(J); *Cg*(I)-Perp = Perpendicular distance of *Cg*(I) on ring J; *Cg*(J)-Perp = perpendicular distance of *Cg*(J) on ring I; β = angle *Cg*(I)→*Cg*(J) vector and normal to ring I; γ = angle *Cg*(I) →*Cg*(J) vector and normal to plane J;

Cg(2) is the centroid of atoms N(1)/C(4)/C(5)/ C(6)/C(7)/C(8);

Cg(5) is the centroid of atoms C(19)/C(20)/C(21)/C(22)/C(23)/C(28);

Symmetry codes: (a) = 2-*x*,1-*y*,1-*z*; (b) = 3-*x*,1-*y*,1-*z*;

Table S5. X—Y•••Cg(J)(π-ring) interactions for **1A**.

Y-X(I)•••Cg(J)	X(I)•••Cg(J) [Å]	X-Perp [Å]	γ [°]	Y-X(I)•••Cg(J) [°]
C(2)-O(2)•••Cg(4)[c]	3.340(5)	-3.394	9.85	84.3(3)

γ = angle X(I)→Cg(J) vector and normal to plane J;

Cg(4) is the centroid of atoms N(3)/C(14)/C(15)/ C(16)/C(17)/C(18)

Symmetry codes: (c) = *x*, *y*, *z*;

Thermal properties

The thermal properties of Re(I) complexes were studied using DSC upon two heating scans, and the results are summarized in Table S6 below:

Table S6. Thermal properties of Re(I) complexes

Compounds	DSC				
	I heating scan		II heating scan		
	T_c [°C]	T_m [°C]	T_g [°C]	T_c [°C]	T_m [°C]
1A*	272	266, 291	175	280	301
2A**	–	288, 298	196	–	–
3A	–	297	238	–	–
4A	–	322, 349	257	–	–
1B	–	242	175	–	–
2B	–	305***	–	–	–
3B	–	255	215	–	–
4B****	–	298	232	–	–

* I heating scan: crystal→ crystal transition=147, 169°C

** I heating scan: crystal→ crystal transition=209°C;

*** with decomposition

**** I heating scan: crystal→ crystal transition=154°C

In the case of three complexes, i.e. **1A**, **2A** and **4B**, multiple endo- and exothermic peaks are seen, as shown in the representative DSC thermograms for the complex **2A**. The endotherm peaks registered at lower temperatures are caused by solid-to-solid - probably crystal-to-crystal transitions.

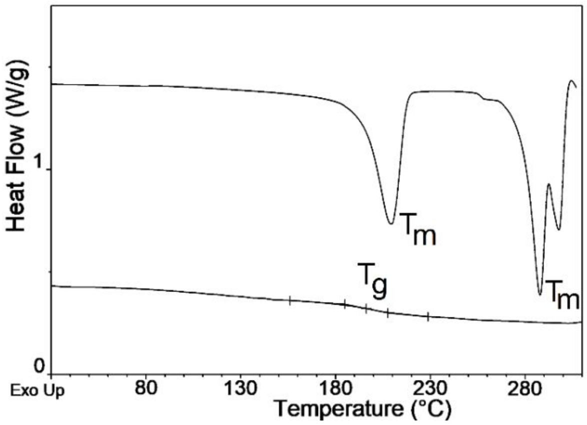

Figure S26. DSC thermograms for the complex **2A**.

Except for **2B**, which melted with thermal decomposition, the obtained Re(I) complexes are molecular glasses. They exhibited very high glass transition temperature (T_g), falling in the range of 175-257 °C. As no crystallization and melting were observed during further heating above T_g, it can be assumed that examined Re(I) complexes form stable amorphous materials.

Only in the case of **1A**, heating above T_g revealed crystallization (T_c) exotherm, and then the melting (T_m) process. The thermal behaviour of **1A**–**4A** and **1B**–**4B** was affected by both triimine core and aromatic group. The compounds with *dppy* ligands exhibited lower T_m and T_g relative to is analogues with *terpy*, except for **1A** and **1B**, which showed the same value of T_g. Considering aryl substituents, it was found that the presence of 9-phenanthrenyl and 1-pyrenyl substituents increase both T_m and T_g.

DFT calculations

Table S7. Experimental and theoretical bond lengths [Å] and angles [°] for **1A**.

	**1A**	
	experimental	theoretical
**Bond lengths**		
Re(1)–C(1)	1.948(4)	1.901
Re(1)–C(2)	1.921(4)	1.923
Re(1)–C(3)	1.913(4)	1.906
Re(1)–N(1)	2.164(3)	2.179
Re(1)–N(2)	2.218(3)	2.240
Re(1)–Cl(1)	2.4847(9)	2.499
C(1)–O(1)	1.076(5)	1.155
C(2)–O(2)	1.148(5)	1.149
C(3)–O(3)	1.145(4)	1.153
**Bond angles**		
C(2)–Re(1)–C(1)	90.57(17)	89.94
C(3)–Re(1)–C(1)	90.69(14)	88.51
C(3)–Re(1)–C(2)	87.81(17)	85.88
C(1)–Re(1)–N(1)	92.57(14)	93.89
C(2)–Re(1)–N(1)	174.53(14)	175.05
C(3)–Re(1)–N(1)	96.62(14)	97.34
C(1)–Re(1)–N(2)	97.00(11)	97.32
C(2)–Re(1)–N(2)	100.79(14)	102.09
C(3)–Re(1)–N(2)	168.35(12)	170.07
N(1)–Re(1)–N(2)	74.38(10)	74.33
C(1)–Re(1)–Cl(1)	177.27(10)	178.13
C(2)–Re(1)–Cl(1)	91.46(13)	91.83
C(3)–Re(1)–Cl(1)	91.22(10)	92.23
N(1)–Re(1)–Cl(1)	85.27(7)	84.31
N(2)–Re(1)–Cl(1)	80.82(7)	81.70

Figure S27. Selected molecular orbitals of the complexes **1A–4A** along with their percentage composition.

	**1A**	**2A**	**3A**	**4A**	**Composition**
**H-4**	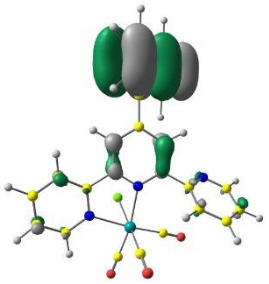	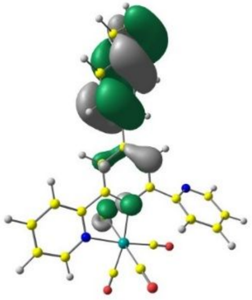	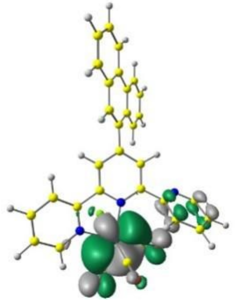	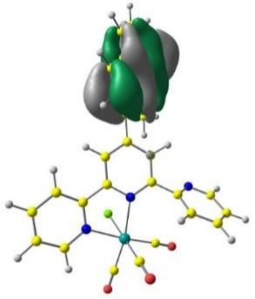	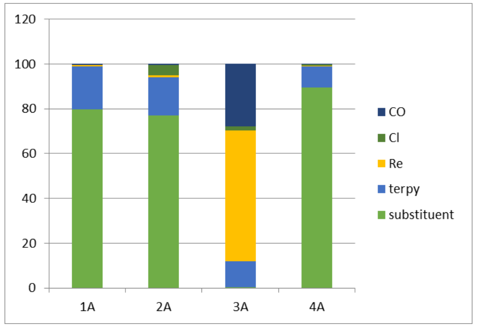
**H-3**	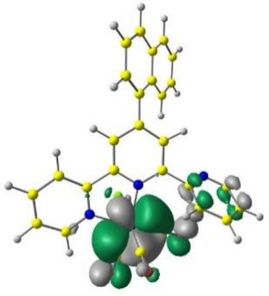	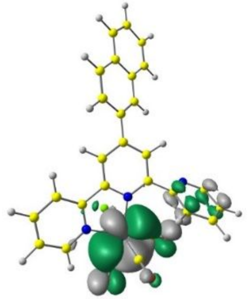	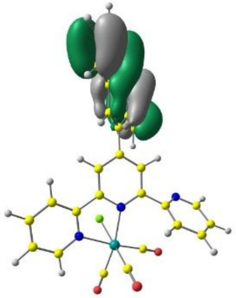	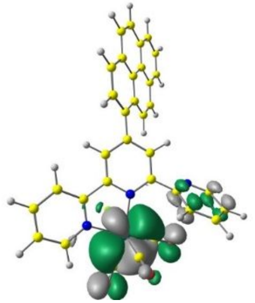	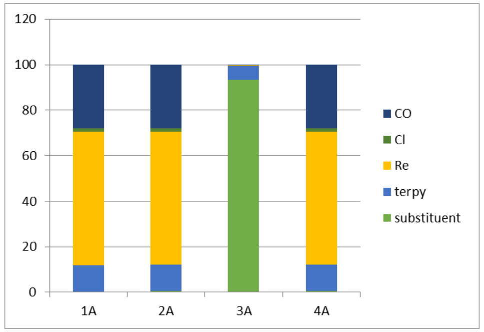
**H-2**	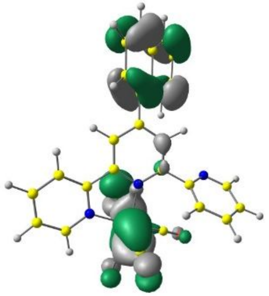	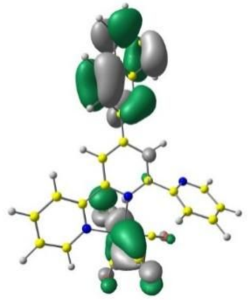	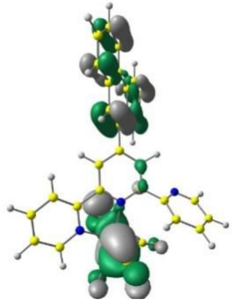	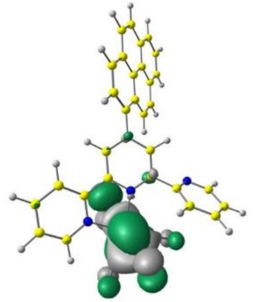	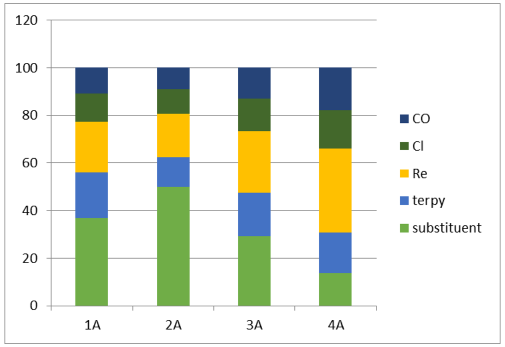

**H-1**	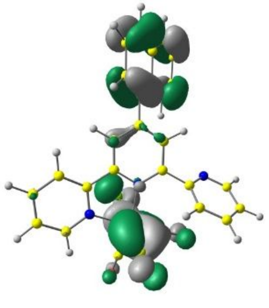	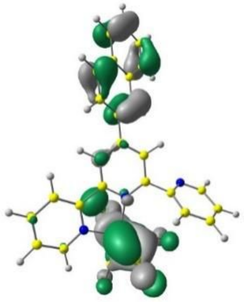	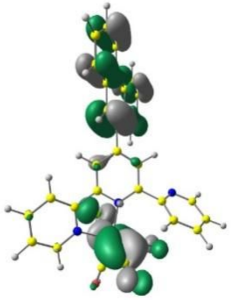	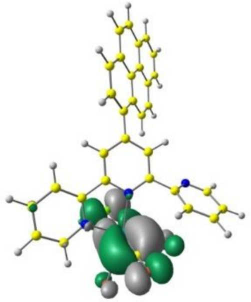	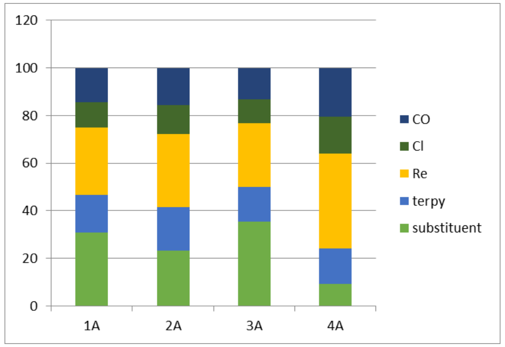120 100 80 60 40 20 0<br>1A 2A 3A 4A<br>CO<br>Cl<br>Re<br>terpy<br>substituent
**HOMO**	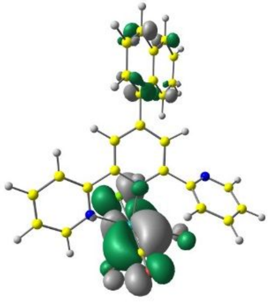	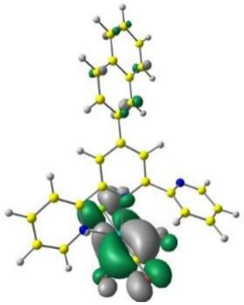	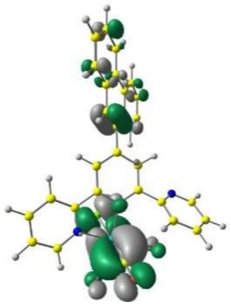	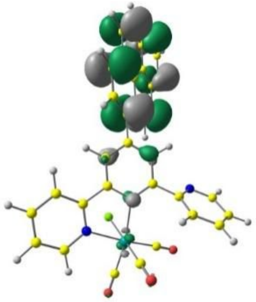	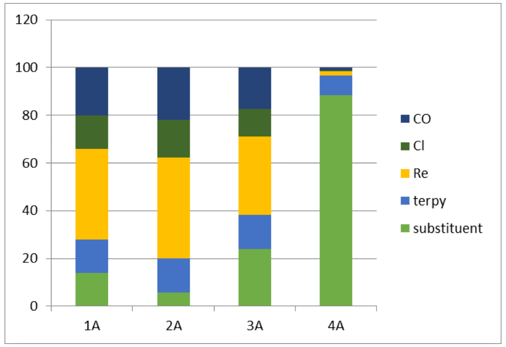120 100 80 60 40 20 0<br>1A 2A 3A 4A<br>CO<br>Cl<br>Re<br>terpy<br>substituent
**LUMO**	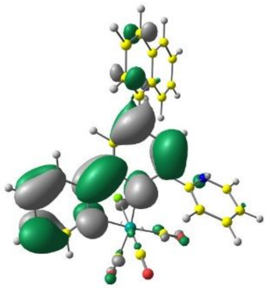	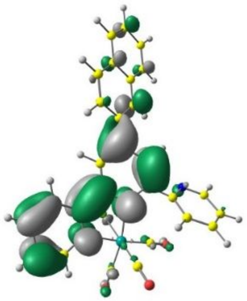	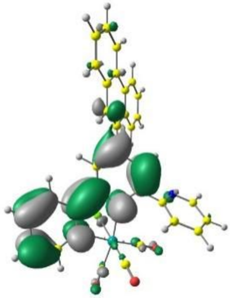	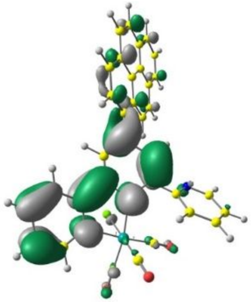	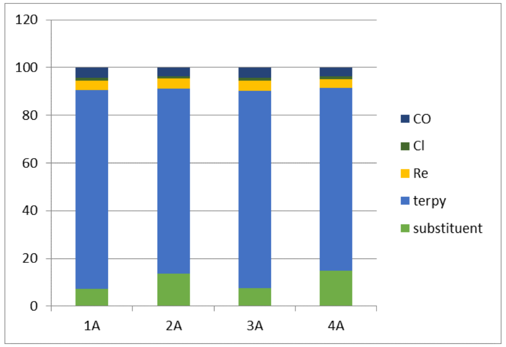120 100 80 60 40 20 0<br>1A 2A 3A 4A<br>CO<br>Cl<br>Re<br>terpy<br>substituent

**L+1**	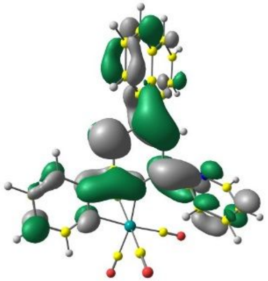	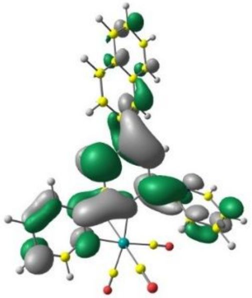	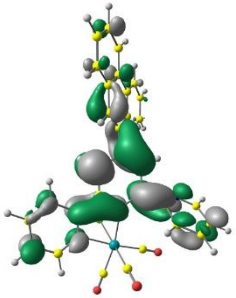	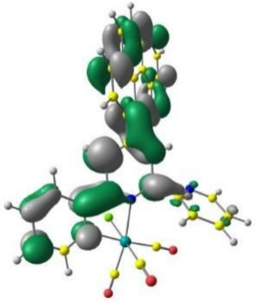	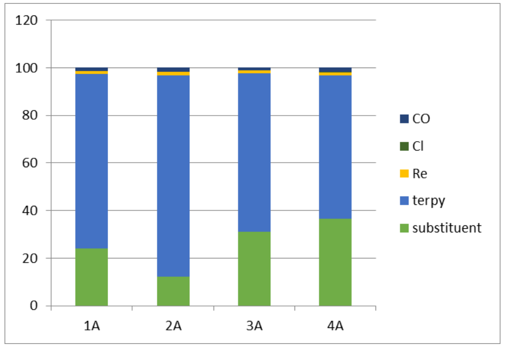120<br>100<br>80<br>60<br>40<br>20<br>0<br>1A<br>2A<br>3A<br>4A<br>CO<br>Cl<br>Re<br>terpy<br>substituent
**L+2**	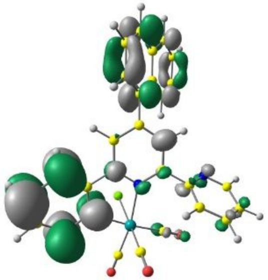	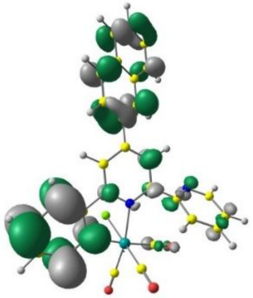	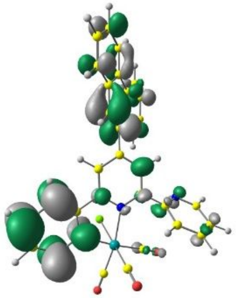	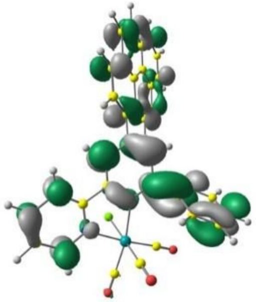	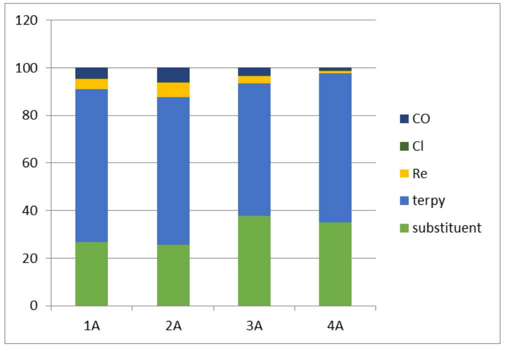120<br>100<br>80<br>60<br>40<br>20<br>0<br>1A<br>2A<br>3A<br>4A<br>CO<br>Cl<br>Re<br>terpy<br>substituent

Figure S28. Selected molecular orbitals of the complexes **1B–4B** along with their percentage composition.

	1B	2B	3B	4B	Composition
**H-4**	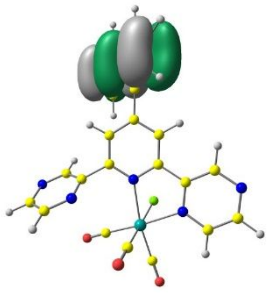	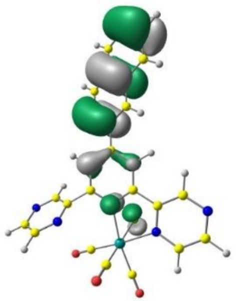	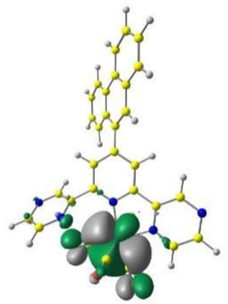	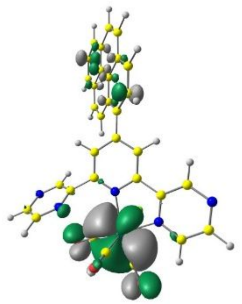	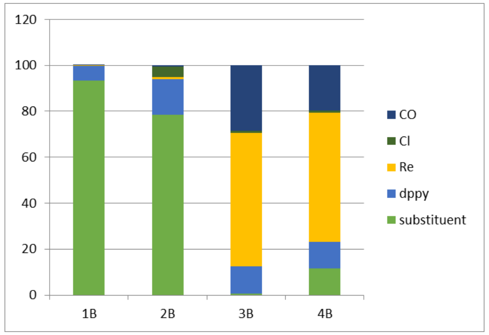
**H-3**	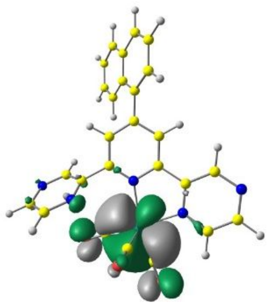	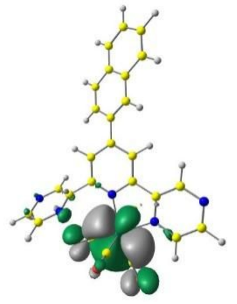	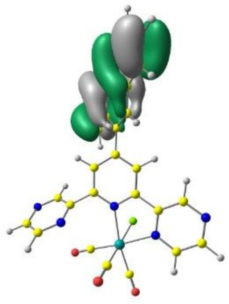	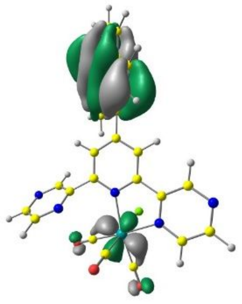	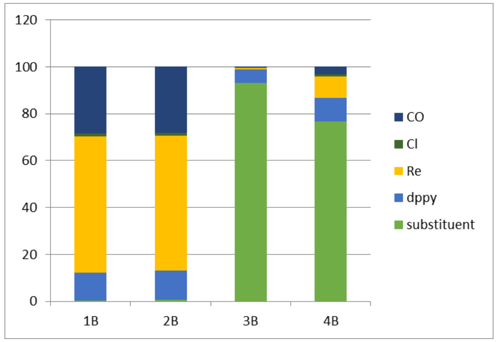
**H-2**	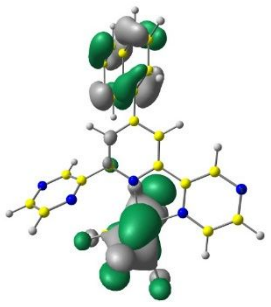	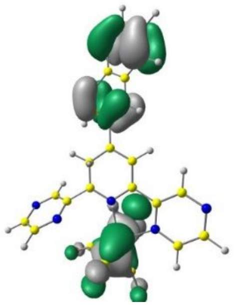	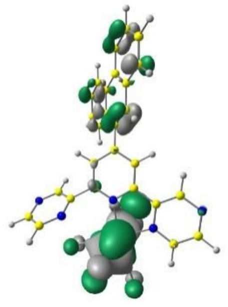	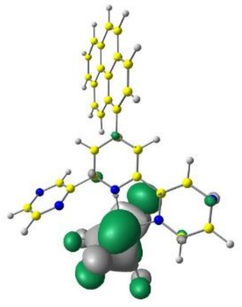	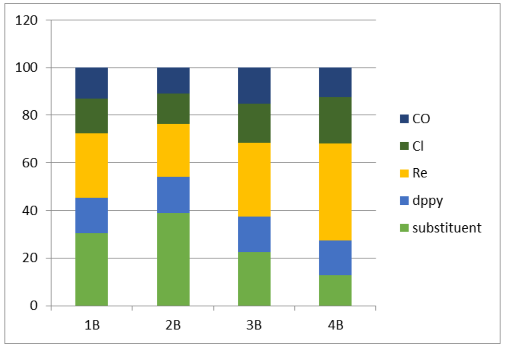

**H-1**	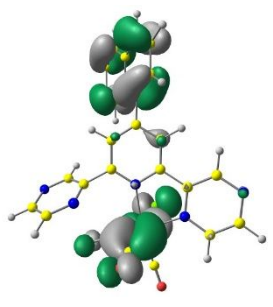	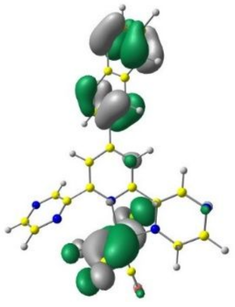	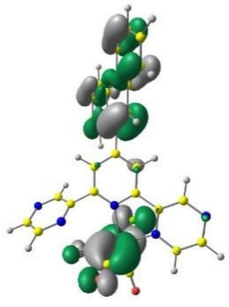	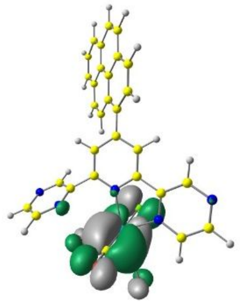	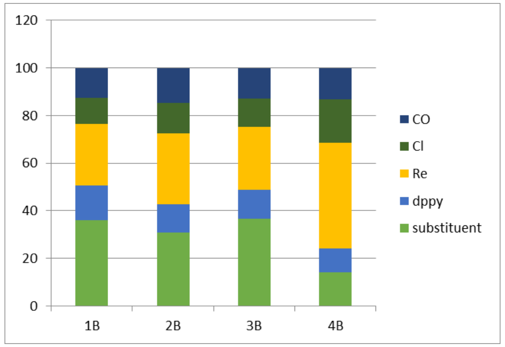120 100 80 60 40 20 0<br>1B 2B 3B 4B<br>CO<br>Cl<br>Re<br>dppy<br>substituent
**HOMO**	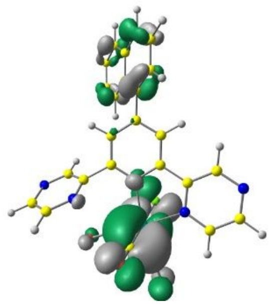	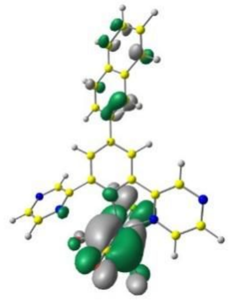	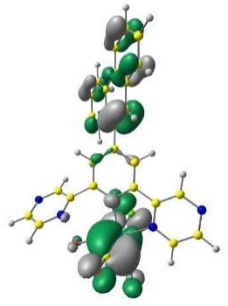	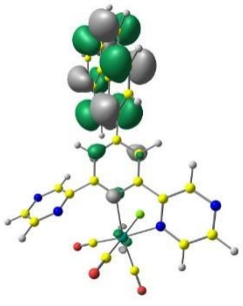	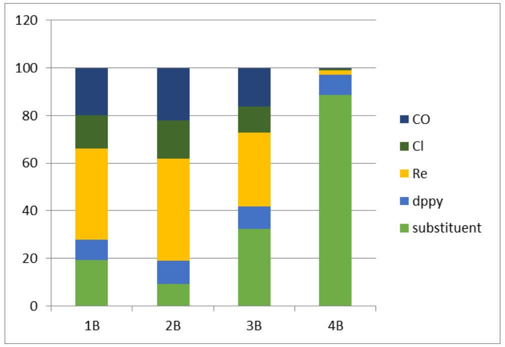120 100 80 60 40 20 0<br>1B 2B 3B 4B<br>CO<br>Cl<br>Re<br>dppy<br>substituent
**LUMO**	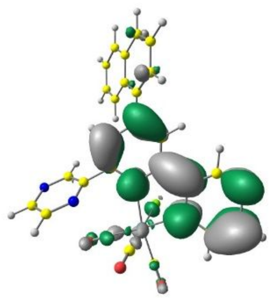	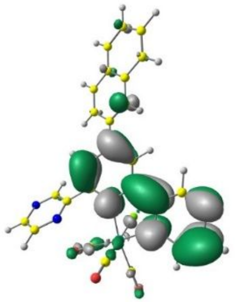	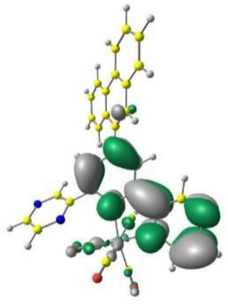	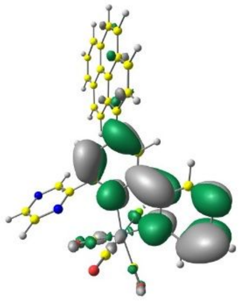	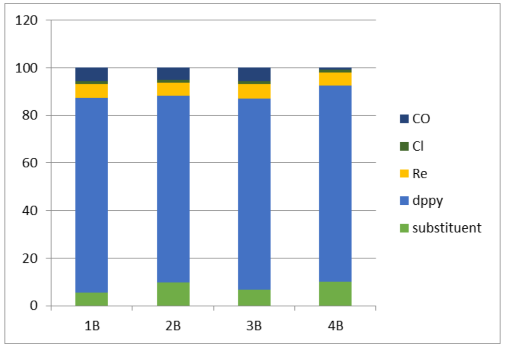120 100 80 60 40 20 0<br>1B 2B 3B 4B<br>CO<br>Cl<br>Re<br>dppy<br>substituent

**L+1**	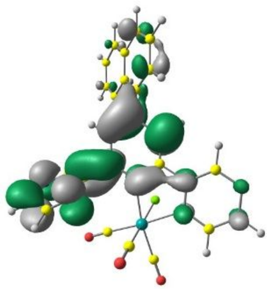	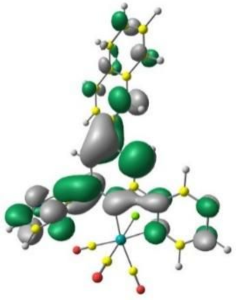	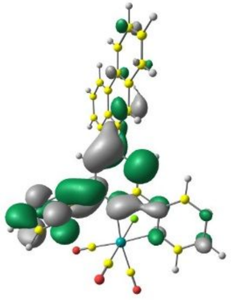	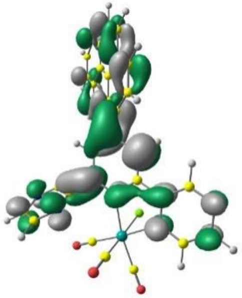	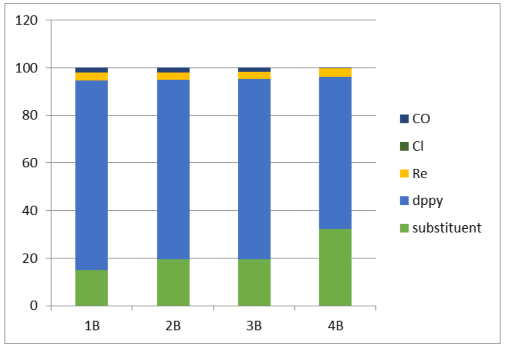120<br>100<br>80<br>60<br>40<br>20<br>0<br>1B<br>2B<br>3B<br>4B<br>CO<br>Cl<br>Re<br>dppy<br>substituent
**L+2**	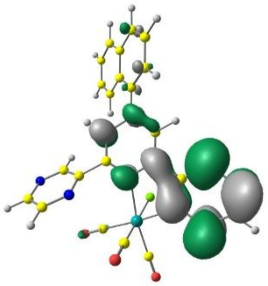	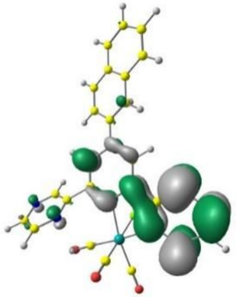	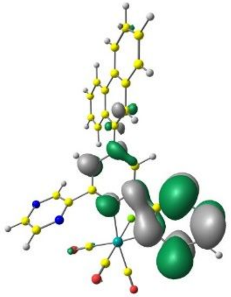	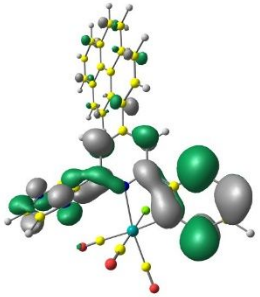	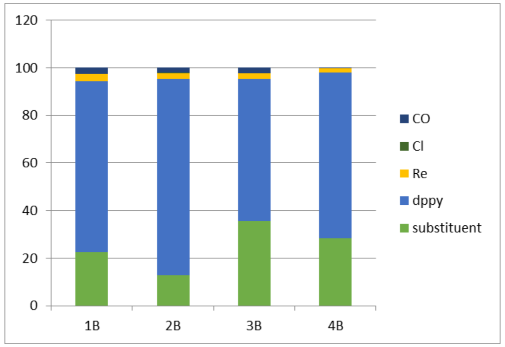120<br>100<br>80<br>60<br>40<br>20<br>0<br>1B<br>2B<br>3B<br>4B<br>CO<br>Cl<br>Re<br>dppy<br>substituent

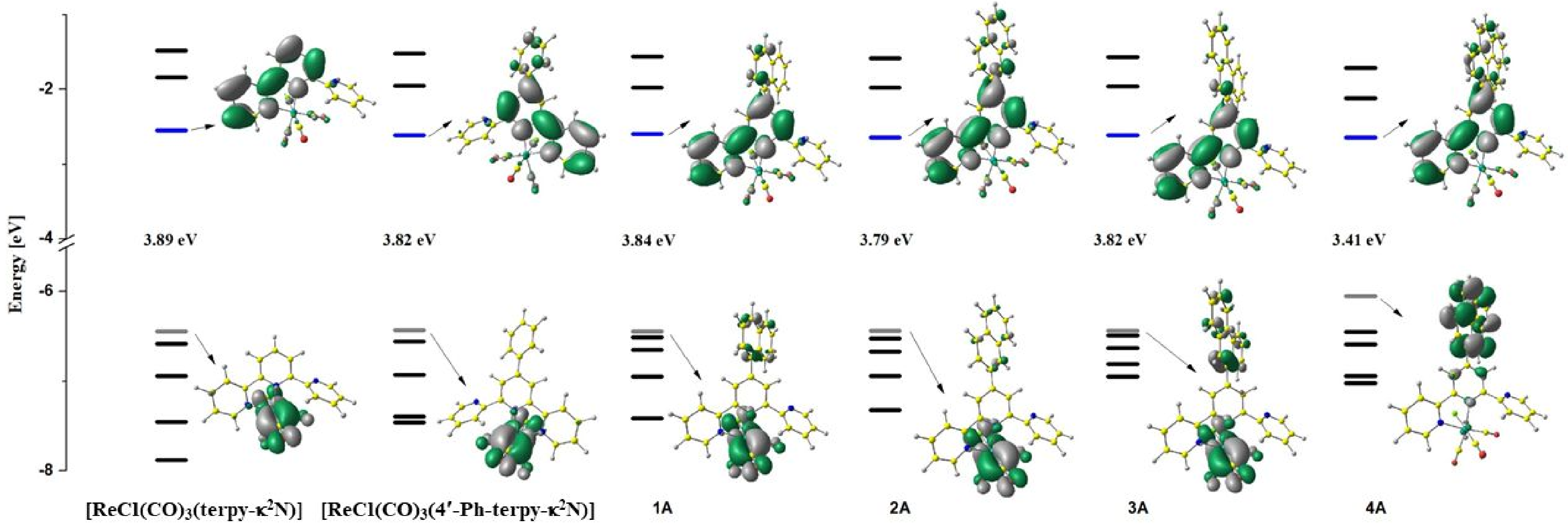

Figure S29. The partial molecular orbital energy level diagrams for **1A–4A** compared to [ReCl$(CO)_3$(terpy-κ^{2}N)] and [ReCl$(CO)_3$(4′-Ph-terpy-κ^{2}N)] [16-17], along with the plots of the frontier molecular orbitals of [ReCl$(CO)_3$(4′-Ar^n-terpy-κ^{2}N)]. The partial molecular orbital curves of [ReCl$(CO)_3$(terpy-κ^{2}N)] and [ReCl$(CO)_3$(4′-Ph-terpy-κ^{2}N)] were reproduced from ref [16-17]. Copyright John Wiley and Sons 2018 and Royal Society of Chemistry 2020.

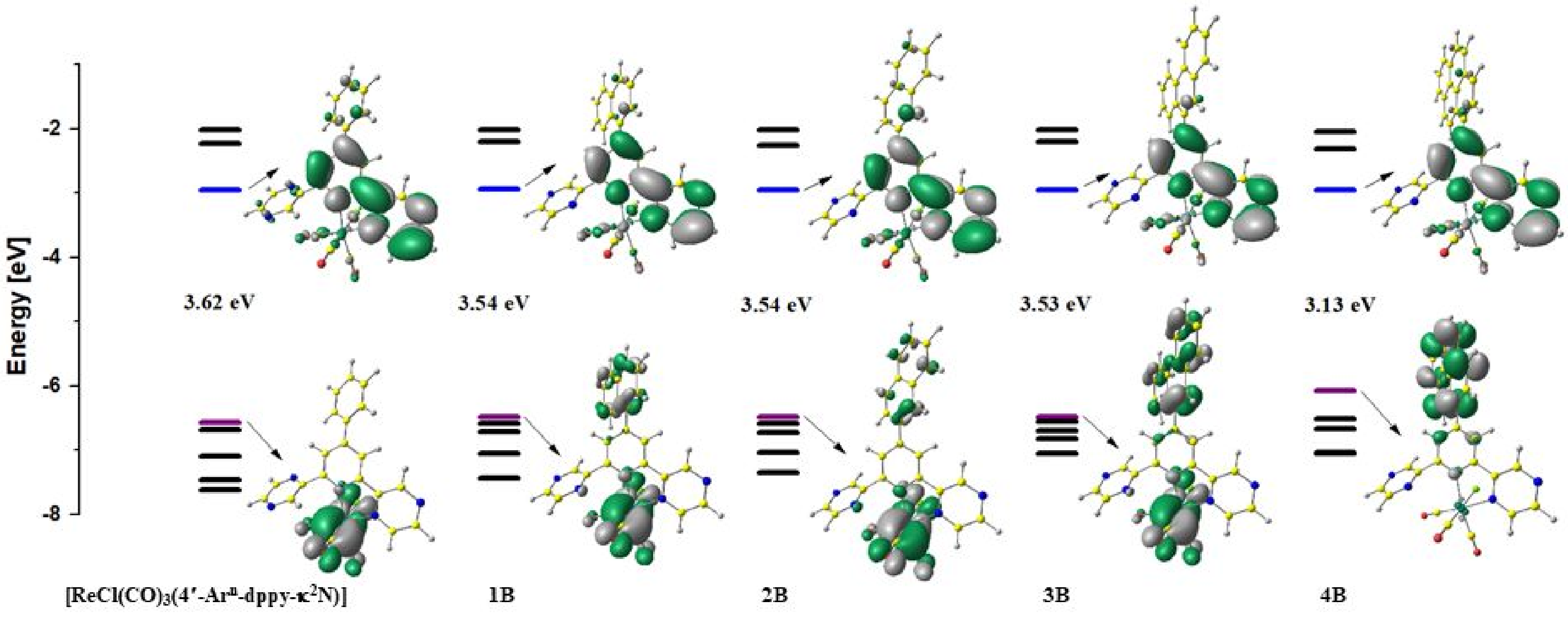

Figure S30. The partial molecular orbital energy-level diagrams for **1B–4B** compared to [ReCl$(CO)_3$(4-Ph-dppy-κ^{2}N)][4], along with the plots of the frontier molecular orbitals of [ReCl$(CO)_3$(4-Ar^n-dppy-κ^{2}N)]. The partial molecular orbital curves of [ReCl$(CO)_3$(4-Ph-dppy-κ^{2}N)] was reproduced from ref [16]. Copyright John Wiley and Sons 2018.

Table S8. Calculated ionization potentials and electron affinities (vertical and adiabatic), energy gap, as well as hole and electrons reorganization energies and extraction potentials (DFT/PBE1PBE/def2-TZVPD/def2-TZVP) for **1A–4A** and **1B–4B**.

Complex	IP(v) [eV]	IP(a) [eV]	EA(v) [eV]	EA(a) [eV]	λ_{hole} [eV]	$\lambda_{electron}$ [eV]	HEP [eV]	EEP [eV]	Energy gap (a) [eV]
**1A**	6.17	5.84	2.88	3.05	0.71	0.34	5.46	3.22	2.79
**2A**	6.16	5.83	2.92	3.08	0.71	0.32	5.45	3.24	2.75
**3A**	6.17	5.84	2.89	3.06	0.70	0.34	5.47	3.23	2.78
**4A**	5.77	5.70	2.91	3.07	0.16	0.31	5.62	3.22	2.63
**1B**	6.22	5.83	3.22	3.38	0.99	0.33	5.23	3.54	2.45
**2B**	6.21	5.82	3.22	3.37	1.01	0.31	5.20	3.53	2.45
**3B**	6.22	5.83	3.23	3.39	1.01	0.33	5.21	3.55	2.44
**4B**	5.81	5.74	3.23	3.38	0.16	0.31	5.65	3.54	2.36

$EEP = E^0(M^-) - E^-(M^-)$; $HEP = E^+(M^+) - E^0(M^+)$;

$\lambda_{electron} = EEP - EA_v$; $\lambda_{hole} = IP_v - HEP$

Electrochemistry

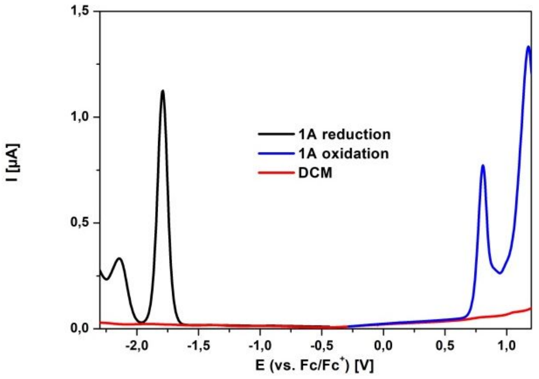

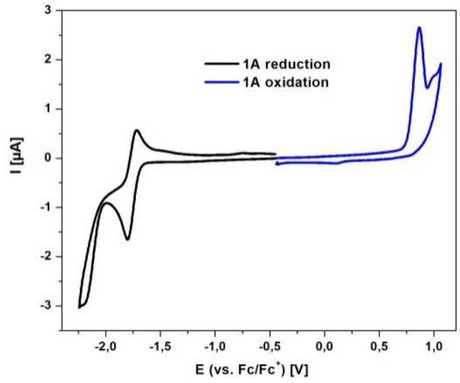

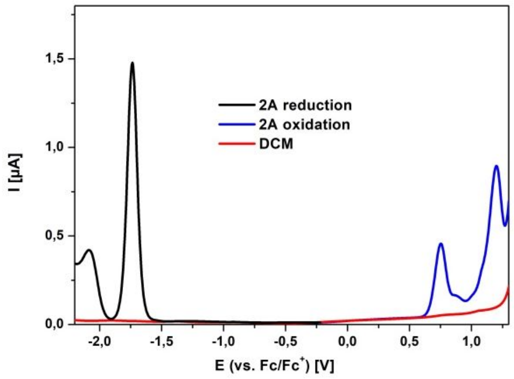

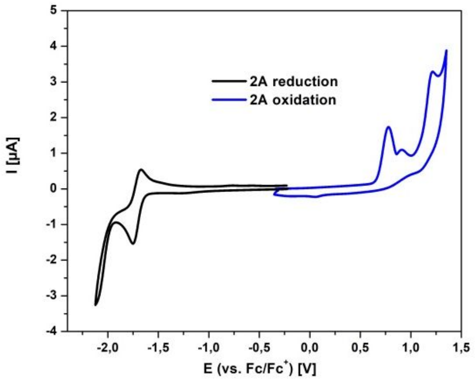

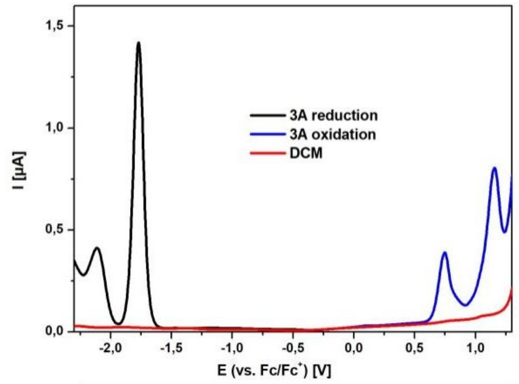

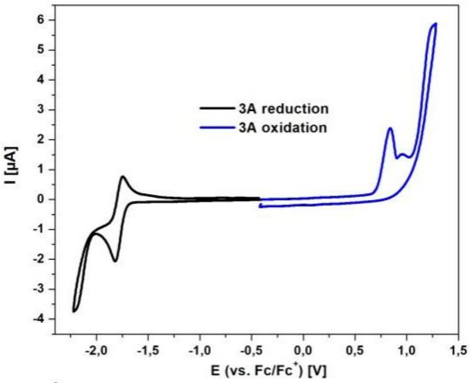

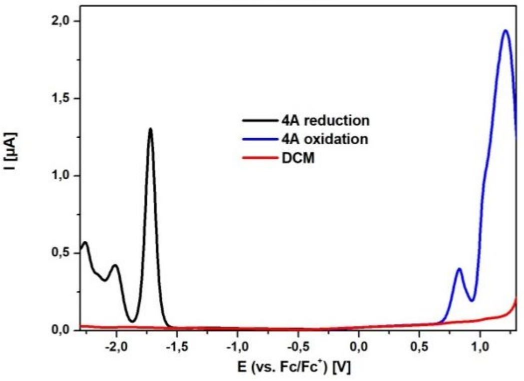

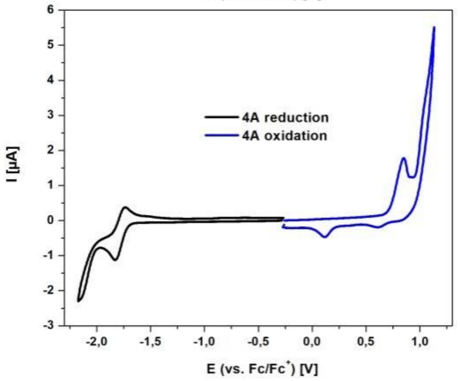

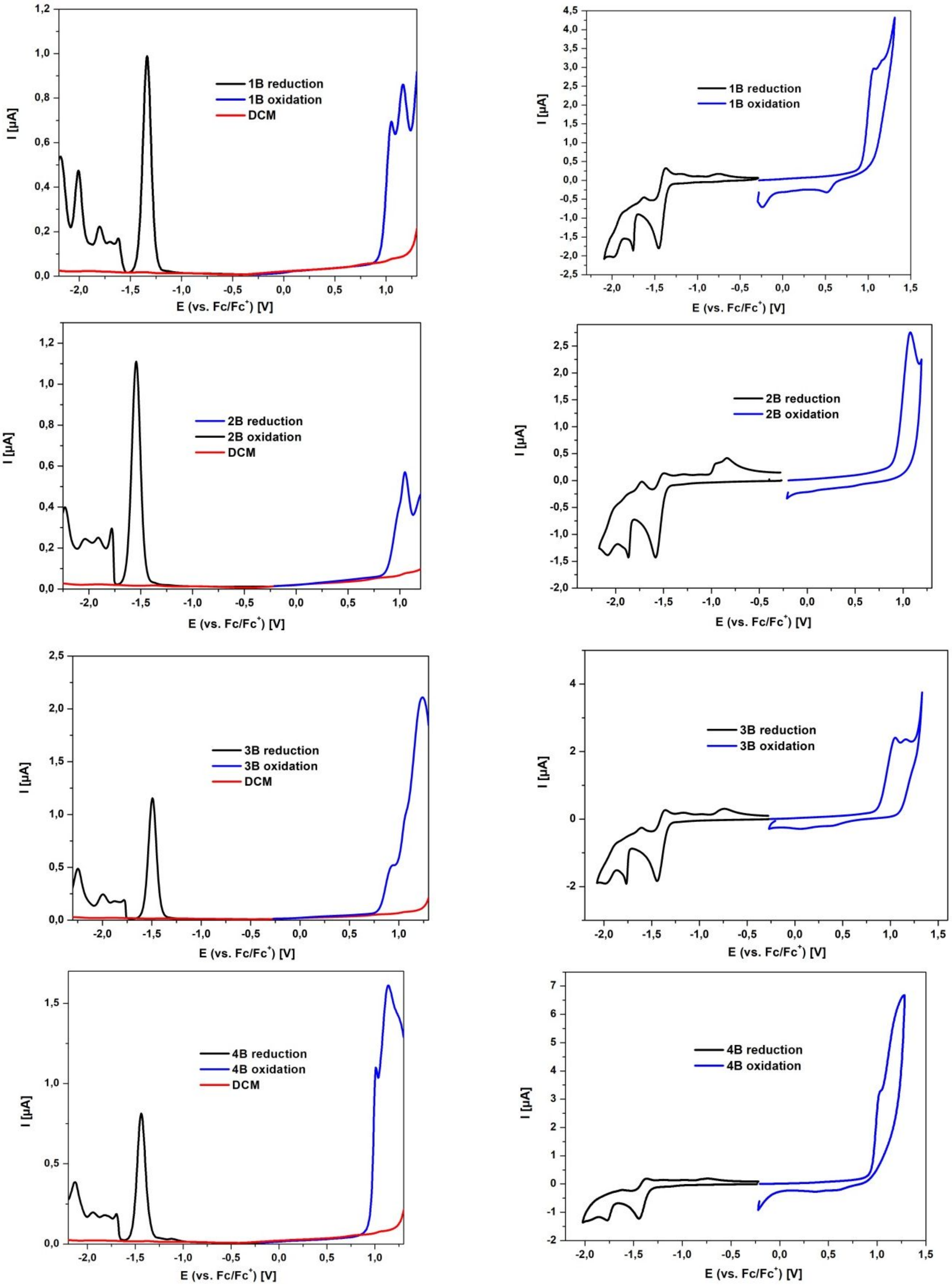

Figure S31. Cyclic voltammetry (CV, right side) and differential pulse voltammetry (DPV, left side) of **1A**–**4A** and **1B**–**4B**. Processes were recorded in dichloromethane with 0.1M Bu_4NPF_6 as the supporting electrolyte under argon atmosphere at scan rate 100 mV/s.

Table S9. Electrochemical properties of **1A–4A** and **1B–4B**.

Compound	E_{1red}^{onset} [V]	E_{2red}^{onset} [V]	E_{3red}^{onset} [V]	E_{1ox}^{onset} [V]	E_{2ox}^{onset} [V]	E_{3ox}^{onset} [V]	IP[a] (CV)	EA[b] (CV)	$E_{g(CV)}$[c] [eV]
**1A**	-1.67 (-1.69)[d]	-2.04 (-2.01)[d]	—	0.73 (0.71)[d]	0.95 (0.98)[d]	—	-5.83	-3.43	2.40
**2A**	-1.63 (-1.64)[d]	-1.96 (-1.96)[d]	—	0.67 (0.65)[d]	0.86 (0.84)[d]	1.02 (1.02)[d]	-5.77	-3.47	2.30
**3A**	-1.67 (-1.65)[d]	-2.03 (-2.00)[d]	—	0.66 (0.64)[d]	1.03 (1.02)[d]	—	-5.76	-3.43	2.33
**4A**	-1.65 (-1.65)[d]	-1.97 (-1.94)[d]	—	0.71 (0.71)[d]	0.97 (0.96)[d]	—	-5.81	-3.45	2.36
**1B**	-1.32 (-1.29)[d]	-1.70 (-1.66)[d]	-1.86 (-1.83)[d]	0.96 (0.94)[d]	1.10 (1.10)[d]	—	-6.06	-3.78	2.28
**2B**	-1.46 (-1.43)[d]	-1.75 (-1.76)[d]	-1.99 (-1.97)[d]	0.90 (0.87)[d]	—	—	-6.00	-3.64	2.36
**3B**	-1.35 (-1.38)[d]	-1.78 (-1.76)[d]	-1.90 (-1.93)[d]	0.83 (0.80)[d]	1.11 (1.09)[d]	—	-5.93	-3.75	2.18
**4B**	-1.29 (-1.31)[d]	-1.63 (-1.66)[d]	-1.87 (-1.87)[d]	0.92 (0.94)[d]	1.05 (1.04)[d]	—	-6.02	-3.81	2.21

[a] IP = -5.1 - E_{ox}; [b] EA = -5.1 – E_{red}; [c] $Eg_{(CV)} = E_{ox\ (onset)} - E_{red\ (onset)}$; [d]determined by DPV measurement

UV-Vis

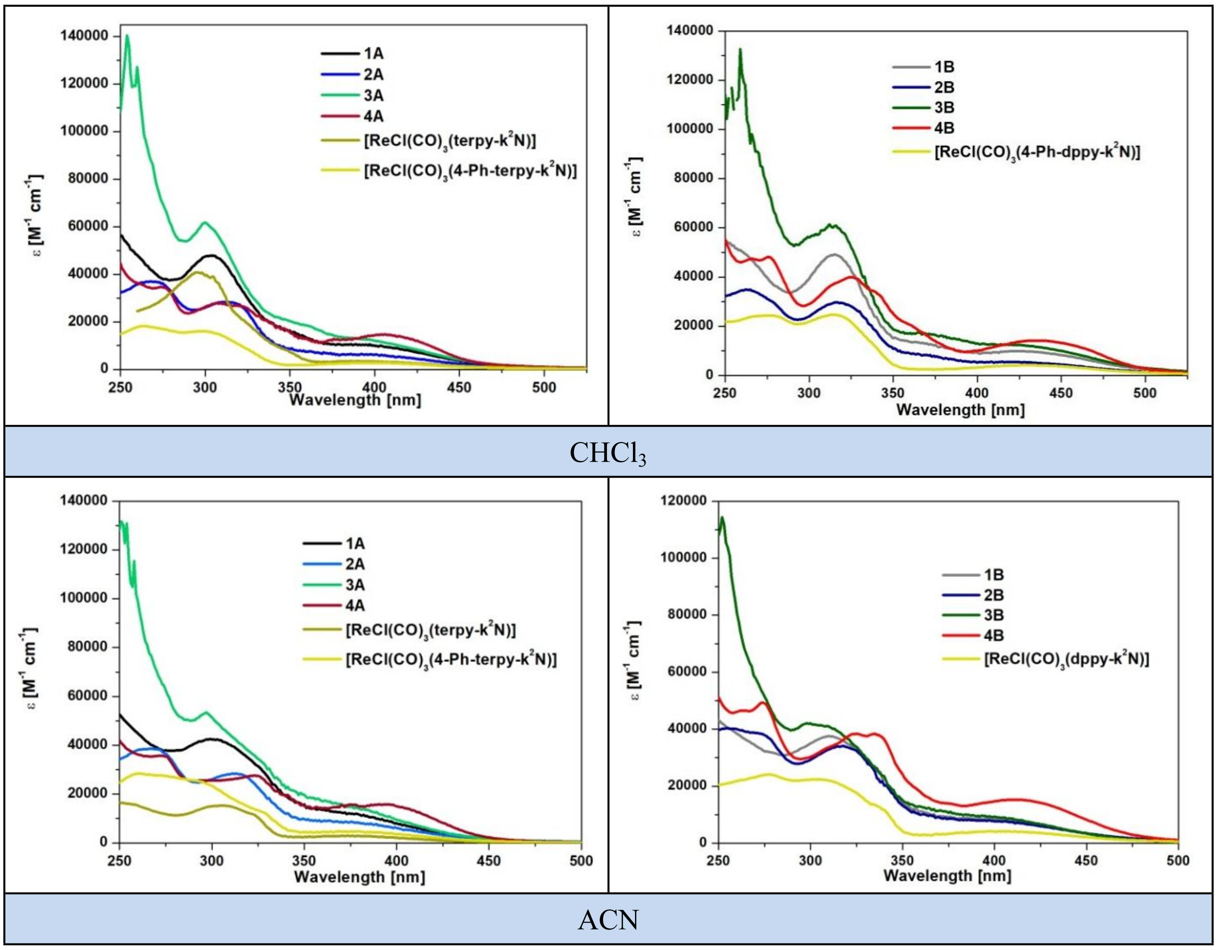

Figure S32. UV-Vis absorption spectra of **1A**–**4A** and **1B**–**4B** in $CHCl_3$ and CH_3CN, compared to UV-Vis spectra of [ReCl(CO)$_3$(terpy-κ^2N)], [ReCl(CO)$_3$(4′-Ph-terpy-κ^2N)] and [ReCl(CO)$_3$(4-Ph-dppy-κ^2N)][3-4]. UV-vis spectra of [ReCl(CO)$_3$(terpy-κ^2N)], [ReCl(CO)$_3$(4′-Ph-terpy-κ^2N)] and [ReCl(CO)$_3$(4-Ph-dppy-κ^2N)] were reproduced from ref. [16-17]. Copyright John Wiley and Sons 2018 and Royal Society of Chemistry 2020

Table S10. The absorption maxima and molar extinction coefficient for **1A–4A** and **1B–4B** in two solvents of different polarity ($CHCl_3$ and CH_3CN) and in thin film on glass substrate.

Compound	Medium	λ (nm) (ε ($M^{-1}cm^{-1}$))
**1A**	$CHCl_3$	399 (10332), 305 (48211)
	CH_3CN	380 (11372), 301 (42525)
	film	390, 303
**2A**	$CHCl_3$	404 (6175), 313 (28162), 269 (36639)
	CH_3CN	382 (8295), 314 (28357), 266 (38577)
	film	381, 310
**3A**	$CHCl_3$	401 (12655), 300 (62106), 260 (125296), 254 (137908)
	CH_3CN	376 (14735), 297 (53178), 257 (115284), 251 (130020)
	film	385, 302
**4A**	$CHCl_3$	409 (14360), 321 (26567), 306 (278380); 274 (34498)
	CH_3CN	395 (15862), 324 (27434), 272 (35784)
	film	429, 338, 304
**1B**	$CHCl_3$	436 (9691), 366 (13054), 315 (49268)
	CH_3CN	405 (8217), 311 (37480)
	film	412, 321
**2B**	$CHCl_3$	434 (13912), 373 (19581), 330 (6598), 320 (72629), 263 (84643)
	CH_3CN	402 (10739), 319 (49277), 271 (55284) 257 (57494)
	film	410, 331
**3B**	$CHCl_3$	434 (11811), 370 (17232), 315 (60433), 259 (129199)
	CH_3CN	402 (8373), 306 (41785), 252 (112008)
	film	400, 300
**4B**	$CHCl_3$	438 (14191), 340 (33674), 324 (40078), 276 (48168), 265 (47258)
	CH_3CN	413 (15235), 334 (38056), 324 (38224), 273 (49213), 262 (46483)
	film	444, 330

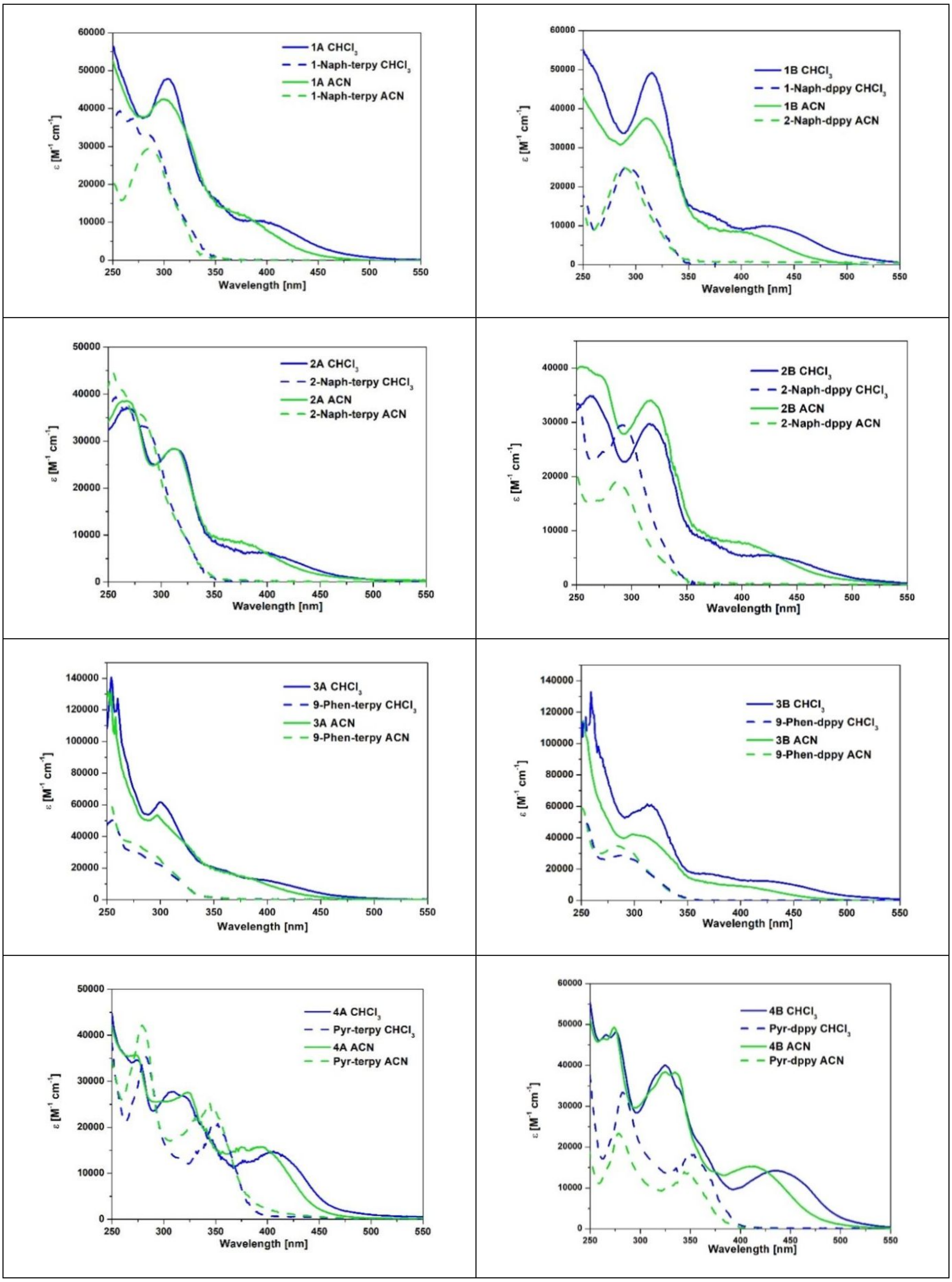

Figure S33. UV-Vis absorption spectra of **1A**–**4A** and **1B**–**4B** in comparison to the electronic spectra of the free ligands.

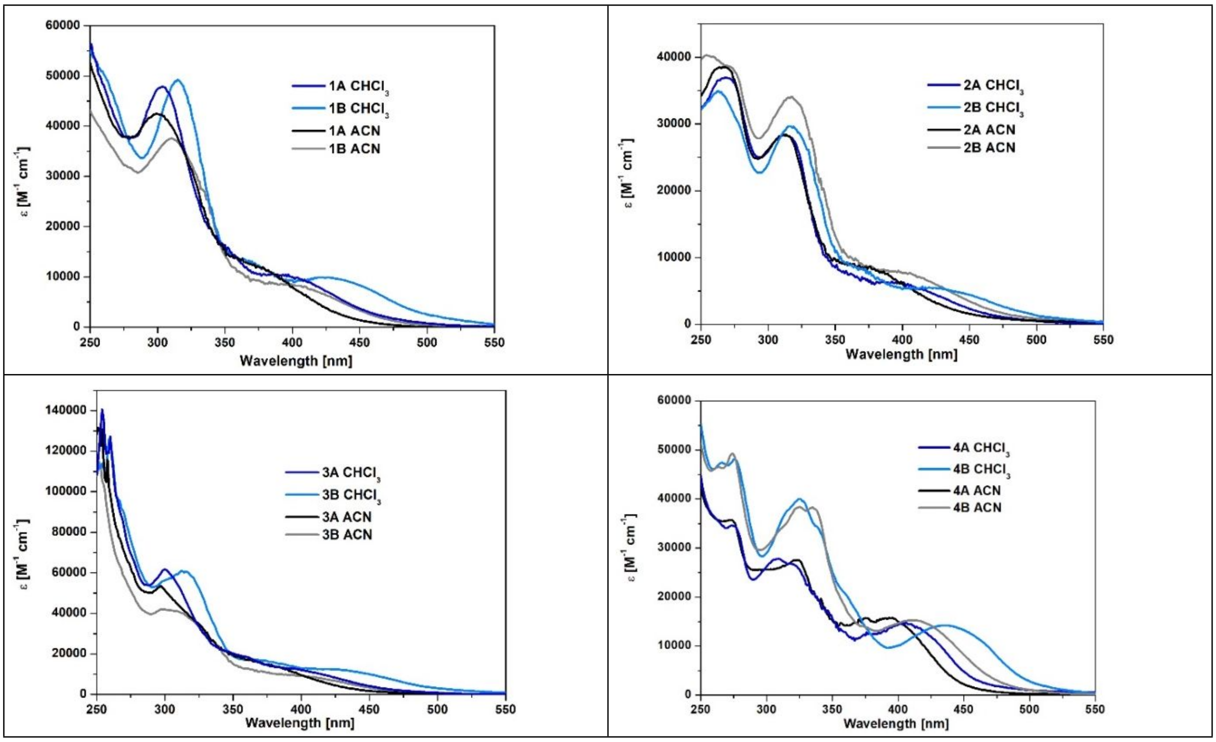

Figure S34. The impact of the triimine core and solvent polarity on the absorption properties of the investigated Re(I) complexes.

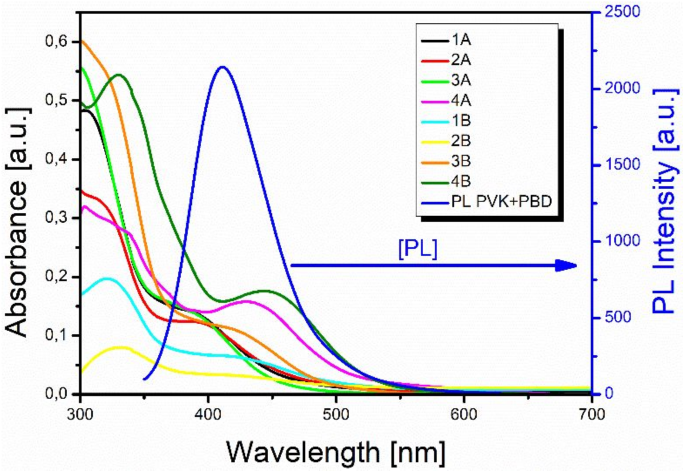

Figure S35. UV-Vis absorption spectra of **1A–4A** and **1B–4B** in film together with photoluminescence (PL) spectrum of PVK:PBD.

TD-DFT calculations

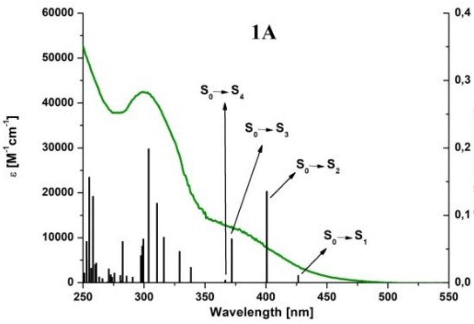

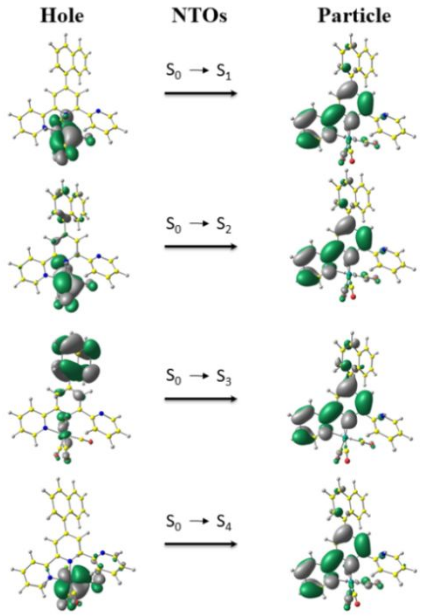

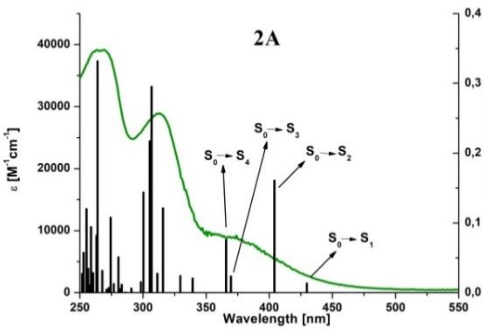

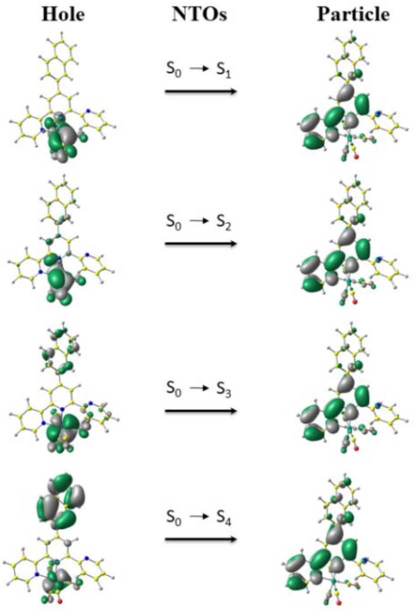

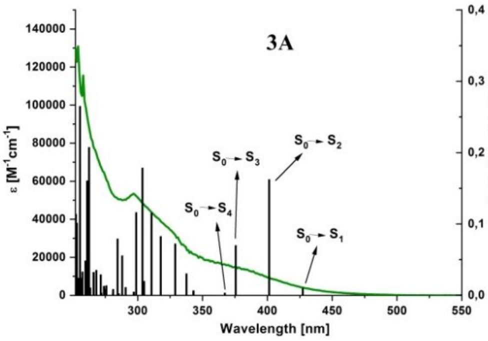
3A
ε [M-1cm-1]
Oscilator strength
Wavelength [nm]
S0 → S1
S0 → S2
S0 → S3
S0 → S4

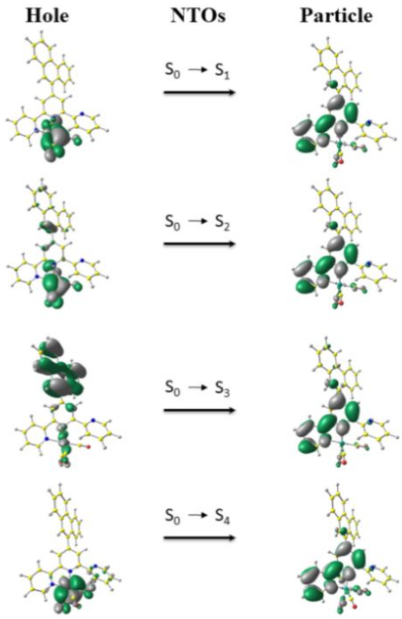
Hole
NTOs
Particle
S0 → S1
S0 → S2
S0 → S3
S0 → S4

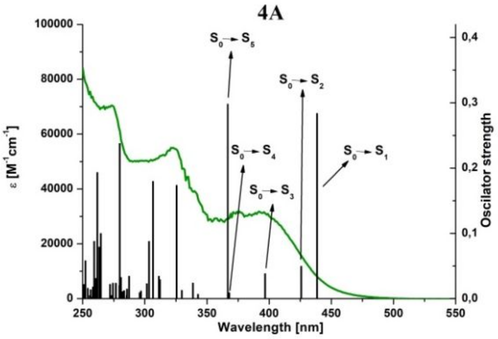
4A
ε [M-1cm-1]
Oscilator strength
Wavelength [nm]
S0 → S1
S0 → S2
S0 → S3
S0 → S4
S0 → S5

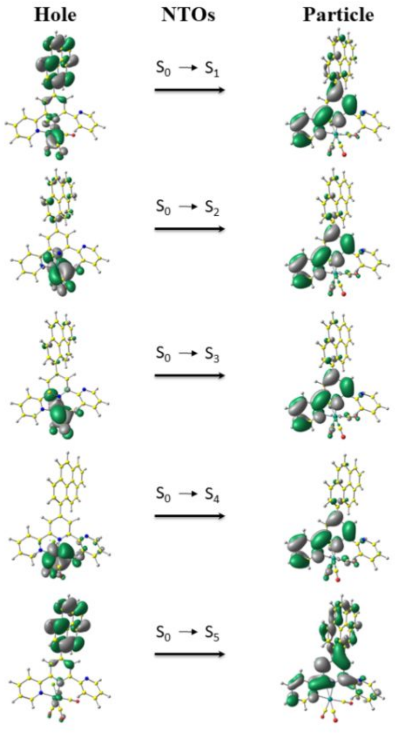
Hole
NTOs
Particle
S0 → S1
S0 → S2
S0 → S3
S0 → S4
S0 → S5

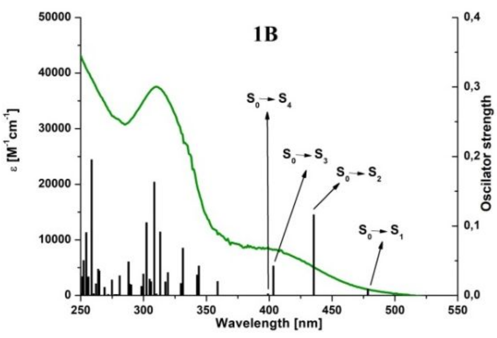
1B
ε [M⁻¹cm⁻¹]
Oscilator strength
Wavelength [nm]
S0→S4
S0→S3
S0→S2
S0→S1

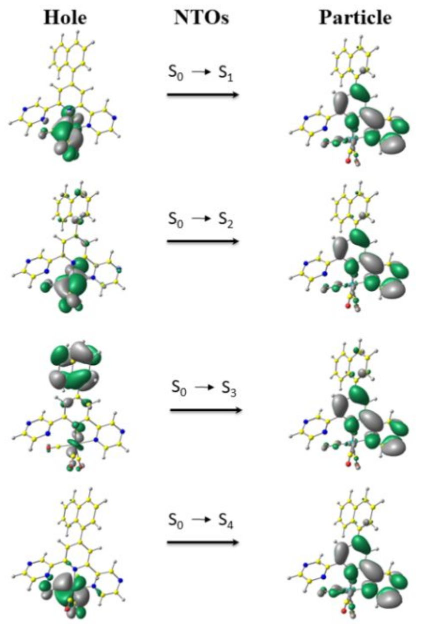
Hole
NTOs
Particle
S0 → S1
S0 → S2
S0 → S3
S0 → S4

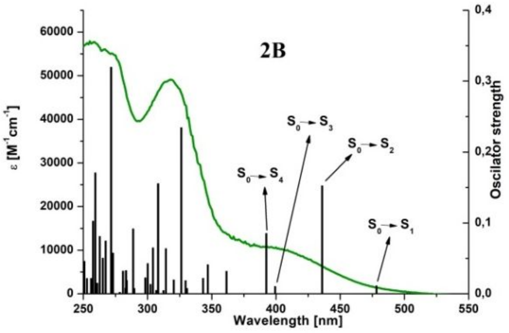
2B
ε [M⁻¹cm⁻¹]
Oscilator strength
Wavelength [nm]
S0→S4
S0→S3
S0→S2
S0→S1

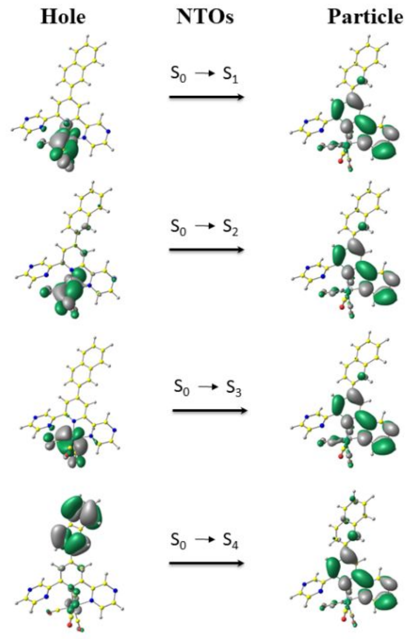
Hole
NTOs
Particle
S0 → S1
S0 → S2
S0 → S3
S0 → S4

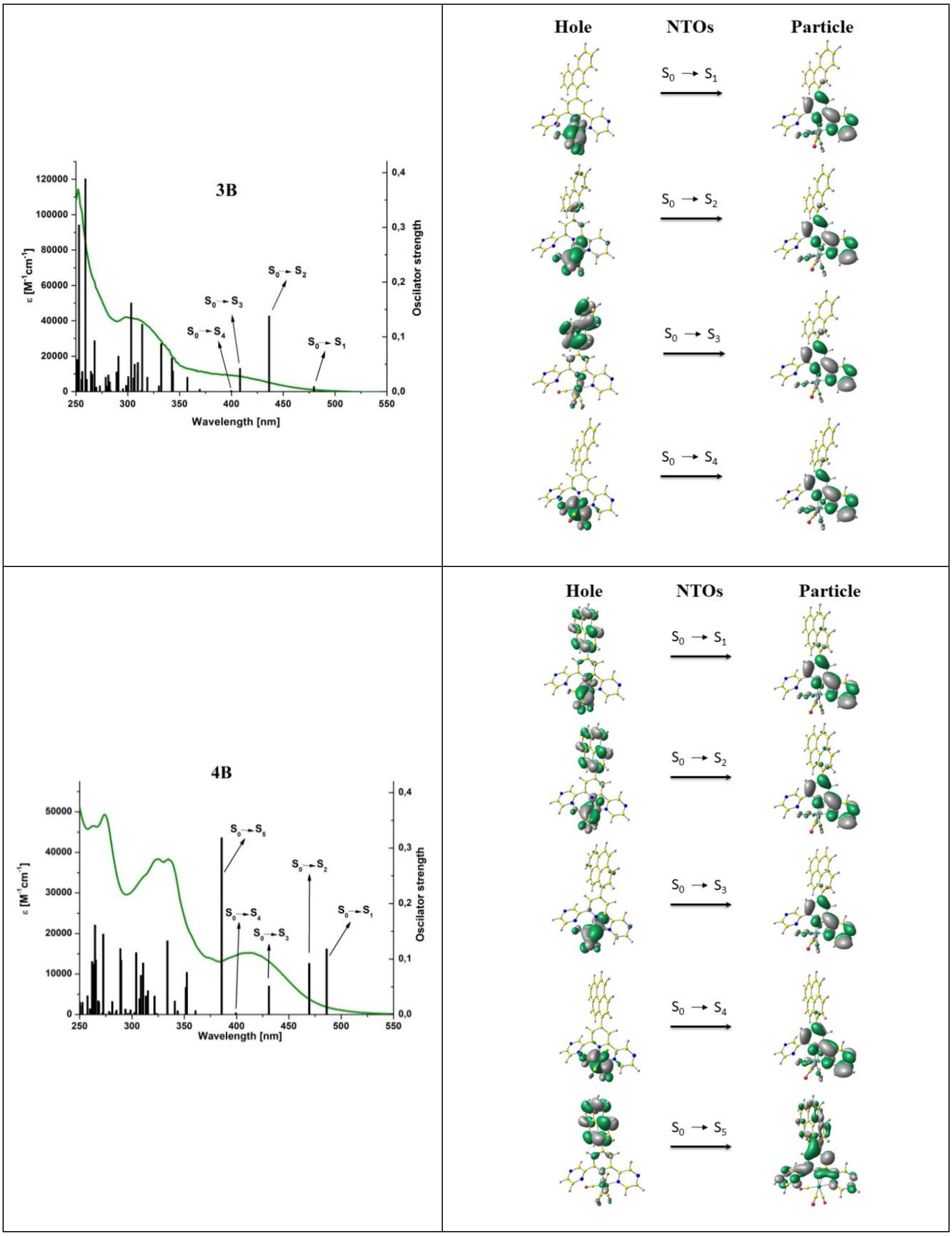

Figure S36. Electronic spectra of **1A–4A** to **1B–4B** in acetonitrile alongside with national transition orbitals calculated for vertical excitations, which were assigned to the lowest-energy absorption band.

Table S11. The energies and characters of the spin-allowed electronic transitions assigned to the lowest wavelength absorption bands of **1A–4A** to **1B–4B** computed at TD-DFT/PBE0/def2-TZVPD/def2-TZVP level with the use of the PCM model at polarities corresponding to CH_3CN.

Experimental absorption, λ [nm] (10^4ε [$M^{-1}cm^{-1}$])	Calculated transitions				
	Excitation	Character	E[eV]	λ [nm]	Oscillator strength
**1A**					
380 (11372)	$S_0 \rightarrow S_1$	MLCT	2.90	426.4	*0.0111*
	$S_0 \rightarrow S_2$	MLCT	3.09	400.6	*0.1356*
	$S_0 \rightarrow S_3$	IL, MLCT	3.33	371.9	*0.0653*
	$S_0 \rightarrow S_4$	MLCT	3.38	366.6	*0.0037*
**2A**					
373 (8991)	$S_0 \rightarrow S_1$	MLCT	2.88	429.7	*0.0142*
	$S_0 \rightarrow S_2$	MLCT	3.07	404.0	*0.1616*
	$S_0 \rightarrow S_3$	MLCT	3.35	369.6	*0.0242*
	$S_0 \rightarrow S_4$	IL, MLCT	3.38	365.8	*0.0763*
**3A**					
376 (14735)	$S_0 \rightarrow S_1$	MLCT	2.90	427.3	*0.0101*
	$S_0 \rightarrow S_2$	MLCT	3.09	401.3	*0.1624*
	$S_0 \rightarrow S_3$	IL, MLCT	3.30	375.6	*0.0700*
	$S_0 \rightarrow S_4$	MLCT	3.38	367.3	*0.0033*
**4A**					
391 (31586)	$S_0 \rightarrow S_1$	ILCT/IL/MLCT	2.83	438.5	*0.2840*
	$S_0 \rightarrow S_2$	MLCT	2.91	425.7	*0.0497*
	$S_0 \rightarrow S_3$	MLCT	3.13	396.6	*0.0383*
	$S_0 \rightarrow S_4$	MLCT	3.37	367.9	*0.0093*
	$S_0 \rightarrow S_5$	ILCT/IL	3.38	366.6	*0.2982*
**1B**					
405 (8217)	$S_0 \rightarrow S_1$	MLCT	2.59	478.4	*0.0093*
	$S_0 \rightarrow S_2$	MLCT	2.85	435.4	*0.1162*
	$S_0 \rightarrow S_3$	IL, MLCT	3.07	403.2	*0.0425*
	$S_0 \rightarrow S_4$	MLCT	3.11	399.0	*0.0023*
**2B**					
402 (10739)	$S_0 \rightarrow S_1$	MLCT	2.59	478.2	*0.0114*
	$S_0 \rightarrow S_2$	MLCT	2.84	435.9	*0.1526*
	$S_0 \rightarrow S_3$	MLCT	3.10	399.3	*0.0104*
	$S_0 \rightarrow S_4$	IL, MLCT	3.16	392.3	*0.0853*
**3B**					
402 (8373)	$S_0 \rightarrow S_1$	MLCT	2.58	479.6	*0.0093*
	$S_0 \rightarrow S_2$	MLCT	2.84	436.3	*0.1384*
	$S_0 \rightarrow S_3$	IL, MLCT	3.03	408.3	*0.0426*
	$S_0 \rightarrow S_4$	MLCT	3.10	399.8	*0.0022*

4B					
413 (15235)	$S_0 \rightarrow S_1$	ILCT/IL/MLCT	2.55	486.1	*0.1188*
	$S_0 \rightarrow S_2$	ILCT/IL/MLCT	2.64	469.4	*0.0924*
	$S_0 \rightarrow S_3$	MLCT	2.87	430.9	*0.0517*
	$S_0 \rightarrow S_4$	MLCT	3.11	399.2	*0.0033*
	$S_0 \rightarrow S_5$	ILCT/IL	3.21	385.6	*0.3187*

Luminescence studies

<table>
<tr><th>Medium</th><th>Emission and Excitation</th><th>Lifetime</th></tr>
<tr><th colspan="3">1A</th></tr>
<tr><td>CH_3CN</td><td>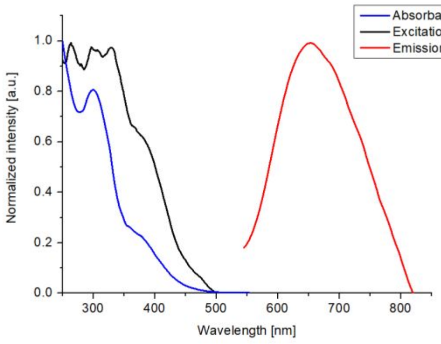
</td><td>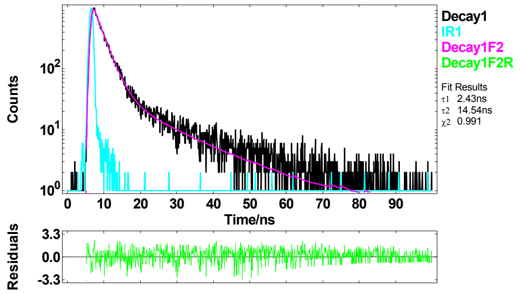

Fit results:
τ_1 2.43 ns ±0.02 ns 82.81%
τ_2 14.54 ns ±0.57 ns 17.19%</td></tr>
<tr><td>$CHCl_3$</td><td>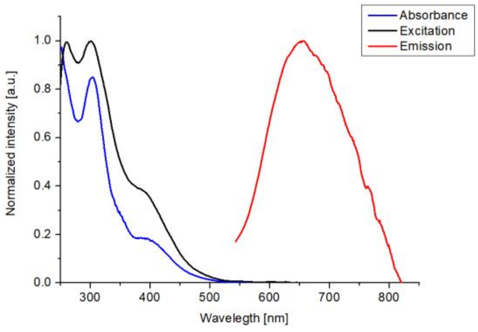
</td><td>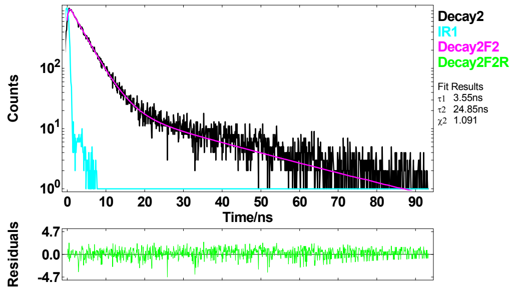

Fit results:
τ_1 3.55 ns ±0.03 ns 86.12%
τ_2 24.85 ns ±1.78 ns 13.88%</td></tr>
<tr><td>Solid</td><td>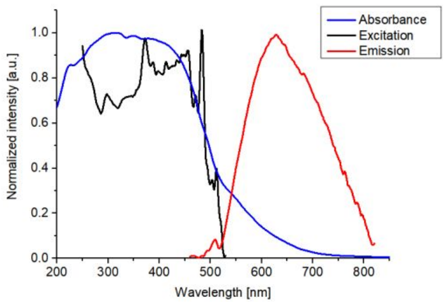
</td><td>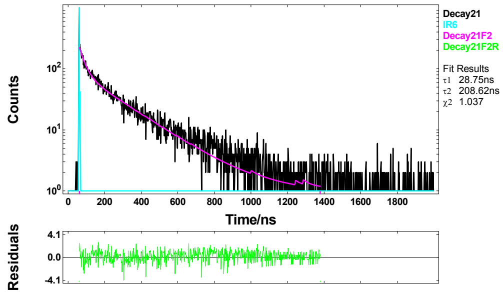

Fit results:
τ_1 28.75 ns ±2.27 ns 25.45%
τ_2 208.62 ns ±5.51 ns 74.55%</td></tr>
</table>

<table>
<tr>
<td>BuCN (77K)</td>
<td>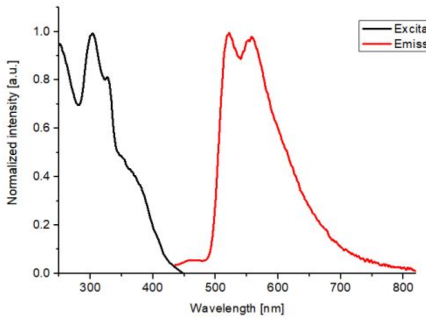
</td>
<td>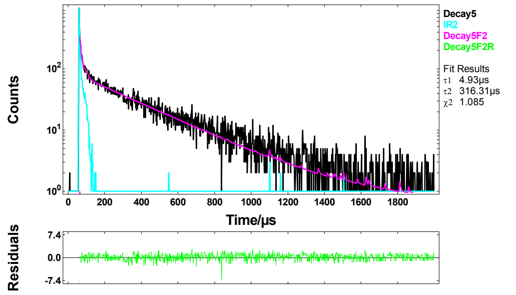

Fit results:
τ_1 4.93 μs ±0.69 μs 21.55%
τ_2 316.31 μs ±5.14 μs 78.45%</td>
</tr>
<tr>
<td colspan="3">2A</td>
</tr>
<tr>
<td>CH_3CN</td>
<td>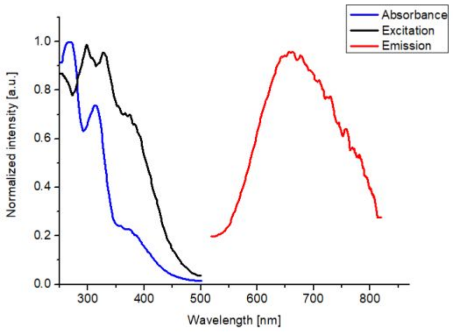
</td>
<td>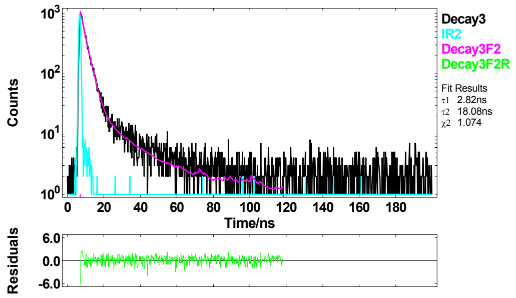

Fit results:
τ_1 2.82 ns ±0.04 ns 92.03%
τ_2 18.08 ns ±1.41 ns 7.97%</td>
</tr>
<tr>
<td>$CHCl_3$</td>
<td>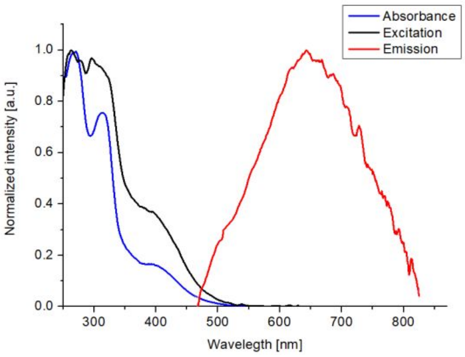
</td>
<td>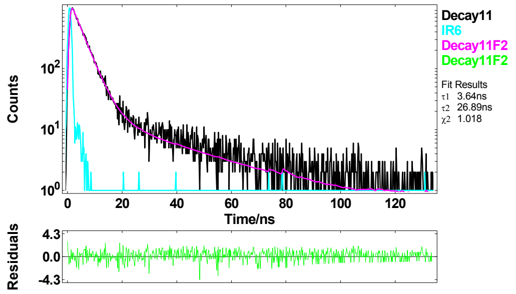

Fit results:
τ_1 3.64 ns ±0.04 ns 89.28%
τ_2 26.89 ns ±2.17 ns 10.72%</td>
</tr>
</table>

<table>
<tr><td>Solid</td><td>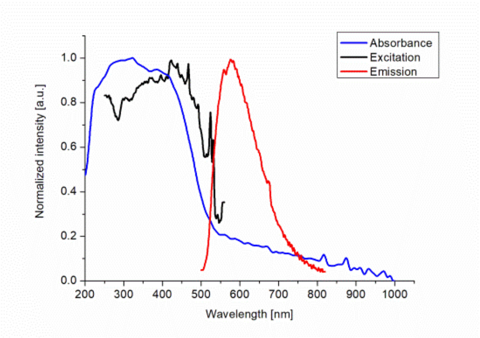
</td><td>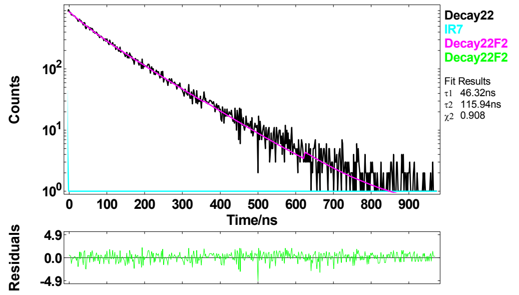

Fit results:
ττ_1 46.32 ns ±6.55 ns 19.72%
τ_2 115.94 ns ±2.64 ns 80.28%</td></tr>
<tr><td>BuCN (77K)</td><td>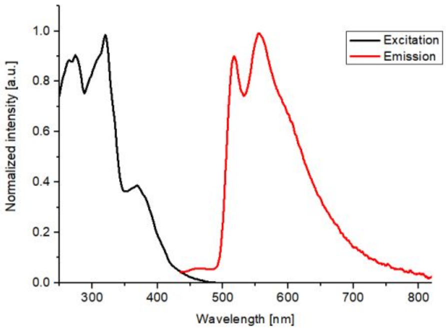
</td><td>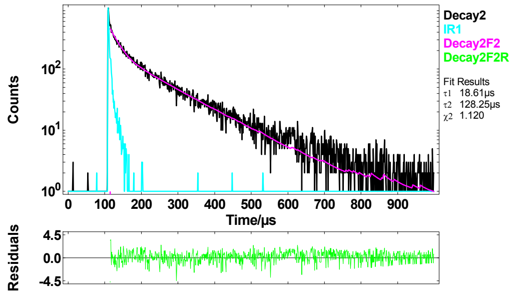

Fit results:
τ_1 18.61 μs ±0.97 μs 29.81%
τ_2 128.25 μs ±1.89 μs 70.19%</td></tr>
<tr><td colspan="3">3A</td></tr>
<tr><td>CH_3CN</td><td>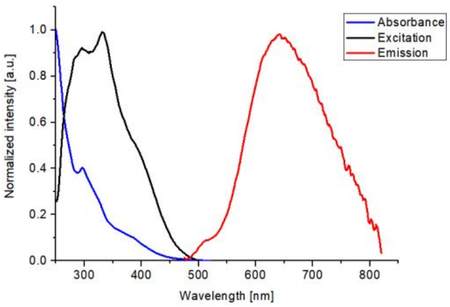
</td><td>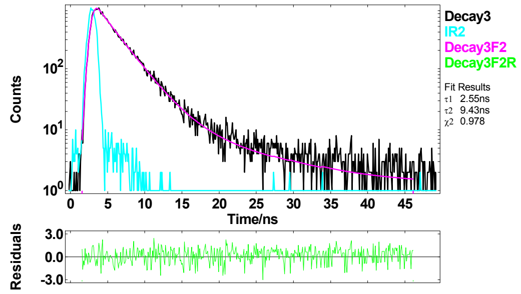

Fit results:
τ_1 2.55 ns ±0.04 ns 92.56%
τ_2 9.43 ns ±2.03 ns 7.44%</td></tr>
</table>

<table>
<tr><td>CHCl$_3$</td><td>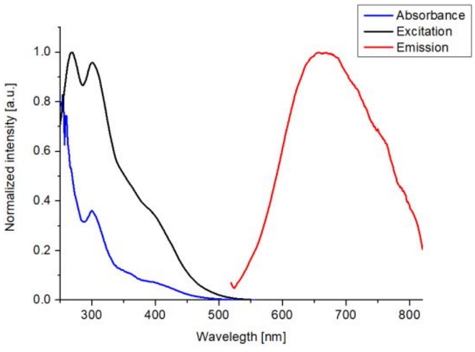
</td><td>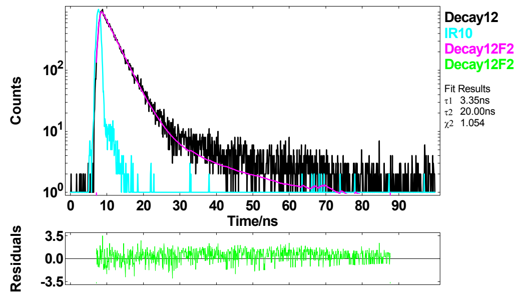

Fit results:
τ_1 3.35 ns ±0.03 ns 94.69%
τ_2 20.00 ns ±3.68 ns 5.31%</td></tr>
<tr><td>Solid</td><td>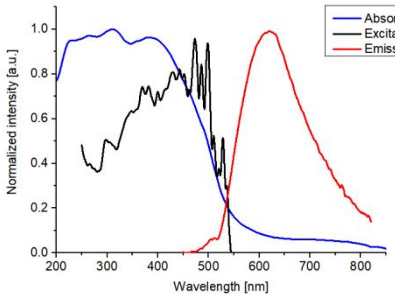
</td><td>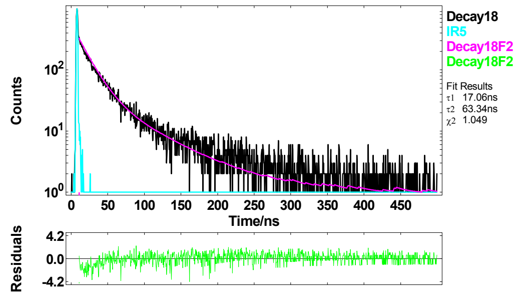

Fit results:
τ_1 17.06 ns ±0.54 ns 70.13%
τ_2 63.34 ns ±3.53 ns 29.87%</td></tr>
<tr><td>BuCN (77K)</td><td>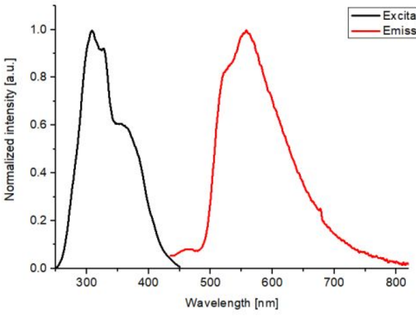
</td><td>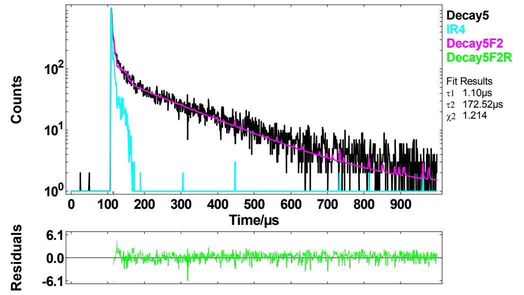

Fit results:
τ_1 1.10 μs ±0.38 μs 39.61%
τ_2 172.52 μs ±3.58 μs 60.39%</td></tr>
</table>

<table>
<tr><th colspan="3">4A</th></tr>
<tr><td rowspan="1">CH_3CN</td><td>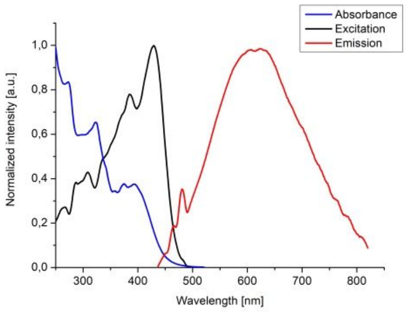
</td><td>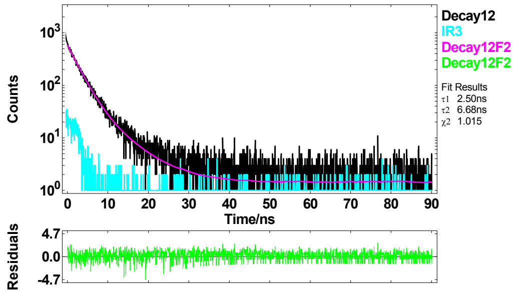

Fit results:
τ_1 2.50 ns ±0.06 ns 82.36%
τ_2 6.68 ns ±0.39 ns 17.64%</td></tr>
<tr><td>$CHCl_3$</td><td>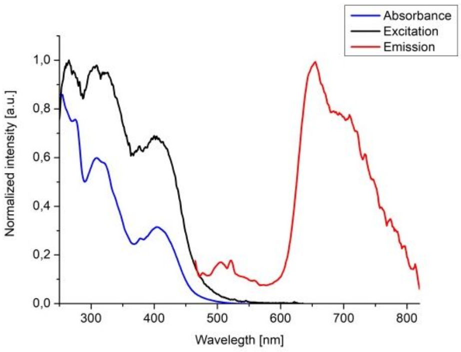
</td><td>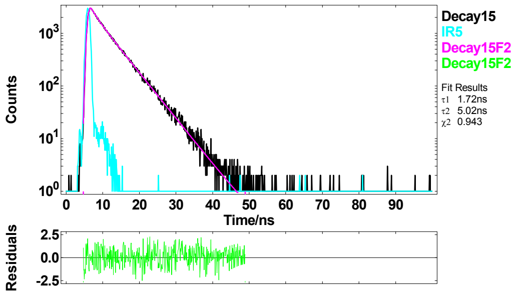

Fit results:
τ_1 1.72 ns ±0.16 ns 11.06%
τ_2 5.02 ns ±0.04 ns 88.94%
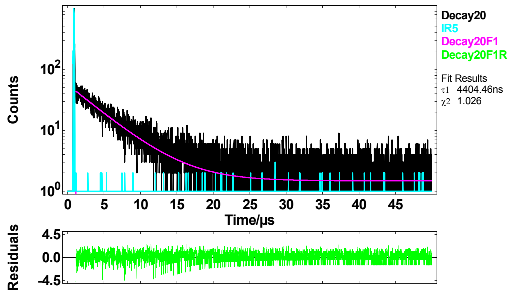

Fit results:
τ_1 4404.46 ns ±53.65 ns 100%</td></tr>
</table>

**BuCN (77K)**	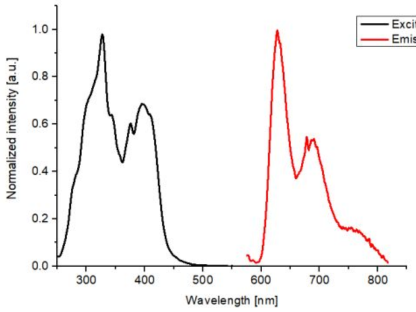	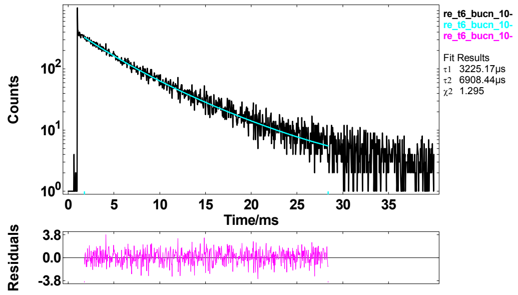<br>Fit results:<br>τ_1 3225.17 μs ±739.60 μs 32.64%<br>τ_2 6908.44 μs ±1451.00 μs 67.36%
**1B**		
**CH_3CN**	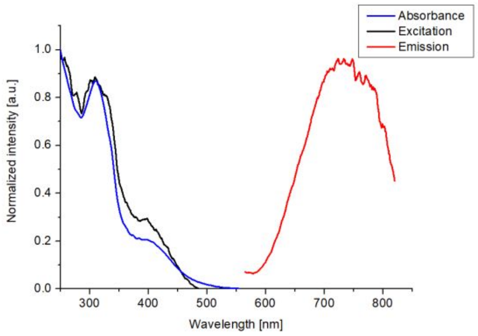	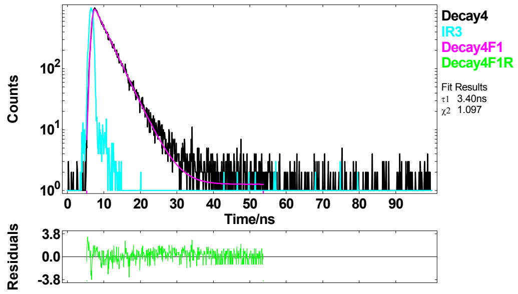<br>Fit results:<br>τ_1 3.40 ns ±0.02 ns 100%
**$CHCl_3$**	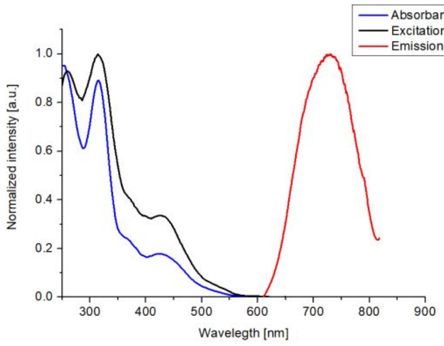	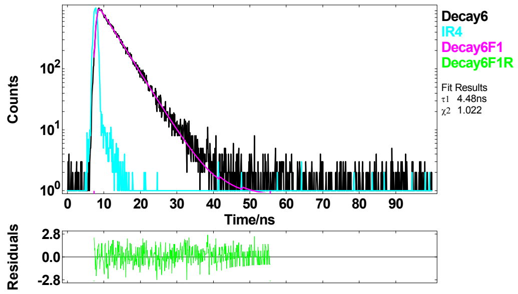<br>Fit results:<br>τ_1 4.48 ns ±0.02 ns 100%

<table>
<tr><td>Solid</td><td>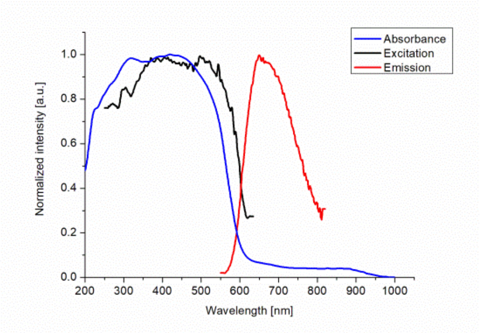
</td><td>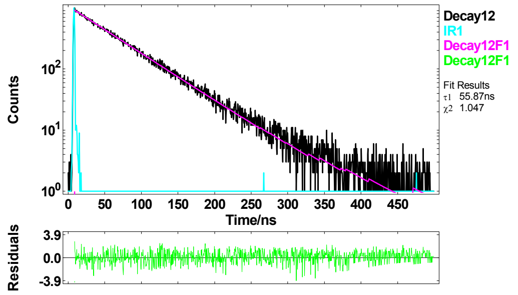

Fit results:
τ_1 55.87 ns ±0.20 ns 100%</td></tr>
<tr><td>BuCN (77K)</td><td>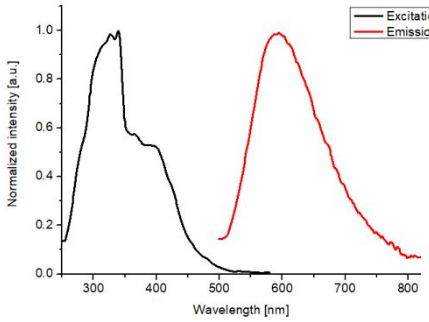
</td><td>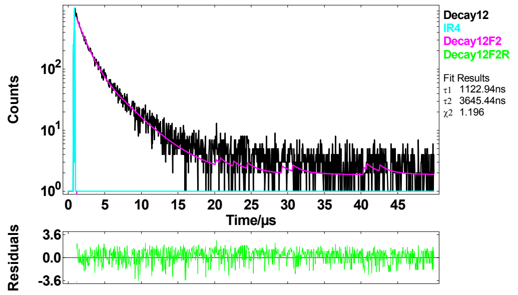

Fit results:
τ_1 1122.94 ns ±40.25 ns 60.46%
τ_2 3645.44 ns ±120.80 ns 39.54%</td></tr>
<tr><td colspan="3">2B</td></tr>
<tr><td>CH_3CN</td><td>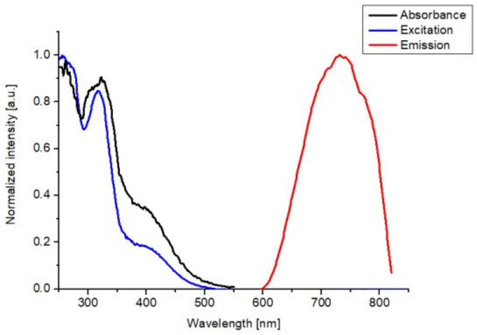
</td><td>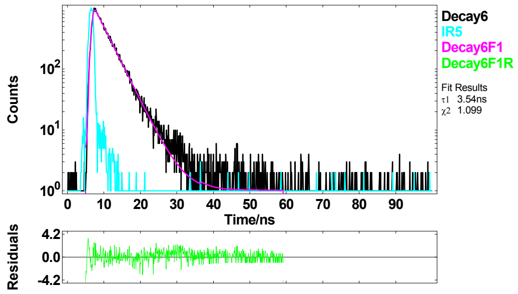

Fit results:
τ_1 3.54 ns ±0.02 ns 100%</td></tr>
</table>

**$CHCl_3$**	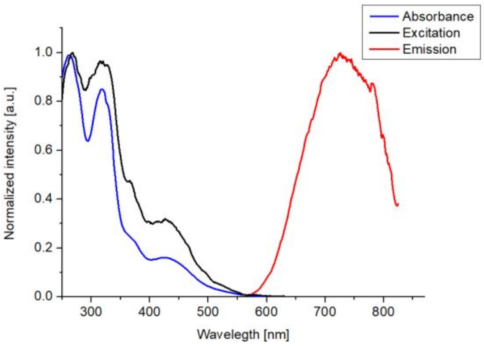	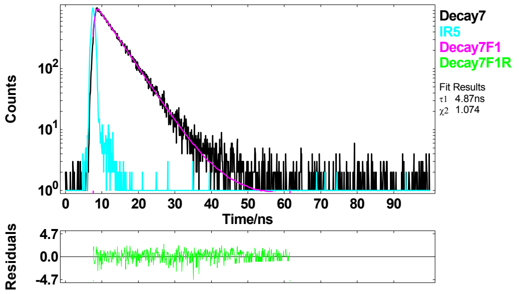 Fit results:<br>τ_1 4.87 ns ±0.02 ns 100%
**Solid**	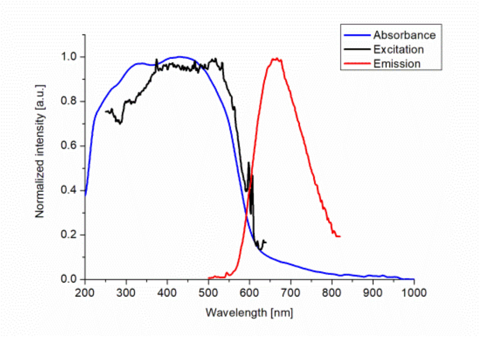	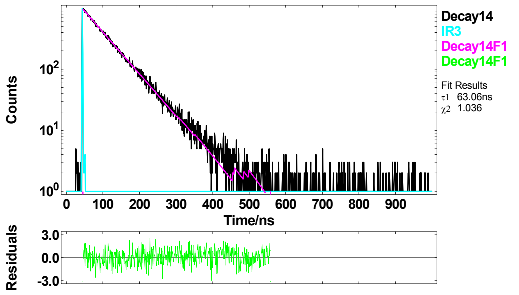 Fit results:<br>τ_1 63.06 ns ±0.29 ns 100%
**BuCN (77K)**	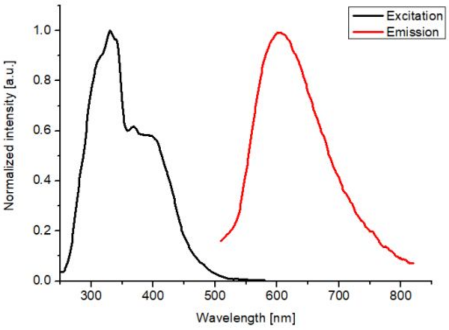	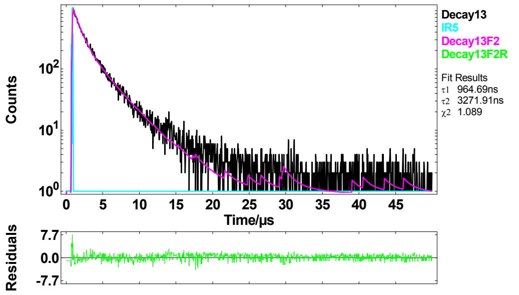 Fit results:<br>τ_1 964.69 ns ±32.34 ns 41.36%<br>τ_2 3271.91 ns ±65.21 ns 58.64%

<table>
<tr><th colspan="3">3B</th></tr>
<tr><td>CH_3CN</td><td>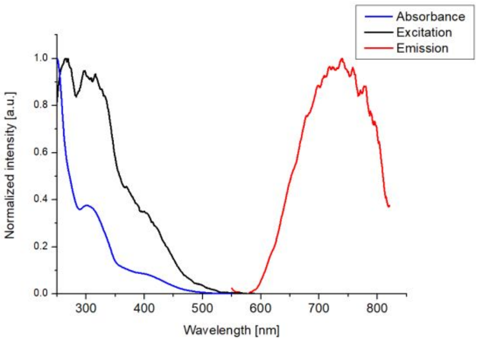
</td><td>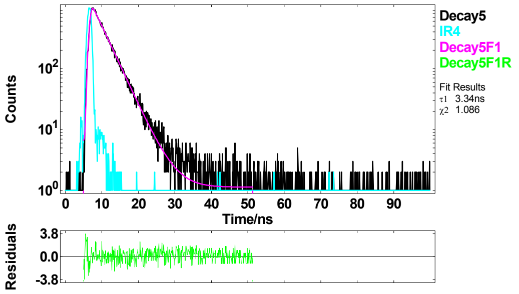

Fit results:
τ_1 3.34 ns ±0.02 ns 100%</td></tr>
<tr><td>$CHCl_3$</td><td>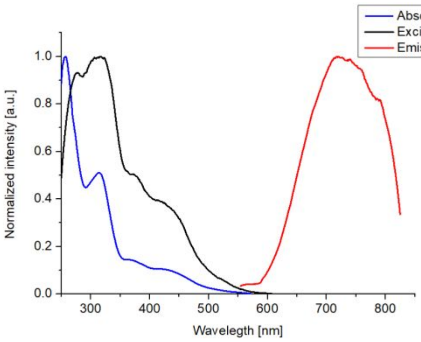
</td><td>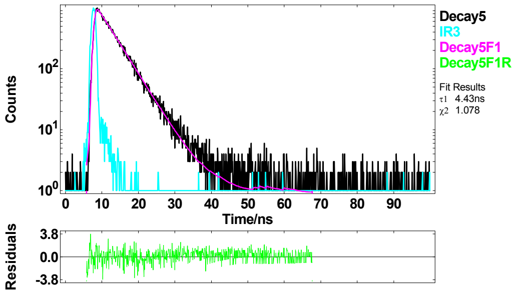

Fit results:
τ_1 4.43 ns ±0.02 ns 100%</td></tr>
<tr><td>Solid</td><td>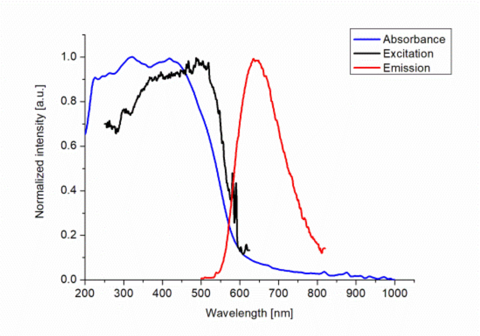
</td><td>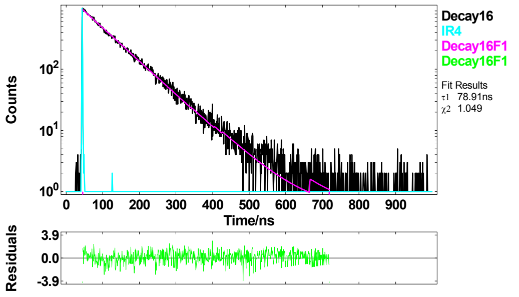

Fit results:
τ_1 78.91 ns ±0.33 ns 100%</td></tr>
</table>

<table>
<tr><td>BuCN (77K)</td><td>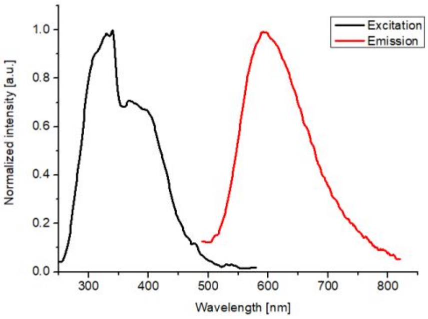
</td><td>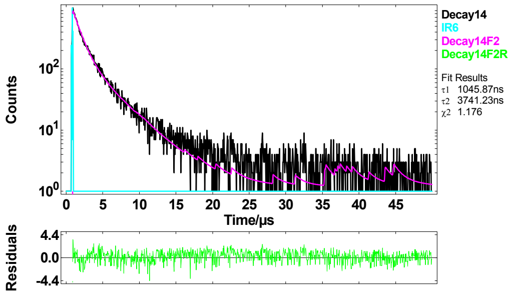

Fit results:
τ_1 1045.87 ns ±29.28 ns 55.80%
τ_2 3741.23 ns ±99.44 ns 44.20%</td></tr>
<tr><td colspan="3">4B</td></tr>
<tr><td rowspan="2">CH_3CN</td><td rowspan="2">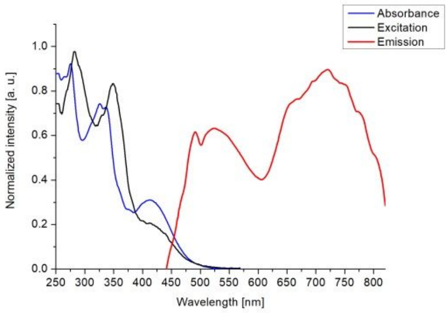
</td><td>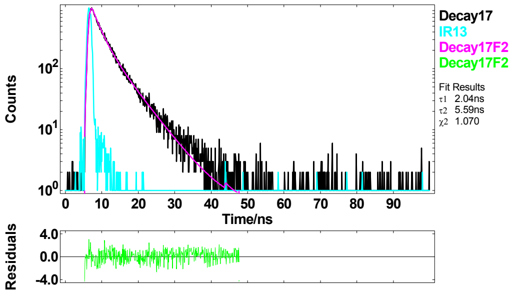

Fit results:
τ_1 2.04 ns ±0.09 ns 43.91%
τ_2 5.59 ns ±0.17 ns 56.09%</td></tr>
<tr><td>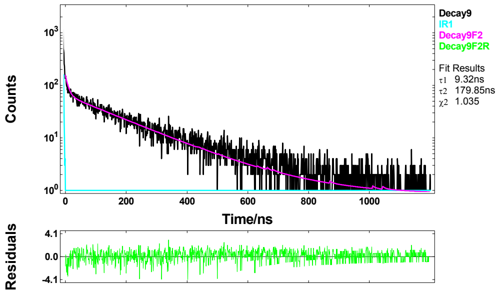

Fit results:
τ_1 9.32 ns ±0.91 ns 33.37%
τ_2 179.85 ns ±3.05 ns 66.63%</td></tr>
</table>

<table>
<tr>
<td rowspan="2">$CHCl_3$</td>
<td rowspan="2">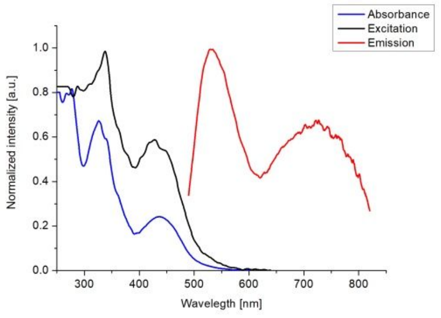
</td>
<td>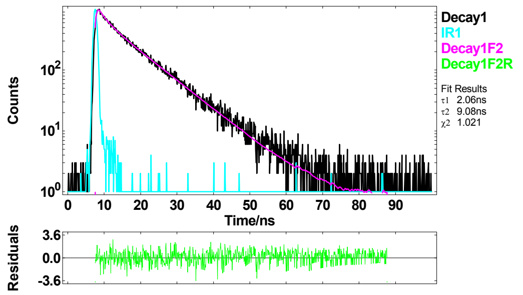

Fit results:
τ_1 2.06 ns ±0.16 ns 12.32%
τ_2 9.08 ns ±0.07 ns 87.68%</td>
</tr>
<tr>
<td>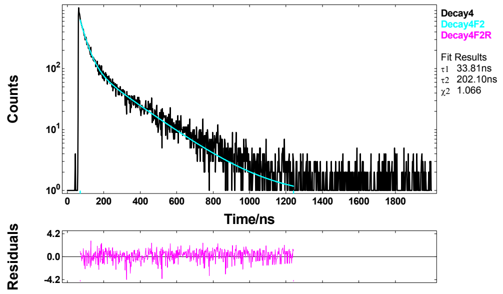

Fit results:
τ_1 33.81 ns ±0.99 ns 42.30%
τ_2 202.10 ns ±5.29 ns 57.70%</td>
</tr>
<tr>
<td>BuCN (77K)</td>
<td>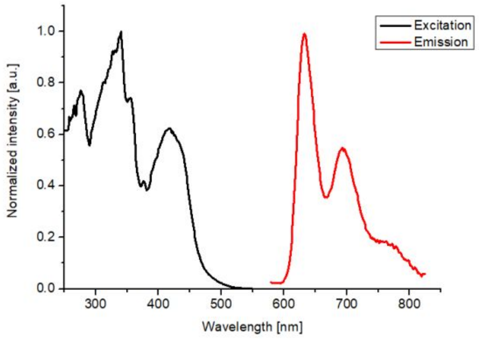
</td>
<td>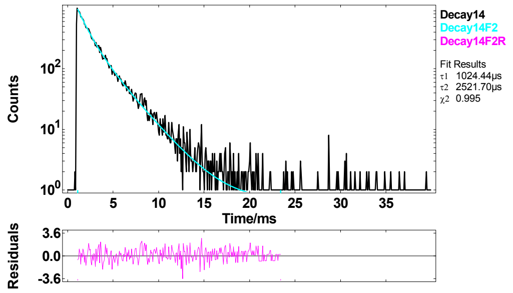

Fit results:
τ_1 1024.44 μs ±77.59 μs 39.26%
τ_2 2521.70 μs ±105.40 μs 60.74%</td>
</tr>
</table>

Figure S37. Emission spectral data of **1A–4A** and **1B–4B** in two solvents of different polarity ($CHCl_3$, ε = 4.8 and CH_3CN, ε = 37.5), rigid matrix at 77 K (BuCN) and solid state as a powder.

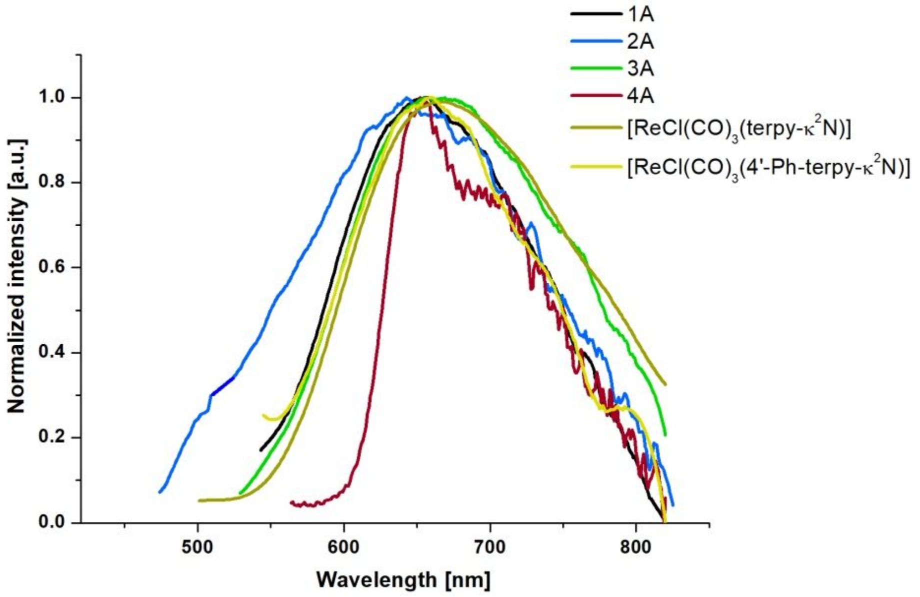

Figure S38. Normalized emission spectra of **1A–4A** in $CHCl_3$, along with the emission spectra of [ReCl(CO)$_3$(terpy-κ^{2}N)] and [ReCl(CO)$_3$(4′-Ph-terpy-κ^{2}N)]. Emission spectra of [ReCl(CO)$_3$(terpy-κ^{2}N)], [ReCl(CO)$_3$(4′-Ph-terpy-κ^{2}N)] and in $CHCl_3$ were Reproduced from ref. [16-17]. Copyright John Wiley and Sons 2018 and Royal Society of Chemistry 2020.

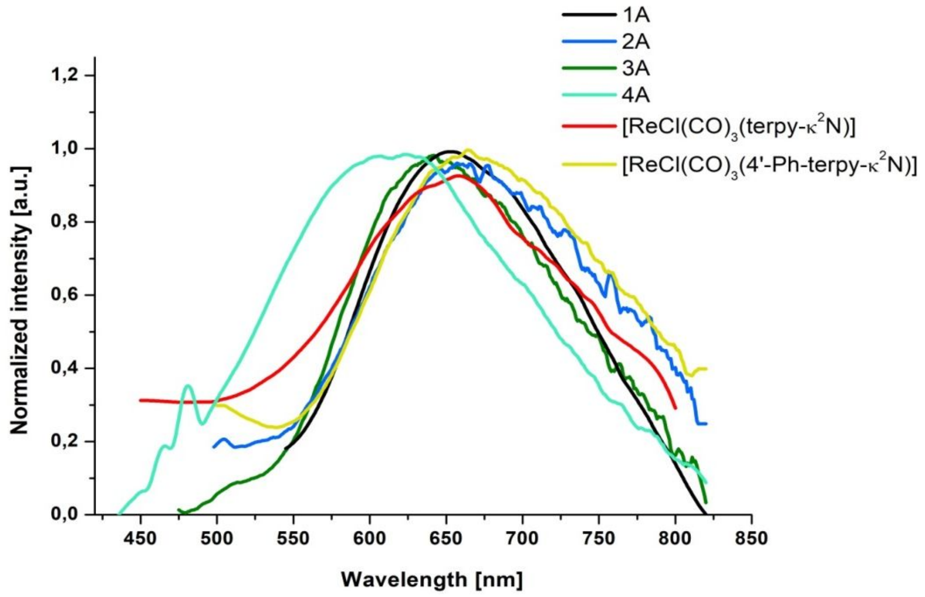

Figure S39. Normalized emission spectra of **1A–4A** in CH_3CN, along with the emission spectra of [ReCl(CO)$_3$(terpy-κ^{2}N)] and [ReCl(CO)$_3$(4′-Ph-terpy-κ^{2}N)]. Emission spectra of [ReCl(CO)$_3$(terpy-κ^{2}N)], [ReCl(CO)$_3$(4′-Ph-terpy-κ^{2}N)] and in CH_3CN were Reproduced from ref. [16-17]. Copyright John Wiley and Sons 2018 and Royal Society of Chemistry 2020.

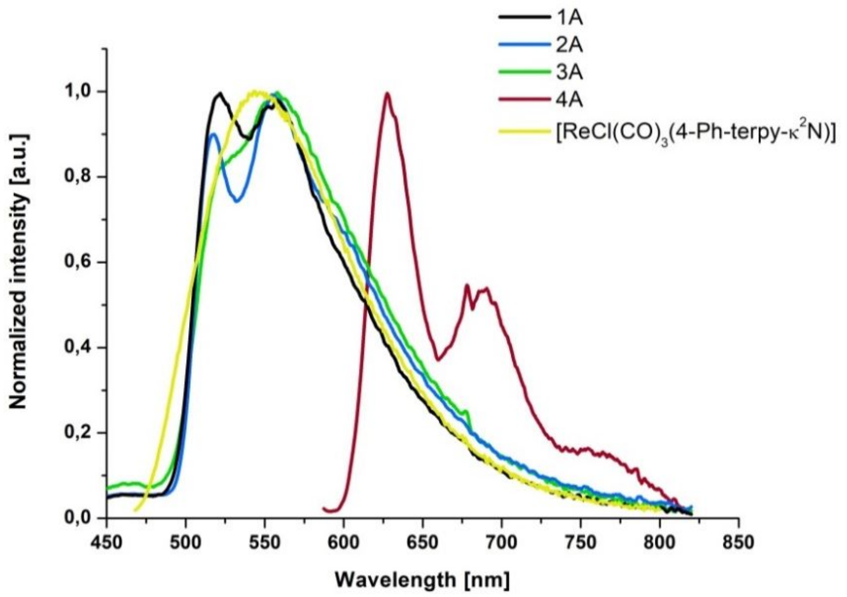

Figure S40. Normalized emission spectra of 1A–4A in rigid matrix at 77 K, along with the emission spectra of [ReCl(CO)$_3$(terpy-κ^{2}N)]. Emission spectra of [ReCl(CO)$_3$(terpy-κ^{2}N)], in 77K was reproduced from ref [16]. Copyright John Wiley and Sons 2018.

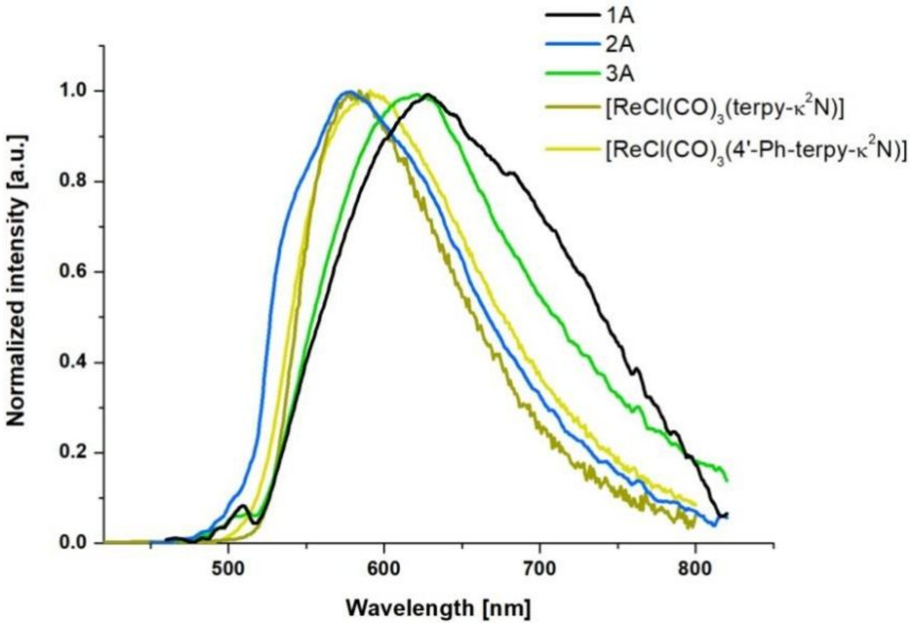

Figure S41. Normalized emission spectra of **1A–4A** in the solid state, along with the emission spectra of [ReCl(CO)$_3$(terpy-κ^{2}N)] + [ReCl(CO)$_3$(4′-Ph-terpy-κ^{2}N)]. Emission spectra of [ReCl(CO)$_3$(terpy-κ^{2}N)], [ReCl(CO)$_3$(4′-Ph-terpy-κ^{2}N)] and in solid state were reproduced from ref. [16-17]. Copyright John Wiley and Sons 2018 and Royal Society of Chemistry 2020.

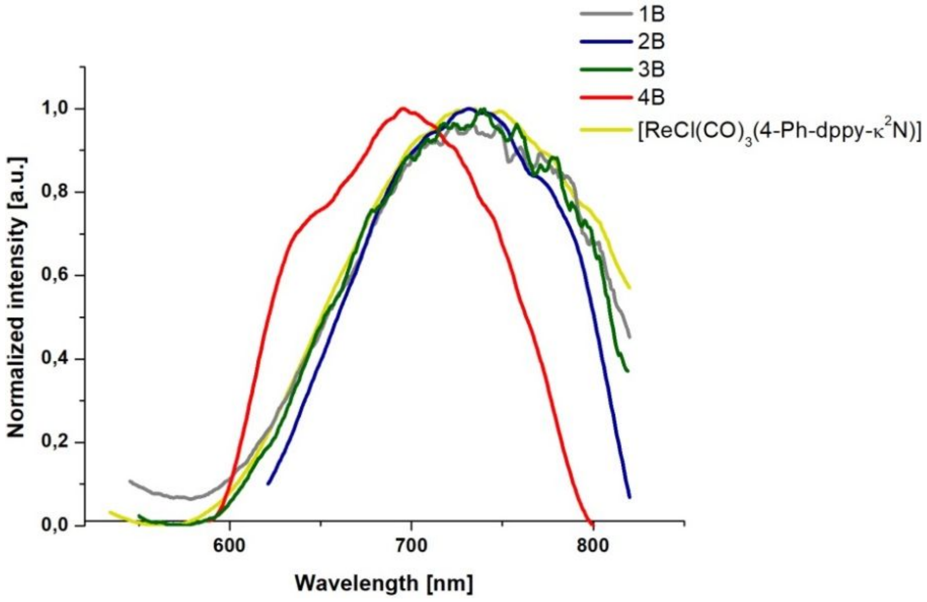

Figure S42. Normalized phosphorescence spectra of **1B–4B** in CH_3CN, along with the emission spectrum of [ReCl(CO)$_3$(4-Ph-dppy-κ^{2}N)]. Emission spectra of [ReCl(CO)$_3$(4-Ph-dppy-κ^{2}N)] in CH_3CN was reproduced from ref [16]. Copyright John Wiley and Sons 2018.

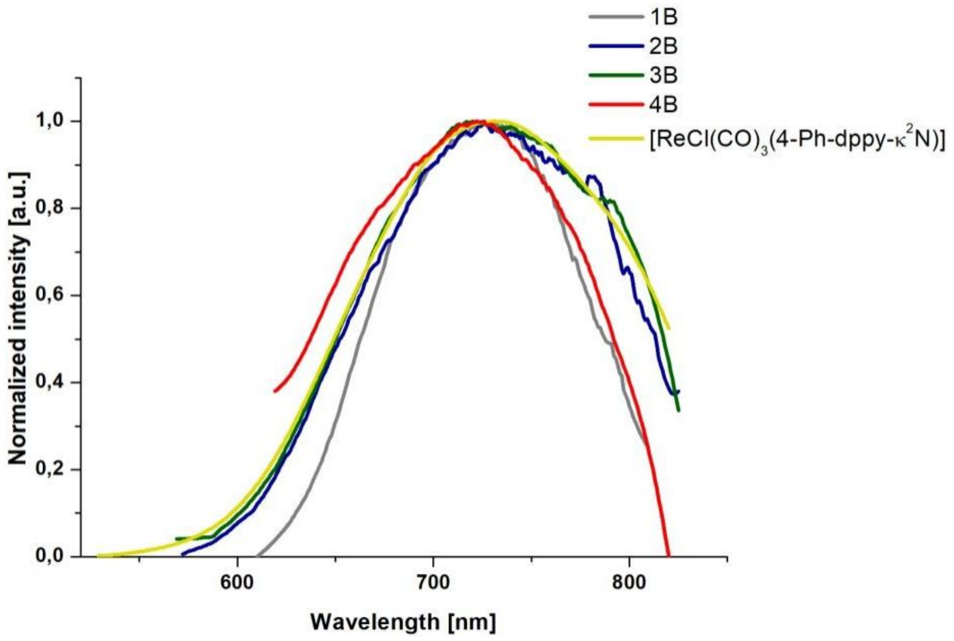

Figure S43. Normalized phosphorescence spectra of **1B–4B** in $CHCl_3$, along with emission spectrum of [ReCl(CO)$_3$(4-Ph-dppy-κ^{2}N)]. Emission spectra of [ReCl(CO)$_3$(4-Ph-dppy-κ^{2}N)] in $CHCl_3$ was reproduced from ref [16]. Copyright John Wiley and Sons 2018.

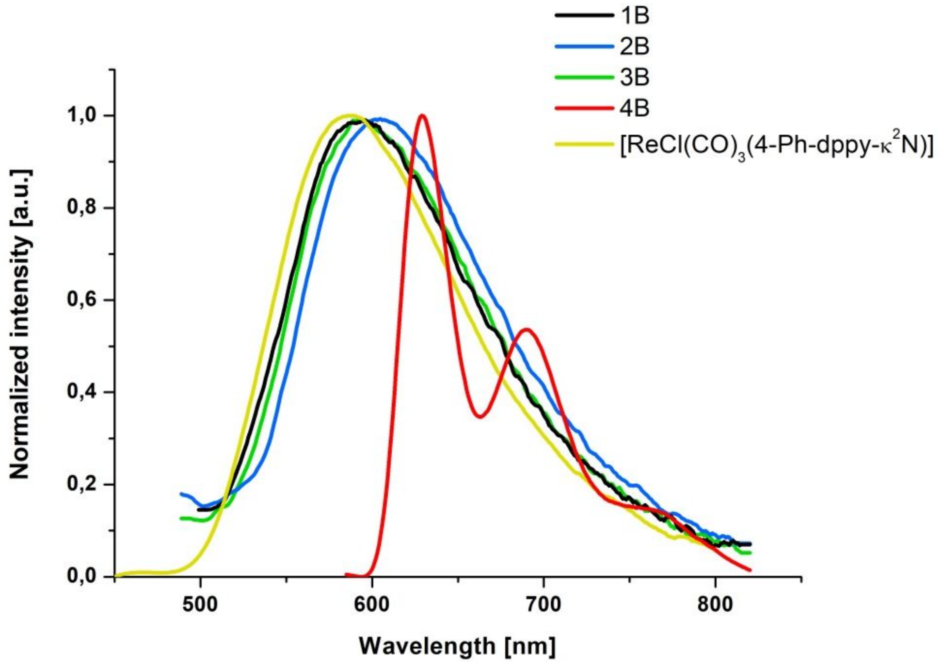

Figure S44. Normalized emission spectra of **1B–4B** in the rigid matrix at 77 K, along with the emission spectrum of [ReCl(CO)$_3$(4-Ph-dppy-κ^{2}N)]. Emission spectra of [ReCl(CO)$_3$(4-Ph-dppy-κ^{2}N)] in 77K was reproduced from ref [16]. Copyright John Wiley and Sons 2018.

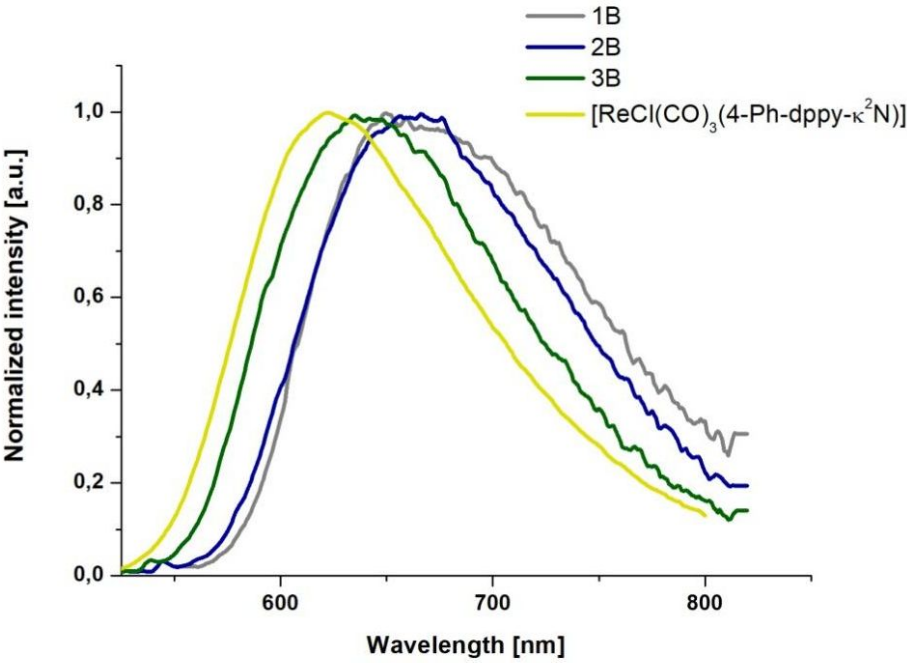

Figure S45. Normalized emission spectra of **1B–3B** in the solid state, along with the emission spectrum of [ReCl(CO)$_3$(4-Ph-dppy-κ^{2}N)]. Emission spectra of [ReCl(CO)$_3$(4-Ph-dppy-κ^{2}N)] in solid state was reproduced from ref [16]. Copyright John Wiley and Sons 2018.

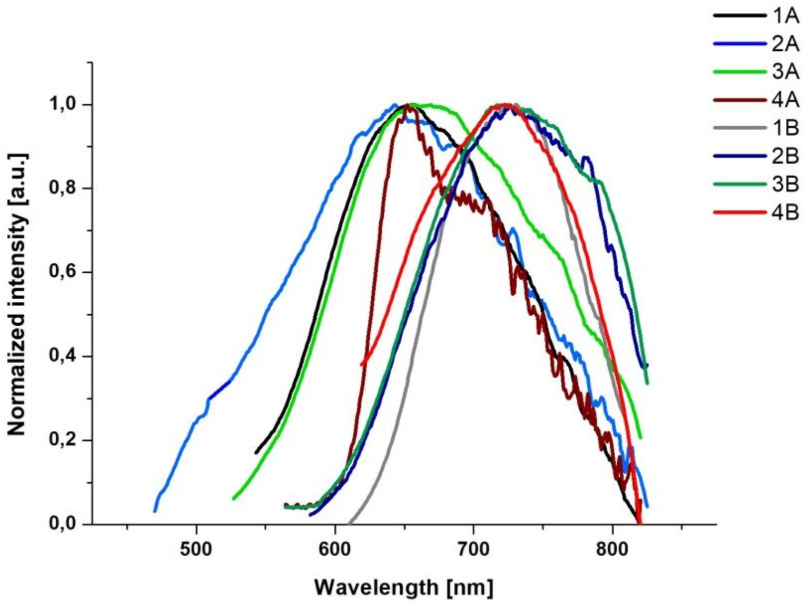

Figure S46. Normalized phosphorescence spectra of **1A–4A** and **1B–4B** in $CHCl_3$.

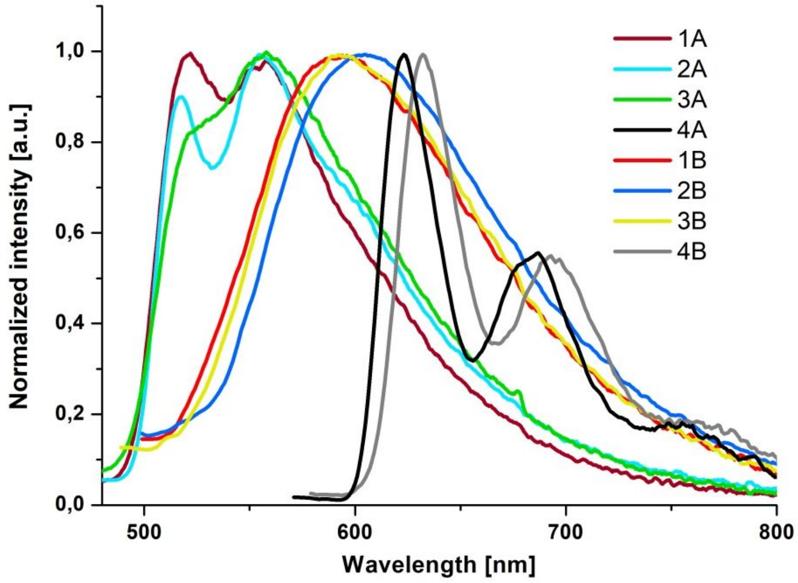

Figure S47. Normalized phosphorescence emission spectra of **1A**–**4A** and **1B**–**4B** at 77 K.

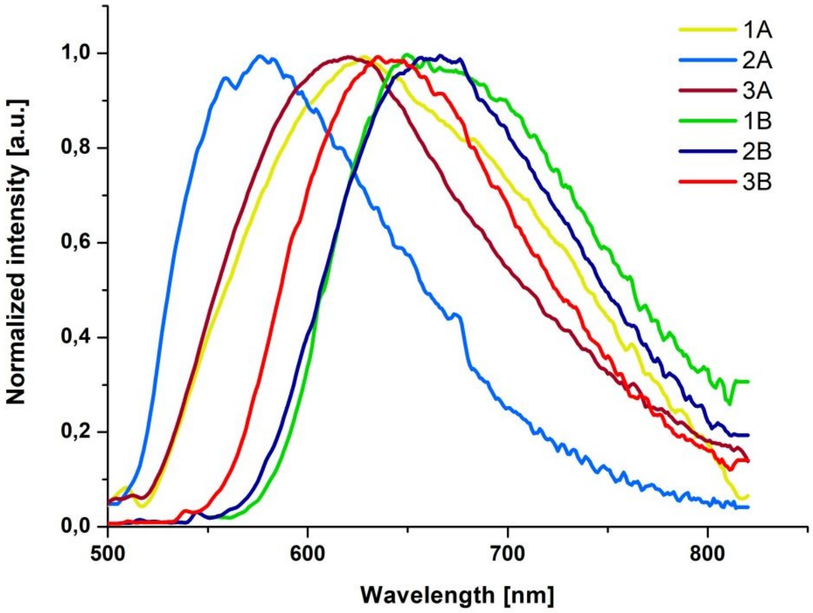

Figure S48. Normalized emission spectra of **1A**–**3A** and **1B**–**3B** in the solid state.

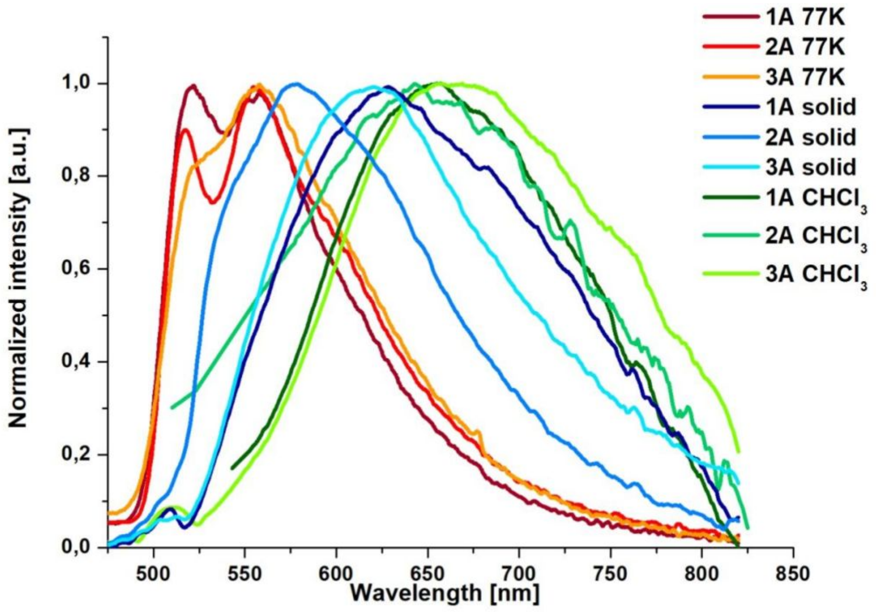

Figure S49. Normalized emission spectra of **1A–3A** in $CHCl_3$, solid state and rigid matrix at 77 K.

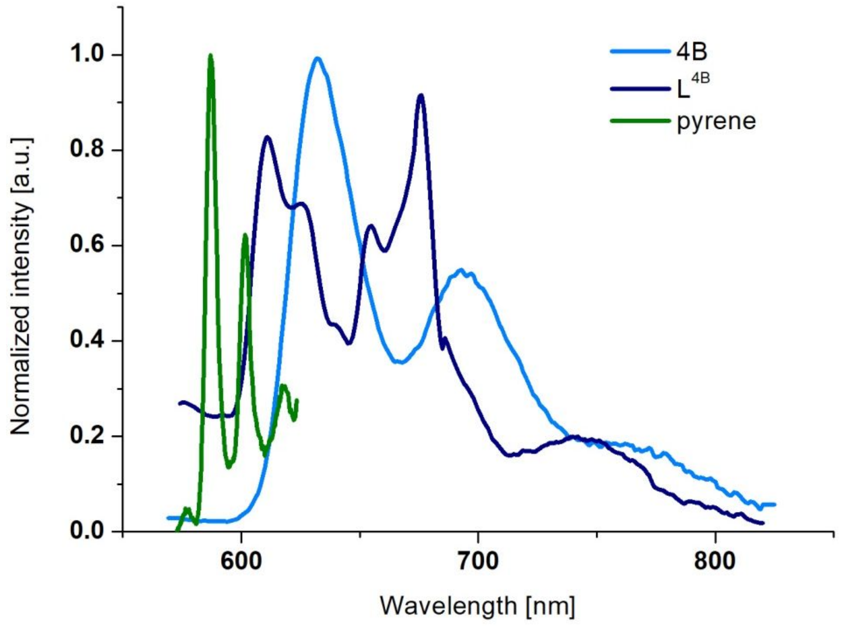

Figure S50. Phosphorescence spectra of **4B** at 77 K along with the phosphorescence spectra of the free ligands and pyrene.

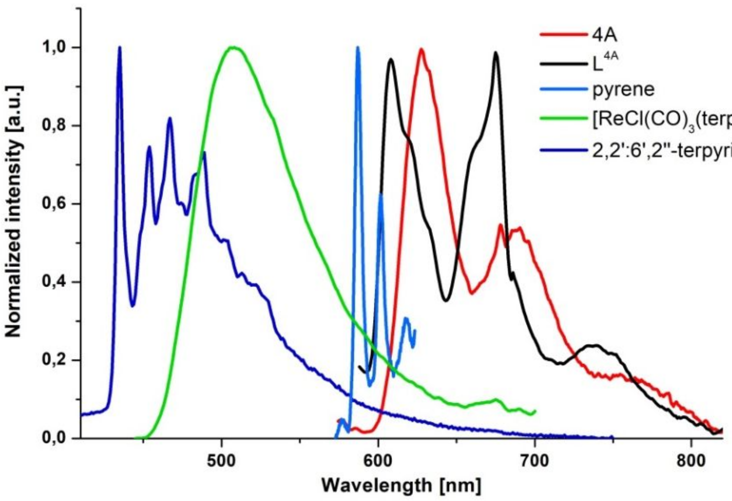

Figure S51. Phosphorescence spectra of **4A** and [ReCl(CO)$_3$(terpy-κ^{2}N)] at 77K along with the phosphorescence spectra of the free ligands and pyrene.

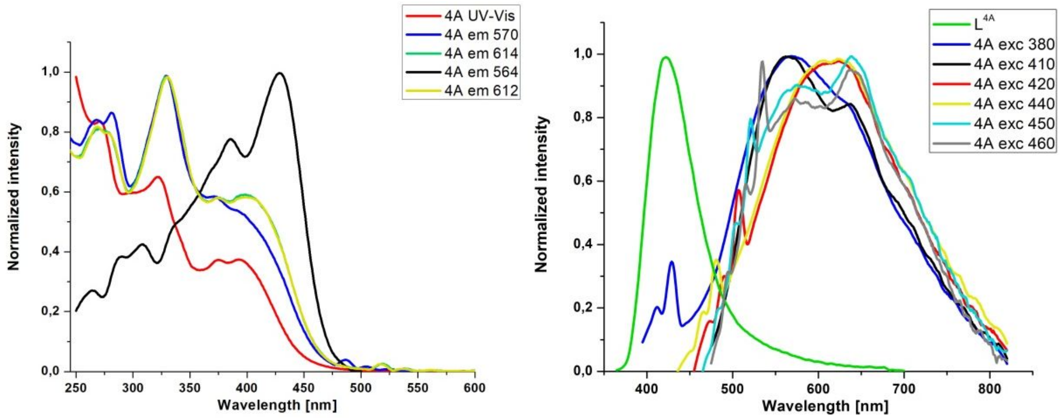

Figure S52. Excitation and emission spectra of **4A** in CH_3CN.

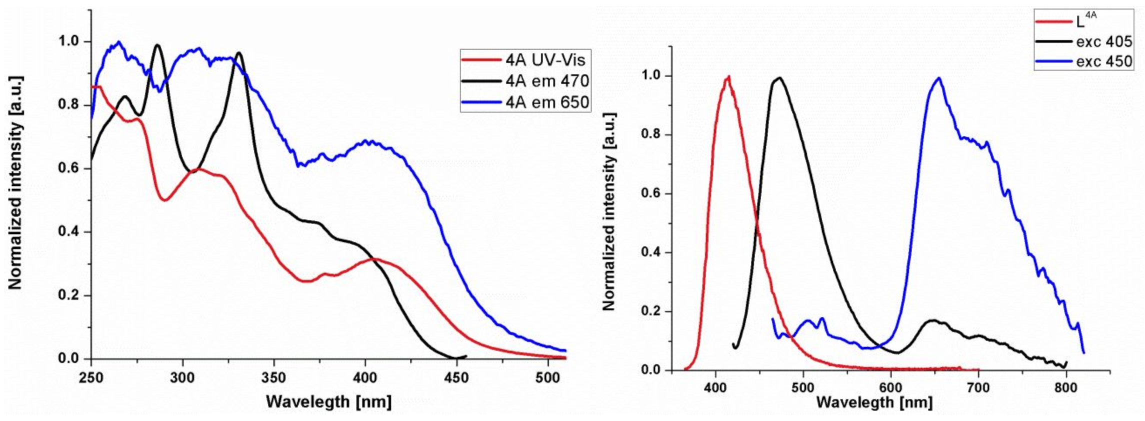

Figure S53. Excitation and emission spectra of **4A** in $CHCl_3$.

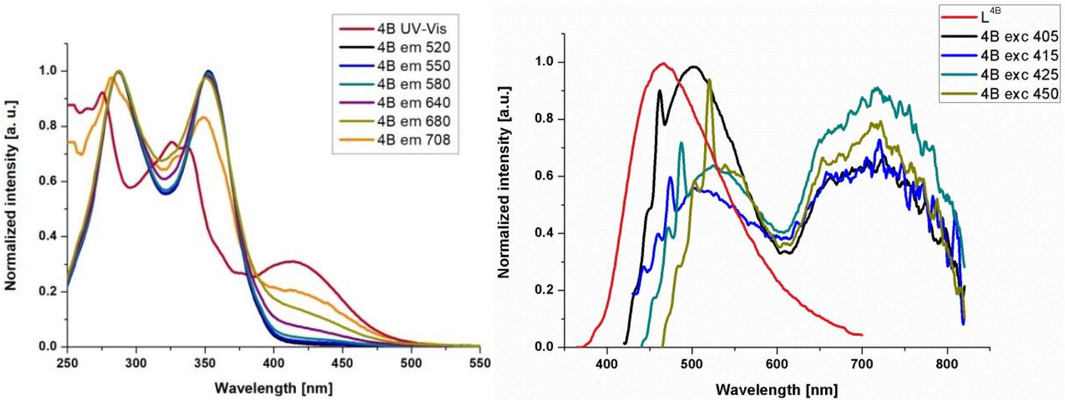

Figure S54. Excitation and emission spectra of **4B** in CH_3CN.

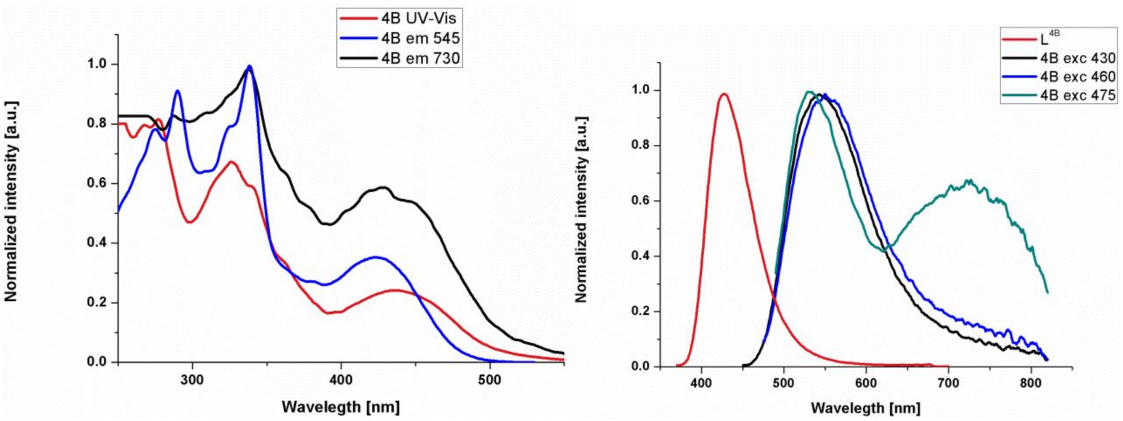

Figure S55. Excitation and emission spectra of **4B** in $CHCl_3$.

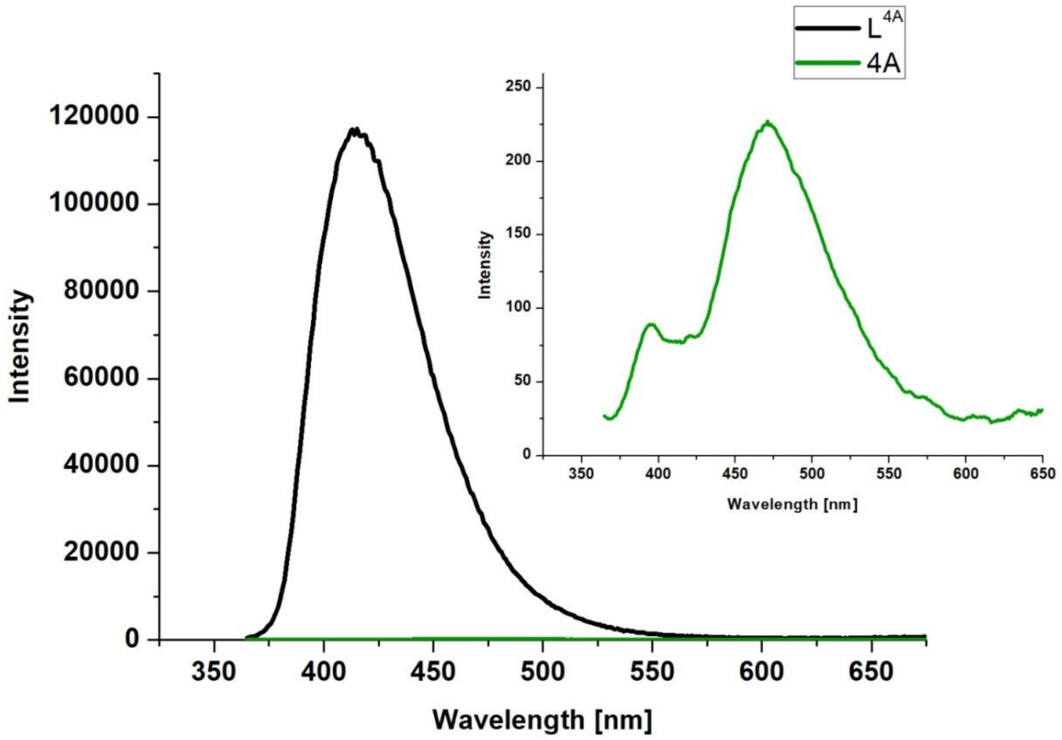

Figure S56. Comparison of fluorescence band of $\mathbf{L^{4A}}$ and **4A** in $\mathbf{CHCl_3}$ estimated at excitation wavelength 350nm. EnT estimated as 99%

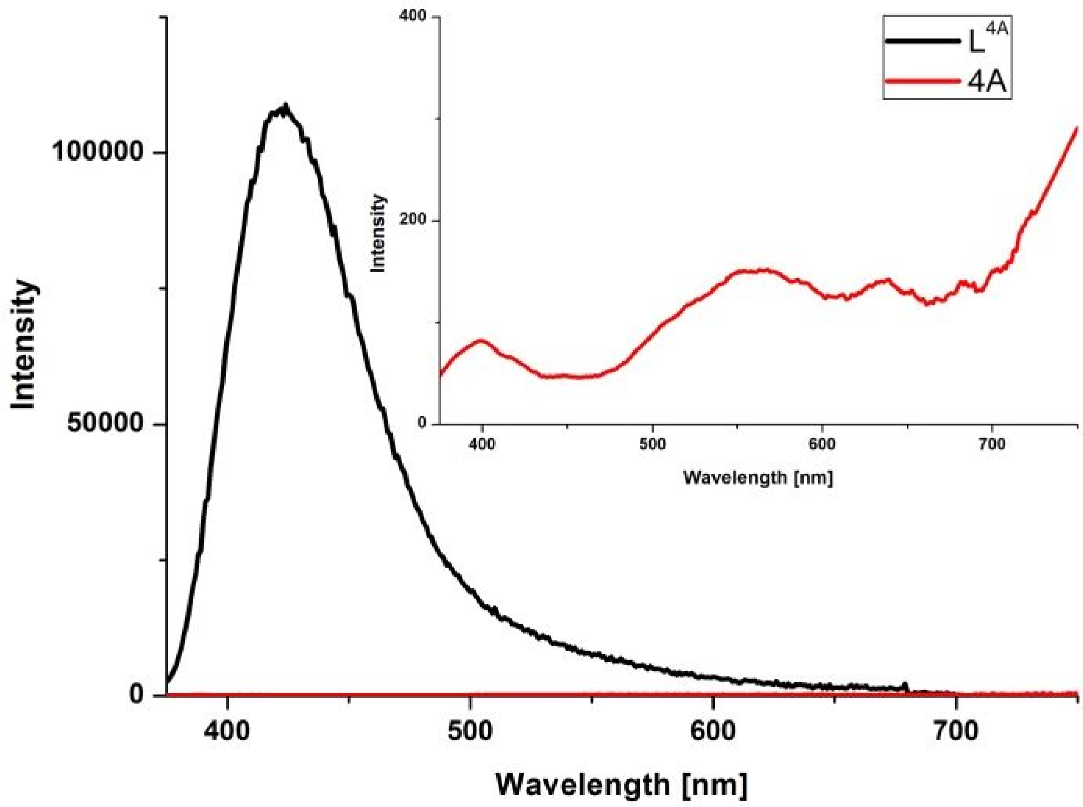

Figure S57. Comparison of fluorescence band of $\mathbf{L^{4A}}$ and **4A** in $\mathbf{CH_3CN}$ estimated at excitation wavelength 350nm. EnT estimated as 87%.

<table>
<tr><th>CH_3CN</th><th>$CHCl_3$</th></tr>
<tr><td colspan="2">4A</td></tr>
<tr><td>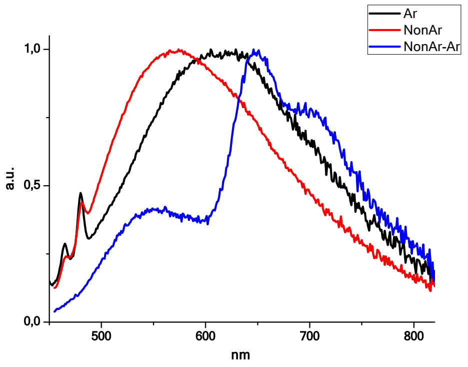
</td><td>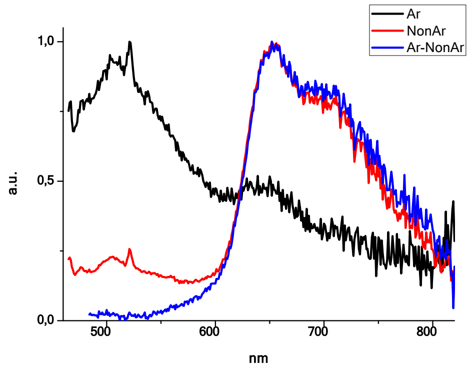
</td></tr>
<tr><td colspan="2">4B</td></tr>
<tr><td>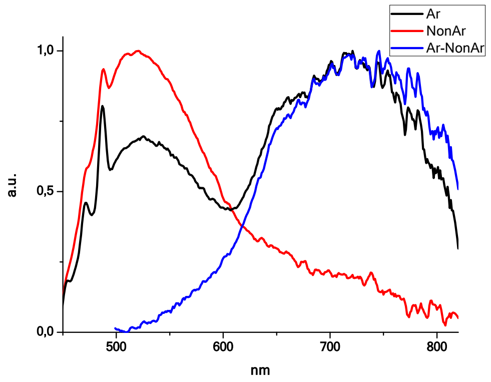
</td><td>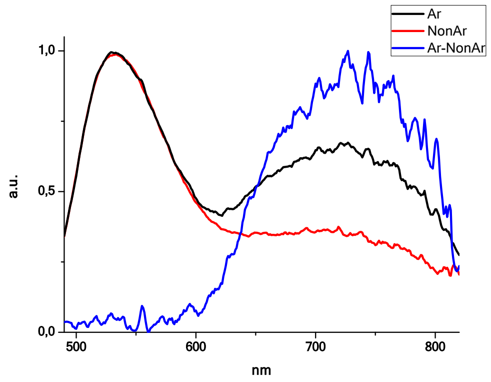
</td></tr>
</table>

Figure S58. Emission spectra of deaerated and aerated solutions of 4A and 4B in CH3CN $CHCl_3$ solutions and, demonstrating that The emission peak at longer wavelength arises from a triplet state.

Phosphorescence emissions – computational data

Table S12. The energies of theoretical phosphorescence emissions, calculated from the difference between the ground singlet and the triplet state $\Delta E_{T_1-S_0}$, along with the experimental values and the spin density surface plots for **1A**–**3A**. Grey and green colours show regions of excess α spin density and excess β spin density values, respectively.

Code	Experimental emission maximum	Calculated emission maximum	Spin density surface plots
**1A**	654 nm (1.89 eV)	686 (1.80 eV)	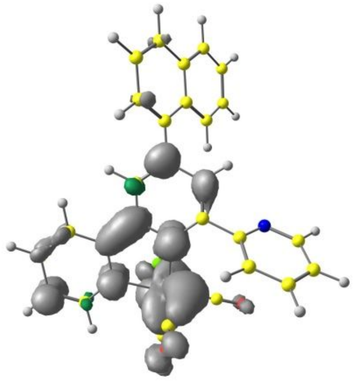
**2A**	663 (1.87 eV)	697 (1.77 eV)	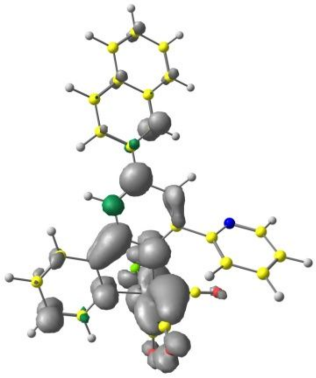
**3A**	641 (1.93 eV)	687 (1.80 eV)	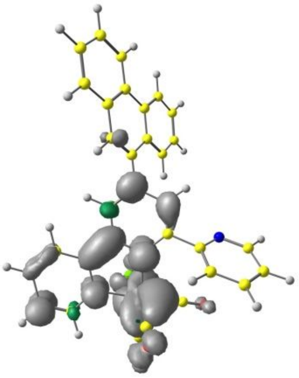

Table S13. The energies of theoretical phosphorescence emissions, calculated from the difference between the ground singlet and the triplet state $\Delta E_{T_1-S_0}$, along with the experimental values and the spin density surface plots for **4A** and **4B**. Grey and green colours show regions of excess α spin density and excess β spin density values, respectively.

Code	Experimental emission maximum	Calculated emission maximum	Spin density surface plots
**4A**	620 (1.99 eV)	783 (1.58 eV)	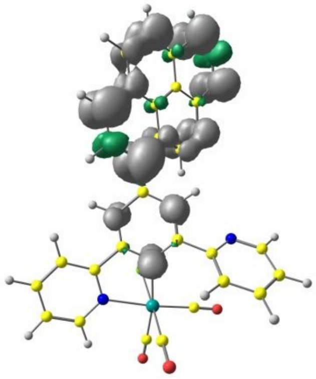
**4B**	708 (1.75 eV)	792 (1.56 eV)	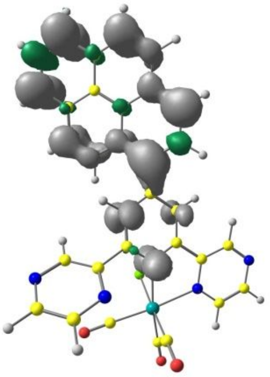

Stability and photo-stability of 4A and 4B

Photo-stability experiment was prepared with the use of NanoDrop One C spectrophotometer in range 250–600 nm during 24h for solutions of **4A** and **4B** (25µM). Photoclevage experiment was conducted using 450W Xe arc lamp as a source of continuous radiation. 10 mm cuvette with the sample solution was placed in sample holder of FLS-980 nm, and exposed to radiation for a given period of time. The light coming from Xe lamp was first monochromated to 420nm and passed through the slit of 10 nm.

**Photostability**	
**CH_3CN**	**$CHCl_3$**
**4A**	
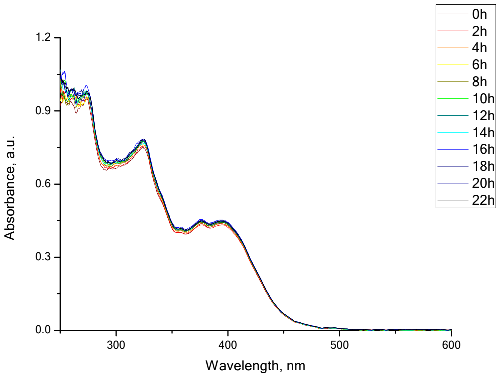	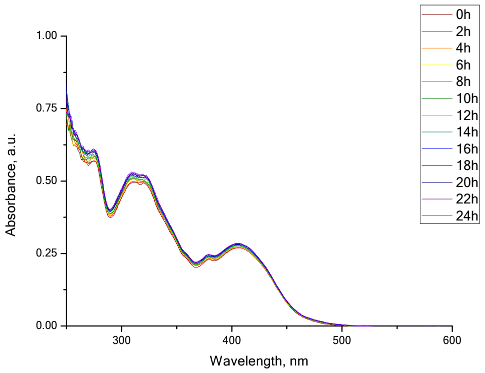
**4B**	
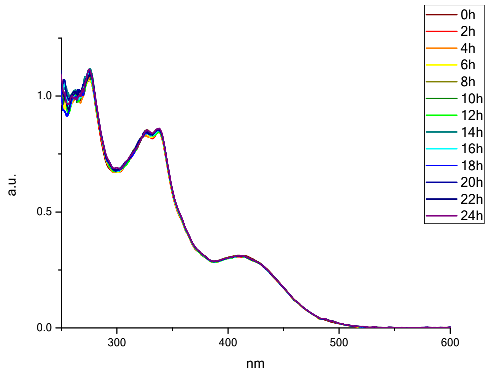	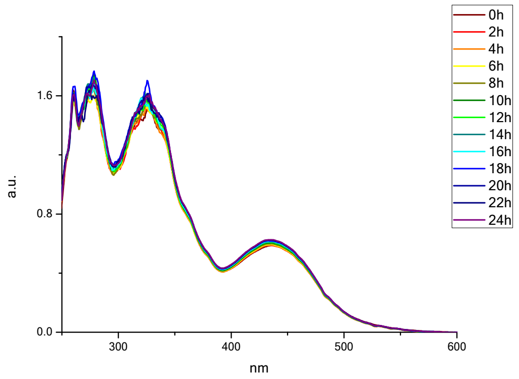

Figure S59. UV-Vis spectra **4A** and **4B** in CH_3CN and $CHCl_3$ recorded once every two hours over 24h at room temperature.

Photoclevage

CH_3CN	$CHCl_3$

4A

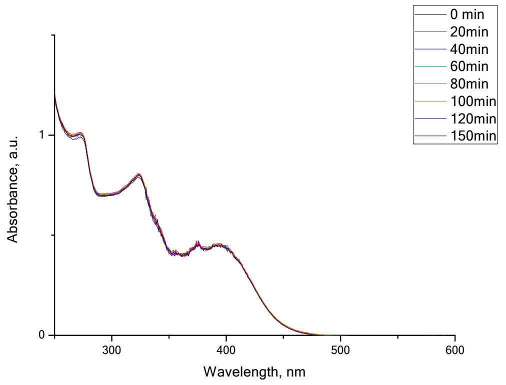

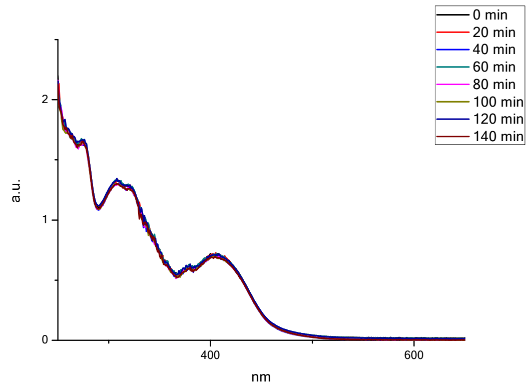

4B

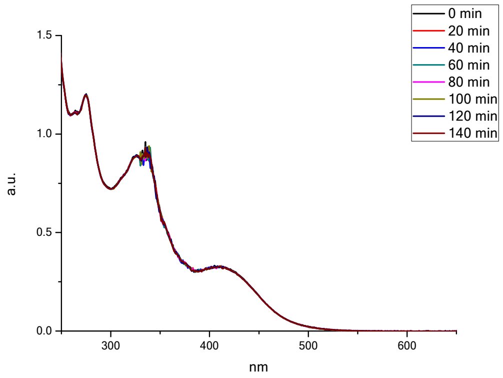

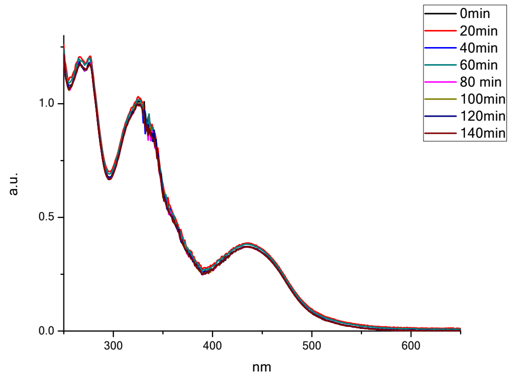

Figure S60. UV-Vis spectra **4A** and **4B** in CH_3CN and $CHCl_3$ after exposure of Xe arc lamp radiation.

Time-resolved emission map

The series of time correlating single photon counting (TCSPC) experiments were performed with 5 nm emission wavelength step. Prior to the experiment, optimisation of the signal rate on the photomultiplier tube (PMT) and the time conditions was acquired for emission maximum wavelength obtained from steady state experiments. Each single decay curve was collected for 300s (77K) or 600s (room temperature). Emission wavelength range depends on sample.

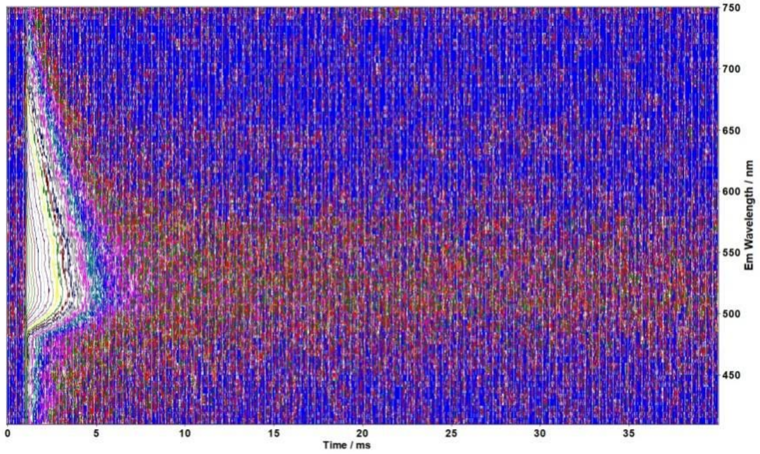	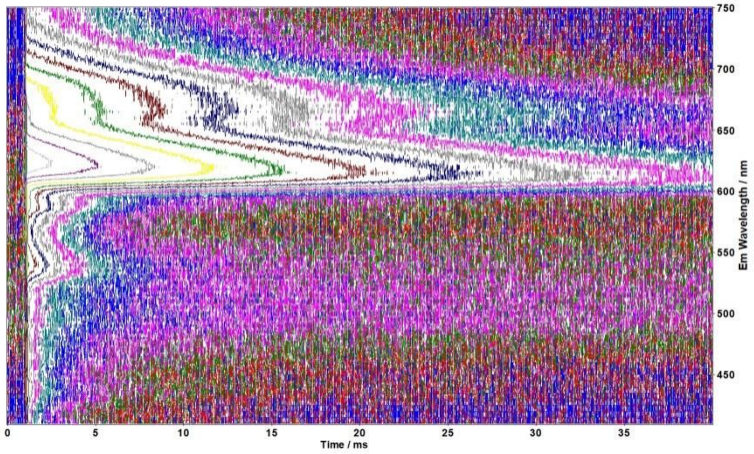
**1A**	**4A**

Figure S61. Comparison of TRES spectra of **1A** and **4A** in butyronitrile rigid matrix at 77 K (time window 40ms, excitation wavelength 395 nm). TRES map of **4A** displays two emission bands: ~540 nm disappearing within 10ms time window as well as ~620 nm persisted to the end of time window (which is consistent with steady state phosphorescence emission of 4A at 77K). The band of **4A** at ~540 nm is overlapped with ^{3}MLCT emission of **1A**.

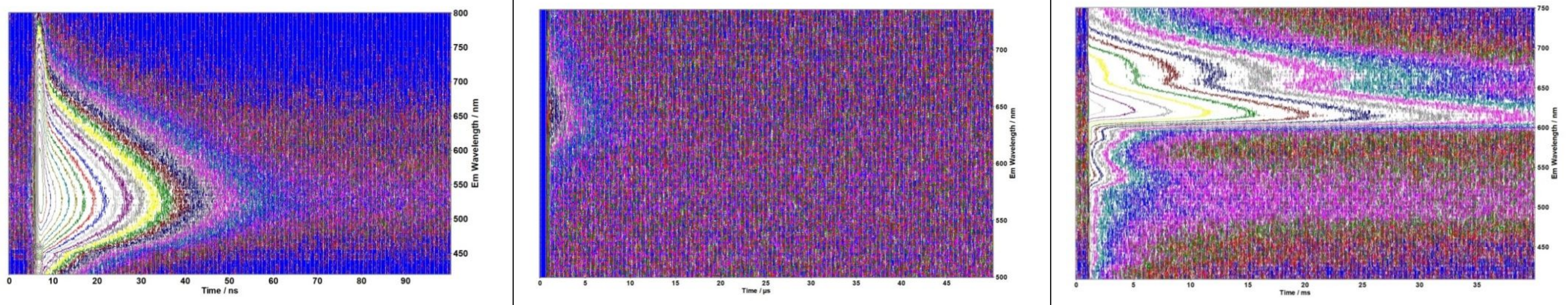

CH_3CN	$CHCl_3$	77 K

Figure S62. Comparison of TRES map of **4A** at room temperature (RT) in CH_3CN (time window 100ns, excitation wavelength 405 nm) and in $CHCl_3$ (time window 50µs, excitation wavelength 475 nm) and low temperature (77 K). TRES of **4A** at RT in $CHCl_3$ displays only one emission band comparable to 620 nm emission band observed in the TRES at 77K, while for CH_3CN sample whole spectral region is covered by strong fluorescence signal.

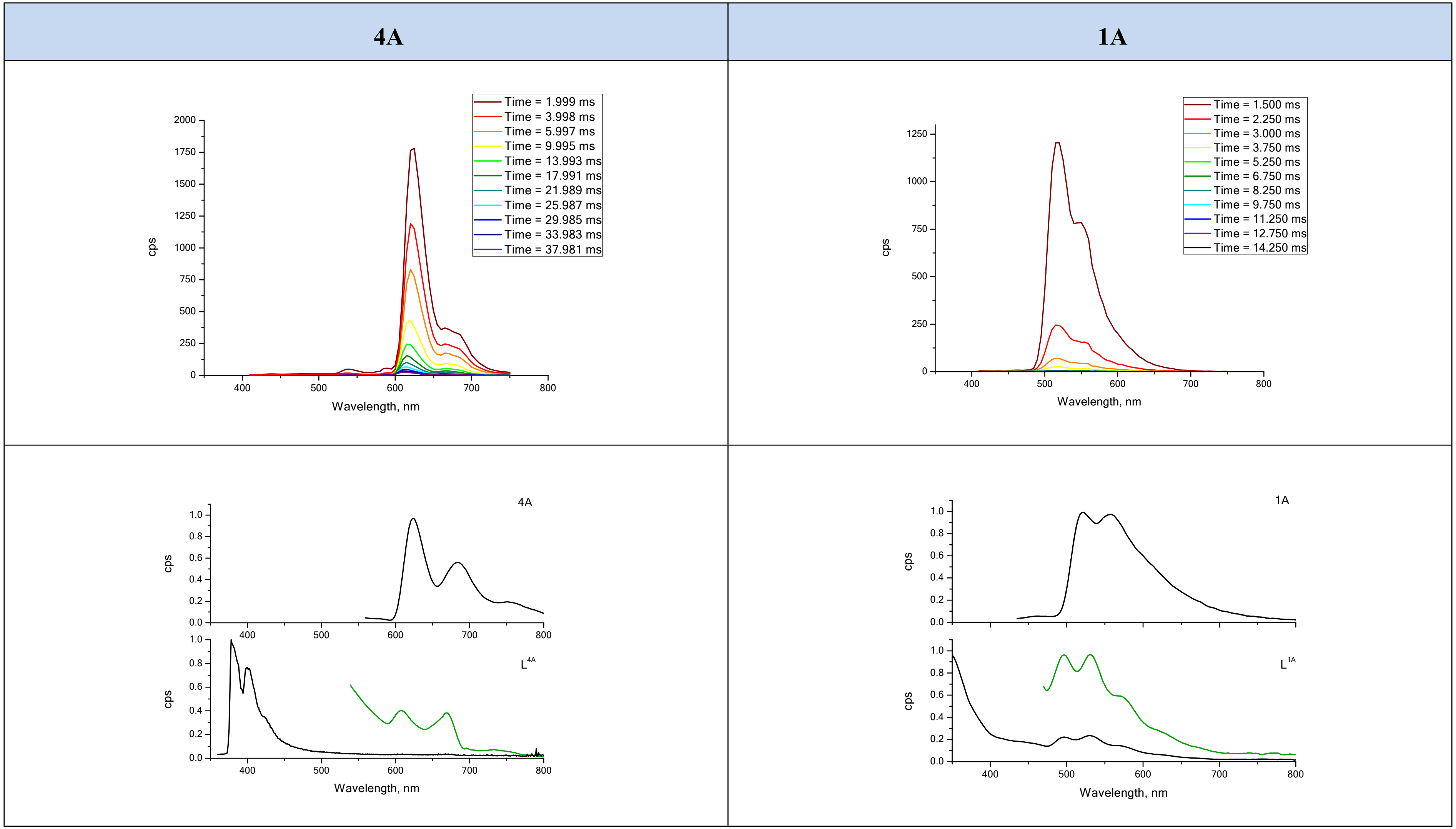

Figure S63. Comparison of TRES slices of **4A**, **1A** and steady-state emission spectra of pairs **4A-L^{4A}** and **1A-L^{1A}** at 77K (in case of spectra of ligands black line represent fluorescence spectrum, while green line represent phosphorescence spectrum).

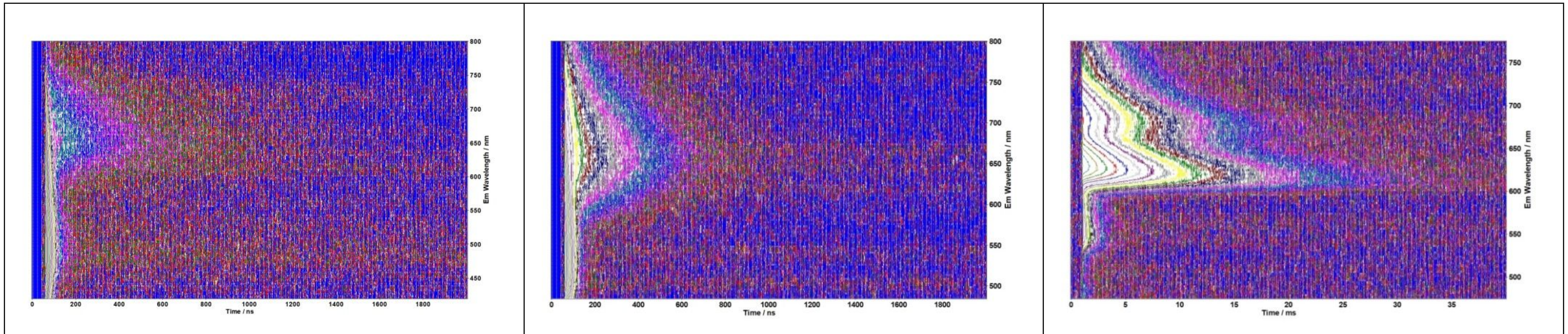

CH_3CN	$CHCl_3$	77K

Figure S64. Comparison of TRES maps of **4B** at room temperature (RT, time window 2μs, excitation wavelength 405 nm) in CH_3CN and $CHCl_3$ and low temperature (77 K, time window 40μs, excitation wavelength 420 nm). TRES map of **4B** at 77K displays two emission bands: ~540 nm disappearing within 5ms time window as well as ~610 nm disappearing within 25ms (which is consistent with steady state phosphorescence emission of **4B** at 77K). TRES maps of **4B** at RT in both solvents (CH_3CN and $CHCl_3$) show that phosphorescence band is affected by strong, prompt fluorescence signal, therefore in wavelength range of phosphorescence band biexponential fits of decay curves appear.

Preliminary investigations of the PL ability of Re(I) complexes in film blends

Investigations of the PL ability of Re(I) complexes in thin film revealed that only two compounds, that is, with *terpy* core bearing 2-naphthyl unit (**2A**) and *dppy* with attached 1-naphthyl group (**1B**) were emissive (Table S14 and Figure S65). In PL spectra of blends consisting of 15wt% complexes dispersed molecularly in PVK:PBD, two (**1A**–**3A** and **3B**) or one (**4A**, **1B**, **2B**, and **4B**) emission bands were seen (Table S14). Whereas the intense band observed at shorter wavelengths originate from the emission of the matrix (Figure S66), weaker peaks at lower energy region can be ascribed to the photoluminescence of complex (**1A**–**3A** and **3B**). Thus, in the case of blends with **4A**, **1B**, **2B**, and **4B** only emission of PVK:PBD was detected. The PL measurements results proved, that in the case of blends with 15wt% content of complex, the energy transfer from the matrix to the luminophore is not complete or absent at all. Next, the content of complex in matrix was reduced to 2wt%, and in PL spectra of all blends emission from the complex was also weakly pronounced, except for **2B** and **4B**.

Table S14. Photoluminescence of Re(I) complexes in thin films and blends with of 15wt% complexes dispersed molecularly in PVK:PBD.

**Compound**	**Film**		**Blend**	
	**λ_{ex} [nm]**	**λ_{em} [nm]**	**λ_{ex} [nm]**	**λ_{em} [nm]**
**1A**	390	nd	310	378, 570
**2A**	381	567	310	372, 387, 570
**3A**	385	nd	310	373, 390, 567
**4A**	429	nd	310	380
**1B**	412	617	310	377
**2B**	410	nd	310	375
**3B**	400	nd	310	379, 594
**4B**	444	nd	310	379

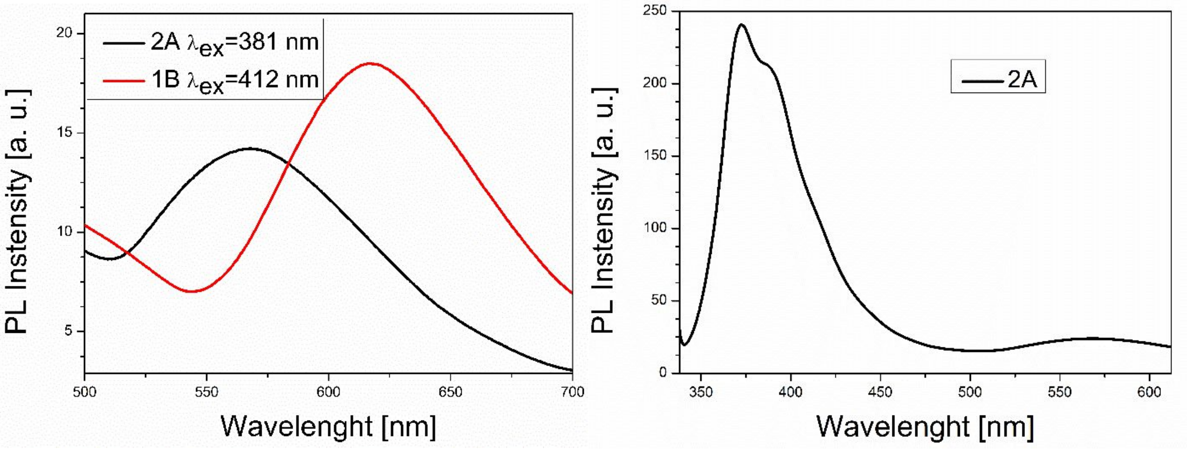

Figure S65. PL spectra of 2A in film and blend (15wt.%).

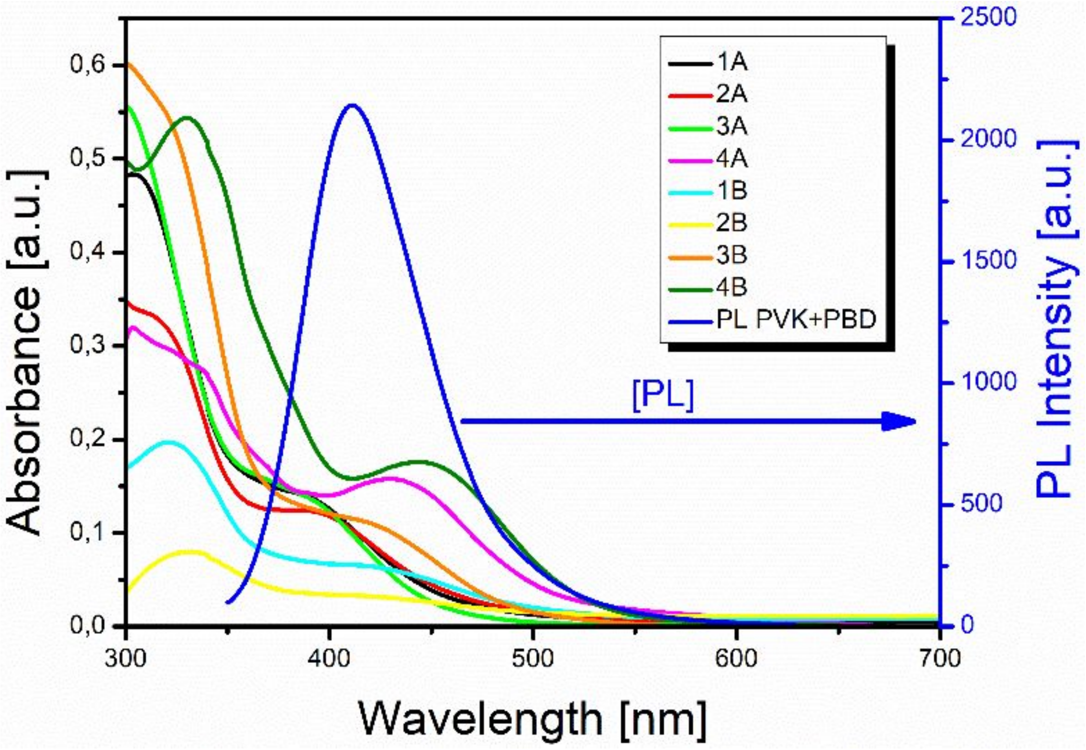

Figure S66. UV-Vis absorption spectra of 1A–4A and 1B–4B in film together with photoluminescence (PL) spectrum of PVK:PBD.

Two compounds **2A** and **1B**, which showed PL in film, were applied as active layer in devices with structure: ITO/PEDOT:PSS/complex/Al, but the prepared diodes were not emissive. The diodes with guest-host configuration (ITO/PEDOT:PSS/PVK:PBD:complex/Al) with all complexes, except for **2B** and **4B**, were also fabricated. The devices emitted light with maximum electroluminescence band (λ_{EL}) in the range of 595-650 nm with various intensity (Figure S67). The highest EL intensity was induced by high applied external voltage (18-27 V).

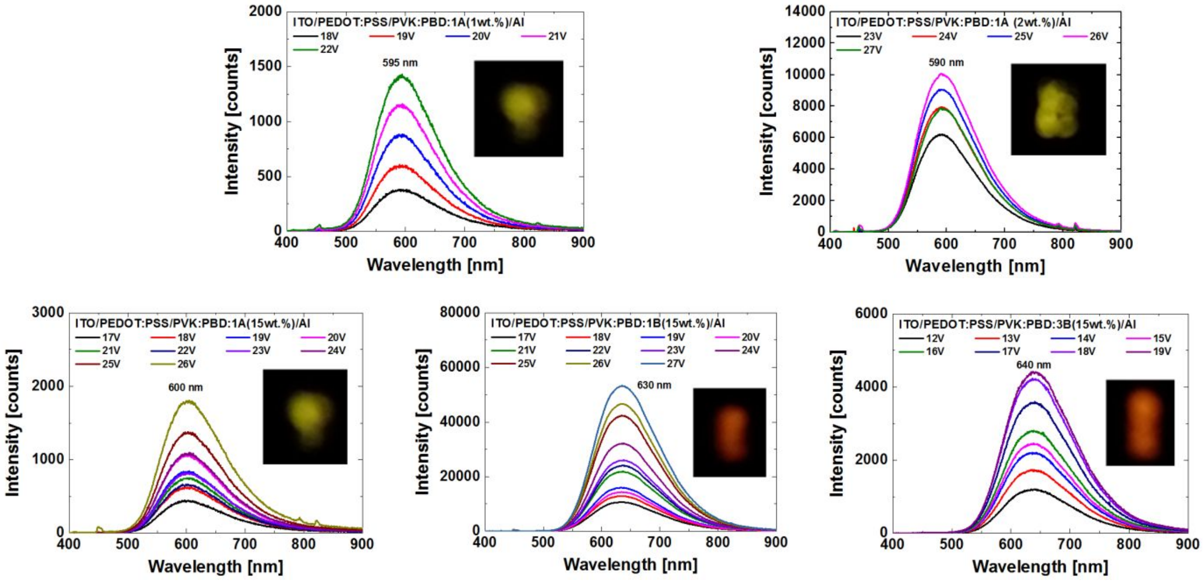

(a)

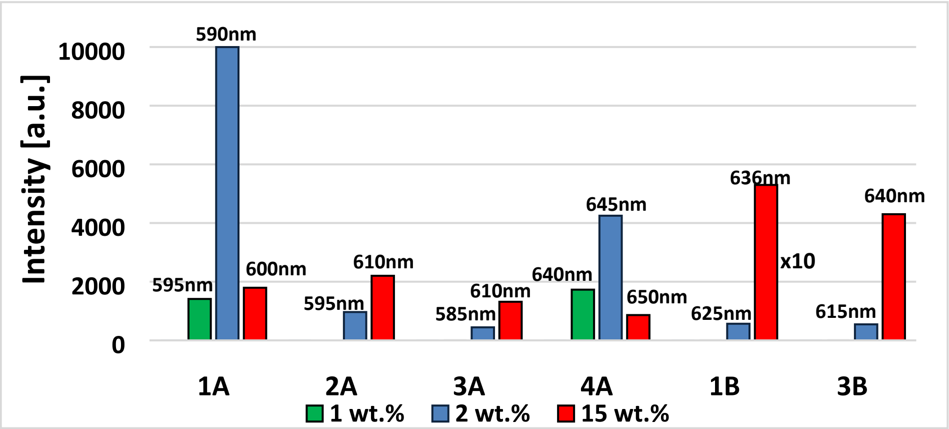

(b)

Figure S67. (a) Electroluminescence spectra of selected diodes and (b) diagram with maximal reached EL intensity with λ_{EL} position.

The most intense light emission exhibited diode containing complex consisting of *dppy* core substituted with 1-naphthyl unit (**1B**) with its 15wt% content in active layer. The reduction of Re(I) carbonyl complex content from 15 to 2wt.% resulted in lowering of emitted light intensity, except for **1A** and **4A**, for which the EL intensity significantly increased. It can be explained considering the PL spectra of **1A** and **4A** dispersed in the PVK:PBD matrix (Figure S68). One can notice from Figure S64 that the intensity of PL band at longer wavelength in blend with 2wt.% content of **1A** is more pronounced compared to the blend with higher amount of this complex. In the case of **4A,** the band originated from the emission of the complex is seen only in blend bearing 2 wt.%. Further reduction of **1A** and **4A** content to 1 wt.% did not raise the EL intensity and was lower compared to device with 2wt.% complex in active layer.

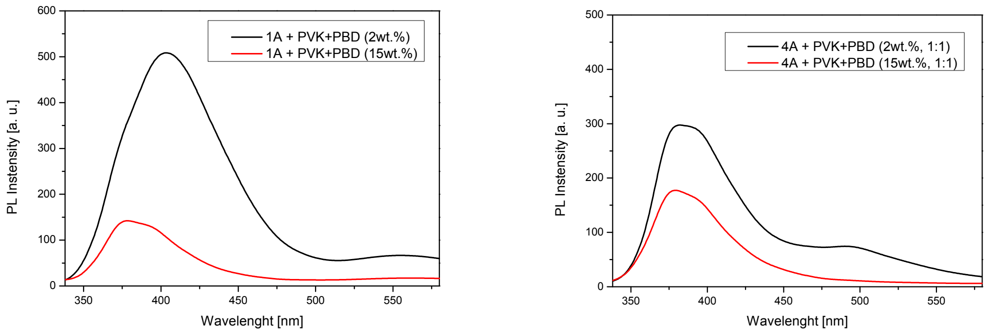

Figure S68. The PL spectra of **1A** and **4A** dispersed molecularly in PVK:PBD matrix.

Nano- and femtosecond transient absorption

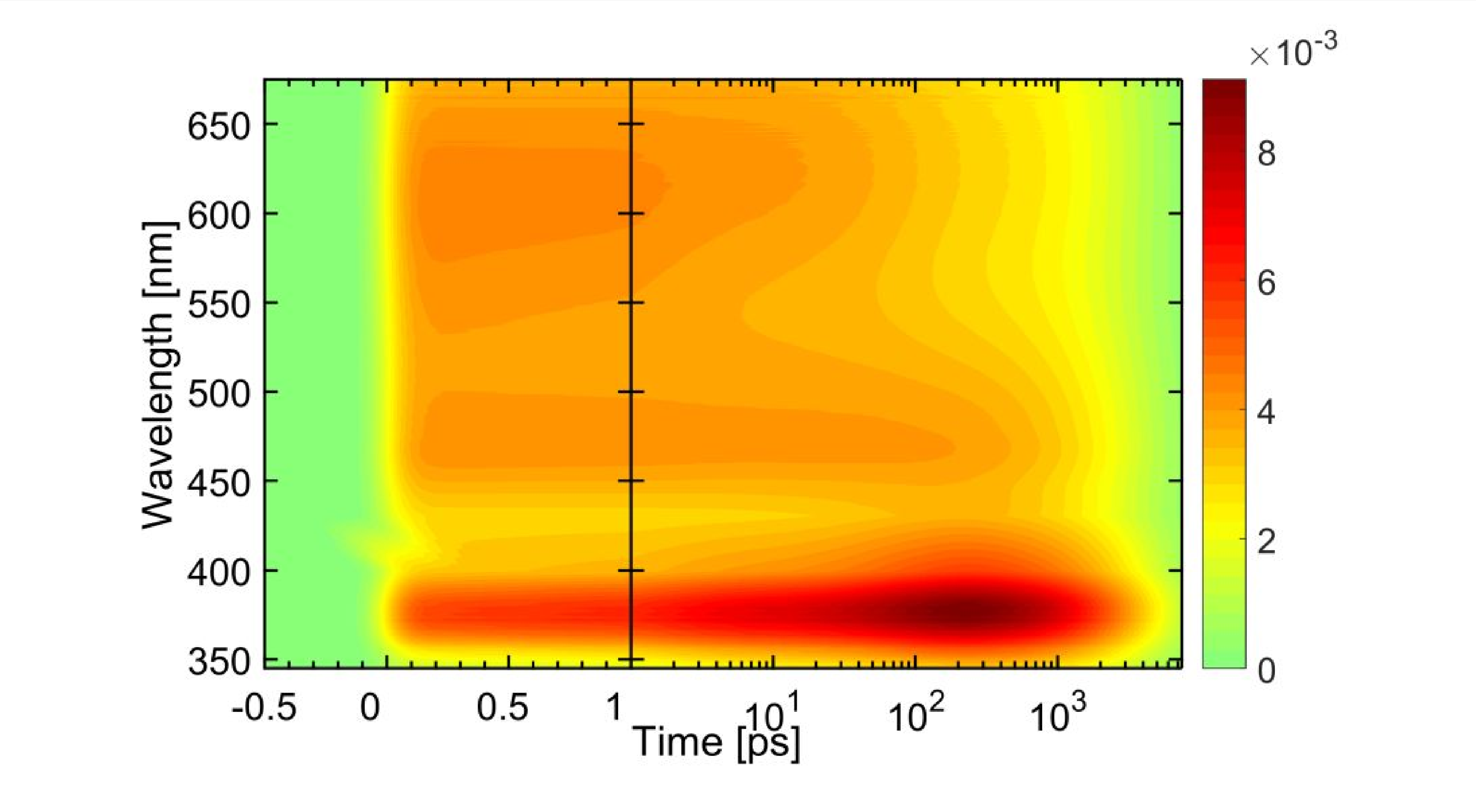

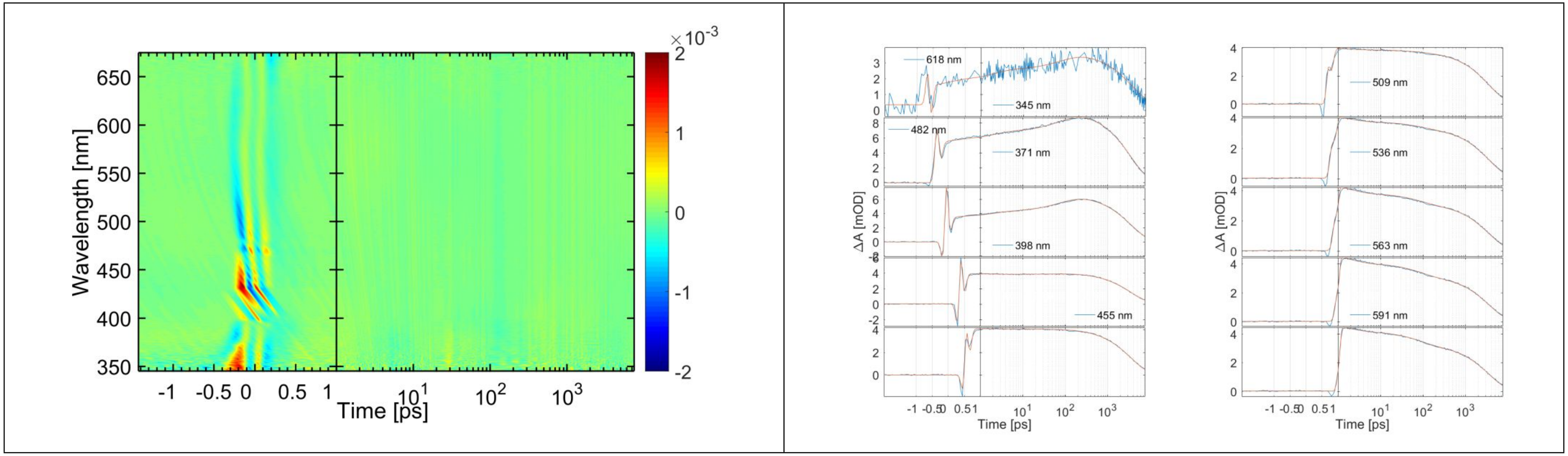

Wavelength [nm]
Time [ps]
×10-3
618 nm
345 nm
482 nm
371 nm
398 nm
455 nm
509 nm
536 nm
563 nm
591 nm
ΔA [mOD]

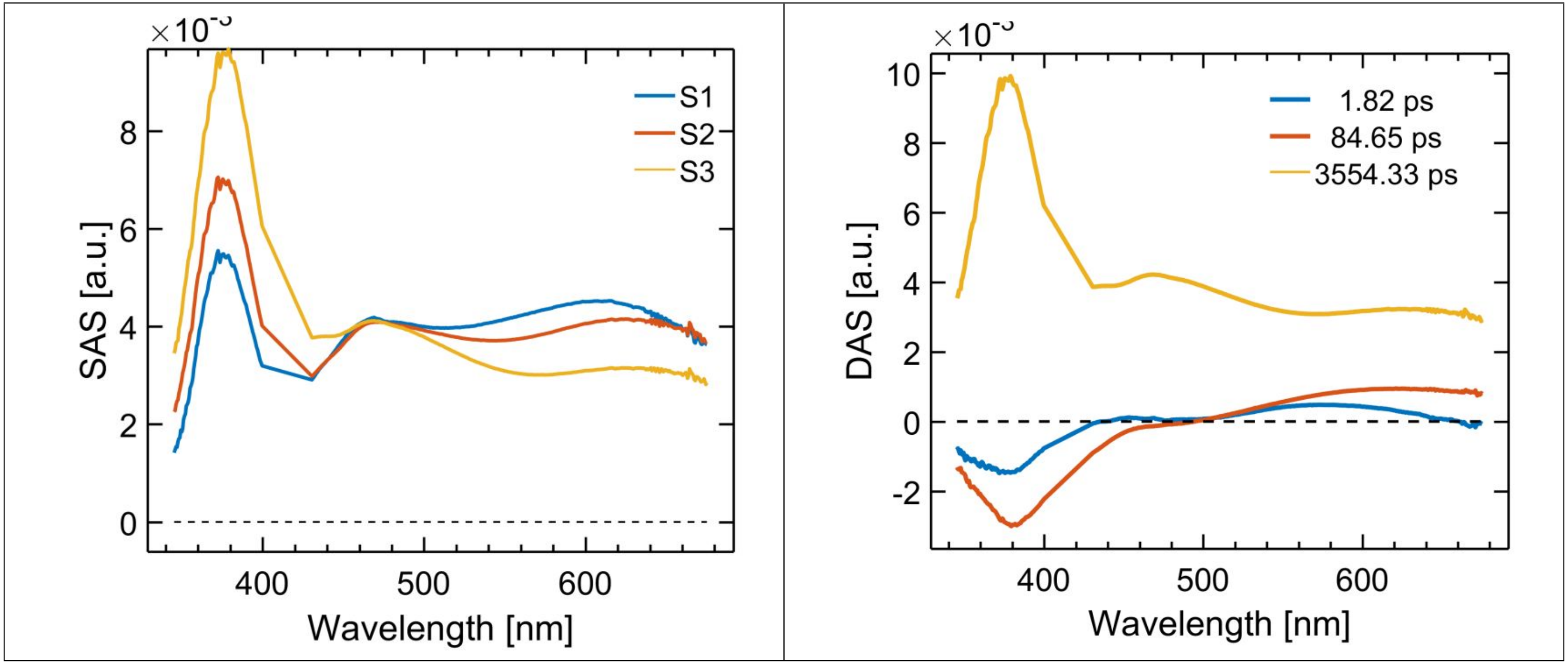

Figure S69. Summary of the global lifetime analysis of **1A** (pump 420 nm) containing 3D fsTA map, residual map, time traces at several wavelength, evolution associated spectra, decay associated spectra.

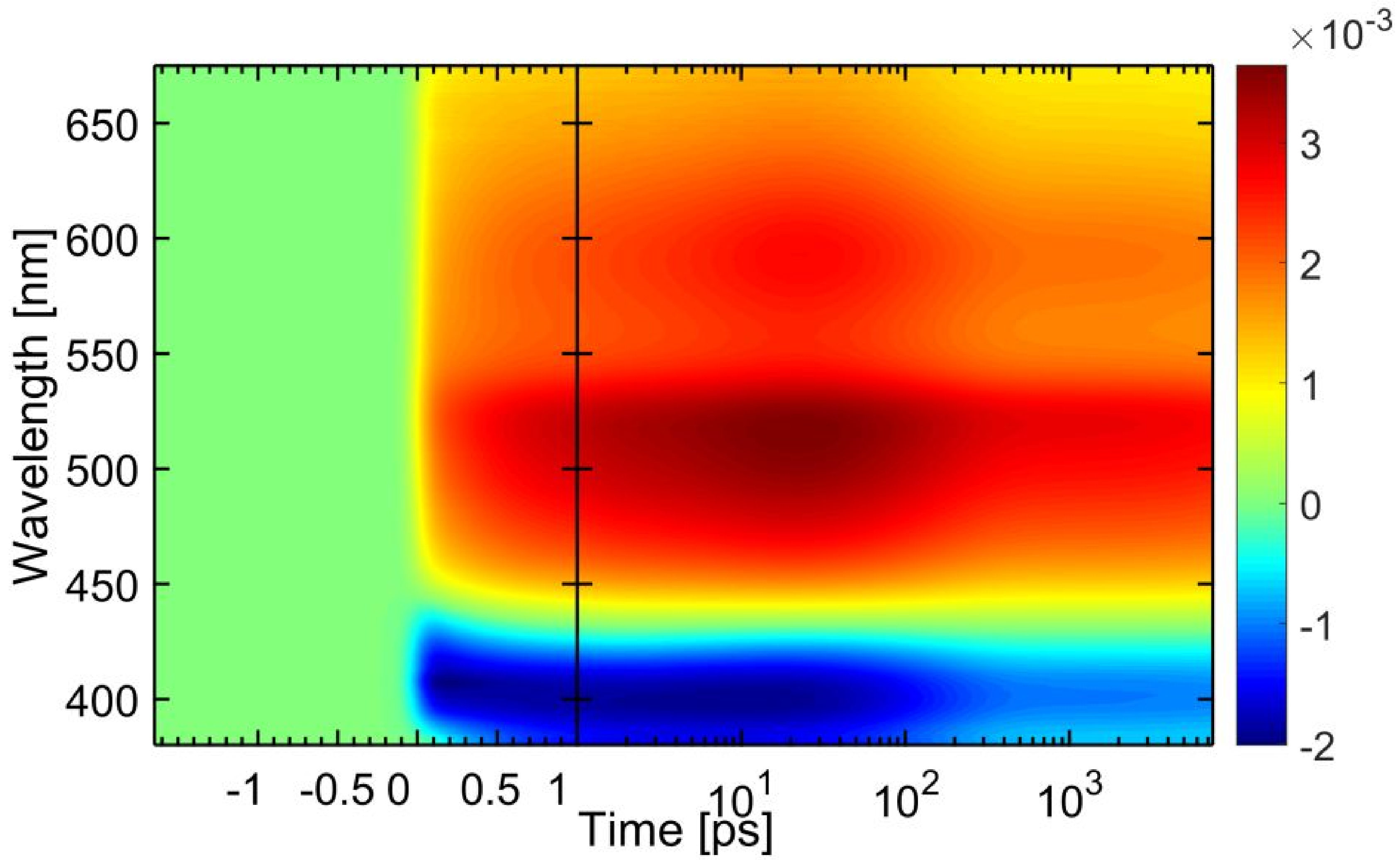

×10-3
3
2
1
0
-1
-2
650
600
550
500
450
400
Wavelength [nm]
-1
-0.5
0
0.5
1
10^1
10^2
10^3
Time [ps]

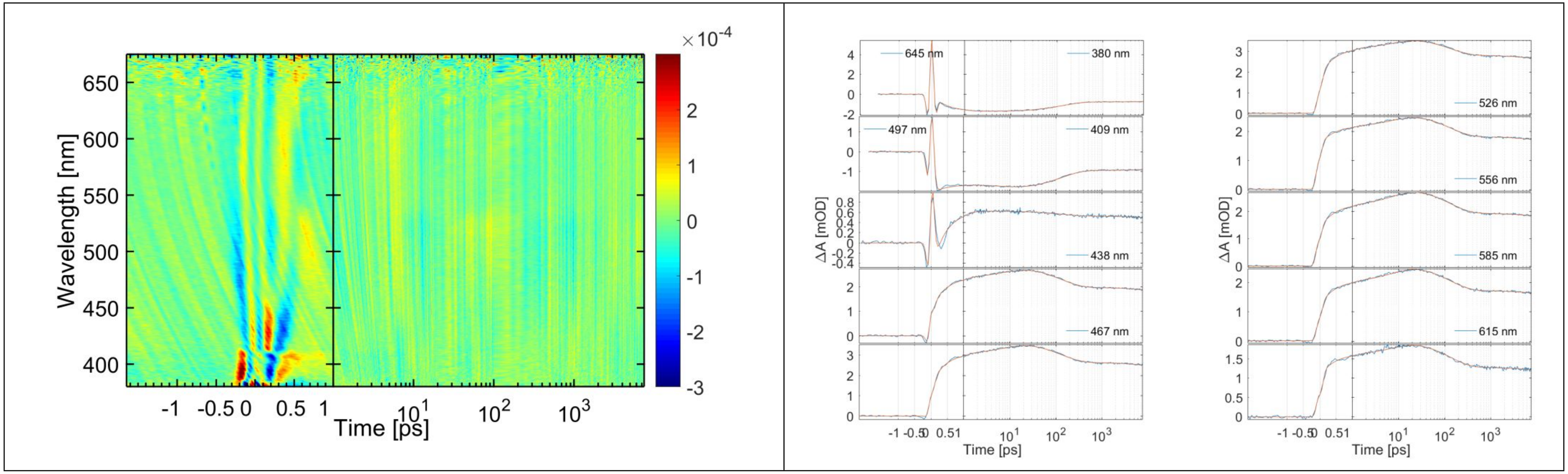
Wavelength [nm]
650
600
550
500
450
400
-1
-0.5
0
0.5
1
10^1
10^2
10^3
Time [ps]
$\times 10^{-4}$
2
1
0
-1
-2
-3
645 nm
380 nm
497 nm
409 nm
438 nm
467 nm
526 nm
556 nm
585 nm
615 nm
ΔA [mOD]
Time [ps]

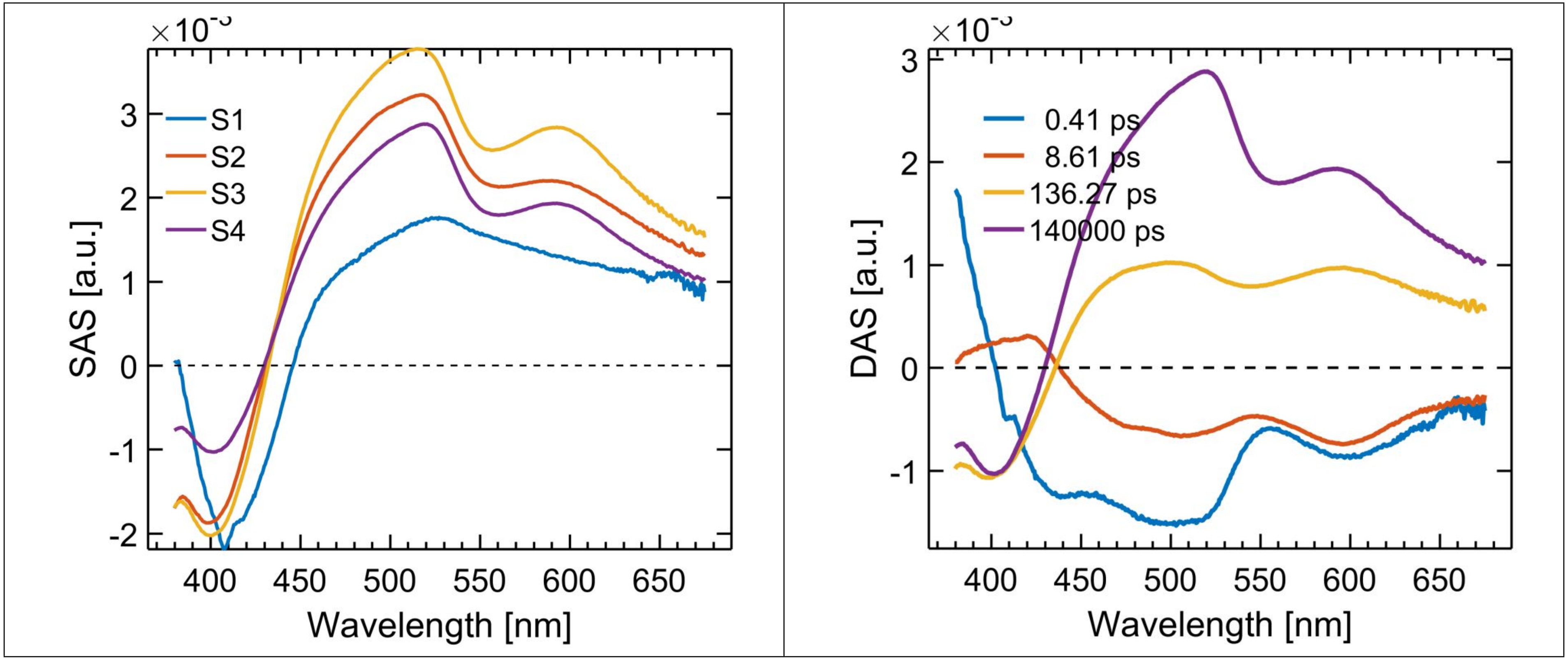

Figure S70. Summary of the global lifetime analysis of **4A** (pump 355 nm) containing 3D fsTA map, residual map, time traces at several wavelength, evolution associated spectra, decay associated spectra.

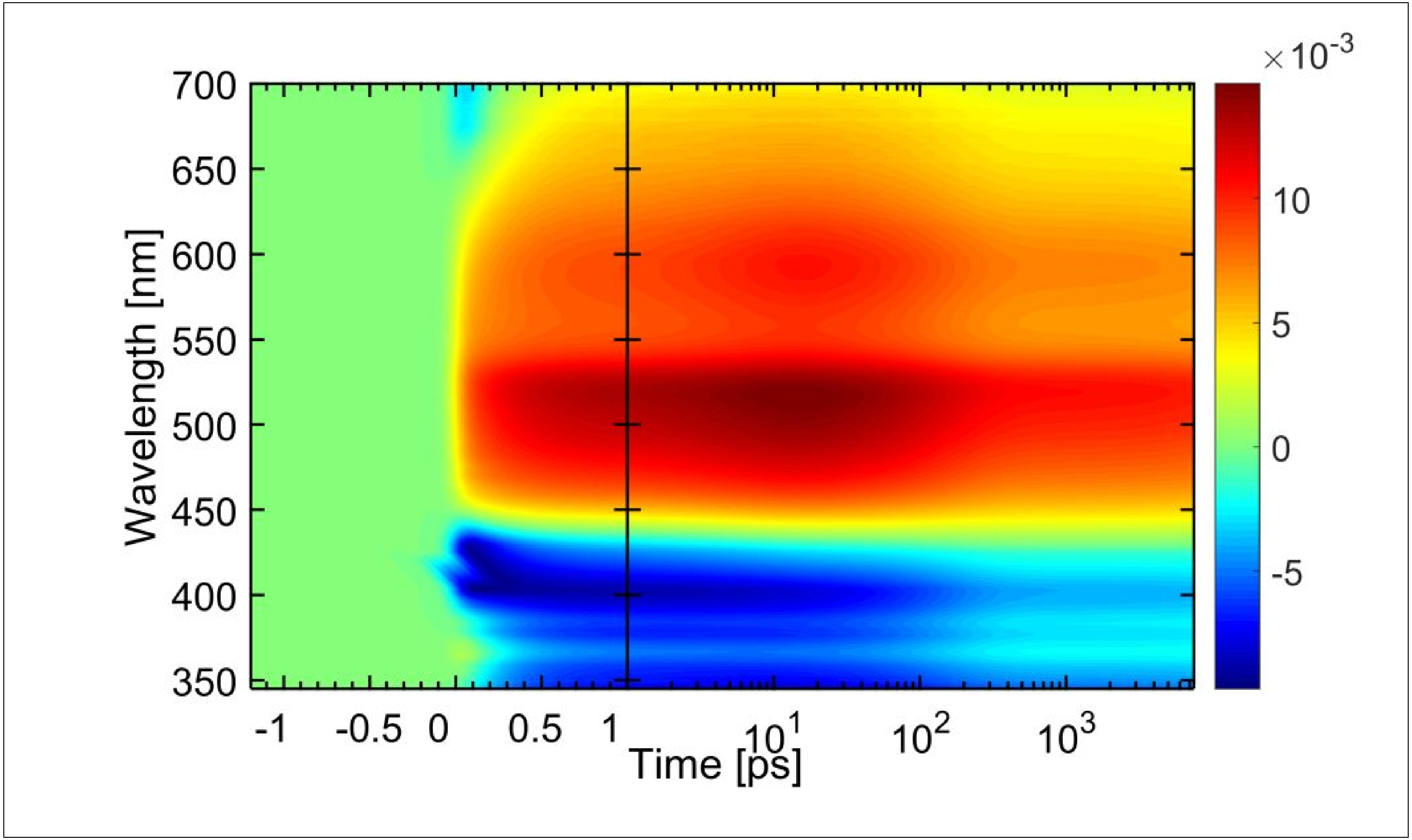

700
650
600
550
500
450
400
350
Wavelength [nm]
-1
-0.5
0
0.5
1
10^1
10^2
10^3
Time [ps]
$\times 10^{-3}$
10
5
0
-5

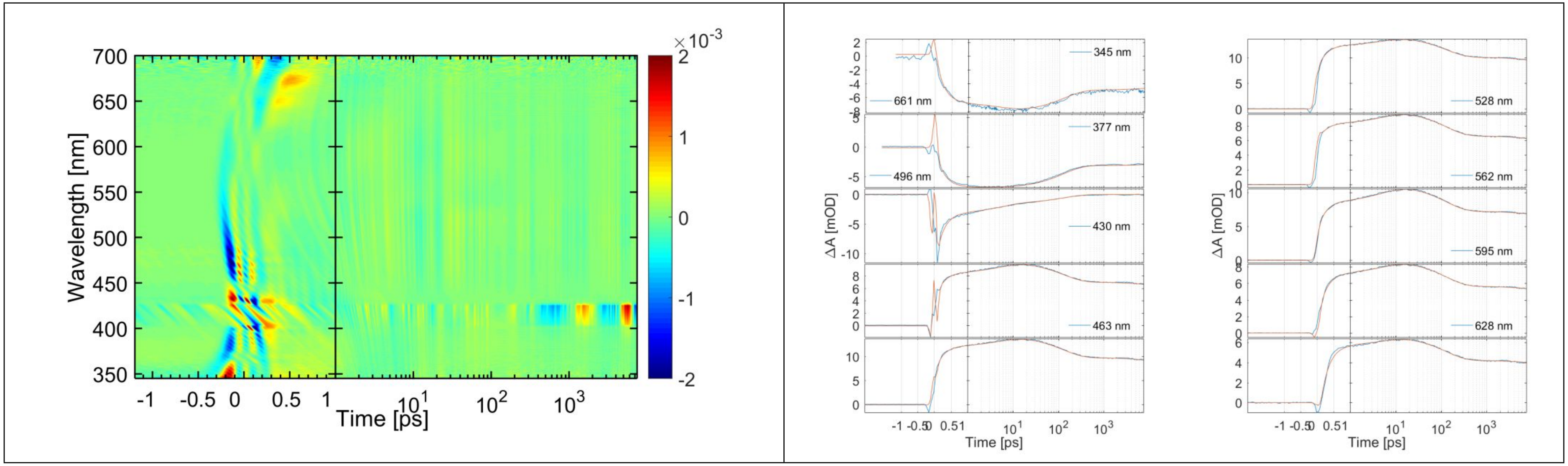
×10-3
Wavelength [nm]
Time [ps]
ΔA [mOD]
345 nm
661 nm
377 nm
496 nm
430 nm
463 nm
528 nm
562 nm
595 nm
628 nm

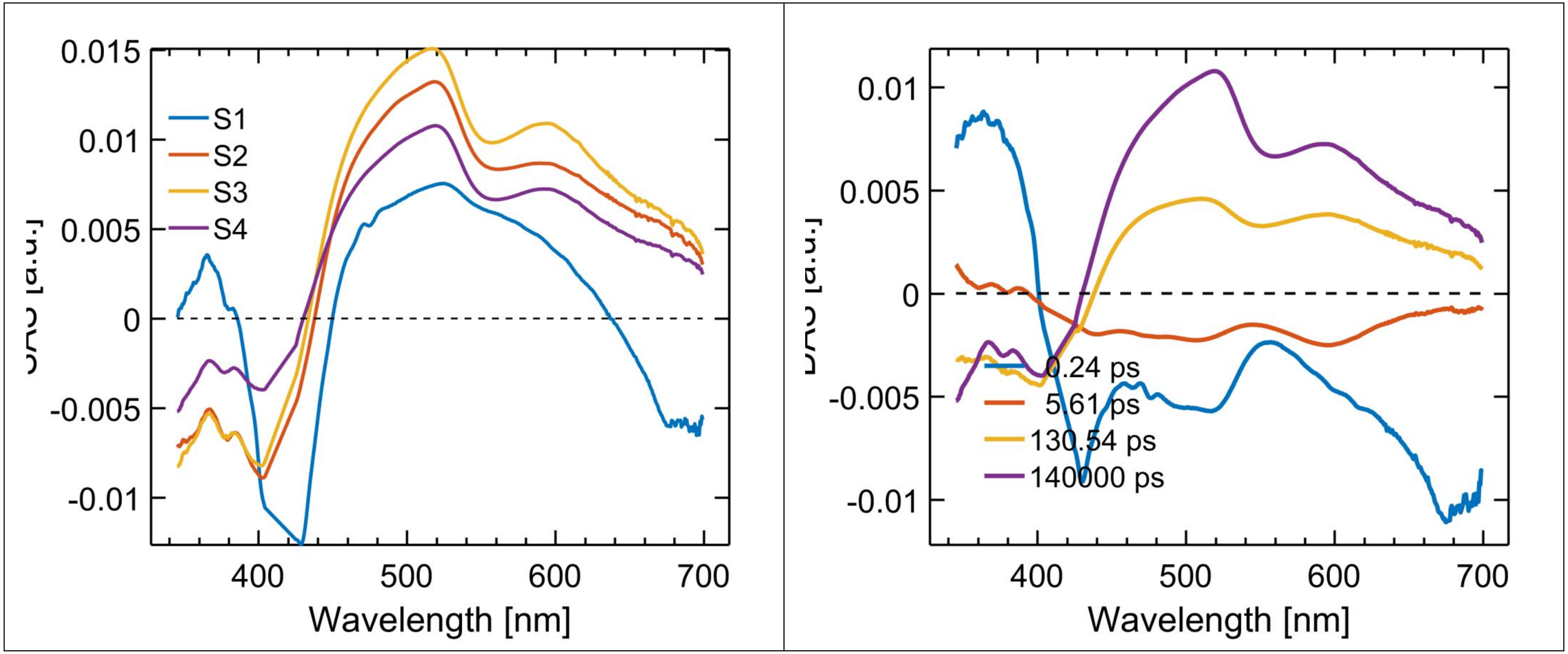

Figure S71. Summary of the global lifetime analysis of **4A** (pump 420 nm) containing 3D fsTA map, residual map, time traces at several wavelength, evolution associated spectra, decay associated spectra.

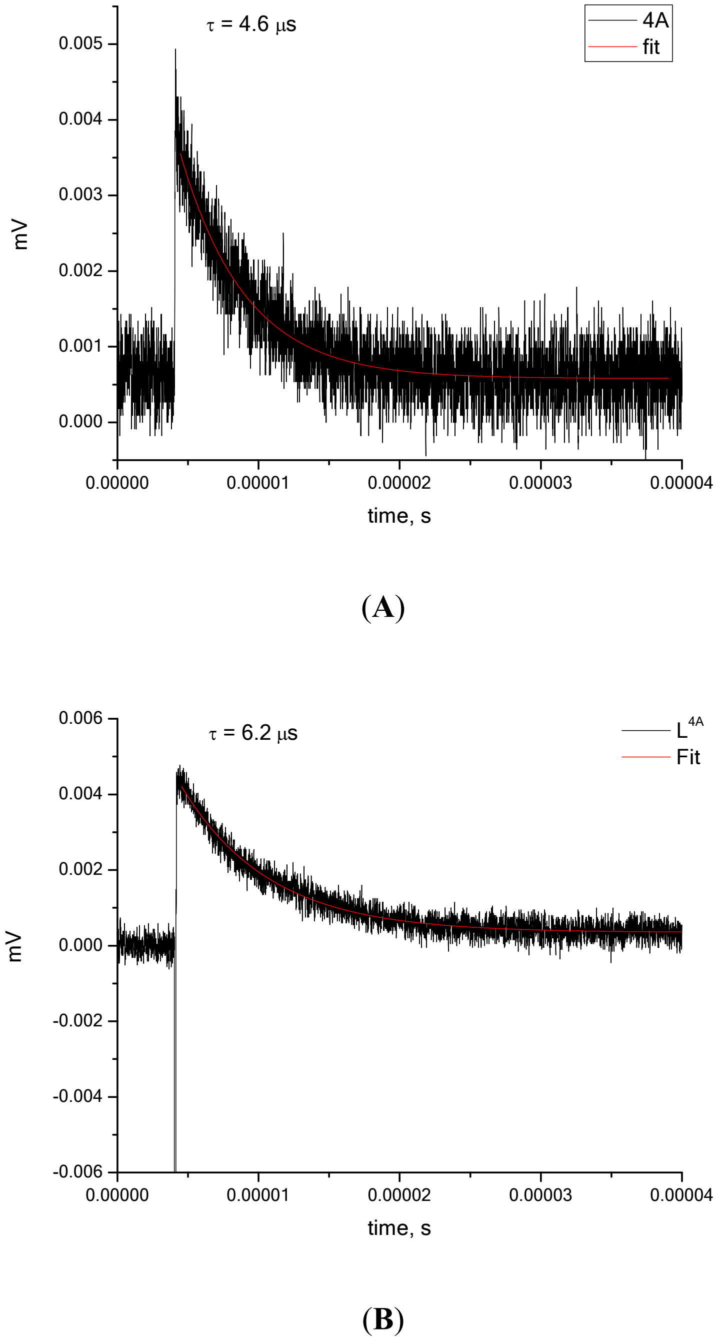

Figure S72. Fits of decay curves of triplet-triplet excited state absorption maxima of 4A (λ_{ESA} = 510 nm) (**A**) and L^{4A} (λ_{ESA} = 420 nm) (**B**).

References

1. CrysAlisRED,version1.171.37.35g, Oxford Diffraction Ltd., Abingdon, **2014**.
2. Sheldrick, G. M. Crystal structure refinement with SHELXL. *Acta Cryst.* **2015**, C71, 3–8.
3. Spek, A. L. PLATON SQUEEZE: a tool for the calculation of the disordered solvent contribution to the calculated structure factors. *Acta Crystallogr. Sect. C* **2015**, C71, 9-18.
4. Frisch, M. J.; Trucks, G. W.; Schlegel, H. B.; Scuseria, G. E.; Robb, M. A.; Cheeseman, J. R.; Scalmani, G.; Barone, V.; Petersson, G. A.; Nakatsuji, H.; Li, X.; Caricato, M.; Marenich, A. V.; Bloino, J.; Janesko, B. G.; Gomperts, R.; Mennucci, B.; Hratchian, H. P.; Ortiz, J. V.; Izmaylov, A. F.; Sonnenberg, J. L.; Williams-Young, D.; Ding, F.; Lipparin, F.; Egidi, F.; Goings, J.; Peng, B.; Petrone, A.; Henderson, T.; Ranasinghe, D.; Zakrzewski, V. G.; Gao, J.; Rega, N.; Zheng, G.; Liang, W.; Hada, M.; Ehara, M.; Toyota, K.; Fukuda, R.; Hasegawa, J.; Ishida, M.; Nakajima, T.; Honda, Y.; Kitao, O.; Nakai, H.; Vreven, T.; Throssell, K.; Montgomery, J. A.; Jr., Peralta, J. E.; Ogliaro, F.; Bearpark, M. J.; Heyd, J. J.; Brothers, E. N.; Kudin, K. N.; Staroverov, V. N.; Keith, T. A.; Kobayashi, R.; Normand, J.; Raghavachari, K.; Rendell, A. P.; Burant, J. C.; Iyengar, S. S.; Tomasi, J.; Cossi, M.; Millam, J. M.; Klene, M.; Adamo, C.; Cammi, R.; Ochterski, J. W.; Martin, R. L.; Morokuma, K.; Farkas, O.; Foresman, J. B.; Fox, D. J. Gaussian 16, Revision C.01, Gaussian, Inc., Wallingford CT, **2016**.
5. Adamo, C.; Barone, V. Toward reliable density functional methods without adjustable parameters: The PBE0 model. *J. Chem. Phys.* **1999**, 110, 6158-69.
6. Ernzerhof, M.; Scuseria, G. E. Assessment of the Perdew-Burke-Ernzerhof exchange-correlation functional. *J. Chem. Phys.* **1999**, 110, 5029-5036.
7. Weigend, F.; Ahlrichs, R. Balanced basis sets of split valence, triple zeta valence and quadruple zeta valence quality for H to Rn: design and assessment of accuracy. *Phys. Chem. Chem. Phys.* **2005**, 7, 3297-3305.
8. Rappoport, D.; Furche, F. Property-optimized Gaussian basis sets for molecular response calculations. *J. Chem. Phys.* **2010**, 133, 134105.
9. Andrae, D.; Häußermann, U.; Dolg, M.; Stoll, H.; Preuß, H. Energy-adjusted ab initio pseudopotentials for the second and third row transition elements. *Theor. Chim. Acta* **1990**, 77, 123-141.
10. Cancès, E.; Mennucci, B.; Tomasi, J. A new integral equation formalism for the polarizable continuum model: theoretical background and applications to isotropic and anisotropic dielectrics. *J. Chem. Phys.* **1997**, 107, 3032-3041.
11. Mennucci, B.; Tomasi, J. Continuum solvation models: a new approach to the problem of solute's charge distribution and cavity boundaries. *J. Chem. Phys.* **1997**, 106, 5151-5158.
12. Cossi, M.; Barone, V.; Mennucci, B.; Tomasi, J. Ab initio study of ionic solutions by a polarizable continuum dielectric model. *Chem. Phys. Lett.* **1998**, 286, 253-260.
13. Pedzinski, T.; Markiewicz, A.; Marciniak, B. Photosensitized oxidation of methionine derivatives. Laser flash photolysis studies. *Res. Chem. Intermed.* **2009**, 35, 497–506.

14. Maroń, A. M.; Choroba, K.; Pedzinski, T.; Machura, B. Towards better understanding of the photophysics of platinum(II) coordination compounds with anthracene- and pyrene-substituted 2,6-bis(thiazol-2-yl)pyridines. *Dalton Trans*. **2020**, 49, 13440-13448.
15. Slavov, C.; Hartmann, H.; Wachtveitl, J. Implementation and Evaluation of Data Analysis Strategies for Time-Resolved Optical Spectroscopy. *Anal. Chem*. **2015**, 87, 2328–2336.
16. Klemens, T.; Świtlicka, A.; Szlapa-Kula, A.; Krompiec, S.; Lodowski, P.; Chrobok, A.; Godlewska, M.; Kotowicz, S.; Siwy, M.; Bednarczyk, K.; Libera, M.; Maćkowski, S.; Pędziński, T.; Schab-Balcerzak, E.; Machura, B. Experimental and computational exploration of photophysical and electroluminescent properties of modified 2,2′:6′,2″-terpyridine, 2,6-di(thiazol-2-yl)pyridine and 2,6-di(pyrazin-2-yl)pyridine ligands and their Re(I) complexes. *Appl. Organometal. Chem*. **2018**, 32, e4611.
17. Maroń, A. M.; Szlapa-Kula, A.; Matussek, M.; Kruszynski, R.; Siwy, M.; Janeczek, H.; Grzelak, J.; Maćkowski, S.; Schab-Balcerzak, E.; Machura, B. Photoluminescence enhancement of Re(I) carbonyl complexes bearing D–A and D–π–A ligands. *Dalton Trans.* **2020**, 49, 4441–4453.
